# Next-generation genetic testing for retinitis pigmentosa

**DOI:** 10.1002/humu.22045

**Published:** 2012-02-14

**Authors:** Kornelia Neveling, Rob W.J. Collin, Christian Gilissen, Ramon A.C. van Huet, Linda Visser, Michael P. Kwint, Sabine J. Gijsen, Marijke N. Zonneveld, Nienke Wieskamp, Joep de Ligt, Anna M. Siemiatkowska, Lies H. Hoefsloot, Michael F. Buckley, Ulrich Kellner, Kari E. Branham, Anneke I. den Hollander, Alexander Hoischen, Carel Hoyng, B. Jeroen Klevering, L. Ingeborgh van den Born, Joris A. Veltman, Frans P.M. Cremers, Hans Scheffer

**Affiliations:** 1 Department of Human Genetics, Radboud University Nijmegen Medical CentreNijmegen, The Netherlands; 2 Department of Ophthalmology, Radboud University Nijmegen Medical CentreNijmegen, The Netherlands; 3 Nijmegen Centre for Molecular Life SciencesNijmegen, The Netherlands; 4 Institute for Genetic and Metabolic DiseaseNijmegen, The Netherlands; 5 Rotterdam Eye HospitalRotterdam, The Netherlands; 6 Augen Zentrum SiegburgSiegburg, Germany; 7 Department of Ophthalmology and Visual Sciences, University of MichiganAnn Arbor, Michigan

**Keywords:** NGS, DNA diagnostics, clinical molecular diagnostics, retinitis pigmentosa, blindness

## Abstract

Molecular diagnostics for patients with retinitis pigmentosa (RP) has been hampered by extreme genetic and clinical heterogeneity, with 52 causative genes known to date. Here, we developed a comprehensive next-generation sequencing (NGS) approach for the clinical molecular diagnostics of RP. All known inherited retinal disease genes (*n* = 111) were captured and simultaneously analyzed using NGS in 100 RP patients without a molecular diagnosis. A systematic data analysis pipeline was developed and validated to prioritize and predict the pathogenicity of all genetic variants identified in each patient, which enabled us to reduce the number of potential pathogenic variants from approximately 1,200 to zero to nine per patient. Subsequent segregation analysis and in silico predictions of pathogenicity resulted in a molecular diagnosis in 36 RP patients, comprising 27 recessive, six dominant, and three X-linked cases. Intriguingly, De novo mutations were present in at least three out of 28 isolated cases with causative mutations. This study demonstrates the enormous potential and clinical utility of NGS in molecular diagnosis of genetically heterogeneous diseases such as RP. De novo dominant mutations appear to play a significant role in patients with isolated RP, having major implications for genetic counselling.

## Introduction

Retinitis pigmentosa (RP; MIM# 268,000) is the most frequent subtype of inherited retinal disease and is clinically and genetically a highly heterogeneous disorder [den Hollander at al., 2010]. Fifty-two genes are known to be associated with nonsyndromic RP, involving all modes of inheritance [Berger et al., 2010]. A further 59 genes are known to underlie other subtypes of syndromic and nonsyndromic retinal diseases (RetNet; http://www.sph.uth.tmc.edu/Retnet/). With a few exceptions, there are no ophthalmologic characteristics specifically associated with the genetic subtypes of RP, impeding the prioritization of genes for analysis by Sanger sequencing.

Molecular diagnostics is particularly challenging for isolated RP patients, who constitute majority of RP cases [van den Born et al., 1992; Najera et al., 1995; Hayakawa et al., 1997]. Because of the unknown mode of inheritance, mutations in any of the 52 known RP genes may be causative. The most widely applied diagnostic test for allelic and genetic heterogeneous diseases, arrayed primer extension (APEX) chip technology, is only able to detect known mutations [Ávila-Fernández et al., 2010]. In addition, these chips are designed to separately test for the presence of mutations in autosomal dominant or recessive RP genes, resulting in a diagnostic yield for autosomal recessive RP of only ∼10% [Ávila-Fernández et al., 2010]. Altogether, the yield of diagnostic testing has remained disappointingly low for RP patients, despite many important disease gene discoveries in the last two decades [Berger et al., 2010].

In this study, we analyzed all known inherited retinal dystrophy (RD) genes in parallel by targeted next-generation sequencing (NGS) in a cohort of 100 RP patients. All identified genetic variants entered a systematic data analysis pipeline that included Sanger sequencing validation, segregation analysis, and a bioinformatic prediction of pathogenicity.

## Materials and Methods

### Clinical Diagnosis of RP

The diagnosis of RP was made in all individuals on the basis of ophthalmologic examination, including best-corrected visual acuity, slit-lamp biomicroscopy, ophthalmoscopy, and fundus photography. Electroretinograms, recorded according to the protocol of the International Society for Clinical Electrophysiology of Vision [Marmor et al., 2009], and Goldmann visual field measurements were available for majority of patients.

### Previous Genotyping and Patient Ascertainment

A cohort of 234 RP patients had been collected over a period of 15 years (Supp. Fig. S1). Mutation screening by APEX analyses and Sanger sequencing had identified a molecular diagnosis in 20 patients [Cremers et al., 1998; den Hollander et al., 1999; Maugeri et al., 2000; den Hollander et al., 2001; Yzer et al., 2003; Klevering et al., 2004]. In 186 of the remaining probands, genome-wide homozygosity mapping had been performed, which had resulted in the identification of mutations in three novel autosomal recessive RP genes, *EYS* (MIM# 612,424), *C2ORF71* (MIM# 613,425), and *IMPG2* (MIM# 607,056) [Collin et al., 2008; Bandah-Rozenfeld et al., 2010; Collin et al., 2010] in 13 patients, together with 24 homozygous mutations in previously known RD genes [Collin et al., 2011]. From the remaining 177 probands, 100 were selected for the targeted NGS analysis. This selection was based on the availability of DNA samples from both patients and their family members. It included 78 cases with nonsyndromic isolated RP and 22 cases with autosomal recessive RP (e.g., unaffected parents and two or more affected siblings), all without a molecular diagnosis. No apparent dominant RP cases were present in the cohort. Affected and nonaffected relatives were either included prior to this study or requested to participate after the identification of potentially causative variants in the proband. Some patients were clinically re-evaluated upon identification of potentially causative genetic variants.

After explaining the nature of this study, informed consents adhering to the tenets of the Declaration of Helsinki were obtained from all patients and their relatives. DNA was extracted from peripheral blood using standard procedures [Miller et al., 1988].

In addition to the 100 selected patients, DNAs from 12 cases with various types of autosomal recessive retinal diseases, carrying known compound heterozygous mutations in genes represented on the NGS array (Supp. Table S1) were investigated to design and optimize the current approach.

### Array-Based Sequence Capture and Targeted Resequencing

**Figure 1:**
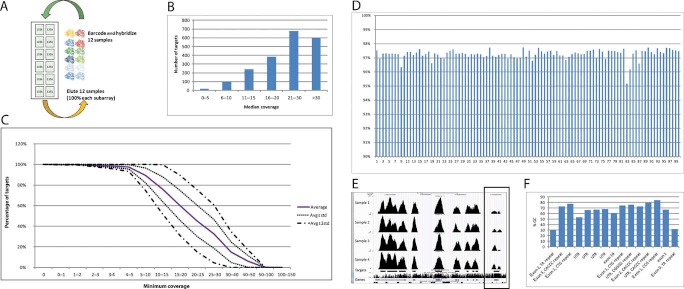
Sequencing statistics. A: Schematic drawing of a 12-plex 2.1M NimbleGen sequence-capture array. Samples were barcoded and hybridized individually. After hybridization, all 12 samples were eluted simultaneously, allowing to proceed with one pooled sample consisting of 12 different DNAs. B: Histogram of median target coverage. Only 15 targets were covered less than five times. Most targets show a coverage of 20–30×. C: The minimum coverage of a percentage of targets. Solid line indicates the average across 100 samples, whereas dotted and dash lines indicate first and second standard deviation respectively. D: Evenness of coverage for all samples. The average evenness score over all samples is 97.3%. E: Example of a poorly covered target. This screenshot shows the coverage of some exons of *GRM6*. The box highlights exon 1, which is poorly covered in comparison with the rest of the gene. F: The 15 targets that are poorly covered (median coverage of less than five times) have either a high GC content (often an UTR or exon 1) or are containing repeat-rich regions.

To enrich multiple DNAs in a single procedure, a 12-plex NimbleGen sequence-capture array (Roche NimbleGen, Madison, WI) consisting of 12 subarrays of 135K oligonucleotides was used (Fig. 1A). Probes targeting all coding exons, noncoding exons, and untranslated regions of 111 known blindness genes were included on these arrays, as well as probes for a fragment of intron 26 of *CEP290* (MIM# 610,142) that harbors a frequent mutation causing Leber congenital amaurosis (MIM# 204,000; Supp. Table S2). The *RPGR* (MIM# 312,610) exon ORF15 was not included because of highly repetitive regions hampering enrichment and sequencing. The array design comprised a total of 723,662 bases targeting 111 genes consisting of 2,011 individual regions (each individual target region mostly targets the sequence of a known exon and is at least 250 bp long). Sequence capture was performed following the “Titanium Optimized Sequence Capture Array Delivery” protocol (version 1.0), as supplied by Roche and optimized for sequence capture by NimbleGen arrays. Minor changes were made to adapt this protocol (for 385K arrays) to the 12-plex format. In brief, 5 μg of genomic DNA per sample were used in the preparation of DNA for sequence-capture hybridization. DNA was sheared using the Covaris S2 system (Covaris Inc., Woburn, MA) according to the instructions by the manufacturer for 500 bp fragments. Molecular-identifier-adapter ligation was performed as described in Technical Bulletins TCB #004-2009 and TCB #005-2009 (Roche NimbleGen). After pre-ligation-mediated polymerase chain reaction (LM-PCR), a final mass of 1.125 μg prepared sample and 35.5 μg Cot1 DNA was co-hybridized to each subarray. The reagent volume used for hybridization was reduced in proportion to the smaller loading volume of the 12-plex format (6 μl). Samples were eluted 72 hr after hybridization and subsequently amplified by post LM-PCR, resulting in a 12-plex sequencing library. Small-volume emulsion PCRs (emPCRs) were performed for each library using four different DNA concentrations as input to generate a titration curve, to determine the optimal input of DNA for each run. Based on that titration curve, a large-volume emPCR was performed and sequencing of each library was carried out on Roche GS FLX (454 Life Sciences, Branford, CT) sequencer with Titanium series reagents.

### Assembly and Variant Calling

Sequence reads were mapped against the human reference genome (hg18) using the Roche Newbler software (version 2.3; 454 Life Sciences). Signal processing parameters were changed to use less stringent quality filtering. Additional mapping and coverage statistics were extracted from the mapping output files using custom software. Variations were considered as high-quality differences when either (1) the variation was sequenced in at least three nonduplicate reads including at least one forward and one reverse read, or (2) the variation was seen in at least five reads with quality scores greater than 20.

Variants were annotated using a custom annotation pipeline adding the following annotation for each detected variant: annotation with known polymorphism data from dbSNP130, genomic feature annotation (e.g., exon and intron), amino acid translations, and PhyloP conservation score (based on conservation of 44 vertebrate species) [Hoischen et al., 2010; Vissers et al., 2010]. Additionally, variants were annotated with frequencies of the variants in (1) all 100 blindness samples, (2) an internal database of 86 exomes, (3) an internal database with known pathogenic blindness mutations, and (4) an internal database with mutations from the professional version of the Human Gene Mutation Database (HGMD).

Nucleotide numbering reflects cDNA numbering with +1 corresponding to the A of the ATG translation initiation codon in the reference sequence, according to the journal guidelines (www.hgvs.org/mutnomen). The initiation codon is codon 1.

### Quality Control and Coverage

Coverage was calculated by counting the number of sequenced bases mapping to the target regions. Bases mapping to regions within a 500 bp range of a target were considered “near target.” From this, the average coverage across all the regions was calculated for each sample (Supp. Tables S3A and S3B). To evaluate the performance of targets, we calculated the average coverage of each target across all 100 samples (Supp. Table S2). Evenness of coverage was calculated for all samples according to Mokry et al. [2010] (Fig. 1D and Supp. Tables S3A and S3B).

### Prioritization of Variants

Variants were selected for follow-up when all of the following criteria applied:

1. Variants were either nonsynonymous variants or splice site variants (8 bp splice acceptor, 20 bp splice donor).
2. Variants were not included in dbSNP130 unless they were reported in HGMD or were previously defined as a pathogenic blindness mutation.
3. Variants did not occur with the 86 in-house exomes with a frequency greater than 5%.
4. Variants did not occur with a frequency of more than 15% within all 100 blindness samples.
5. Variants were covered by at least 10 reads with at least 20% variation reads or at least 5 reads with 80–100% variation reads.
6. Variants were consistent with the known pattern of inheritance of the respective gene (i.e., homozygous/compound heterozygous or heterozygous).

If prioritization resulted in a single strong candidate allele (a nonsense or canonical splice site variant) in a recessively acting gene, a manual search for a second variant allele was performed in the unfiltered variant set. Variants that were selected for further analysis were validated using Sanger sequencing and analyzed in all available relatives.

### Data Validation and Segregation Analysis

Variant validation was performed using conventional Sanger sequencing. Dependent on the selection criteria described above, between one and 17 variants per sample were selected for validation (on average, 3.6 variants per sample; Supp. Tables S3A and S3B), leading to a total of 359 validated variants in the cohort of 100 samples. Where available, DNAs from additional family members were sequenced to enable segregation analysis.

### Determination of Pathogenicity of Variants

To systematically determine the pathogenicity of genetic variants, segregating variants were sorted based of the type of variation. Nonsense, frameshift, and canonical splice site variants were considered to be pathogenic. Based on existing guidelines [Bell et al., 2007], we developed a classification system for missense changes as well as for noncanonical splice-site variants. Evidence was based on (1) in silico evidence and (2) co-occurrence of two mutations in a recessive gene. With regard to in silico evidence, three different features were considered important: (a) missense or splice-site prediction software (acting on amino acid level), (b) evolutionary conservation (acting on nucleotide level), and (c) population frequencies (Supp. Fig. S2).

#### Missense/splice-site prediction tools

In the case of missense prediction programs (SIFT [Ng & Henikoff, 2003], PolyPhen [Adzhubei et al., 2010], MutPred [Li et al., 2009]), the different scores for the three different tools were combined by a majority vote resulting in a single classification as “pathogenic,” “unknown,” or “benign.” For the splice-site prediction programs (Splice Site Finder, MaxEntScan, NNSplice), wild-type score (wt) and mutation score (mut) were compared using the following cutoffs. Splice-site finder: if wt > 50 and mut < 50, then the prediction is “pathogenic.” If the difference between wt and mut is >5, then the prediction is “unknown,” otherwise the prediction is “benign.” MaxEntScan: if the difference between wt and mut is >0.8, then the prediction is “pathogenic.” If there is no difference between wt and mut, then the prediction is “benign,” else the prediction is “unknown.” NNSplice: if wt > 0.5 and mut < 0.5, then the prediction is “pathogenic.” If the difference between wt and mut is >0.05, it is “unknown,”; otherwise it is “benign” (see Supp. Fig. S2). The different scores for the three different tools were combined by a majority vote, resulting in a single classification as “pathogenic,” “unknown,” or “benign.”

#### Evolutionary conservation

For a classification based on evolutionary conservation, all variants with a PhyloP (44 vertebrate species) score of less than 1 were considered “benign,” all variants with a PhyloP value above 2.5 were considered to be “pathogenic,” and variants with intermediary values were classified as “unknown” (cutoffs were based upon a comparison of evolutionary conservation scores of dbSNP [build 130] and HGMD, as described in Vissers et al. [2010]).

#### Population frequencies

We established the frequencies of variants within 86 whole-exome sequencing samples of patients with unrelated disorders, as well as in the 100 RP samples. Variants found at a frequency greater than 3% in the exome samples were classified as “benign,” and variants with an exome frequency between 1% and 3% or variants with a frequency of 3% or more in the 100 blindness samples were classified “unknown.” The remaining variants were classified as “pathogenic.” The three classifications (i.e., prediction tools, conservation, and frequency) were combined according to the following rules: If the prediction based on frequencies was “benign,” then the final classification of the variant was “probably benign” regardless of the other predictions. In all other cases, the three predictions were combined into a single prediction using a majority vote. In case of three different votes, the variant was predicted as “unknown.”

As a result, all segregating (pairs of) variants were classified as either probably pathogenic, unknown, or benign.

### Biostatistical Analyses

The diagnostic performance in this study was evaluated by calculating the mutation detection rate as a percentage of mutations detected for the cohort of 12 patients with 24 known mutations, and by calculating the diagnostic yield as the percentage of patients for which a molecular diagnosis was obtained (for details, see *Results*).

## Results

### Targeted Resequencing of 111 RD Genes

This study has rigorously tested the power of large-scale targeted resequencing to perform molecular genetic analysis for RP, one of the most genetically heterogeneous human diseases. The coding regions of 111 RD genes were enriched by target capture and screened for mutations by NGS in 112 subjects—12 RD patients carrying two known heterozygous mutations in one of the retinal disease genes and 100 RP patients without a molecular diagnosis. The average amount of mappable sequence data per sample was 31 Mb, resulting in an evenly distributed average coverage of 26× per exon per sample (evenness score = 97.3%; Figs. 1B–1D). On average, 89% of all bases were covered at least 10×. Among the 2,011 targeted exons, only 15 exons (0.7%) were covered poorly (less than five times on average), either due to high GC contents or the abundant presence of repetitive sequences (Figs. 1E and 1F).

### Development and Application of a Variant Prioritization Pipeline

**Figure 2:**
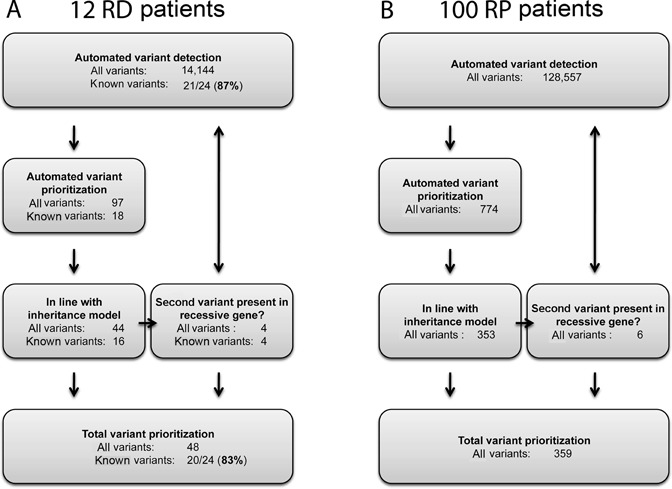
Systematic variant prioritization tool to efficiently reduce the number of identified variants. A: Filtering of identified variants in the 12 samples, each carrying two variants in recessive RD genes. 14,144 genetic variants were automatically detected, including 21 of the 24 known mutations. A further systematic prioritization (described in *Material and Methods*) reduced the number of variants to 97, including 18 of the 24 known pathogenic mutations (step 2). Of these 97 variants, all variants were selected that were consistent with the known inheritance pattern of the respective gene, resulting in 44 remaining variants, including 16 of the known variants (step 3). A manual search for a variant on the second allele was performed for recessive genes where only one variant was found (step 4), resulting in a total number of 48 variants, now including 20 of the 24 known mutations (step 5). B: In the group of 100 RP samples with unknown cause of disease, in total 128,557 variants (including six larger deletions) were automatically annotated (step 1). After applying the same filtering steps as for the 12 known RD samples, 359 variants remained after prioritization (step 5). RD, retinal dystrophy.

Automated variant detection for all 112 samples resulted in an average of 1,274 variants per sample (Supp. Tables S3A and S3B). A systematic variant prioritization tool was developed to identify the pathogenic mutation(s) amongst this substantial number of variants. To assess and optimize the performance of this methodology, 12 retinal disease samples, each carrying two known mutations, were analyzed. In these 12 patients, a total of 14,144 genetic variants were automatically detected, including 21 of the 24 known mutations (Fig. 2A), resulting in a technical mutation detection rate of 87.5%. Two of the three mutations that were not detected were located in an exon with an extreme high GC content (exon 1 of *GRM6*; MIM# 604,096), included in this study with the explicit purpose of determining whether the approach can deal with extreme GC content. Systematic filtering of variants was accomplished as described in *Materials and Methods* and is summarized in Fig. 2A. Filtering reduced the total number of putative pathogenic variants from 14,144 to 48 (99.7% reduction), whereas only one of the known causative variants was eliminated during the course of the filtering. Hence, filtering enriched the percentage of disease-causing mutations from 0.15% (21/14,144) to 41.6% (20/48).

The same systematic prioritization process was subsequently applied to all variants in the molecularly undiagnosed RP cohort of 100 patients. In total, 128,557 variants (including six large deletions) were identified (Fig. 2B). After applying the same filtering steps as for the 12 known RD samples, 359 variants remained (Fig. 2B), achieving the same reduction of putative causative variants of 99.7% (from 128,557 to 359).

### Confirmation of Variants and Segregation Analysis

Systematic Sanger sequencing of all 359 variants confirmed 283 variants (79%) in the total RP cohort. The majority of nonconfirmed variants (76%) were falsely called indels, many in the vicinity of homopolymer stretches, a known pitfall of the Roche 454 pyrosequencing technology. On the other hand, a number of larger homozygous and heterozygous deletions (≥43 bp) were detected and validated by Sanger sequencing, underlining an advantageous feature of the Roche long-read technology. Subsequently, all confirmed variants in a given sample were sequenced in available samples from relatives (Supp. Fig. S3).

### Interpretation of Genetic Variants

**Figure 3:**
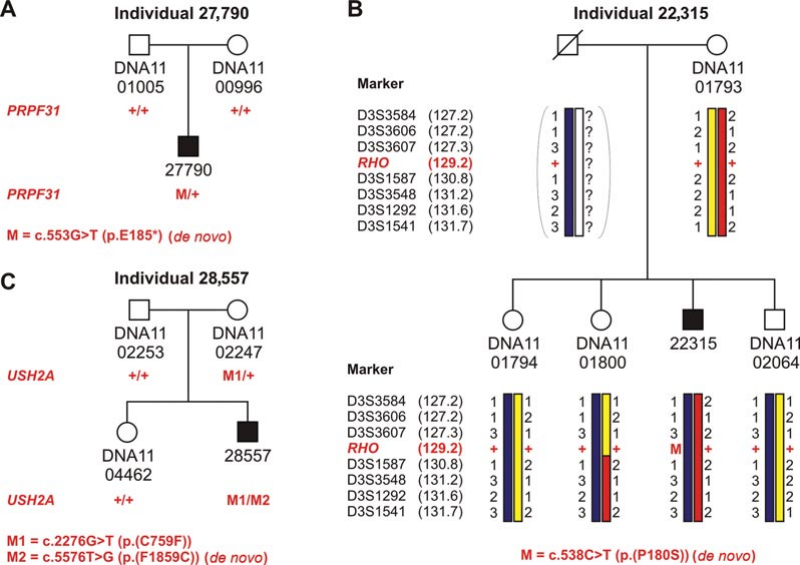
De novo mutations in isolated RP patients. A: De novo mutation in *PRPF31*. In patient 27,790, a heterozygous nonsense mutation was detected in the autosomal dominant RP gene *PRPF31* that was not present in both the unaffected parents. To confirm that individuals DNA11-01005 and DNA11-00996 are the biological parents, 16 highly polymorphic markers distributed across the genome were analyzed, showing a perfect Mendelian inheritance for all markers, thereby confirming the de novo event (Supp. Fig. S4). B: De novo mutation in *RHO*. In patient 22,315, a heterozygous missense mutation was detected that was predicted to be pathogenic (indicated in red). The mutation was not present in three unaffected siblings and the unaffected mother. Because the unaffected father was deceased, seven microsatellite markers surrounding and in close proximity of *RHO* were analyzed to determine haplotypes and the likelihood of a de novo event. The proband 22,315 and his three siblings all appeared to have inherited the same *RHO* allele from the deceased father, strongly suggesting that the *RHO* mutation has occurred by a de novo event. The genomic position of the microsatellite markers and *RHO* is indicated between parentheses. C: De novo mutation in *USH2A*. In patient 28,557, compound heterozygous mutations were detected in the autosomal recessive RP gene *USH2A*. One mutation (M1) was inherited from the mother, but the second mutation (M2) was not present in both parents, and as such, had occurred by a de novo event. Again, parental testing confirmed that DNA11-02253 and DNA11-02247 were the biological parents of the patient (Supp. Fig. S4).

To further determine whether a variant might be pathogenic, a systematic classification scheme was developed (*Materials and Methods*; Supp. Fig. S2). In brief, classification of pathogenicity was performed using a combination of prediction programs, evolutionary conservation, and frequency data. This method was validated with 100% sensitivity on an independent set of 20 functionally proven pathogenic missense mutations (Supp. Table S4A), whereas it reached a specificity of 94% for a set of 36 putatively benign missense mutations (selected based on frequency data, Supp. Table S4B). This classification method suggested disease-causing variants in 39 of the 100 investigated families (Table 1, Supp. Table S5). After checking all putative pathogenic variants in the Exome Variant Server of the NHLBI Exome Sequencing Project (ESP) (http://evs.gs.washington.edu/EVS/, release version: v.0.0.10), two presumed mutations in dominant genes (c.1730C>A in *TOPORS* (MIM# 609507) and c.1724C>T in *GUCY2D* (MIM# 600179)), present in three of our families, were re-classified to ‘benign’ due to frequency data of these variants. Of the remaining 36 families, the pathogenicity was supported in 21 by a full segregation in available relatives. Of all 36 families, inheritance was recessive in 27 cases, X-linked in three cases, and dominant in six cases. In two samples from isolated cases (samples 22,315 and 27,790), disease-causing mutations in the dominant genes *RHO* (MIM# 180,380) and *PRPF31* (MIM# 606,419), respectively, occurred as de novo events (Figs. 3A and 3B). In addition, in one case (family 28,557), a de novo mutation in the recessive gene *USH2A* (MIM# 608,400) was identified, with the second mutation being inherited from the mother (Fig. 3C). For all three cases, parental testing proved paternity (Supp. Fig. S4). Additional de novo mutations in dominant genes are likely in two other cases (samples 17,792 and 31,035), although the absence of data from at least one parent hampers a definite conclusion.

**Table 1:** Diagnostic Results of 36 RP Patients with Validated Pathogenic Variants

Patient	Gene	Inheritance in gene	Inheritance in family	Validated mutation M1	Validated mutation M2
7,554	*ABCA4*	ar	Isolated female	c.1554G>A (p.[?])^a^	c.4254-2A>G (p.(?))^a^
8,625	*IMPG2*	ar	Familial recessive	c.379G>A (p.[R127^*^])^a^	c.3423-8_c.3423-5del (p.(?))^a^
9,437	*USH2A*	ar	Familial recessive	c.486-14G>A (p.[?])^a^	c.12729G>A (p.(W4243^*^))^a^
9,470	*RP2*	xl	Isolated male	c.323G>A (p.[C108Y])^b^	–
9,472	*PDE6B*	ar, ad	Familial recessive	c.1920+2T>C (p.[?])^a^	c.1920+2T>C (p.(?))^a^
	*PRPH2*	ad	Familial recessive	c.424C>T (p.[R142W])^c^	–
9,493	*PDE6B*	ar, ad	Isolated male	c.1043_1044insCG (p.[A349fs])^a^	c.1927_1969delinsGG (p.(N643fs))^a^
9,518	*RP1*	ar, ad	Familial recessive	c.515T>G (p.[L172R])^b^	c.515T>G (p.(L172R))^b^
13,480	*RPE65*	ar	Isolated male	c.208T>G (p.[F70V])^d^	c.1102T>C (p.(Y368H))^d^
15,569	*PRPH2*	ad	Familial female sibship	c.441del (p.[P147fs])^a^	–
17,597	*USH2A*	ar	Isolated male	c.10525A>T (p.[K3509^*^])^a^	c.[12343C>T;13274C>T] (p.[(R4115C);(T4425M)])^d^
17,792	*RHO*	ar, ad	Isolated female	c.403C>T (p.[R135W])^d^	–
18,060	*SPATA7*	ar	Isolated female	c.3G>A (p.[M1?])^a^	c.322C>T (p.(R108^*^))^a^
18,336	*PDE6B*	ar, ad	Isolated male	c.2326G>A (p.[D776N])^b^	c.1927_1969delinsGG (p.(N643fs))^a^
19,693	*PDE6B*	ar, ad	Isolated male	c.299G>A (p.[R100H])^b^	c.1927_1969delinsGG (p.(N643fs))^a^
19,733	*USH2A*	ar	Isolated female	c.(4957C>T; 7379G>A) (p.[(R1653^*^);(R2460H)])^a,b^	c.10073G>A (p.(C3358Y))^b^
19,735	*RPE65*	ar	Isolated female	c.271C>T (p.[R91W])^d^	c.715T>G (p.(Y239D))^d^
20,984	*PDE6B*	ar, ad	Isolated female	c.1107+3A>G (p.[?])^a^	c.1107+3A>G (p.(?))^a^
21,141	*CEP290*	ar	Isolated male	c.3559del (p.[L1187fs])^a^	c.4705-1G>T (p.(?))^a^
21,334	*CACNA1F*	xl	Isolated male	c.220T>C (p.[C74R])^d^	–
21,933	*RP1*	ar, ad	Isolated male	c.368_369dup (p.[P214fs])^a^	c.4241_4242del (p.(H1414fs))^a^
22,315	*RHO*	ar, ad	Isolated male	c.538C>T (p.[P180S])^b^	–
22,393	*PDE6B*	ar, ad	Isolated male	c.892C>T (p.[Q298^*^])^a^	c.892C>T (p.(Q298^*^))^a^
22,777	*USH2A*	ar	Isolated female	c.1256G>T (p.[C419F])^d^	c.[12343C>T;13274C>T] (p.[(R4115C);(T4425M)])^d^
27,790	*PRPF31*	ad	Isolated male	c.553G>T (p.[E185^*^])^a^	–
28,557	*USH2A*	ar	Isolated female	c.2276G>T (p.[C759F])^d^	c.5576T>G (p.(F1859C))^b^
31,035	*NR2E3*	ar, ad	Isolated male	c.95G>A (p.[W32^*^])^a^	–
31,124	*USH2A*	ar	Male sibship	c.486-14G>A (p.[?])^a^	c.2276G>T (p.(C759F))^d^
31,343	*USH2A*	ar	Familial recessive	c.917_918insGCTG (p.[S307fs])^a^	c.11007C>A (p.(Ser3669Arg))^b^
31,723	*ARL6*	ar	Isolated female	c.185+1G>A (p.[?])^a^	c.185+1G>A (p.(?))^a^
31,933	*CRB1*	ar, ad	Isolated male	c.1602G>T (p.[K534N])^b^	c.2234C>T (p.(T745M))^d^
31,994	*RHO*	ar, ad	Familial dominant	c.641T>A (p.[I214N])^b^	–
32,594	*NRL*	ar	Male sibship	c.508C>A (p.[R170S])^d^	c.654del (p.(C219fs))^a^
32,655	*RP2*	xl	Male sibship	c.318_319delAG (p.[D107fs])^a^	–
33,626	*USH2A*	ar	Isolated female	c.1227G>A (p.[W409^*^])^a^	c.12575G>A (p.(R4192H))^b^
33,672	*RDH12*	ar	Isolated male	c.658+591_^*^603delinsCT (p.[?])^a^	c.658+591_^*^603delinsCT (p.(?))^a^
37,370	*RLBP1*	ar	Isolated female	c.525_945del (p.[?])^a^	c.525_945del (p.(?))^a^

Patient, patient identifier; Gene, RefSeq gene name of the gene in which mutations were identified; Inheritance, possible inheritance of phenotype for known mutations in the corresponding gene; Validated mutation M1 and Validated mutation M2, cDNA and protein annotation of the identified mutations.

a Nonsense/splicing/frameshift mutation.

b Variant predicted to be pathogenic.

c Known mutation identified in proband, not present in other affected siblings, but contributing to a more severe phenotype in the proband.

d Known mutation.

ar, autosomal recessive; ad, autosomal dominant; xl, X-linked.

### Cumulative Effect of Multiple Pathogenic Alleles on the Phenotype

In sample 9,472, a homozygous canonical splice-site mutation in *PDE6B* (MIM# 180,072, c.1920+2T>C [p.(?)]) and a known (dominant) missense mutation in *PRPH2* (MIM# 179,605, c.424C>T [p.(R142W)]) [Boon et al., 2007] were identified. Although the homozygous splice-site mutation in *PDE6B* was segregating with the disease in four affected siblings, the dominant missense mutation was not present in any of the affected siblings. This may help to explain the earlier onset of macular abnormalities, which are known to be associated with *PRPH2* mutations, in patient 9,472; at age 20, a bull's eye maculopathy with significant abnormalities of the retinal pigment epithelium was observed, whereas his sibling 12,273 at age 22 did not show any significant macular abnormality.

### Diagnostic Yield for an Unscreened RP Cohort

The mutation detection rate for the current NGS approach was 87.5% for the 12 RD patients with 24 known mutations (21/24 = 87.5%). In 10 out of these 12 patients, this would have resulted in a molecular diagnosis, resulting in a solved rate (diagnostic yield) of 83% (10/12 = 83%).

We estimate that for an unscreened RP population, the diagnostic yield would be ∼50%, based on the following calculation. The original cohort of 234 patients had undergone previous selected genotyping, resulting in the identification of genetic defects in 57 patients (Supp. Fig. S1). As a result of a solved rate of 83% for the NGS-based approach in 12 control patients, 47 out of these 57 patients (83%) would have been solved when analyzed by NGS, assuming autosomal recessive inheritance. Of the remaining 177 patients, 100 were investigated by our approach, of which 36 were diagnosed (36%). The residual 77 samples (177–100) have so far not been analyzed by NGS. Assuming the same diagnostic yield of 36% with NGS in those, we would diagnose additional 28 patients out of these 77 (36%) with NGS. Together, our data indicate that this approach could be used to establish a molecular diagnosis in nearly 50% of a random RP cohort ([47 + 36 + 28]/234 = 47%).

Overall, the results of this study underscore the extreme genetic heterogeneity in RP by identifying putatively causative mutations in no fewer than 20 different genes (Supp. Fig. S5). Mutations in *USH2A* and *PDE6B* appear to be slightly overrepresented in the mutational spectrum, as has been described previously [Hartong et al., 2006]. In five patients that were initially diagnosed with RP (individuals 7,554, 9,437, 21,141, 21,334, and 31,723), identification of the genetic defect led to a reappraisal of the phenotype to either a different subtype of nonsyndromic RD or to a (mild) syndromic form of RP (Supp. Table S6). For the 64 cases without a clear molecular diagnosis, either no interesting variants were detected among the 111 targeted genes (eight cases), the identified variants were predicted to be benign or did not segregate completely (48 cases), or segregating variants were identified, but their pathogenicity remained uncertain (eight samples).

## Discussion

In this study, we developed a comprehensive diagnostic tool for RP, consisting of a massive parallel sequencing approach for all known retinal disease genes, with systematic analysis and interpretation of all detected genetic variants. Our interpretation workflow provides a general approach for the interpretation of the vast amount of data generated by NGS-based molecular diagnostics. The enormous potential and clinical utility of NGS is highlighted by a high diagnostic yield achieved by the identification of mutations of all modes of inheritance, including de novo mutations. The high degree of (inherited and de novo) autosomal dominant and X-linked mutations confers a significant risk for transmitting the disease to the patients' offspring, thereby illustrating not only the molecular diagnostic power of NGS, but also the huge impact of this method on the affected families.

The strength of this study lies in the parallel analysis of all known retinal disease genes in 100 RP patients with an unknown molecular cause of disease by NGS. Systematic variant prioritization reduced the initially high number of identified variants (∼1,200 per patient) by 99.7% to a number that is manageable to validate by Sanger sequencing (approximately four variants per patient). Combined with a systematic assessment of pathogenicity of the validated variants, this NGS approach resulted in a molecular diagnosis in no fewer than 36 patients. The usage of a completely unscreened, prospective cohort would probably have led to a more accurate estimation of the success rate of this approach. Although such prospective studies can be performed in a research setting, it is, however, impossible within DNA diagnostics, where every effort needs to be made to solve a sample in a timely fashion.

Importantly, the 100 patients investigated here originally belonged to a cohort of 234 patients that had previously undergone selected genotyping [Collin et al., 2011], resulting in the identification of genetic defects in 57 of the patients before the start of this study (Supp. Fig. S1). Taking into consideration a solved rate of 83%, we calculated a potential diagnostic yield of nearly 50% in RP for this NGS-based diagnostic approach (see *Results* for details), thereby outperforming previously used approaches such as traditional Sanger sequencing and APEX analysis [Hartong et al., 2006; Ávila-Fernández et al., 2010]. As the number of 12 samples used for finding known mutations is quite low and technology is still improving, we cannot rule out a certain variability of this given percentage.

Apart from the ability to analyze all known RP genes simultaneously, thus increasing effectiveness of the genetic analysis, this approach has added value and superior clinical relevance because it allowed the identification of a substantial number of cases with apparent de novo mutations, and it has the potential to identify additional mutations potentially contributing to the phenotype—that is, to determine the cumulative mutational load. The 100 investigated samples comprised 78 isolated cases and 22 cases from recessive multiplex families. Among the 22 multiplex families, a molecular diagnosis was achieved in eight families: seven families carried mutations in autosomal recessive RP genes and one carried a mutation in the X-linked gene *RP2* (MIM# 300,757). Of the 78 isolated RP cases, a molecular diagnosis was established in 28 cases, including 20 families that harbored mutations in autosomal recessive RP genes and two male cases with mutations in X-linked genes. In two presumed isolated cases, mutations were found in autosomal dominant RP genes, but re-evaluation of both families revealed that one of the parents also had RP. Intriguingly, de novo mutations were proven to be present in three further isolated cases. Two of these were located in dominant genes, whereas in one patient, one of the two autosomal recessive mutations in *USH2A* occurred as a de novo event. Additional de novo mutations are likely in two other cases because these detrimental variants occur in dominant genes, whereas no phenotypes are reported for the parents. However, the absence of DNA of at least one parent hampers a definite conclusion. Although de novo mutations have recently been shown to play a major role in human diseases with reduced reproductive fitness [Hamdan et al., 2009; Hoischen et al., 2010; Vadlamudi et al., 2010; Vissers et al., 2010], the identification of a significant fraction of de novo dominant mutations in RP is a surprising finding. We speculate that de novo mutations may have been underappreciated as a cause of autosomal dominant RP as the result of a bias in ascertainment towards sizeable families with a clear autosomal dominant inheritance pattern [Daiger et al., 2008]. Given a transmission risk of 50% and a recurrence risk of <1% for autosomal dominant de novo mutations, compared with a transmission risk of <1% and a recurrence risk of 25% for autosomal recessive inheritance, the relatively high number of de novo mutations in our cohort will have significant implications for genetic counseling of our patients and their relatives. An additional advantage of an NGS approach that targets all known RD genes is the unprecedented possibility to study the cumulative effect of multiple pathogenic alleles in different genes on the phenotype [Ng et al., 2010]. This could be observed in family 9,472, where the proband carried three disease-causing mutations in two different genes (one recessive and one dominant gene), whereas his four affected siblings carried only the mutations in the recessive gene. This led us to the conclusion that the additional mutation could explain the earlier onset of macular abnormalities in the proband when compared with his siblings. The characterization of the genetic defect, however, is not only important for understanding the patients' phenotypes, but also confers benefits concerning eligibility for gene therapy. Gene replacement therapy has been proven to be beneficial in clinical trials for patients with *RPE65* (MIM# 180,069) mutations [Bainbridge et al., 2008; Hauswirth et al., 2008; Maguire et al., 2008]. Furthermore, preclinical gene therapy studies in animal models are ongoing for a substantial set of RD genes [den Hollander et al., 2010] and are likely to enter clinical trials in near future.

Further improvements in sequencing technology together with the identification of novel RP genes will undoubtedly boost the success rate of NGS-based diagnostic approaches in RP in near future. Recently, others have also reported on the use of NGS for the identification of disease-causing mutations in genetically heterogeneous disorders, including that of dominant [Bowne et al., 2011] and recessive [Simpson et al., 2011] RP, although the number of genes that were analyzed and/or the cohort sizes were considerably smaller as compared with our study [Shearer et al., 2010; Jones et al., 2011; Otto et al., 2011]. An alternative for parallel, targeted resequencing of known disease genes is whole-exome or whole-genome sequencing [Choi et al., 2009; Lupski et al., 2010; Worthey et al., 2011], permitting a standardized laboratory workflow that can be used for any type of genetic disorder. We think that the diagnostic interpretation workflow developed in this study can also be applied to these approaches, especially given the fact that most diagnostic laboratories will focus on the interpretation and reporting of variants in the coding part of our genome. Importantly, three patients in this study were found to carry pathogenic deletions that were easily detected by the long reads of the Roche 454 sequencing technology used in this study. This stresses the fact that these indels represent a diagnostically important group of genome variants that is not robustly called by the most commonly used whole-exome or whole-genome sequencing approaches.

Together, these studies support our conclusion that NGS will become increasingly important for the identification of disease-causing genes, and underscore the impact of this technology for the future of DNA diagnostics. For this, improvements in sequencing performance and interpretation should be combined with further cost reductions and improvements in turnaround times.
