# Supplementary material for: Next-generation genetic testing for retinitis pigmentosa
Source: Hum Mutat. 2012 Feb 14;33(6):963–72. doi: 10.1002/humu.22045 (PMC3490376; doi:10.1002/humu.22045)
Supplement: Supplementary file 1 [file humu0033-0963-sd1.pdf]

Supp. Figure S1

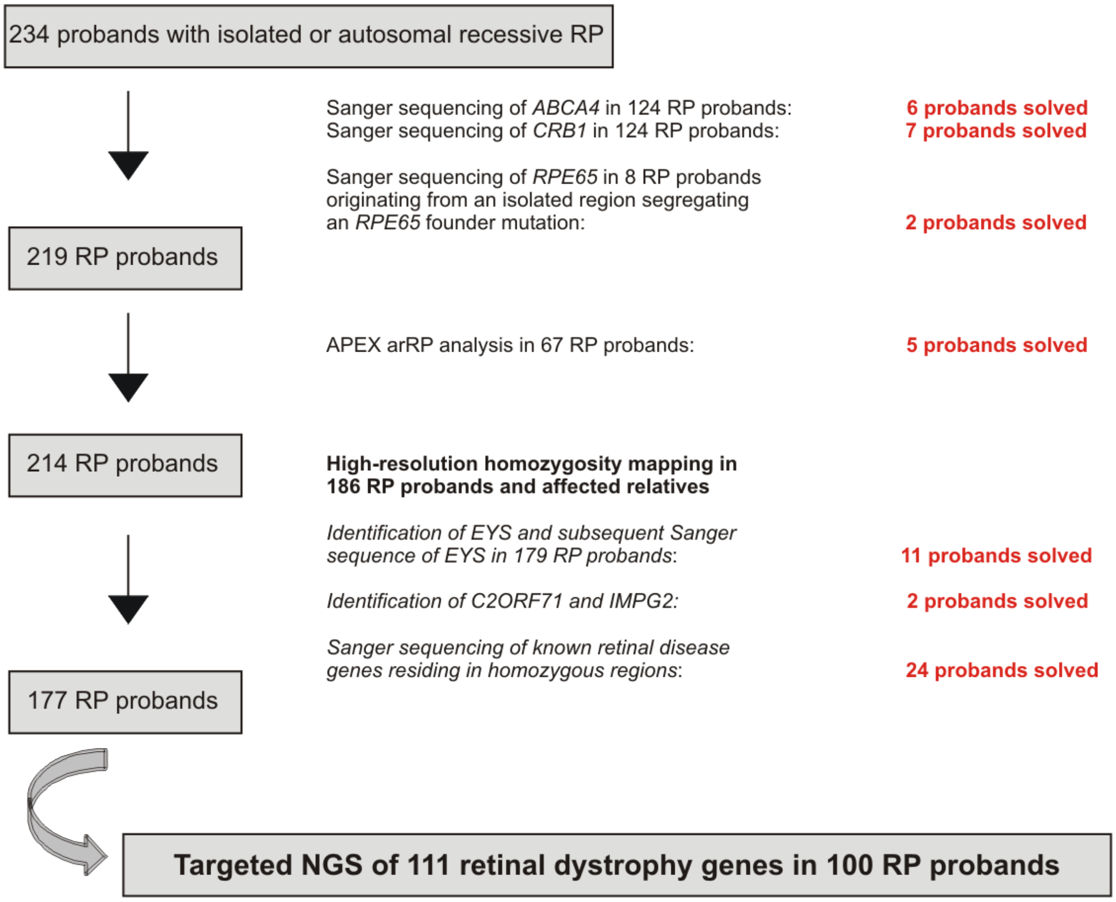

Supp. Figure S1. Overview of previous genetic analyses of our RP cohort
A total of 234 RP patient samples has been collected over the last 15 years. Several of these patients were screened for mutations in *ABCA4, CRB1*, and *RPE65* by Sanger sequencing, resulting in a molecular diagnosis in 20 patients. In 186 of the probands a genome-wide homozygosity mapping was performed, leading to the discovery of the disease-causing mutations in additional 37 patients. From the group of remaining RP probands, 100 were selected for the targeted NGS analysis of 111 retinal dystrophy genes described in this manuscript.

Supp. Figure S2

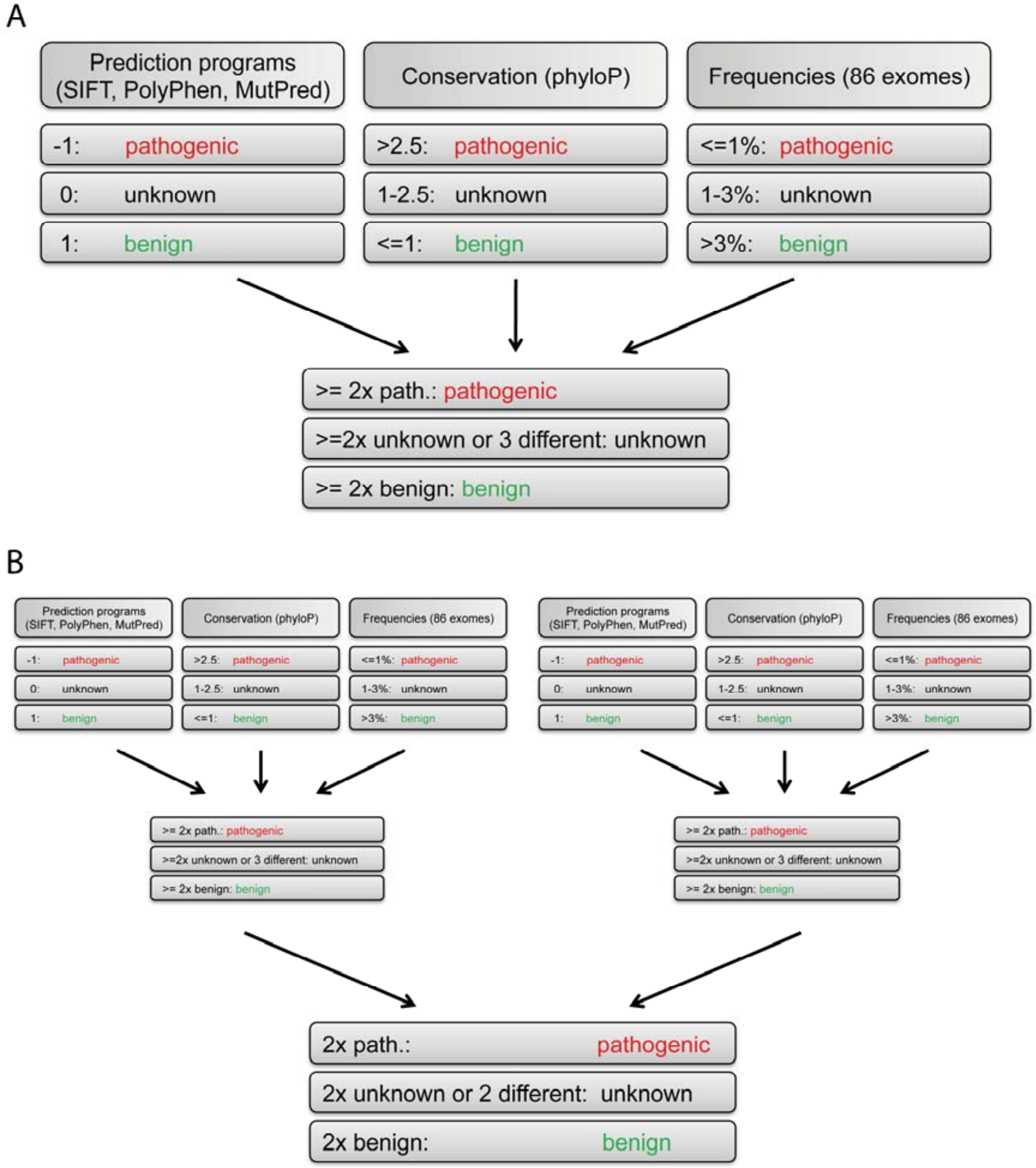

Supp. Figure S2. Systematic classification system for missense changes as well as for noncanonical splice site variants.
A systematic pipeline for the determination of pathogenicity was developed based on A) *in silico* evidence and B) combined evidence of two mutations in a recessive gene. **A)** With regard to *in silico* evidence three different features were considered important: (a) missense prediction software, (b) evolutionary conservation, and (c) population frequencies. In the case of prediction programs, the scores for three different tools were combined resulting in a single classification as “probably pathogenic”, “unknown” or “probably benign” using a majority vote. In case of three different predictions that variant was classified as “unknown” (see supplementary appendix for details). **B)** The final classification for recessive genes was established by accounting for combinations of variants.

Supp. Figure S3

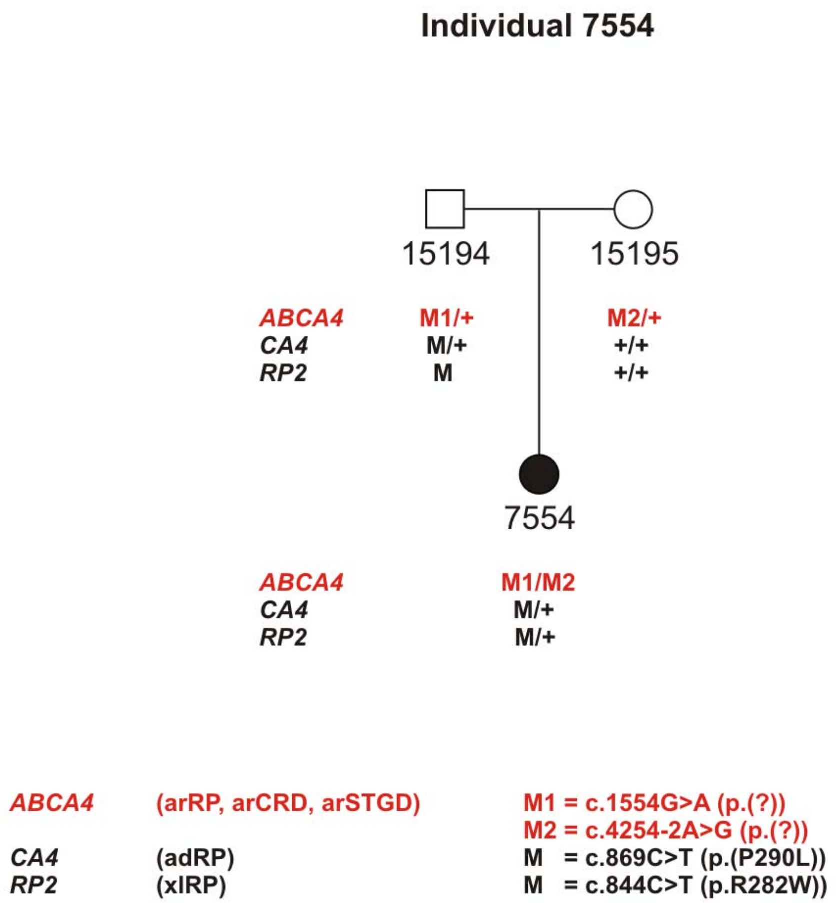

Individual 8322

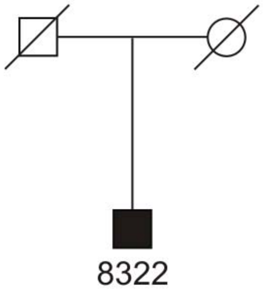

TOPORS **M/+**

TOPORS **(adRP)** **M = c.1730C>A (p.(S577Y))**

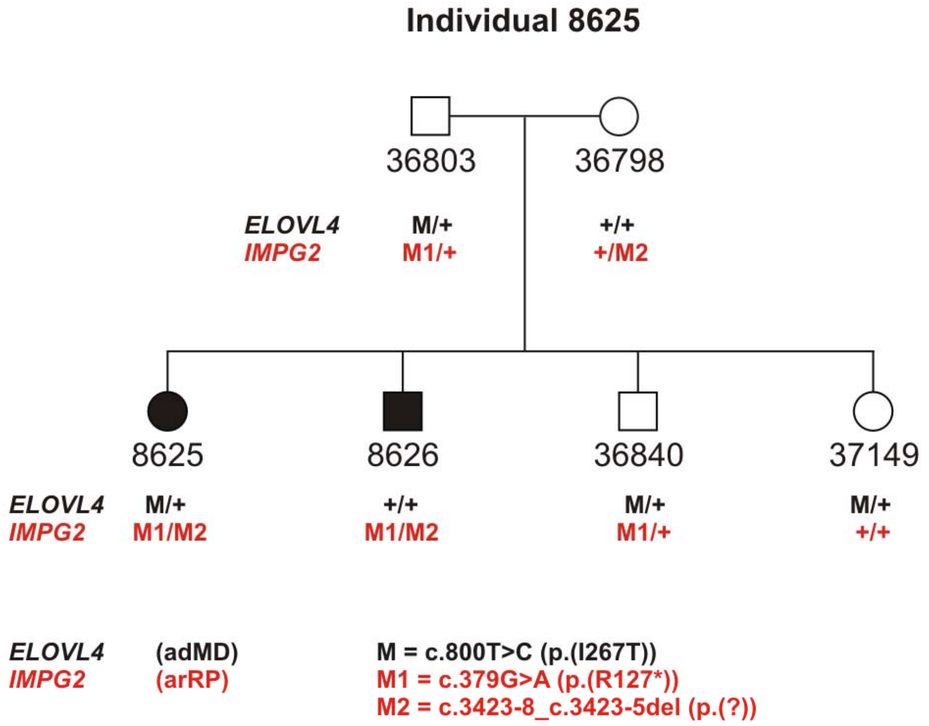
Individual 8625
36803
36798
ELOVL4
IMPG2
M/+
M1/+
+/+
+/M2
8625
8626
36840
37149
ELOVL4
IMPG2
M/+
M1/M2
+/+
M1/M2
M/+
M1/+
M/+
+/+
ELOVL4 (adMD)
IMPG2 (arRP)
M = c.800T>C (p.(I267T))
M1 = c.379G>A (p.(R127*))
M2 = c.3423-8_c.3423-5del (p.(?))

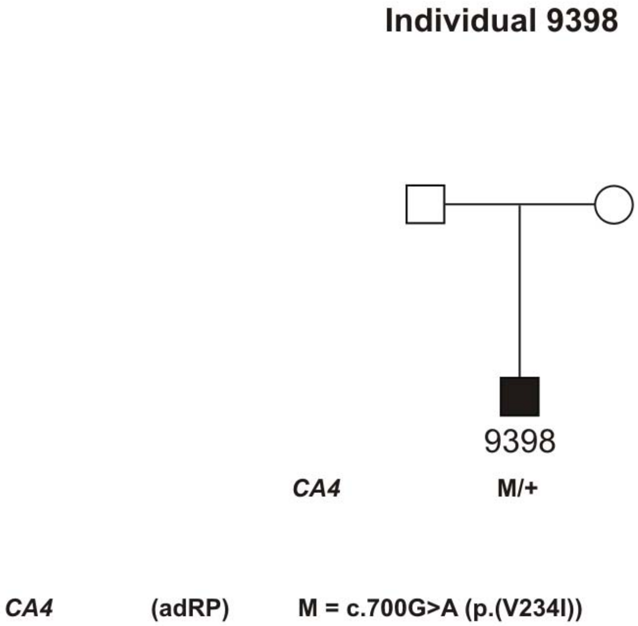
Individual 9398
9398
CA4
M/+
CA4
(adRP)
M = c.700G>A (p.(V234I))

Individual 9402

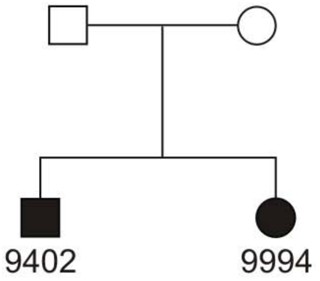

	9402	9994
CC2D2A	M1/M2	M1/M2
CRB1	M/+	+/+
RP2	M	+/+
SEMA4A	M/+	M/+
TOPORS	M/+	+/+
USH2A	M1/M2	+/+

CC2D2A	(arRP+ID)	M1 = c.685_687del (p.(E229del))<br>M2 = c.1810G>A (p.(G604S))
CRB1	(arRP, arLCA, adPPCA)	M = c.614T>C (p.(I205T))
RP2	(xlRP)	M = c.844C>T (p.(R282W))
SEMA4A	(adRP)	M = c.2138G>A (p.(R713Q))
TOPORS	(adRP)	M = c.58C>T (p.(P20S))
USH2A	(arRP, arUSH)	M1 = c.688G>A (p.(V230M))<br>M2 = c.9959G>A (p.(G3320D))

Individual 9437

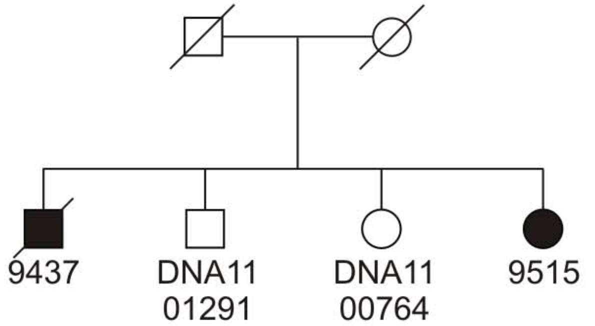

CRB1	M/+	M/+	+/+	M/+
TOPORS	M/+	+/+	M/+	M/+
USH2A	M1/M2	M1/+	+/+	M1/M2

CRB1	(arRP, arLCA, adPPCA)	M = c.14A>G (p.(N5S))
TOPORS	(adRP)	M = c.58C>T (p.(P20S))
USH2A	(arRP, arUSH)	M1 = c.486-14G>A (p.(?))
		M2 = c.12729G>A (p.(W4243*))

Individual 9444

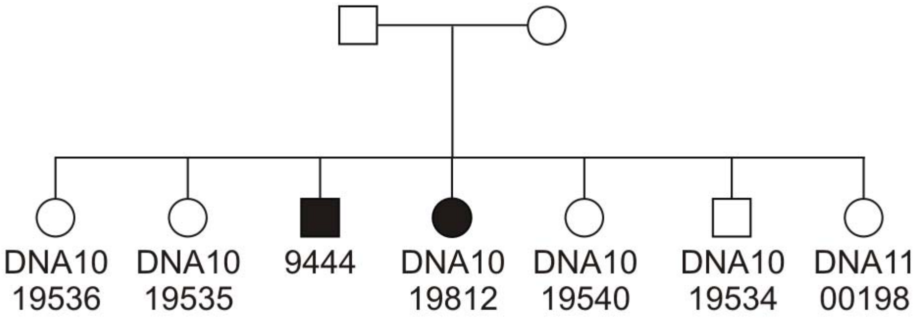

	DNA10 19536	DNA10 19535	9444	DNA10 19812	DNA10 19540	DNA10 19534	DNA11 00198
ABCA4	**M1/M2**	**M1/+**	**M1/M2**	**M1/M2**	**M1/+**	**M1/+**	**M1/+**
CRB1	**+/+**	**+/+**	**M/+**	**M/+**	**+/+**	**+/+**	**M/+**
SEMA4A	**M/+**	**+/+**	**M/+**	**M/+**	**M/+**	**M/+**	**+/+**
TOPORS	**M/+**	**M/+**	**M/+**	**+/+**	**M/+**	**M/+**	**M/+**

ABCA4	**(arRP, arCRD, arSTGD)**	**M1 = c.5843C>T (p.(P1948L))**<br>**M2 = c.6529G>A (p.(D2177N))**
CRB1	**(arRP, arLCA, adPPCA)**	**M = c.484G>A (p.(V162M))**
SEMA4A	**(adRP)**	**M = c.2044C>T (p.(P682S))**
TOPORS	**(adRP)**	**M = c.58C>T (p.(P20S))**

Individual 9470

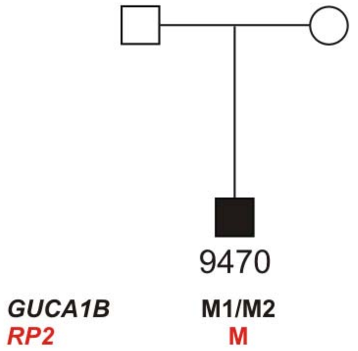

GUCA1B	(adRP)	M1 = c.465G>T (p.(E155D))
		M2 = c.475+8G>A (p.(?))
RP2	(xlRP)	M = c.323G>A (p.(C108Y))

Individual 9472

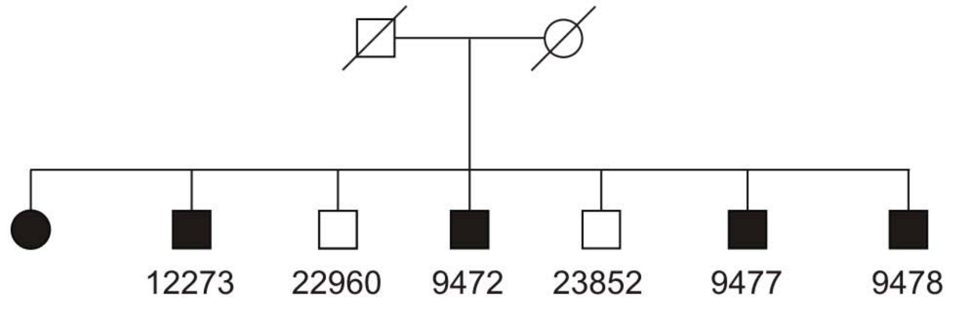

	12273	22960	9472	23852	9477	9478
PDE6B	M/M	+/+	M/M	M/+	M/M	M/M
PRPH2	+/+	+/+	M/+	+/+	+/+	+/+
RGR	M/+	+/+	M/+	+/+	+/+	+/+
SNRNP200	+/+	M/+	M/+	+/+	M/+	+/+

PDE6B	(arRP, adCSNB)	M = c.1920+2T>C (p.(?))
PRPH2	(adMD, adRP)	M = c.424C>T (p.(R142W))
RGR	(arRP, adCS)	M = c.756+5A>G (p.(?))
SNRNP200	(adRP)	M = c.6340A>G (p.(M2114V))

Individual 9493

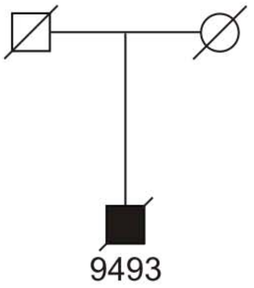

AHI1	**M/M**
EYS	**M1/M2**
PDE6B	**M1/M2**

AHI1	**(arJBTS)**	**M = c.2961+7_21delins20 (p.(?))**
EYS	**(arRP)**	**M1 = c.4891C>T (p.(P1631S))**
		**M2 = c.7796A>G (p.(H2599R))**
PDE6B	**(arRP, adCSNB)**	**M1 = c.1043_1044insCG (p.(A349fs))**
		**M2 = c.1927_1969delinsGG (p.(N643fs))**

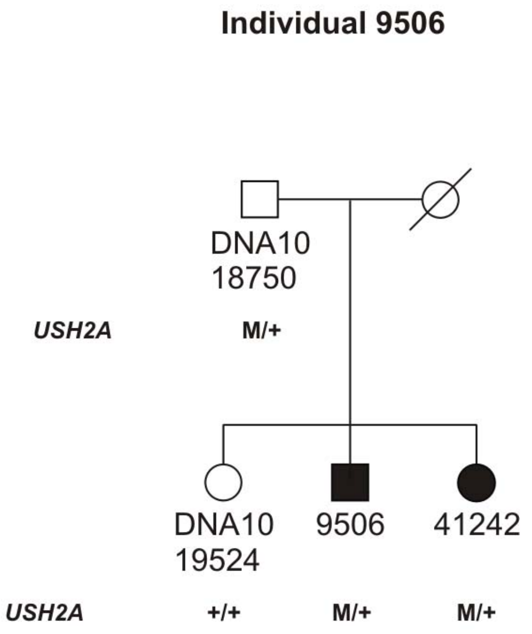

USH2A (arRP, arUSH) M = c.[4976G>A; 10561T>C] (p.[(R1659K);(W3521R)])

Individual 9509

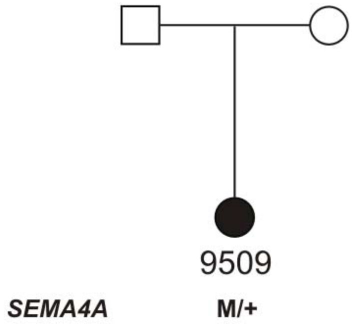

SEMA4A **(adRP)** **M = c.2138G>A (p.(R713Q))**

Individual 9511

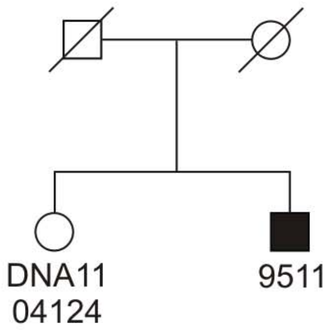

GUCY2D	+/+	M/+
RP1	+/+	M/+

GUCY2D	(arLCA, adCRD)	M = c.3225-7C>T (p.(?))
RP1	(adRP, arRP)	M = c.4735T>G (p.(L1579V))

Individual 9518

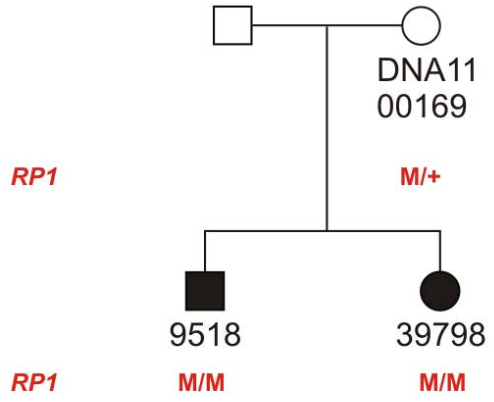

RP1 (adRP, arRP) M = c.515T>G (p.(L172R))

Individual 9520

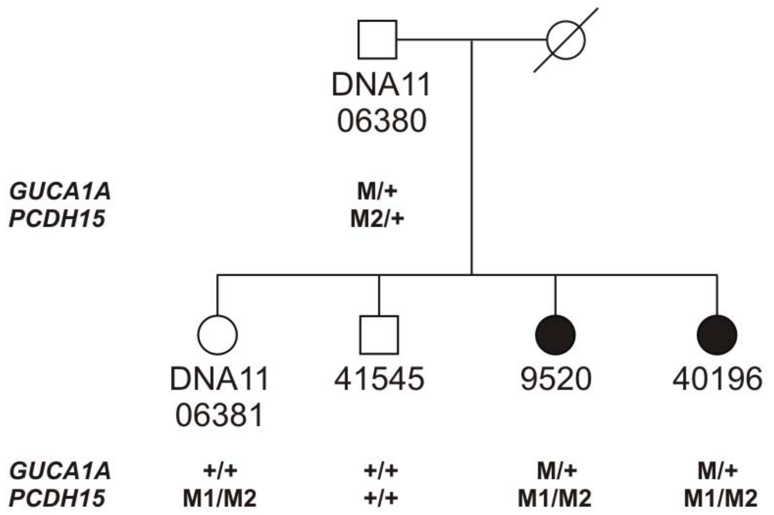

GUCA1A	(adCRD)	M = c.149C>T (p.(P50L))
PCDH15	(arUSH, arNSHI)	M1 = c.3389-4C>T (p.(?))
		M2 = c.4552insTTG (p.(1518insL))

Individual 9527

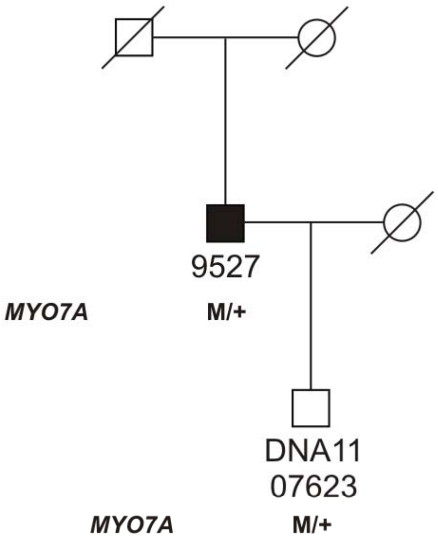

MYO7A **(arUSH, arNSHI)** **M = c.[5156A>G;5560G>A] (p.[(Y1719C;V1854M)])**

Individual 9528

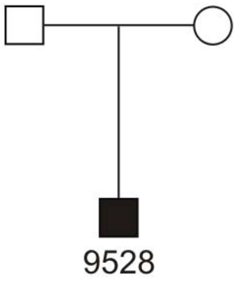

Individual 9537

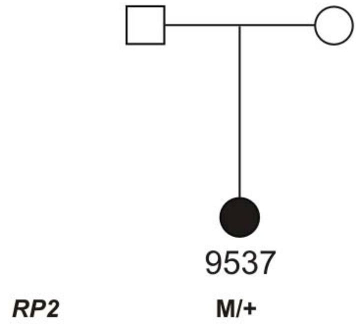

RP2 **(xlRP)** **M = c.844C>T (p.(R282W))**

Individual 9539

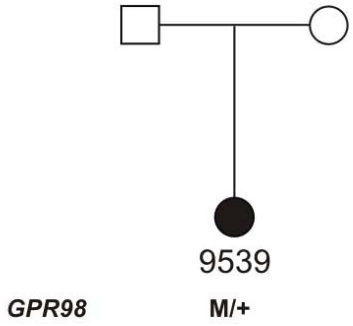

GPR98 **(arUSH)** **M = c.[8407G>A;12269C>A] (p.[(A2803T);(T4090N)])**

Individual 9543

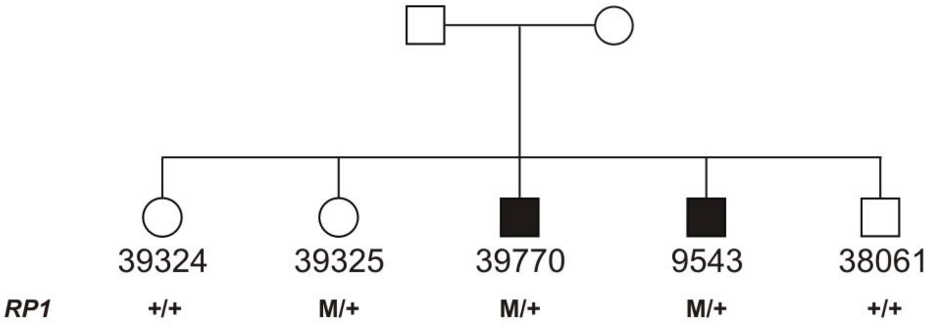

RP1 **(adRP, arRP)** **M = c.616-6T>C (p.(?))**

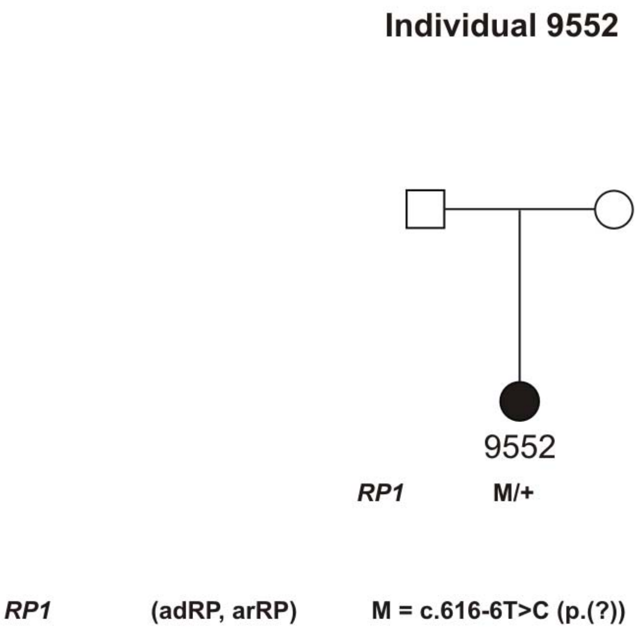
Individual 9552
9552
RP1
M/+
RP1
(adRP, arRP)
M = c.616-6T>C (p.(?))

Individual 11005

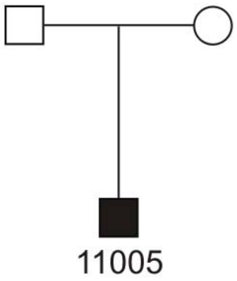

GUCY2D	**M/M**
RPGR	**M**

GUCY2D	**(arLCA, adCRD)**	**M = c.154G>T (p.(A52S))**
RPGR	**(xlRP)**	**M = c.[223A>G;1579_1581del] (p.[(I75V);(Q526del)])**

Individual 11319

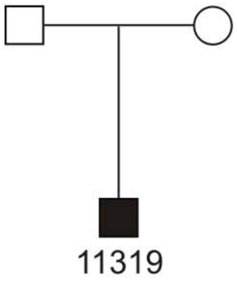

CACNA1F	M
PROM1	M/+
RP2	M
RPGRIP1L	M1/M2
SNRNP200	M/+

CACNA1F	(xlCSNB)	M = c.1556G>A (p.(R519Q))
PROM1	(arRP, adMD)	M = c.1034T>C (p.(V345A))
RP2	(xlRP)	M = c.844C>T (p.(R282W))
RPGRIP1L	(arJBTS, arMKS)	M1 = c.972C>G (p.(C324W))<br>M2 = c.2240G>A (p.(R747Q))
SNRNP200	(adRP)	M = c.4165-9G>A (p.(?))

Individual 11893

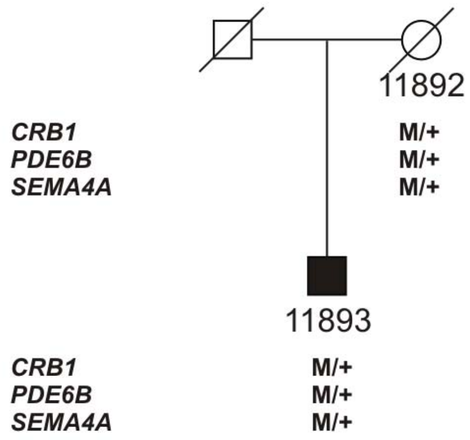

CRB1	**(arRP, arLCA, adPPCA)**	**M = c.2842+5G>A (p.(?))**
PDE6B	**(arRP, adCSNB)**	**M = c.496G>A (p.(E166K))**
SEMA4A	**(adRP)**	**M = c.2138G>A (p.(R713Q))**

Individual 11919

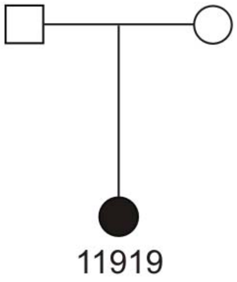

CACNA1F	**M/M**
TOPORS	**M/+**

CACNA1F	**(xlCSNB)**	**M = c.1556G>A (p.(R519Q))**
TOPORS	**(adRP)**	**M = c.58C>T (p.(P20S))**

Individual 13023

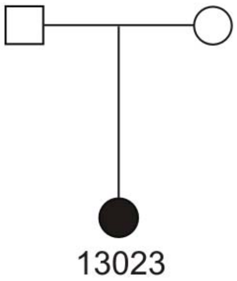

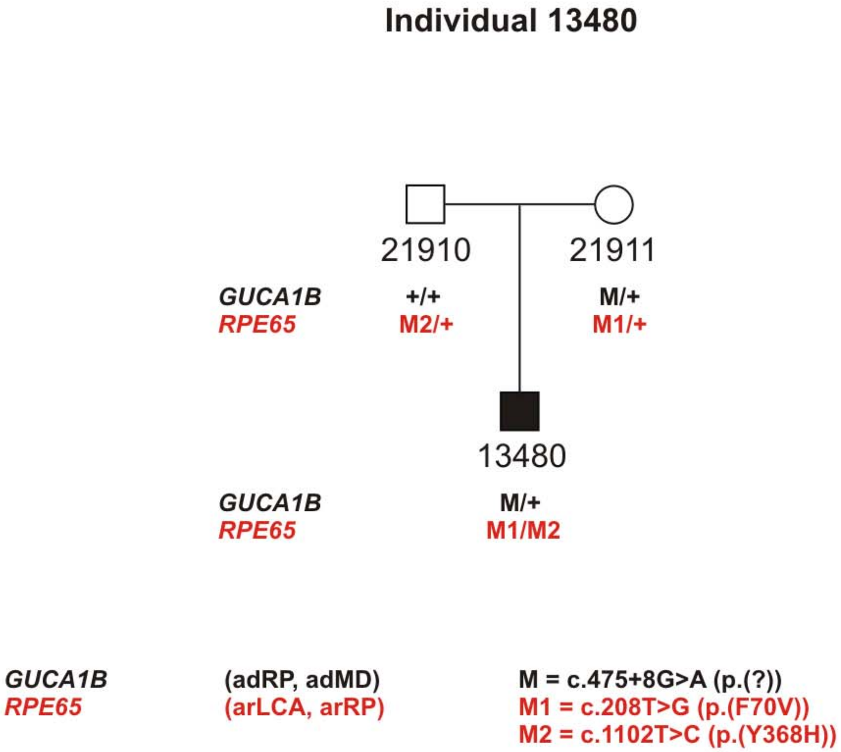
Individual 13480
21910
21911
GUCA1B
RPE65
+/+
M2/+
M/+
M1/+
13480
GUCA1B
RPE65
M/+
M1/M2
GUCA1B (adRP, adMD) M = c.475+8G>A (p.(?))
RPE65 (arLCA, arRP) M1 = c.208T>G (p.(F70V))
M2 = c.1102T>C (p.(Y368H))

Individual 14181

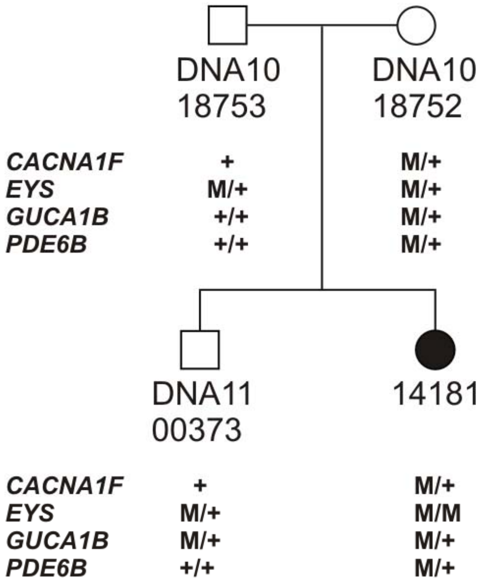

CACNA1F	**(xlCSNB)**	**M = c.1556G>A (p.(R519Q))**
EYS	**(arRP)**	**M = c.6045-4_6045-3del (p.(?))**
GUCA1B	**(adRP, adMD)**	**M = c.475+8G>A (p.(?))**
PDE6B	**(arRP, adCSNB)**	**M = c.1107+3A>G (p.(?))**

Individual 14182

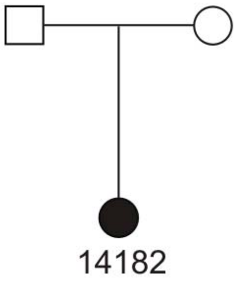

SEMA4A **M/+**

SEMA4A **(adRP)** **M = c.2138G>A (p.(R713Q))**

Individual 14192

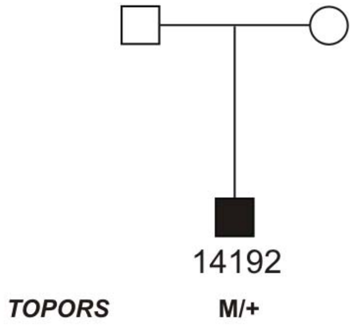

TOPORS (adRP) M = c.58C>T (p.(P20S))

Individual 14267

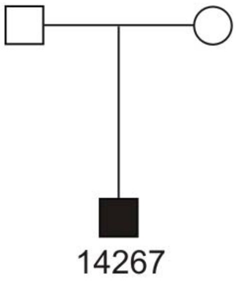

GUCA1B	**M/+**
PDE6B	**M/+**

GUCA1B	**(adRP, adMD)**	**M = c.475+8G>A (p.(?))**
PDE6B	**(arRP, adCSNB)**	**M = c.1060-13G>A (p.(?))**

Individual 14428

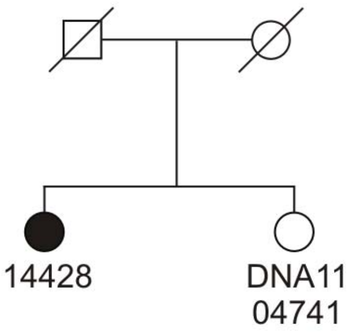

	14428	DNA11 04741
CACNA1F	**M/+**	**M/+**
GPR98	**M1/M2**	**M1/M2**

CACNA1F **(xlCSNB)** **M = c.811G>A (p.(G271R))**
GPR98 **(arUSH)** **M1 = c.7582C>T (p.(P2528S))**
M2 = c.[9607T>A;15440A>G] (p.[(S3203T);(D5147G)])

Individual 14705

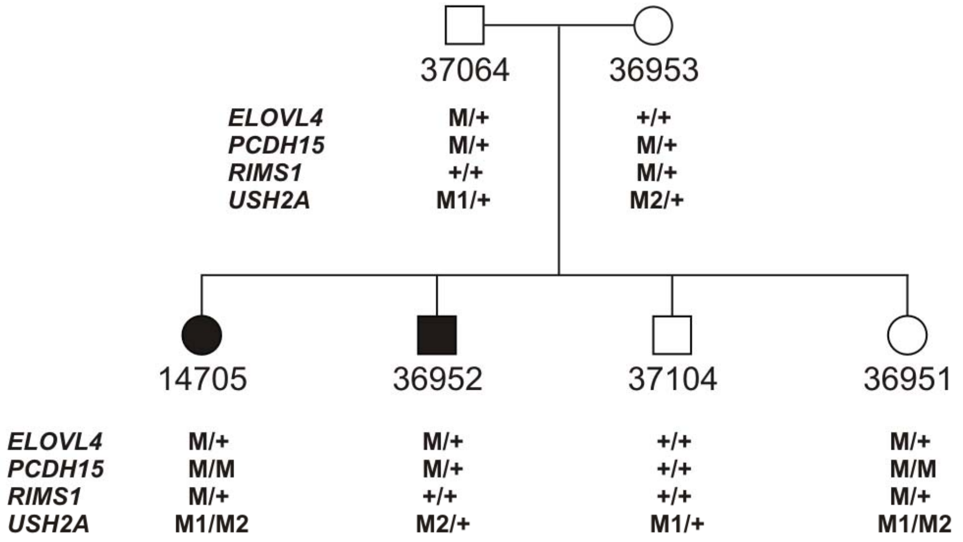

ELOVL4 **(adMD)** **M = c.800T>C (p.(I267T))**
PCDH15 **(arUSH, arNSHI)** **M = c.3733-19C>A (p.(?))**
RIMS1 **(adCRD)** **M = c.2699-8T>C (p.(?))**
USH2A **(arRP, arUSH)** **M1 = c.6257C>A (p.(T2086N))**
M2 = c.11815G>A (p.(E3939K))

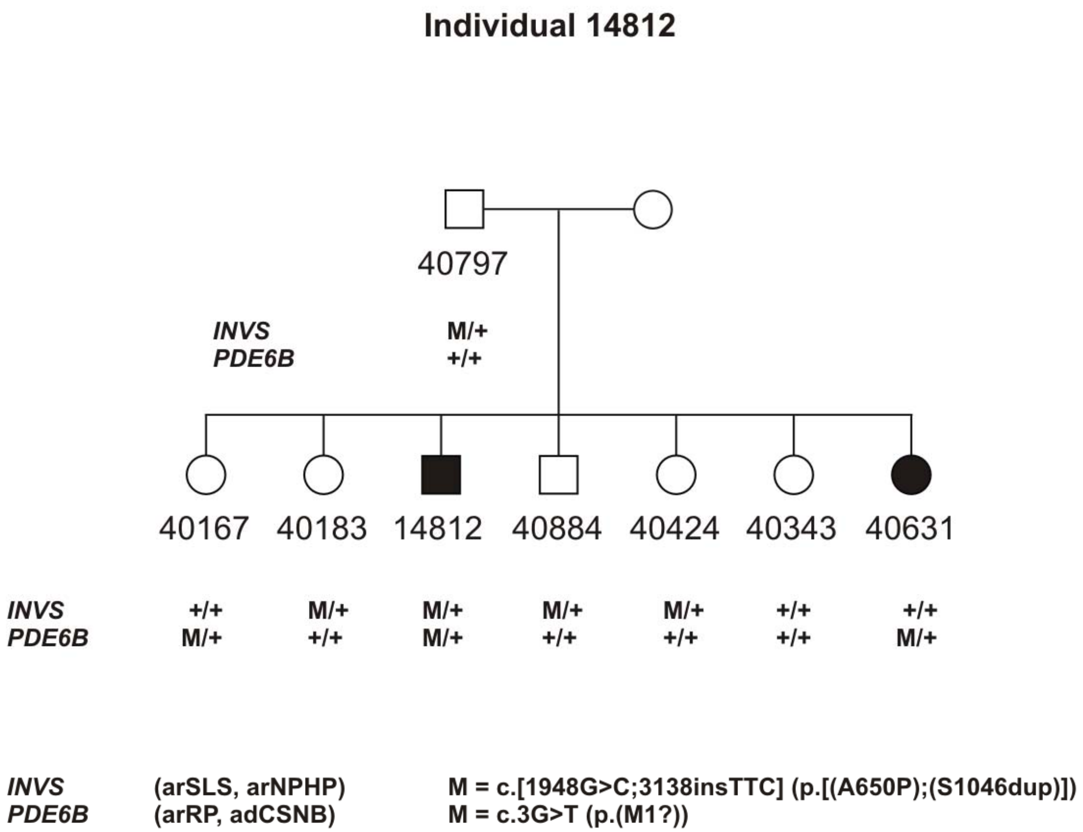
Individual 14812
40797
INVS M/+
PDE6B +/+
40167 40183 14812 40884 40424 40343 40631
INVS +/+ M/+ M/+ M/+ M/+ +/+ +/+
PDE6B M/+ +/+ M/+ +/+ +/+ +/+ M/+
INVS (arSLS, arNPHP) M = c.[1948G>C;3138insTTC] (p.[(A650P);(S1046dup)])
PDE6B (arRP, adCSNB) M = c.3G>T (p.(M1?))

Individual 15569

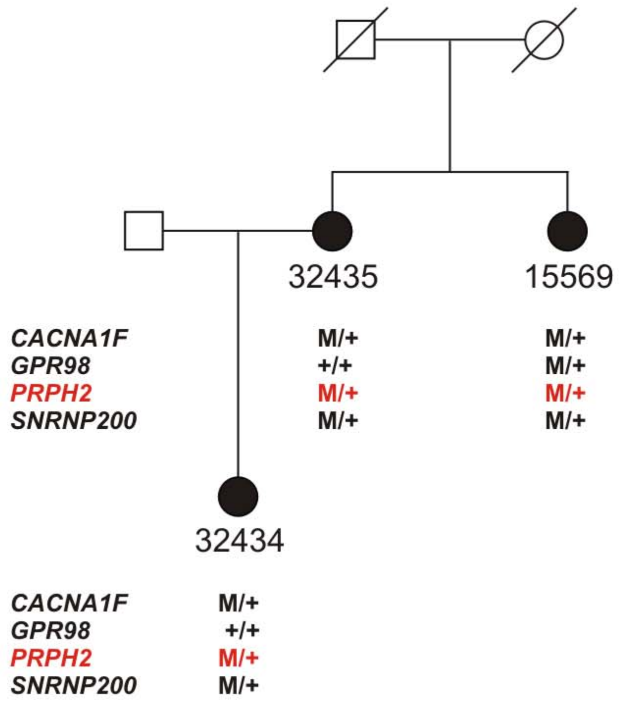

CACNA1F	**(xlCSNB)**	**M = c.1903G>A (p.(V635I))**
GPR98	**(arUSH)**	**M = c.[3151G>T;3191A>C] (p.[(D1051Y);(E1064A)])**
PRPH2	**(adRP, adCRD)**	**M = c.441del (p.(P147fs))**
SNRNP200	**(adRP)**	**M = c.1159A>G (p.(M387V))**

Individual 16389

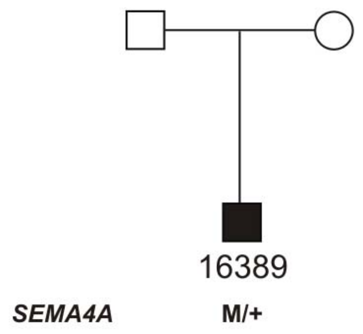

SEMA4A **(adRP)** **M = c.2044C>T (p.(P682S))**

Individual 16430

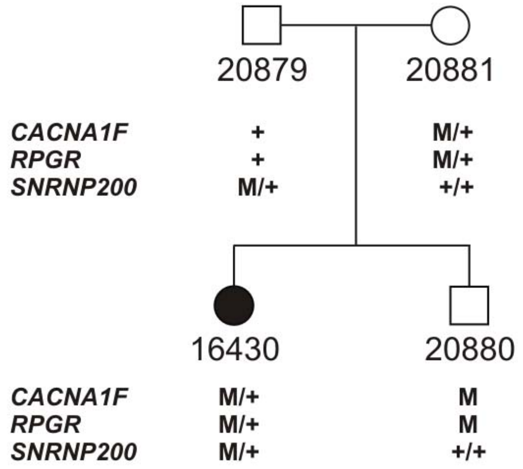

CACNA1F	**(xlCSNB)**	**M = c.1556G>A (p.(R519Q))**
RPGR	**(xlRP)**	**M = c.1579_1581del (p.(Q527del))**
SNRNP200	**(adRP)**	**M = c.5134-20T>C (p.(?))**

Individual 17593

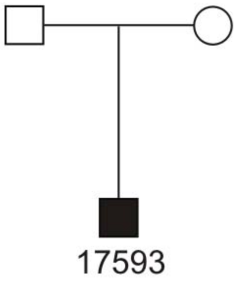

Individual 17597

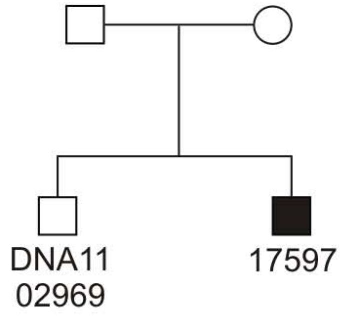

	DNA11 02969	17597
CC2D2A	M/+	M/M
CHM	M	M
SNRNP200	M/+	M/+
USH2A	+/+	M1/M2

CC2D2A	(arRP+ID)	M = c.685_687del (p.(E229del))
CHM	(xlCHM)	M = c.265A>T (p.(S89C))
SNRNP200	(adRP)	M = c.5134-20T>C (p.(?))
USH2A	(arRP, arUSH)	M1 = c.[12343C>T;13274C>T] (p.(R4115C);(T4425M)])<br>M2 = c.10525A>T (p.(K3509*))

Individual 17792

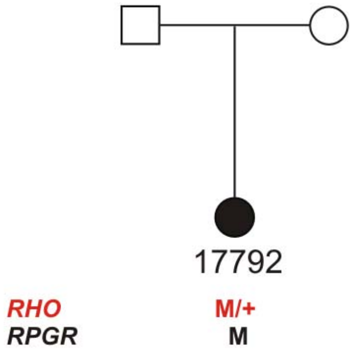

RHO	(arRP, adRP)	M = c.403C>T (p.(R135W)) (potentially *de novo*)
RPGR	(xlRP)	M = c.1579_1581del (p.(Q526del))

Individual 18060

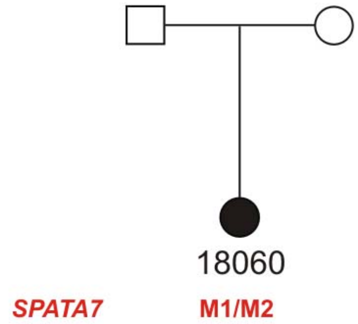

SPATA7 **(arLCA, arRP)** **M1 = c.3G>A (p.(M1?))**
M2 = c.322C>T (p.(R108*))

Individual 18130

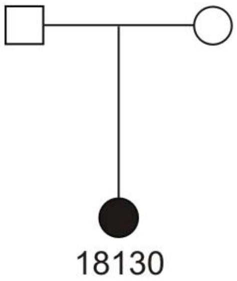

Individual 18336

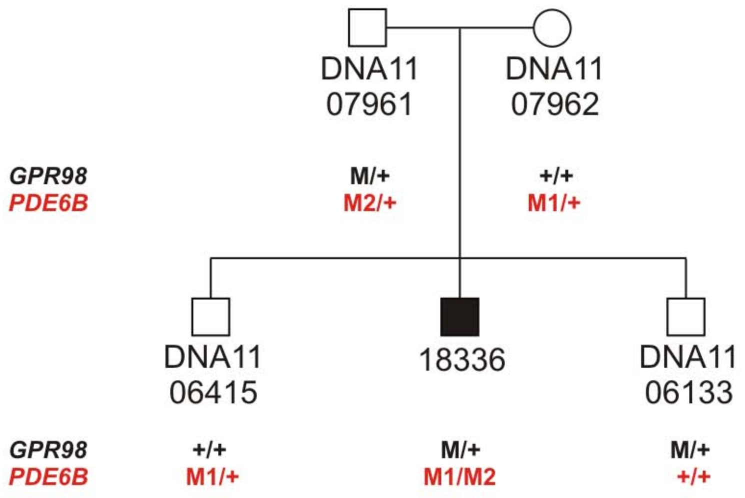

GPR98	(arUSH)	M = c.[8407G>A;12269C>A] (p.[(A2803T);(T4090N)])
PDE6B	(arRP, adCSNB)	M1 = c.1401+4_1401+16delins14;2326G>A] (p.[(?);(D776N)])
		M2 = c.1927_1969delinsGG (p.(N643fs))

Individual 18351

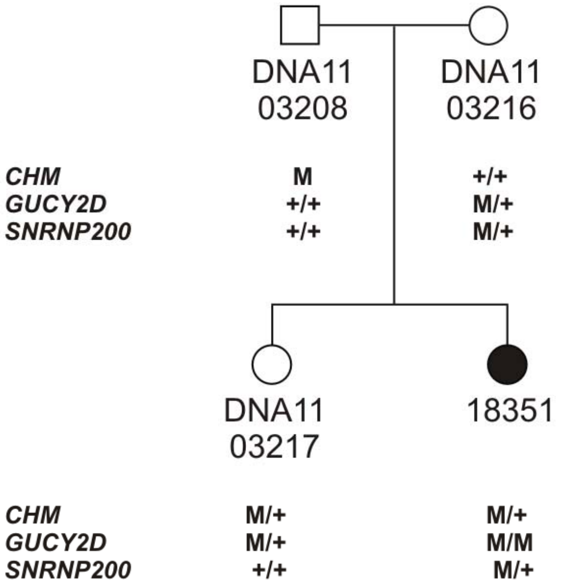

CHM	**(xlCHM)**	**M = c.265A>T (p.(S89C))**
GUCY2D	**(arLCA, adCRD)**	**M = c.154G>T (p.(A52S))**
SNRNP200	**(adRP)**	**M = c.3005A>G (p.(N1002S))**

Individual 18390

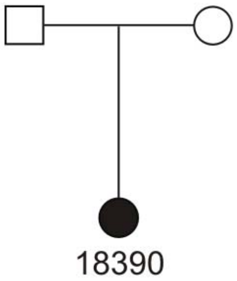

ABCA4	M/M
GUCA1B	M/+
RP2	M/+

ABCA4	(arRP, arCRD, arSTGD)	M = c.5843C>T (p.(P1948L))
GUCA1B	(adRP)	M = c.465G>T (p.(E155D))
RP2	(xlRP)	M = c.844C>T (p.(R282W))

Individual 18777

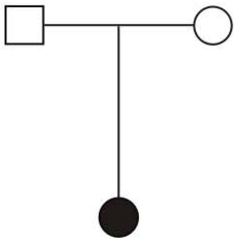

18777

C2ORF71	**M/M**
SEMA4A	**M/+**
SNRNP200	**M/M**

C2ORF71	**(arRP)**	**M = c.2600C>T (p.(P867L))**
SEMA4A	**(adRP)**	**M = c.2138G>A (p.(R713Q))**
SNRNP200	**(adRP)**	**M = c.4165G>A (p.(V1389I))**

Individual 19081

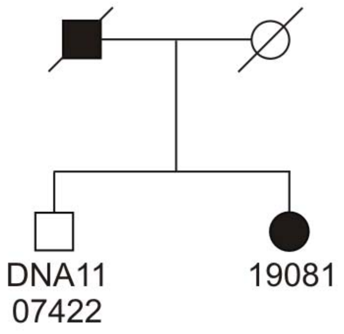

CA4	**+/+**	**M/+**
PDE6B	**M/+**	**M/+**
TOPORS	**+/+**	**M/+**

CA4	**(adRP)**	**M = c.700G>A (p.(V234I))**
PDE6B	**(arRP, adCSNB)**	**M = c.1060-13G>A (p.(?))**
TOPORS	**(adRP)**	**M = c.1730C>A (p.(S577Y))**

Individual 19531

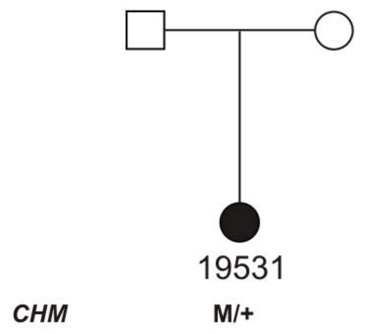

CHM **(xlCHM)** **M = c.265A>T (p.(S89C))**

Individual 19689

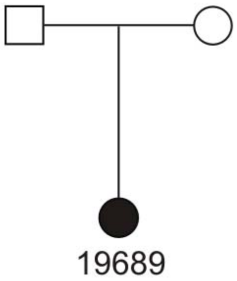

CA4	**M/+**
GUCA1B	**M/+**
RGR	**M/+**

CA4	**(adRP)**	**M = c.700G>A (p.(V234I))**
GUCA1B	**(adRP)**	**M = c.475+8G>A (p.(?))**
RGR	**(arRP, adCS)**	**M = c.756+5A>G (p.(?))**

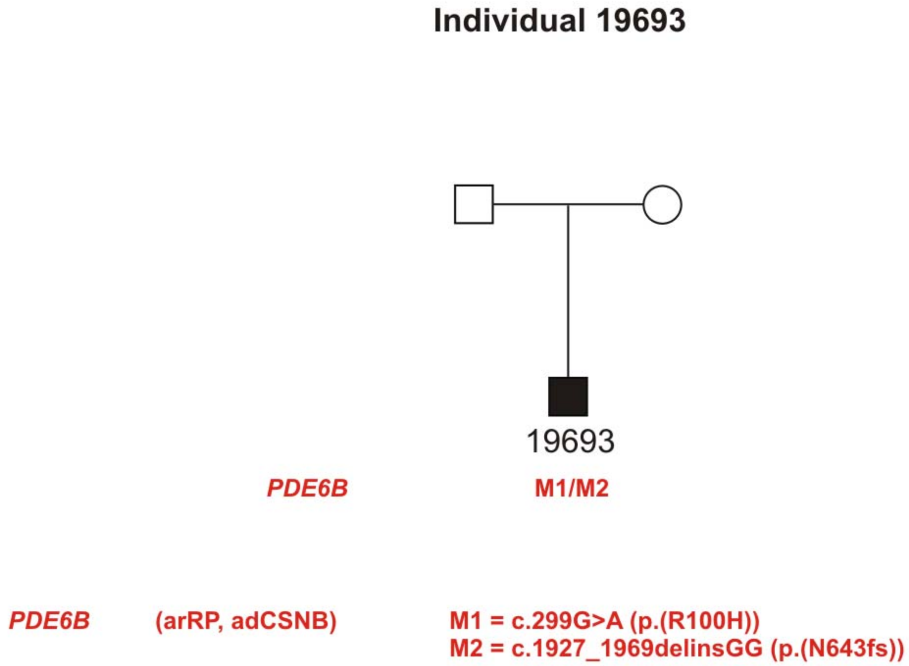
Individual 19693
19693
PDE6B
M1/M2
PDE6B
(arRP, adCSNB)
M1 = c.299G>A (p.(R100H))
M2 = c.1927_1969delinsGG (p.(N643fs))

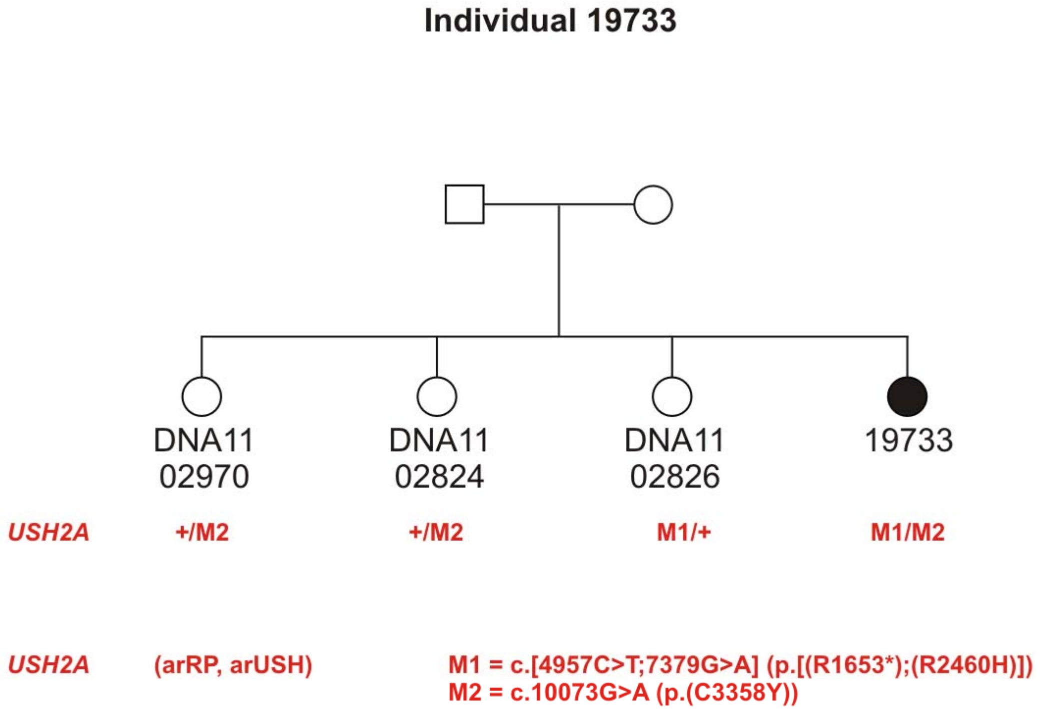

Individual 19733
DNA11 02970
DNA11 02824
DNA11 02826
19733
USH2A
+/M2
+/M2
M1/+
M1/M2
USH2A
(arRP, arUSH)
M1 = c.[4957C>T;7379G>A] (p.[(R1653*);(R2460H)])
M2 = c.10073G>A (p.(C3358Y))

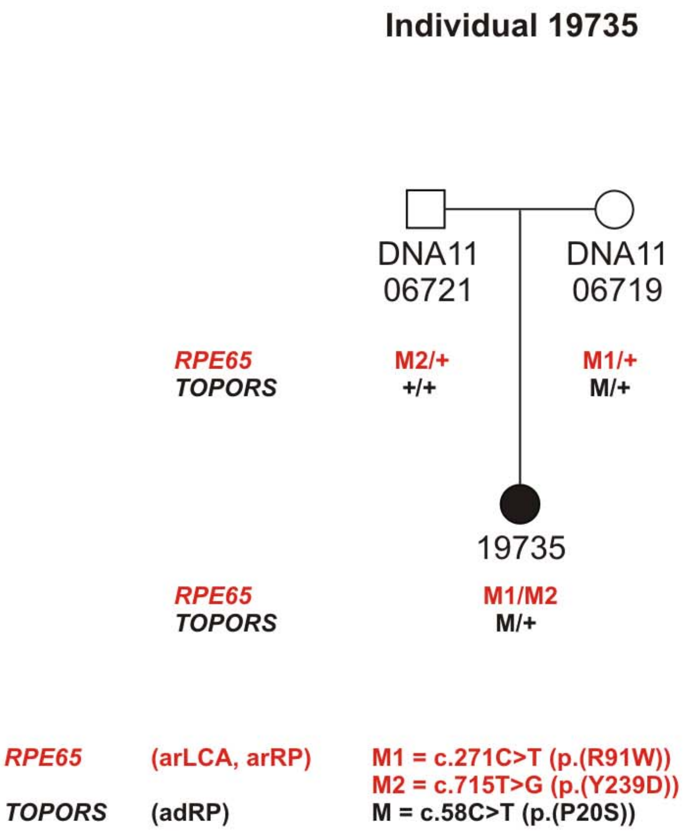
Individual 19735
DNA11
06721
DNA11
06719
RPE65
TOPORS
M2/+
+/+
M1/+
M/+
19735
RPE65
TOPORS
M1/M2
M/+
RPE65 (arLCA, arRP) M1 = c.271C>T (p.(R91W))
M2 = c.715T>G (p.(Y239D))
TOPORS (adRP) M = c.58C>T (p.(P20S))

Individual 20703

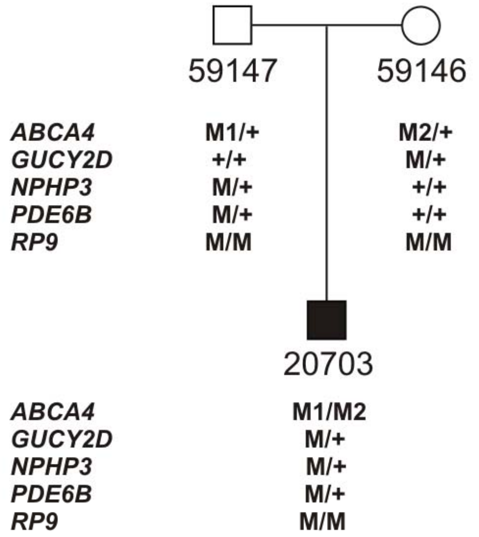

ABCA4	**(arRP, arCRD, arSTGD)**	**M1 = c.2828G>A (p.(R943Q))**
		**M2 = c.6764G>T (p.(S2255I))**
GUCY2D	**(arLCA, adCRD)**	**M = c.3225-7C>T (p.(?))**
NPHP3	**(arSLS, arNPHP)**	**M = c.[154G>A;944A>T] (p.[(A52T);(D315V)])**
PDE6B	**(arRP, adCSNB)**	**M = c.801C>A (p.(Y267*))**
RP9	**(adRP)**	**M = c.629A>G (p.(K210R))**

Individual 20984

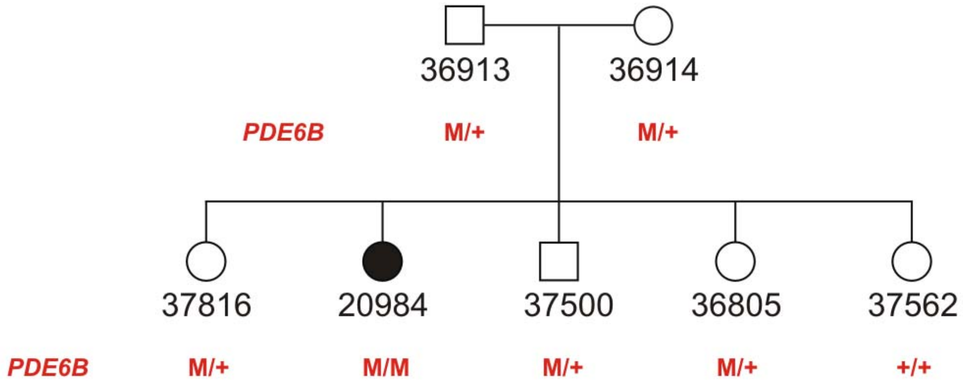

PDE6B **(arRP, adCSNB)** **M = c.1107+3A>G (p.(?))**

Individual 21010

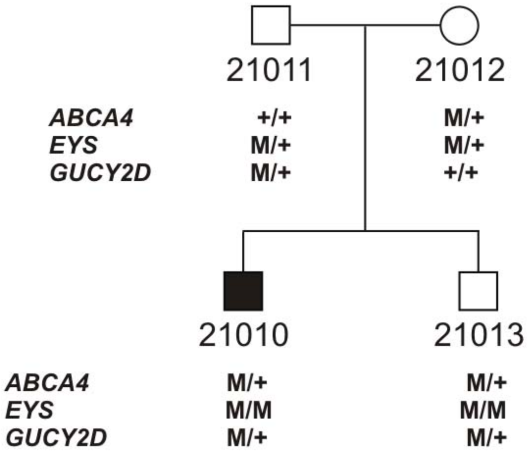

ABCA4	**(arRP, arCRD, arSTGD)**	**M = c.[1522C>T;5381C>A] (p.[(R508C);(A1794D)])**
EYS	**(arRP)**	**M = c.3444-5C>T (p.(?))**
GUCY2D	**(arLCA, adCRD)**	**M = c.3225-7C>T (p.(?))**

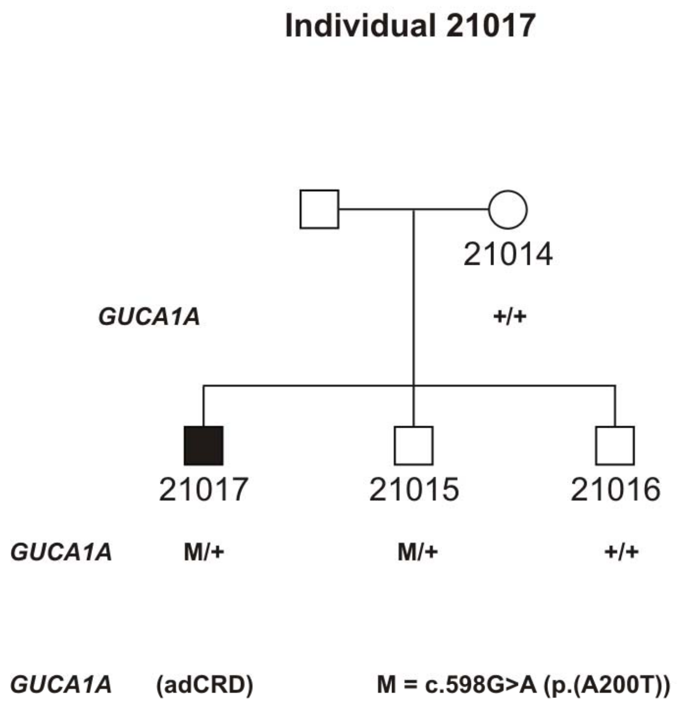
Individual 21017
21014
GUCA1A
+/+
21017
21015
21016
GUCA1A
M/+
M/+
+/+
GUCA1A
(adCRD)
M = c.598G>A (p.(A200T))

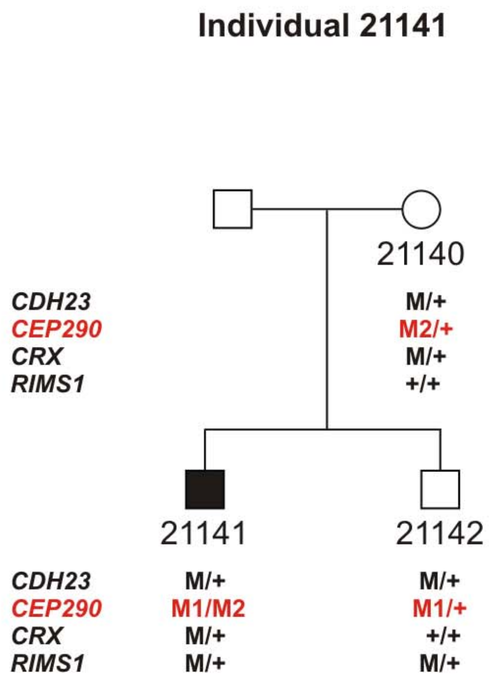

CDH23	(arUSH, arNSHI)	M = c.[3074G>A;4846-19G>C;c.5419G>A] (p.[(G1025D);(?);(V1807M)])
CEP290	(arLCA, arSLS, arJBTS)	M1 = c.3559del (p.(L1187fs)) M2 = c.4705-1G>T (p.(?))
CRX	(arLCA, adRP, adCRD)	M = c.472G>A (p.(A158T))
RIMS1	(adCRD)	M = c.5053A>G (p.(T1685A))

Individual 21213

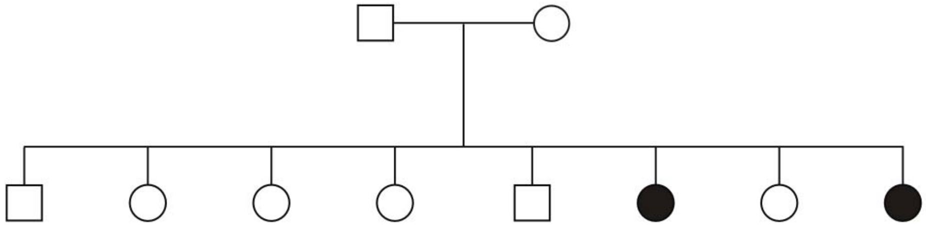

	33745	33466	33741	33602	33673	34457	33603	21213
CACNA1F	**+**	**M/+**	**M/+**	**M/+**	**+**	**M/+**	**M/+**	**M/+**
SEMA4A	**M/+**	**+/+**	**M/+**	**M/+**	**M/+**	**M/+**	**M/+**	**M/+**
USH2A	**M2/+**	**M1/+**	**+/+**	**M1/+**	**M1/M2**	**M1/M2**	**+/+**	**M1/M2**

CACNA1F	**(xlCSNB)**	**M = c.1546G>C (p.(R516G))**
SEMA4A	**(adRP)**	**M = c.2138G>A (p.(R713Q))**
USH2A	**(arRP, arUSH)**	**M1 = c.10448G>A (p.(G3483E))**
		**M2 = c.10561T>C (p.(W3521R))**

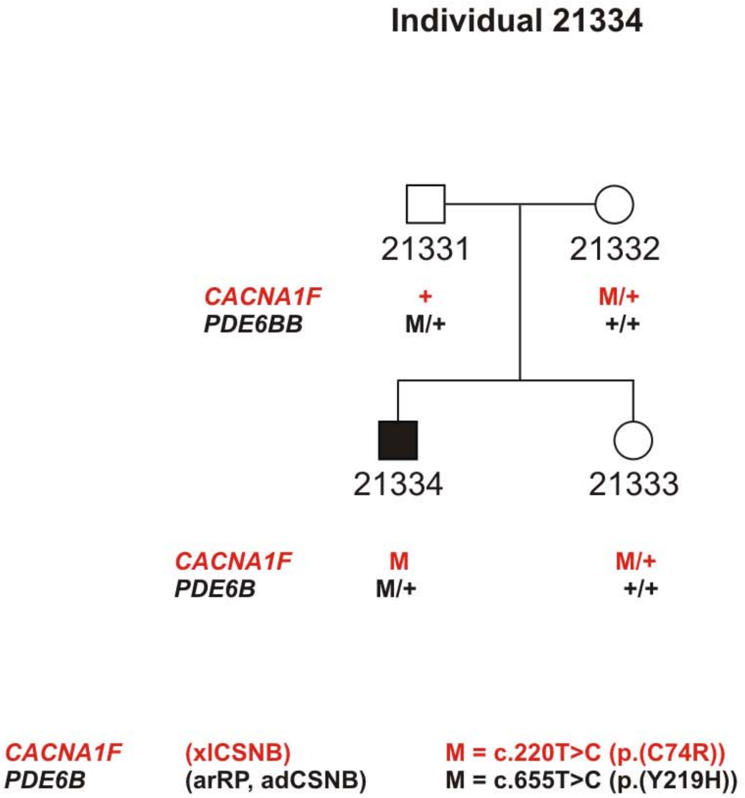
Individual 21334
21331
21332
CACNA1F
PDE6BB
+
M/+
M/+
+/+
21334
21333
CACNA1F
PDE6B
M
M/+
M/+
+/+
CACNA1F (xlCSNB) M = c.220T>C (p.(C74R))
PDE6B (arRP, adCSNB) M = c.655T>C (p.(Y219H))

Individual 21384

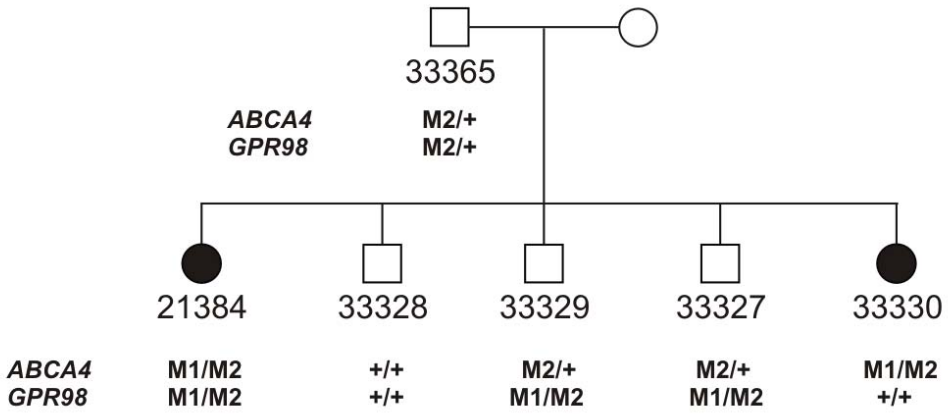

ABCA4	(arRP, arCRD, arSTGD)	M1 = c.3191-2_3191del (p.(?))
		M2 = c.5603A>T (p.(N1868I))
GPR98	(arUSH)	M1 = c.[3151G>T;3191A>C] (p.[(D1051Y);(E1064A)])
		M2 = c.9650C>T (p.(A3217V))

Individual 21642

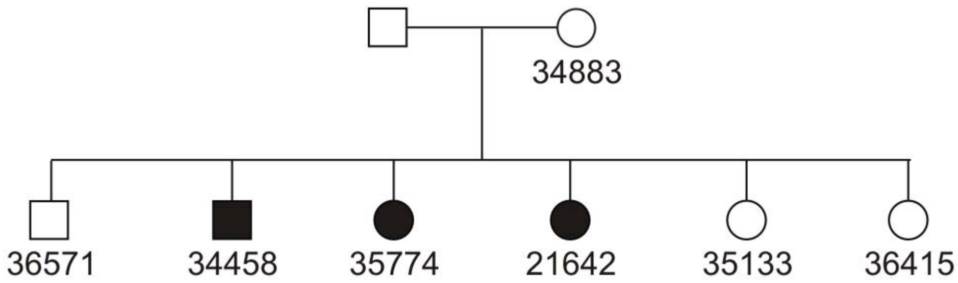

Individual 21933

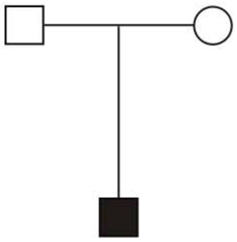

21933

GUCY2D	M/+
PDE6B	M1/M2
RP1	M1/M2
SEMA4A	M1/M2

GUCY2D	(arLCA, adCRD)	M = c.154G>T (p.(A52S))
PDE6B	(arRP, adCSNB)	M1 = c.655T>C (p.(Y219H))
		M2 = c.2503+5G>C (p.(?))
RP1	(arRP, adRP)	M1 = c.368_369dup (p.(P214fs))
		M2 = c.4241_4242del (p.(H1414fs))
SEMA4A	(adRP)	M1 = c.463-17C>A (p.(?))
		M2 = c.2138G>A (p.(R713Q))

Individual 22312

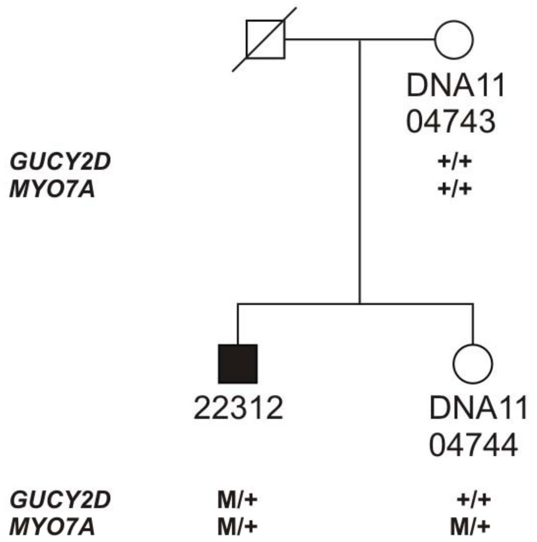

GUCY2D	**(arLCA, adCRD)**	**M = c.1724C>T (p.(P575L))**
MYO7A	**(arUSH, arNSHI)**	**M = c.[4589C>T;6640G>A] (p.[(S1530L);(G2214S)])**

Individual 22315

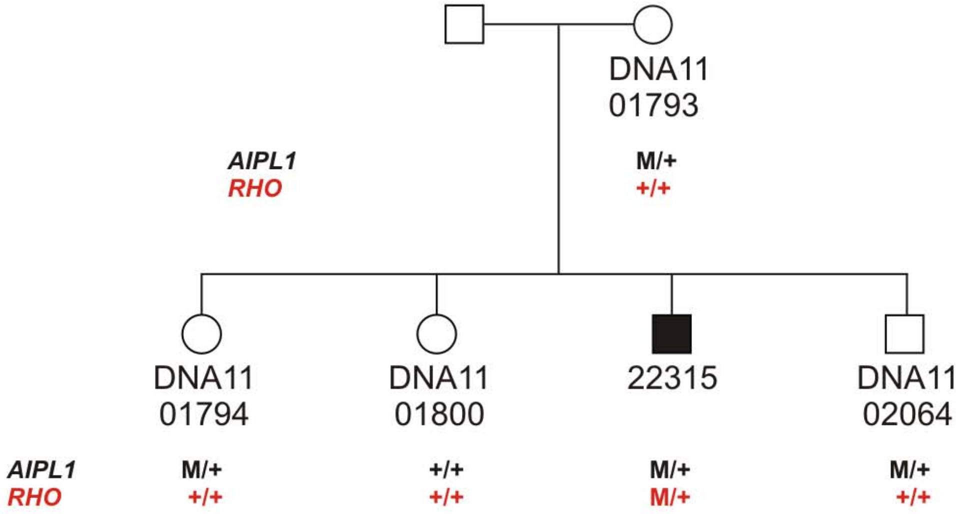

AIPL1 **(arLCA, adCRD)** **M = c.937C>A (p.(A313S))**
RHO **(arRP, adRP)** **M = c.538C>T (p.(P180S)) (*de novo*)**

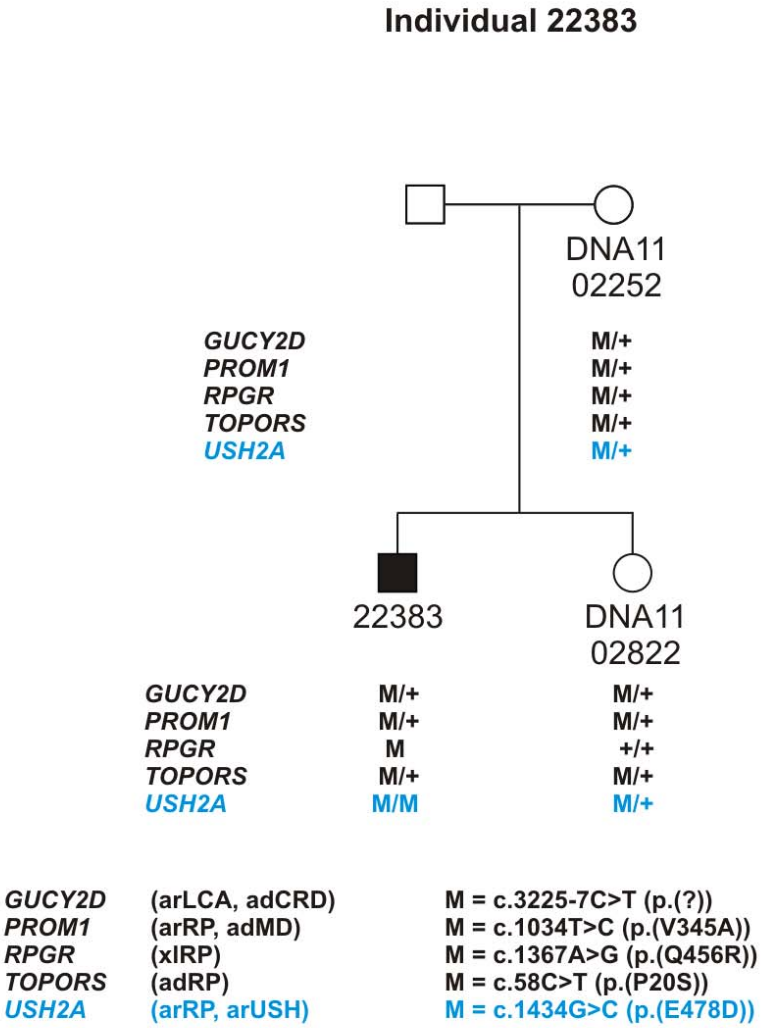
Individual 22383
DNA11
02252
GUCY2D M/+
PROM1 M/+
RPGR M/+
TOPORS M/+
USH2A M/+
22383
DNA11
02822
GUCY2D M/+ M/+
PROM1 M/+ M/+
RPGR M +/+
TOPORS M/+ M/+
USH2A M/M M/+
GUCY2D (arLCA, adCRD) M = c.3225-7C>T (p.(?))
PROM1 (arRP, adMD) M = c.1034T>C (p.(V345A))
RPGR (xlRP) M = c.1367A>G (p.(Q456R))
TOPORS (adRP) M = c.58C>T (p.(P20S))
USH2A (arRP, arUSH) M = c.1434G>C (p.(E478D))

Individual 22393

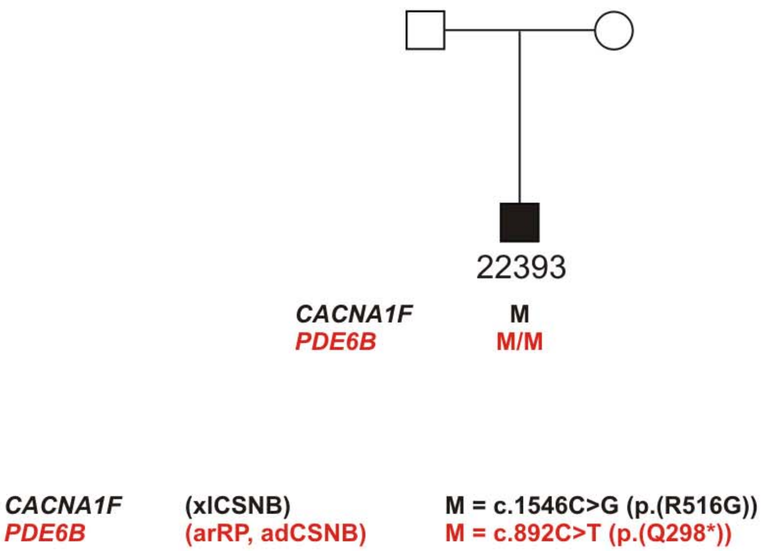

Individual 22731

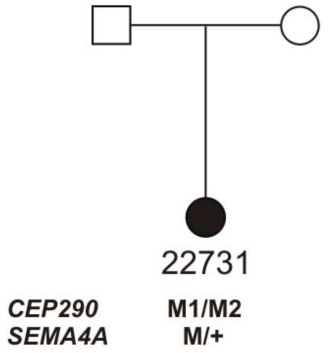

CEP290	**(arLCA, arSLS, arJBTS)**	**M1 = c.1825-14_1825-13del (p.(?))**
		**M2 = c.1991A>G (p.(D664G))**
SEMA4A	**(adRP)**	**M = c.2138G>A (p.(R713Q))**

Individual 22777

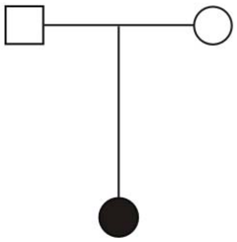

USH2A **M1/M2**

USH2A **(arRP, arUSH)** **M1 = c.1256G>T (p.(C419F))**
M2 = c.[12343C>T;13274C>T] (p.[(R4115C);(T4425M)])

Individual 23224

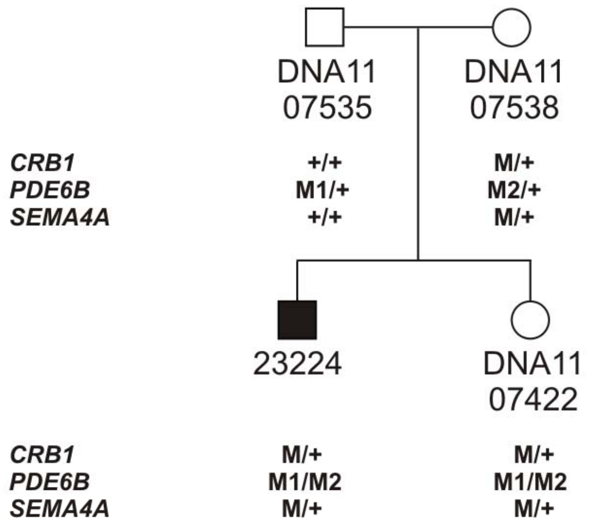

CRB1	**(arRP, arLCA, adPPCA)**	**M = c.135C>G (p.(C45W))**
PDE6B	**(arRP, adCSNB)**	**M1 = c.655T>C (p.(Y219H))**
		**M2 = c.[1401+4C>T;2326G>A] (p.([(?);(D776N)])**
SEMA4A	**(adRP)**	**M = c.2044C>T (p.(P682S))**

Individual 23422

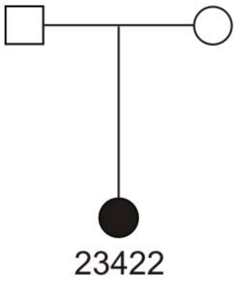

Individual 27528

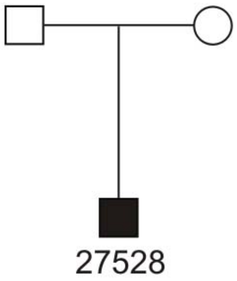

CRB1	M/+
GUCA1B	M/+
SNRNP200	M/+

CRB1	(arRP, arLCA, adPPCA)	M = c.71-12A>T (p.(?))
GUCA1B	(adRP, adMD)	M = c.476-9C>G (p.(?))
SNRNP200	(adRP)	M = c.575-7G>T (p.(?))

Individual 27585

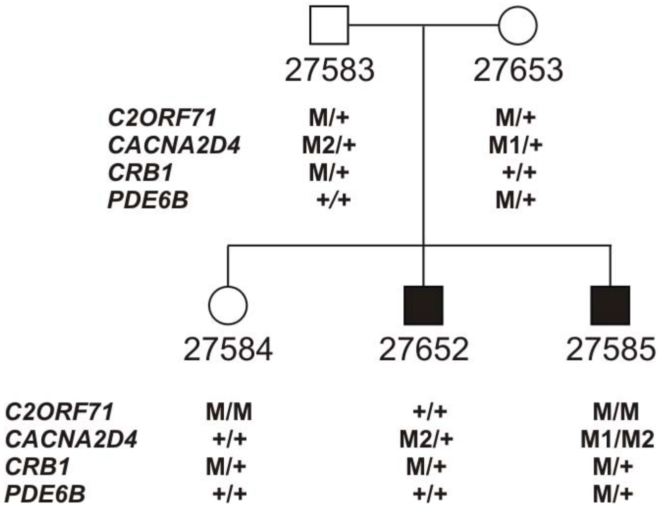

C2ORF71	**(arRP)**	**M = c.3671_3673dup (p.(S1225dup))**
CACNA2D4	**(arCD)**	**M1 = c.782-19C>T (p.(?))**
		**M2 = c.2793-15G>A (p.(?))**
CRB1	**(arRP, arLCA, adPPCA)**	**M = c.2843G>A (p.(C948Y))**
PDE6B	**(arRP, adCSNB)**	**M = c.655T>C (p.(Y219H))**

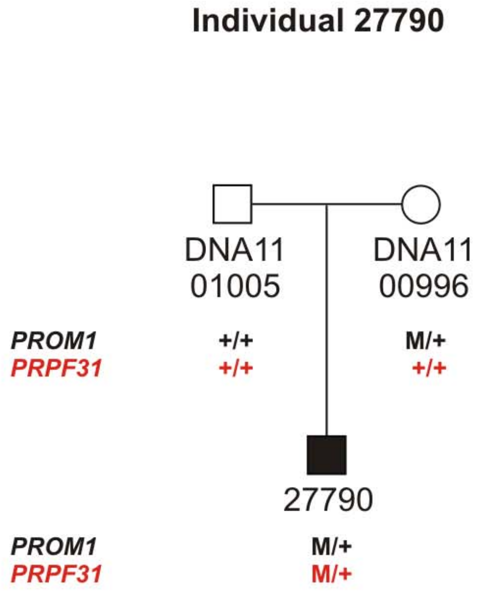

PROM1	(arRP, adMD)	M = c.524A>C (p.(Y175S))
PRPF31	(adRP)	M = c.553G>T (p.(E185*)) (*de novo*)

Individual 28064

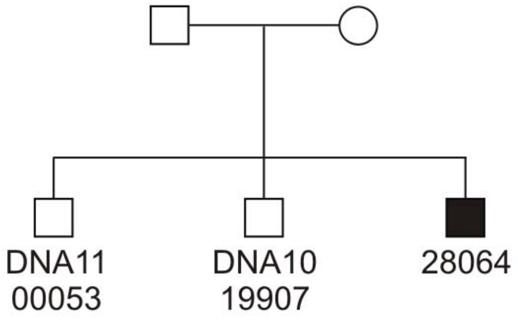

	DNA11 00053	DNA10 19907	28064
CHM	**M**	**+**	**M**
RPGR	**+**	**M**	**M**
SEMA4A	**M/+**	**+/+**	**M/+**

CHM	**(xlCHM)**	**M = c.265A>T (p.(S89C))**
RPGR	**(xlRP, xlCRD)**	**M = c.1793_1794insT (p.(N599fs))**
SEMA4A	**(adRP)**	**M = c.2138G>A (p.(R713Q))**

Individual 28557

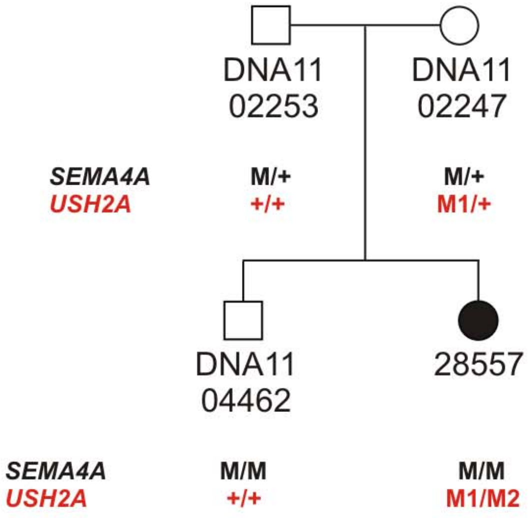

SEMA4A	**(adRP)**	**M = c.2138G>A (p.(R713Q))**
USH2A	**(arRP, arUSHII)**	**M1 = c.2276G>T (p.(C759F))**
		**M2 = c.5576T>G (p.(F1859C)) (*de novo*)**

Individual 28692

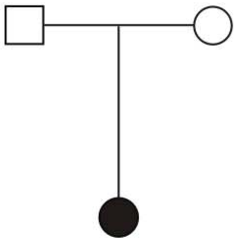

28692

GPR98	**M1/M2**
GUCY2D	**M/+**
USH2A	**M1/M2**

GPR98	**(arUSH)**	**M1 = c.4864T>C (p.(Y1622H))**
		**M2 = c.6229G>A (p.(E2077K))**
GUCY2D	**(arLCA, adCRD)**	**M = c.3225-7C>T (p.(?))**
USH2A	**(arRP, arUSH)**	**M1 = c.1434G>C (p.(E478D))**
		**M2 = c.14543G>A (p.(R4848Q))**

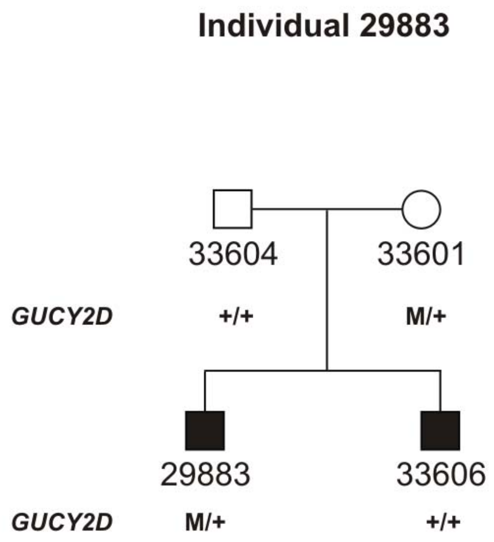

GUCY2D (arLCA, adCRD) M = c.2165G>A (p.(R722Q))

Individual 30364

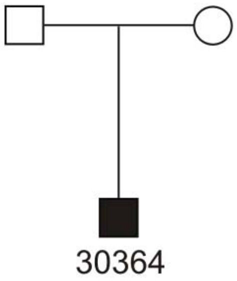

Individual 31035

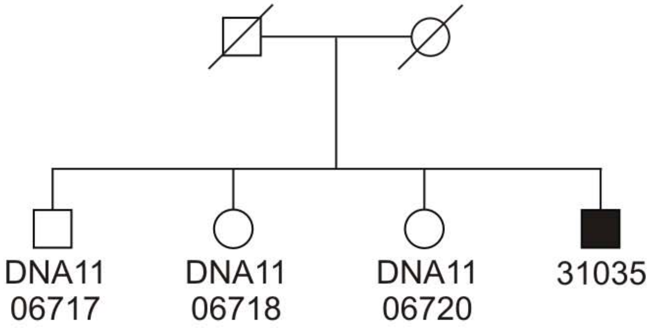

	DNA11 06717	DNA11 06718	DNA11 06720	31035
ABCA4	**M1/M2**	**M1/M2**	**M1/+**	**M1/M2**
AIPL1	**+/+**	**M/+**	**M/+**	**M/+**
GUCY2D	**M/+**	**M/+**	**M/+**	**M/+**
NR2E3	**+/+**	**+/+**	**+/+**	**M/+**

ABCA4	**(arRP, arCRD, arSTGD)**	**M1 = c.2828G>A (p.(R943Q))**
		**M2 = c.6764G>T (p.(S2255I))**
AIPL1	**(arLCA, adCRD)**	**M = c.932G>C (p.(R311L))**
GUCY2D	**(arLCA, adCRD)**	**M = c.61T>C (p.(W21R))**
NR2E3	**(adRP, arESCS)**	**M = c.95G>A (p.(W32*)) (potentially *de novo*)**

Individual 31124

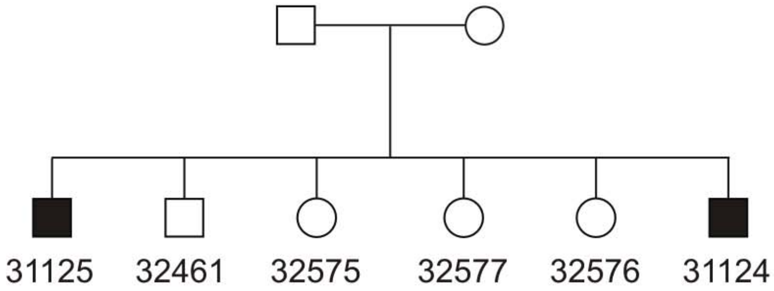

	31125	32461	32575	32577	32576	31124
ABCA4	**M1/M2**	**+/+**	**M1/M2**	**+/+**	**+/+**	**M1/M2**
SEMA4A	**M/+**	**+/+**	**M/+**	**+/+**	**M/+**	**M/+**
USH2A	**M1/M2**	**M1/+**	**M2/+**	**+/+**	**+/+**	**M1/M2**

ABCA4	**(arRP, arCRD, arSTGD)**	**M1 = c.635G>A (p.(R212H))**<br>**M2 = c.2701A>G (p.(T901A))**
SEMA4A	**(adRP)**	**M = c.2138G>A (p.(R713Q))**
USH2A	**(arRP, arUSHII)**	**M1 = c.486-14G>A (p.(?))**<br>**M2 = c.2276G>T (p.(C759F))**

Individual 31343

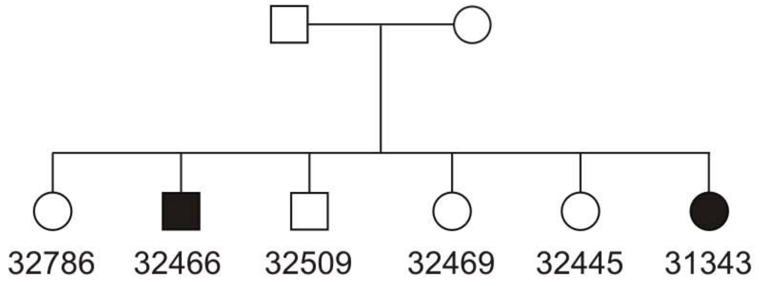

	32786	32466	32509	32469	32445	31343
GUCA1B	**M/+**	**+/+**	**M/+**	**M/+**	**M/+**	**M/+**
GUCY2D	**M/+**	**+/+**	**M/+**	**M/+**	**M/+**	**M/M**
NYX	**M/+**	**+**	**+**	**M/+**	**M/+**	**M/+**
USH2A	**+/+**	**M1/M2**	**M1/+**	**+/+**	**M1/+**	**M1/M2**

GUCA1B	**(adRP)**	**M = c.465G>T (p.(E155D))**
GUCY2D	**(arLCA, adCRD)**	**M = c.154G>T (p.(A52S))**
NYX	**(xlCSNB)**	**M = c.1394G>T (p.(C465F))**
USH2A	**(arRP, arUSH)**	**M1 = c.917_918insGCTG (p.(S307fs))**<br>**M2 = c.11007C>A (p.(S3669R))**

Individual 31494

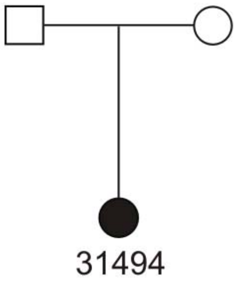

CHM	**M/+**
CRX	**M/+**
RPGR	**M/+**

CHM	**(xlCHM)**	**M = c.265A>T (p.(S89C))**
CRX	**(arLCA, adRP, adCRD)**	**M = c.196G>A (p.(V66I))**
RPGR	**(xlRP, xlCRD)**	**M = c.[1367A>G;1579_1581del] (p.[(Q456R);(Q527del)])**

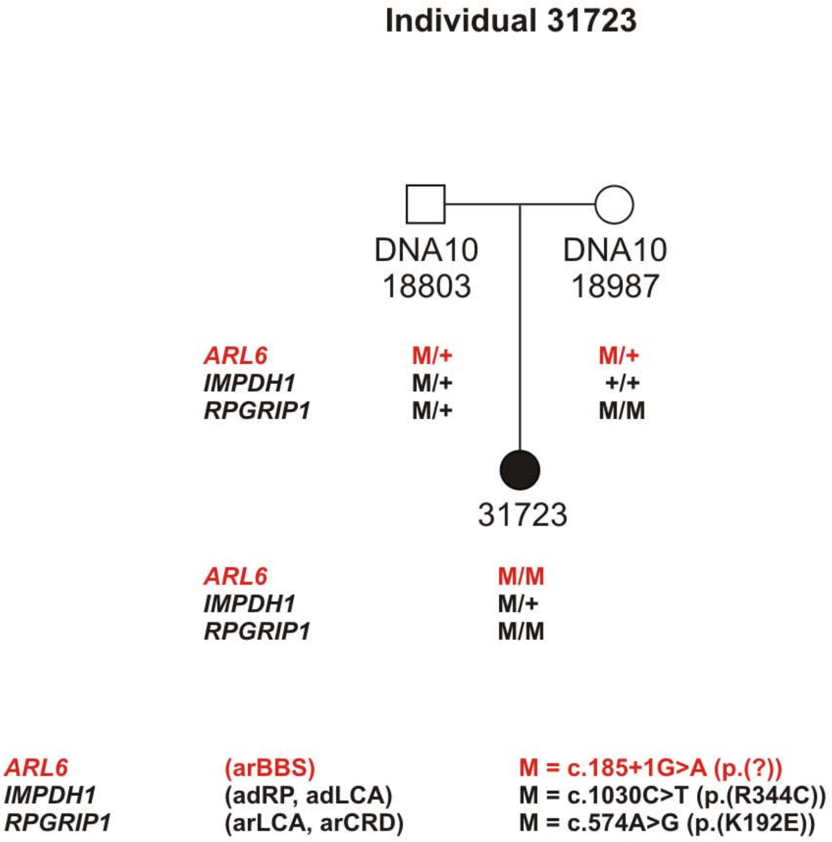
Individual 31723
DNA10 18803
DNA10 18987
ARL6 M/+ M/+
IMPDH1 M/+ +/+
RPGRIP1 M/+ M/M
31723
ARL6 M/M
IMPDH1 M/+
RPGRIP1 M/M
ARL6 (arBBS) M = c.185+1G>A (p.(?))
IMPDH1 (adRP, adLCA) M = c.1030C>T (p.(R344C))
RPGRIP1 (arLCA, arCRD) M = c.574A>G (p.(K192E))

Individual 31910

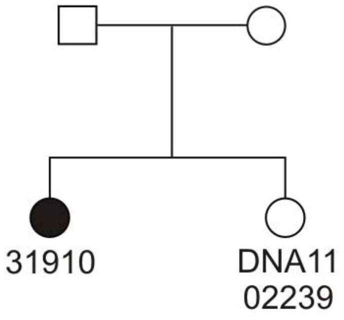

EYS	**M/M**	**+/+**

EYS **(arRP)** **M = c.2309A>C (p.(Q770P))**

Individual 31933

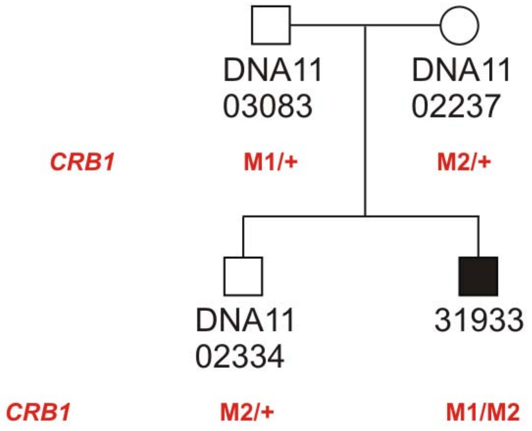

CRB1 (arRP, arLCA, adPPCA) M1 = c.1602G>T (p.(K534N))
M2 = c.2234C>T (p.(T745M))

Individual 31994

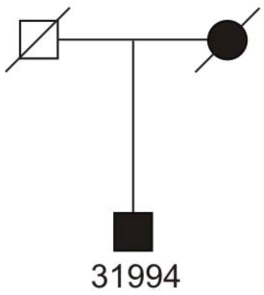

AIPL1	M/+
RHO	M/+

AIPL1	(arLCA, adCRD)	M = c.286G>A (p.(V96I))
RHO	(arRP, adRP)	M = c.641T>A (p.(I214N))

Individual 32111

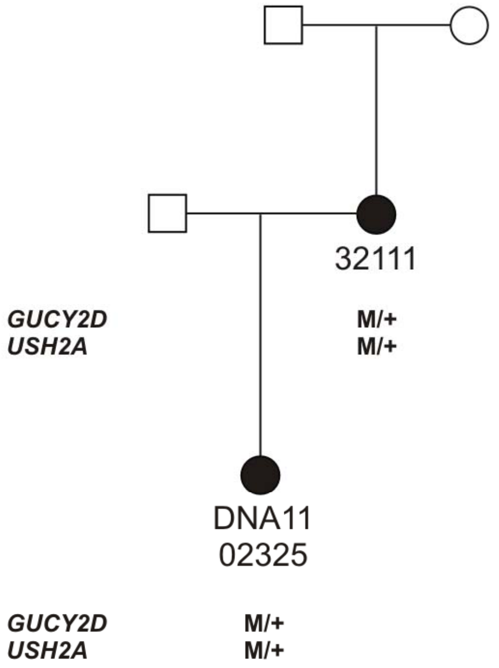

GUCY2D	**(arLCA, adCRD)**	**M = c.154G>T (p.(A52S))**
USH2A	**(arRP, arUSH)**	**M = c.[486-13G>A;6236A>C;6240G>T;12445T>C] (p.[(?);(K2079T);(K2080N);(W4149R)])**

Individual 32594

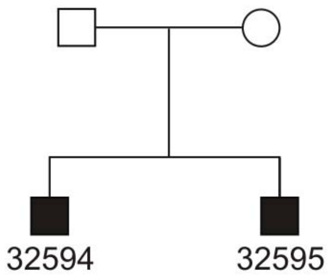

	32594	32595
AIPL1	**M/+**	**+/+**
C2ORF71	**M1/M2**	**M1/M1**
CFH	**M1/M2**	**+/+**
GPR98	**M/+**	**+/+**
NRL	**M1/M2**	**M1/M2**

AIPL1	**(arLCA, adCRD)**	**M = c.932G>T (p.(R311L))**
C2ORF71	**(arRP)**	**M1 = c.[755C>A;3265_3267del] (p.[(A252D);(P1089del)])**
		**M2 = c.3265_3267del (p.(P1089del))**
CFH	**(arMD)**	**M1 = c.2669G>T (p.S890I))**
		**M2 = c.3019G>T (p.(V1007L))**
GPR98	**(arUSH)**	**M = c.[9607T>A;15440A>G] (p.[(S3203T);(D5147G)])**
NRL	**(arRP, arCPRD)**	**M1 = c.508C>A (p.(R170S))**
		**M2 = c.654del (p.(C219fs))**

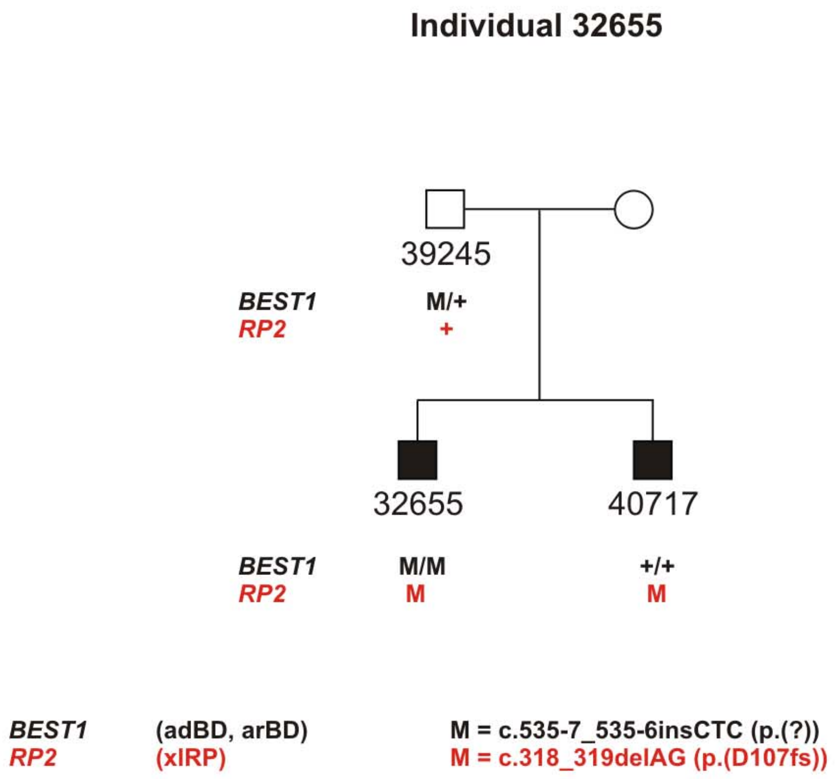
Individual 32655
39245
BEST1
RP2
M/+
+
32655
40717
BEST1
RP2
M/M
M
+/+
M
BEST1 (adBD, arBD) M = c.535-7_535-6insCTC (p.(?))
RP2 (xlRP) M = c.318_319delAG (p.(D107fs))

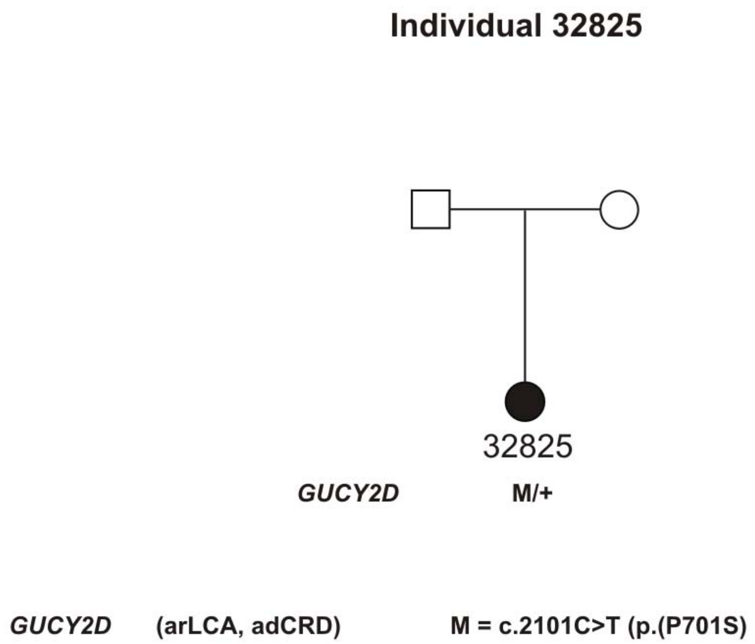
Individual 32825
32825
GUCY2D M/+
GUCY2D (arLCA, adCRD) M = c.2101C>T (p.(P701S)

Individual 33626

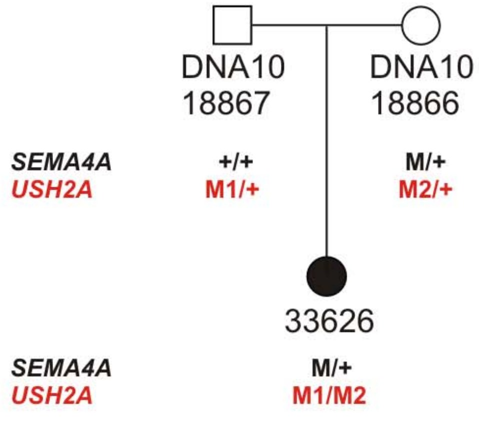

SEMA4A	(adRP)	M = c.2138G>A (p.(R713Q))
USH2A	(arRP, arUSH)	M1 = c.1227G>A (p.(W409*))
		M2 = c.12575G>A (p.(R4192H))

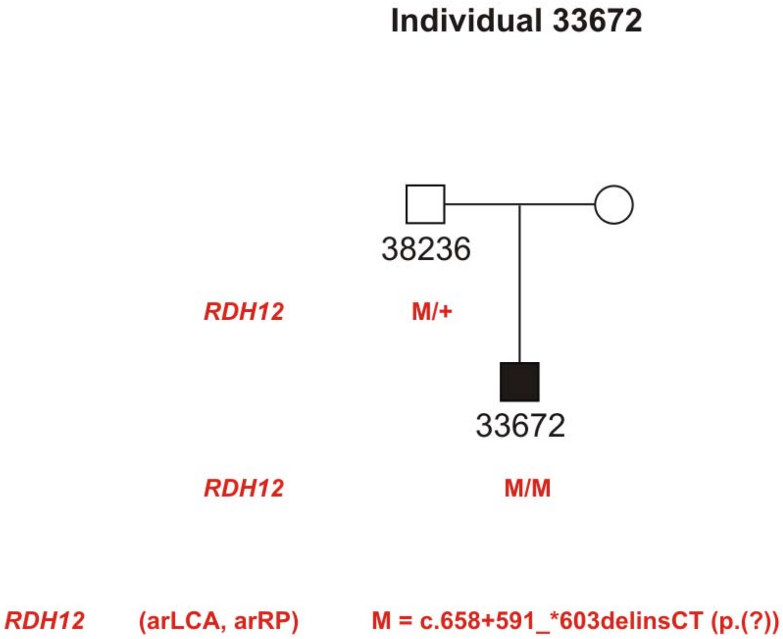
Individual 33672
38236
RDH12
M/+
33672
RDH12
M/M
RDH12
(arLCA, arRP)
M = c.658+591_*603delinsCT (p.(?))

Individual 33884

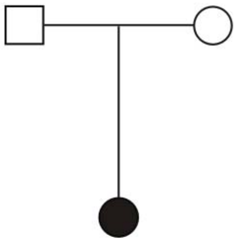

CA4	M/+
ELOVL4	M/+

CA4	(adRP)	M = c.700G>A (p.(V234I))
ELOVL4	(adMD)	M = c.800T>C (p.(I267T))

Individual 34624

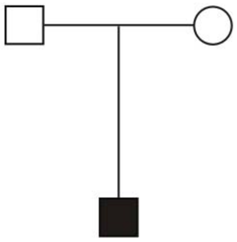

CHM **(xlCHM)** **M = c.265A>T (p.(S89C))**

Individual 34886

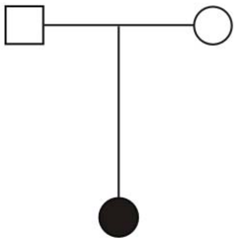

34886

GUCY2D	**M/+**
RP1	**M/+**

GUCY2D	**(arLCA, adCRD)**	**M = c.154G>T (p.(A52S))**
RP1	**(adRP, arRP)**	**M = c.1118C>T (p.(T373I))**

Individual 36832

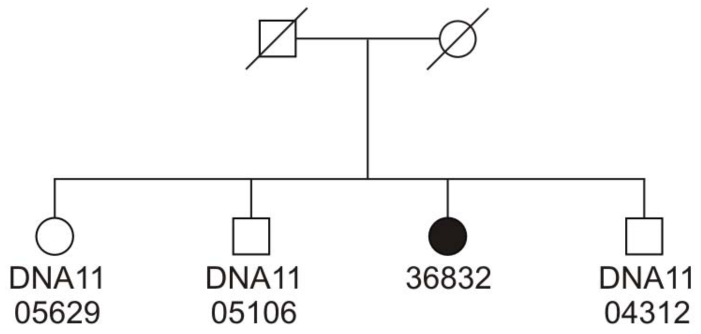

PRPF31	**+/+**	**M/+**	**M/+**	**M/+**

PRPF31 **(adRP)** **M = c.910C>T (p.(R304C))**

Individual 37360

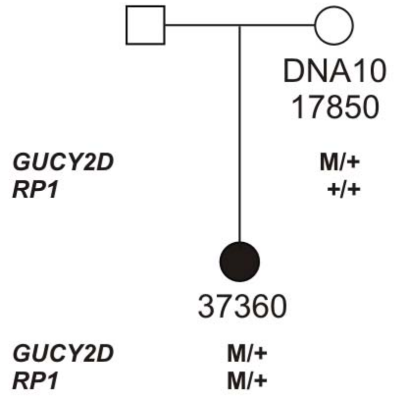

GUCY2D	**(arLCA, adCRD)**	**M = c.1571C>T (p.(A524V))**
RP1	**(adRP, arRP)**	**M = c.4250T>C (p.(L1417P))**

Individual 37370

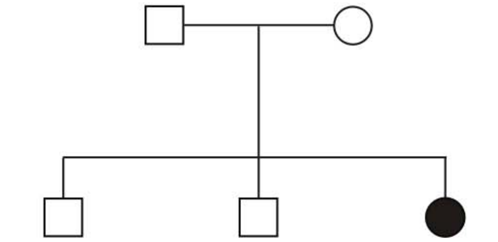

	DNA11 00052	DNA10 19863	37370
BEST1	**M/+**	**M/+**	**M/+**
CRB1	**M/+**	**+/+**	**M/+**
NPHP4	**M1/+**	**+/+**	**M1/M2**
RLBP1	**M/+**	**+/+**	**M/M**

BEST1	**(adBD, arBD)**	**M = c.1699C>T (p.(L567F))**
CRB1	**(arRP, arLCA, adPPCA)**	**M = c.614T>C (p.(I205T))**
NPHP4	**(arNPHP)**	**M1 = c.2902G>A (p.(A968T))**
		**M2 = c.3385G>A (p.(V1129M))**
RLBP1	**(arRP, arFA, arRPA)**	**M = c.525_945del (p.(?))**

Individual 38290

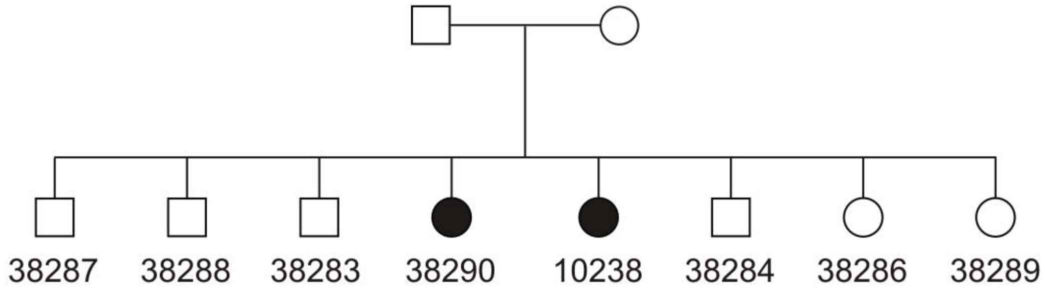

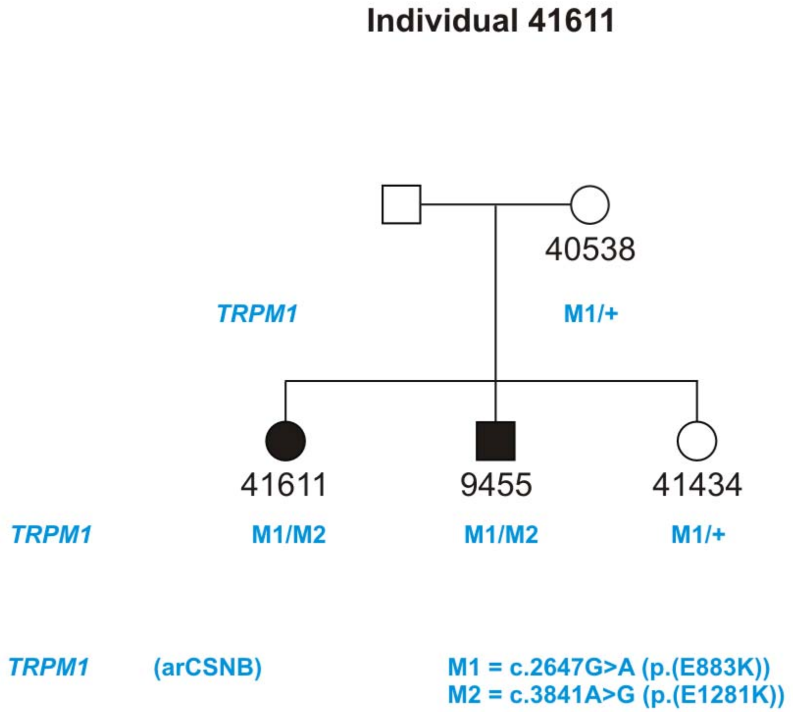

Supp. Figure S3. Pedigrees and segregation analysis of all genetic variants.
Pedigrees are shown for the hundred RP probands, and all available relatives. For all genetic variants that were confirmed with Sanger sequencing, the segregation was determined in all available relatives. Variants that were predicted to be pathogenic or probably pathogenic are depicted in red, whereas variants for which the pathogenicity remains unknown are indicated in blue. In one family (9472), a second mutation is thought to have an additive effect on the phenotype of the proband. This mutation is indicated in green. All variants that were interpreted as benign variants are depicted in black. Abbreviations used: ad; autosomal dominant; ar: autosomal recessive; xl; x-linked; BBS; Bardet-Biedl syndrome; BD: Best disease; CHM; choroideremia; CPRD: clumped pigmentary retinal degeneration; CRD: conerod dystrophy; CS; choroidal sclerosis; CSNB: congenital stationary night blindness; ESCS: enhanced S-cone syndrome; FA; fundus albipunctatus; ID; intellectual disability; JBTS: Joubert syndrome; LCA: Leber congenital amaurosis; MD; macular degeneration; MKS: Meckel-Grüber syndrome; NPHP; nephronophthisis; NSHI; non-syndromic hearing impairment; PPCA: pigmented paravenous chorioretinal atrophy; RP: retinitis pigmentosa; RPA; retinitis punctata albescens; SLS; Senior-Løken syndrome; STGD; Stargardt disease.

Supp. Figure S4

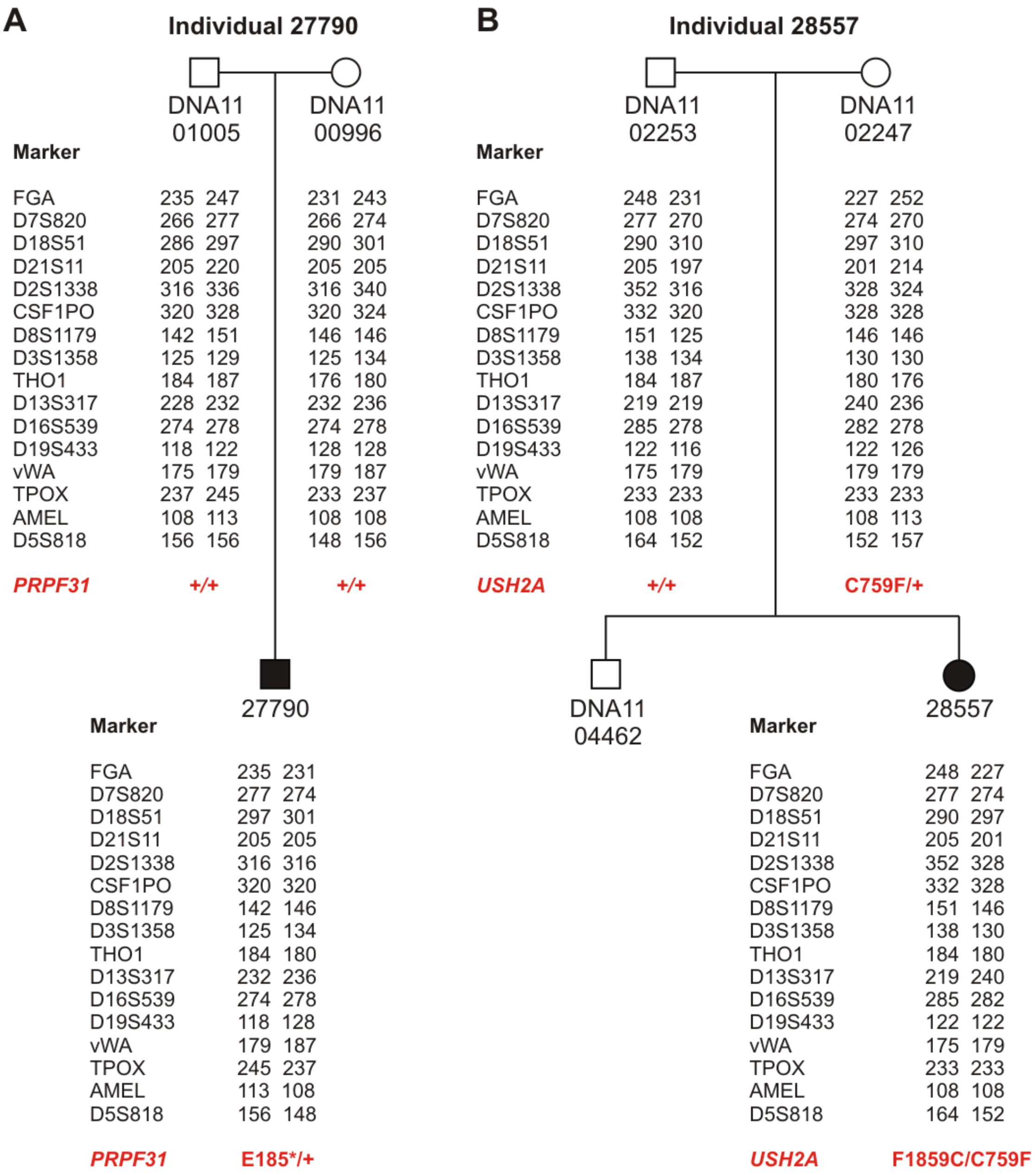

Supp. Figure S4. Parental testing for individuals 27790 and 28557.
Results of the parental testing are shown for individuals 27790 (in **A**) and 28557 (in **B**), who carry *de novo* mutations in *PRPF31* and *USH2A*, respectively. Paternity and maternity was proven by analyzing 16 highly polymorphic markers spread across the genome in both parents and the affected individual. The segregation of the pathogenic mutations (p.E185* in 27790 and p.F1859C in 28857) are depicted in red, and present in neither of the parents.

Supp Figure S5

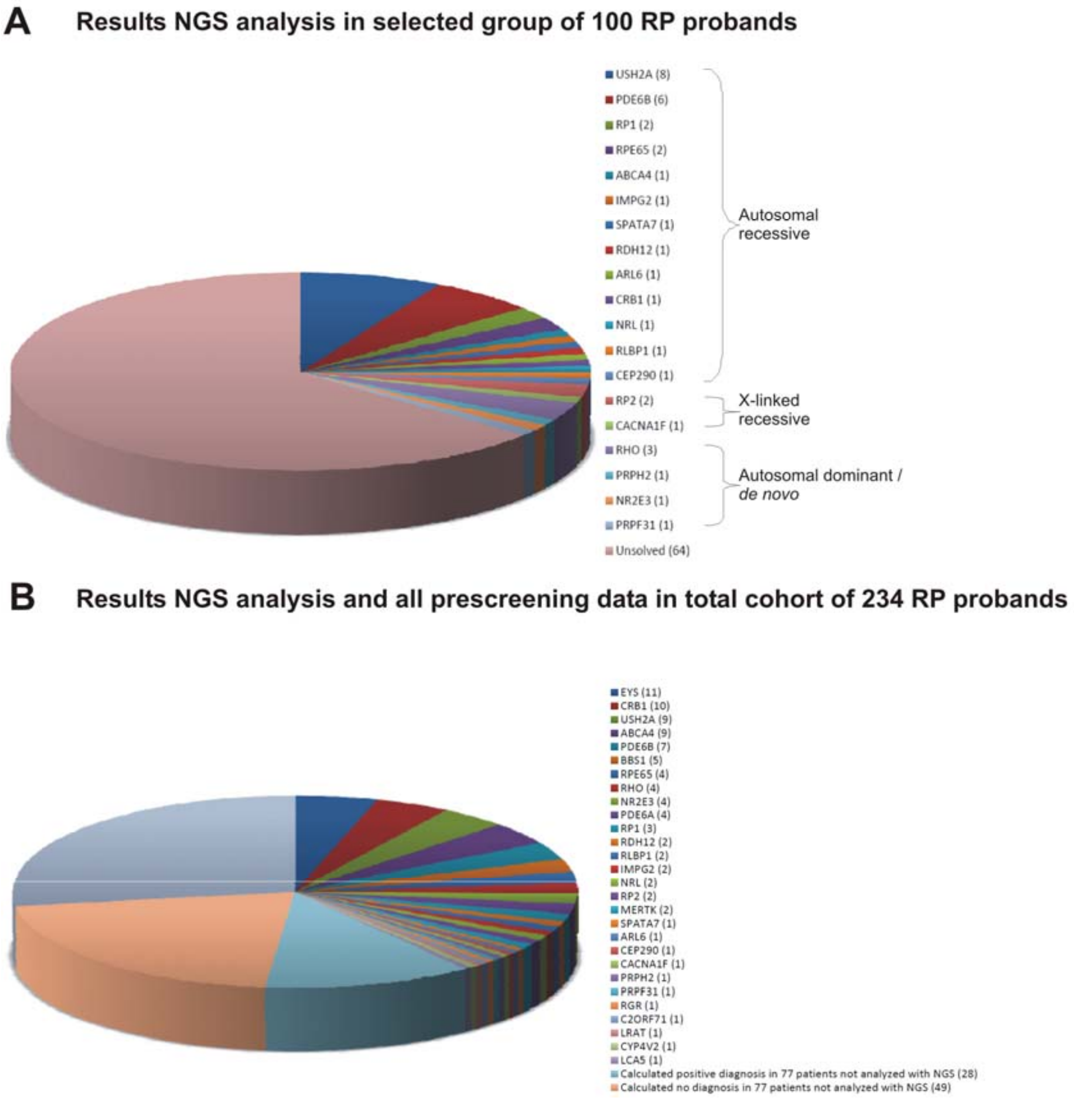

Supp. Figure S5. Summary of the genetic analysis on our complete RP cohort and estimated prevalence of causative genes.
A) Pie chart showing the results of the NGS analysis of 100 RP patients in this study. Pathogenic variants were identified in 36 patients, and were present in 19 different genes, underscoring the extreme heterogeneity of RP. Mutations in 14 genes follow an autosomal recessive inheritance model, four are autosomal dominant or *de novo*, and two are x-linked. **B)** Pie-chart summarizing the combined results of the prescreening analyses (described in Fig. 1) and the targeted NGS analysis performed in this study. Seventy-seven patients have not undergone targeted NGS analysis. We calculated that 28 of these patients will also carry mutations in one of the known retinal dystrophy genes.

Supp. Table S1. Known disease-causing mutations in 12 DNA samples of patients with autosomal recessive retinal dystrophy

Sample	Gene	Allele 1 - cDNA	Allele 1 - protein	Allele 2 - cDNA	Allele 2 - protein	Phenotype
10064	*RDH5*	c.214_215insGTGG	p.(A72fs)	c.914C>G	p.(C267W)	FAP
10559	*CNGB3*	c.1148delC	p.(T383fs)	c.819_826del8bp	p.(R274fs)	ACHM
10719	*CNGA3*	c.1565T>C	p.(I522T)	c.1641C>A	p.(F547L)	ACHM
13079	*NPHP5*	c.424_425delTT	p.(F142fs)	c.1535_1536insATAGC	p.(Q512*)	LCA/SLS
18459	*RPE65*	c.1102T>C	p.(Y368H)	c.11+5G>A	p.(?)	LCA
18872	*CEP290*	c.2991+1655G>A	p.(C998*/wt)[1]	c.451C>T	p.(R151*/ΔExon7/ΔExon7+8)[1]	LCA
21067	*GUCY2D*	c.2302C>T	p.(R768W)	c.2837C>T	p.(A946V)	LCA
21933	*RP1*	c.4241_4242delTA	p.(H1414fs)	c.368_369dupGC	p.(P124fs)	arRP
21974	*GRM6*	c.57_75dupl19	p.(V26*)	c.172G>C	p.(G58R)	arCSNB
29964	*CFH*	c.1222C>T	p.(408*)	c.1204T>C	p.(Y402H)	arBLD
36392	*KCNV2*	c.556_571del16	p.(G186fs)	c.1381G>A	p.(G461R)	arCD
44010	*PDE6C*	c.633G>C	p.(?)	c.1363A>G	p.(M455V)	ACHM

A

bbreviations: **ACHM**, achromatopsia; **arBLD**, autosomal recessive basal laminar drusen; **arCD**, autosomal recessive cone dystrophy; **arCSNB**, autosomal recessive congenital stationary night blindness; **arRP**, autosomal recessive RP; **FAP**, fundus albipunctatus; **LCA**, Leber congenital amaurosis; **SLS**, Senior Loken syndrome. [1]for explanation see Littink KW et al., A novel nonsense mutation in CEP290 induces exon skipping and leads to a relatively mild retinal phenotype, IOVS, 2010. Nucleotide numbering reflects cDNA numbering with +1 corresponding to the A of the ATG translation initiation codon in the reference sequence, according to journal guidelines (www.hgvs.org/mutnomen). The initiation codon is codon 1.

Supp. Table S2. Coverage of sequencing targets

Chromosome	Start position	End position	Size	Med. Coverage	% GC	Gene name	RefSeq Id	Strand	Location
chr1	5845378	5846071	694	30.095	50	*NPHP4*	NM_015102.3	-	Exonic
chr1	5846469	5846741	273	14.41	63.4	*NPHP4*	NM_015102.3	-	Exonic
chr1	5846907	5847223	317	10.32	64.4	*NPHP4*	NM_015102.3	-	Exonic
chr1	5847670	5847981	312	19.85	64.1	*NPHP4*	NM_015102.3	-	Exonic
chr1	5848912	5849199	288	21.15	58.3	*NPHP4*	NM_015102.3	-	Exonic
chr1	5849573	5849854	282	13.995	56.7	*NPHP4*	NM_015102.3	-	Exonic
chr1	5850321	5850604	284	20.24	60.9	*NPHP4*	NM_015102.3	-	Exonic
chr1	5855794	5856080	287	26.25	61	*NPHP4*	NM_015102.3	-	Exonic
chr1	5857038	5857363	326	16.47	62.9	*NPHP4*	NM_015102.3	-	Exonic
chr1	5857440	5857814	375	19.395	63.7	*NPHP4*	NM_015102.3	-	Exonic
chr1	5859664	5860008	345	26.905	63.2	*NPHP4*	NM_015102.3	-	Exonic
chr1	5862683	5862961	279	32.68	52.3	*NPHP4*	NM_015102.3	-	Exonic
chr1	5869857	5870170	314	29.99	64	*NPHP4*	NM_015102.3	-	Exonic
chr1	5873438	5873740	303	30.425	58.4	*NPHP4*	NM_015102.3	-	Exonic
chr1	5887184	5887513	330	30.905	53	*NPHP4*	NM_015102.3	-	Exonic
chr1	5887861	5888187	327	41.375	51.4	*NPHP4*	NM_015102.3	-	Exonic
chr1	5888199	5888479	281	22.975	59.1	*NPHP4*	NM_015102.3	-	Exonic
chr1	5889666	5889943	278	5.22	60.1	*NPHP4*	NM_015102.3	-	Exonic
chr1	5891684	5891965	282	7.15	62.8	*NPHP4*	NM_015102.3	-	Exonic
chr1	5910219	5910501	283	15.92	58.3	*NPHP4*	NM_015102.3	-	Exonic
chr1	5915716	5916043	328	21.96	49.4	*NPHP4*	NM_015102.3	-	Exonic
chr1	5929663	5929948	286	24.495	55.2	*NPHP4*	NM_015102.3	-	Exonic
chr1	5930641	5930965	325	33.74	59.7	*NPHP4*	NM_015102.3	-	Exonic
chr1	5935269	5935545	277	6.58	61.4	*NPHP4*	NM_015102.3	-	Exonic
chr1	5944365	5944662	298	31.625	52	*NPHP4*	NM_015102.3	-	Exonic
chr1	5949827	5950097	271	19.03	57.9	*NPHP4*	NM_015102.3	-	Exonic
chr1	5951683	5951968	286	16.875	51.7	*NPHP4*	NM_015102.3	-	Exonic
chr1	5960837	5961124	288	21.42	58.7	*NPHP4*	NM_015102.3	-	Exonic
chr1	5968721	5969041	321	25.44	53.6	*NPHP4*	NM_015102.3	-	Exonic
chr1	5974814	5975176	363	14.095	74.1	*NPHP4*	NM_015102.3	-	Exonic
chr1	68667014	68667115	102	13.765	25.5	*RPE65*	NM_000329.2	-	UTR
chr1	68667139	68668240	1102	25.26	31.1	*RPE65*	NM_000329.2	-	Exonic
chr1	68669242	68669529	288	57.535	37.5	*RPE65*	NM_000329.2	-	Exonic
chr1	68669425	68669934	510	56.47	40	*RPE65*	NM_000329.2	-	Exonic
chr1	68676372	68676658	287	30.255	35.2	*RPE65*	NM_000329.2	-	Exonic
chr1	68677146	68677391	246	31.19	37.8	*RPE65*	NM_000329.2	-	Exonic
chr1	68677362	68677666	305	35.335	36.1	*RPE65*	NM_000329.2	-	Exonic
chr1	68677727	68677966	240	29.735	36.7	*RPE65*	NM_000329.2	-	Exonic
chr1	68679048	68679327	280	30.715	38.2	*RPE65*	NM_000329.2	-	Exonic
chr1	68682721	68683002	282	52.58	37.9	*RPE65*	NM_000329.2	-	Exonic

chr1	68682921	68683233	313	45.015	40.9	*RPE65*	NM_000329.2	-	Exonic
chr1	68684905	68685189	285	17.955	52.3	*RPE65*	NM_000329.2	-	Exonic
chr1	68686790	68687071	282	13.5	51.8	*RPE65*	NM_000329.2	-	Exonic
chr1	68688050	68688322	273	28.93	44.3	*RPE65*	NM_000329.2	-	Exonic
chr1	94230906	94231453	548	28.485	46.9	*ABCA4*	NM_000350.2	-	Exonic
chr1	94234146	94234430	285	19.05	51.2	*ABCA4*	NM_000350.2	-	Exonic
chr1	94235936	94236321	386	14.91	55.2	*ABCA4*	NM_000350.2	-	Exonic
chr1	94238878	94239317	440	17.72	59.3	*ABCA4*	NM_000350.2	-	Exonic
chr1	94239923	94240196	274	34.385	56.9	*ABCA4*	NM_000350.2	-	Exonic
chr1	94243505	94243788	284	38.125	41.2	*ABCA4*	NM_000350.2	-	Exonic
chr1	94245682	94245963	282	24.21	53.5	*ABCA4*	NM_000350.2	-	Exonic
chr1	94246259	94246535	277	15.085	51.3	*ABCA4*	NM_000350.2	-	Exonic
chr1	94246808	94247091	284	40.73	44	*ABCA4*	NM_000350.2	-	Exonic
chr1	94248863	94249140	278	26.11	57.6	*ABCA4*	NM_000350.2	-	Exonic
chr1	94249322	94249594	273	13.085	61.2	*ABCA4*	NM_000350.2	-	Exonic
chr1	94252607	94252895	289	38.32	45	*ABCA4*	NM_000350.2	-	Exonic
chr1	94253793	94254068	276	17.35	53.3	*ABCA4*	NM_000350.2	-	Exonic
chr1	94257650	94257965	316	13.23	55.7	*ABCA4*	NM_000350.2	-	Exonic
chr1	94259305	94259620	316	22.765	52.2	*ABCA4*	NM_000350.2	-	Exonic
chr1	94259674	94259878	205	40.885	36.1	*ABCA4*	NM_000350.2	-	Exonic
chr1	94259895	94260147	253	34.325	45.8	*ABCA4*	NM_000350.2	-	Exonic
chr1	94261400	94261681	282	42.36	37.6	*ABCA4*	NM_000350.2	-	Exonic
chr1	94262999	94263275	277	34.48	40.1	*ABCA4*	NM_000350.2	-	Exonic
chr1	94267509	94267833	325	9.78	57.5	*ABCA4*	NM_000350.2	-	Exonic
chr1	94268474	94268761	288	17.41	57.3	*ABCA4*	NM_000350.2	-	Exonic
chr1	94269052	94269343	292	19.365	53.1	*ABCA4*	NM_000350.2	-	Exonic
chr1	94269846	94270248	403	10.85	58.1	*ABCA4*	NM_000350.2	-	Exonic
chr1	94274802	94275037	236	13.28	44.1	*ABCA4*	NM_000350.2	-	Exonic
chr1	94275213	94275544	332	41.59	45.8	*ABCA4*	NM_000350.2	-	Exonic
chr1	94278082	94278366	285	26.92	53.7	*ABCA4*	NM_000350.2	-	Exonic
chr1	94279273	94279607	335	19.645	57.6	*ABCA4*	NM_000350.2	-	Exonic
chr1	94280824	94281106	283	16.45	57.6	*ABCA4*	NM_000350.2	-	Exonic
chr1	94281404	94281683	280	21.425	57.5	*ABCA4*	NM_000350.2	-	Exonic
chr1	94282677	94282960	284	18.705	59.2	*ABCA4*	NM_000350.2	-	Exonic
chr1	94284982	94285293	312	26.325	50.3	*ABCA4*	NM_000350.2	-	Exonic
chr1	94286909	94287190	282	20.355	52.5	*ABCA4*	NM_000350.2	-	Exonic
chr1	94289671	94289937	267	32.79	41.2	*ABCA4*	NM_000350.2	-	Exonic
chr1	94293176	94293529	354	33.335	50.8	*ABCA4*	NM_000350.2	-	Exonic
chr1	94294668	94295034	367	20.19	56.1	*ABCA4*	NM_000350.2	-	Exonic
chr1	94298600	94298969	370	30.22	45.7	*ABCA4*	NM_000350.2	-	Exonic
chr1	94300645	94300956	312	20.235	58	*ABCA4*	NM_000350.2	-	Exonic
chr1	94301189	94301526	338	26.395	47	*ABCA4*	NM_000350.2	-	Exonic
chr1	94315754	94316096	343	23.435	50.4	*ABCA4*	NM_000350.2	-	Exonic
chr1	94316641	94316924	284	22.235	47.9	*ABCA4*	NM_000350.2	-	Exonic
chr1	94317409	94317671	263	26	46.8	*ABCA4*	NM_000350.2	-	Exonic

chr1	94318556	94318917	362	31.445	47.2	*ABCA4*	NM_000350.2	-	Exonic
chr1	94321395	94321621	227	19.705	33.9	*ABCA4*	NM_000350.2	-	Exonic
chr1	94336862	94337199	338	14.91	58.3	*ABCA4*	NM_000350.2	-	Exonic
chr1	94341076	94341349	274	18.455	44.5	*ABCA4*	NM_000350.2	-	Exonic
chr1	94346644	94346912	269	23.095	45.4	*ABCA4*	NM_000350.2	-	Exonic
chr1	94349505	94349790	286	19.995	50.7	*ABCA4*	NM_000350.2	-	Exonic
chr1	94351013	94351289	277	25.2	45.1	*ABCA4*	NM_000350.2	-	Exonic
chr1	94359048	94359355	308	21.66	48.4	*ABCA4*	NM_000350.2	-	Exonic
chr1	109947364	109947752	389	39.995	37	*GNAT2*	NM_005272.3	-	Exonic
chr1	109948057	109948311	255	33.935	37.6	*GNAT2*	NM_005272.3	-	Exonic
chr1	109950039	109950311	273	46.06	49.1	*GNAT2*	NM_005272.3	-	Exonic
chr1	109950367	109950653	287	41.945	45.6	*GNAT2*	NM_005272.3	-	Exonic
chr1	109952700	109952990	291	54.835	51.2	*GNAT2*	NM_005272.3	-	Exonic
chr1	109954104	109954393	290	26.81	47.2	*GNAT2*	NM_005272.3	-	Exonic
chr1	109954491	109954758	268	22.23	48.1	*GNAT2*	NM_005272.3	-	Exonic
chr1	109956819	109957295	477	23.8	48.8	*GNAT2*	NM_005272.3	-	Exonic
chr1	148560473	148560737	265	11.035	65.3	*PRPF3*	NM_004698.2	+	Exonic
chr1	148563966	148564229	264	8.085	45.1	*PRPF3*	NM_004698.2	+	Exonic
chr1	148564747	148565035	289	24.36	44.6	*PRPF3*	NM_004698.2	+	Exonic
chr1	148567322	148567610	289	9.16	49.1	*PRPF3*	NM_004698.2	+	Exonic
chr1	148571671	148571952	282	45.89	40.4	*PRPF3*	NM_004698.2	+	Exonic
chr1	148572013	148572230	218	46.435	47.7	*PRPF3*	NM_004698.2	+	Exonic
chr1	148572251	148572355	105	23.52	44.8	*PRPF3*	NM_004698.2	+	Exonic
chr1	148573954	148574399	446	30.705	43.5	*PRPF3*	NM_004698.2	+	Exonic
chr1	148577184	148577480	297	17.395	37.7	*PRPF3*	NM_004698.2	+	Exonic
chr1	148579390	148579534	145	39.22	35.9	*PRPF3*	NM_004698.2	+	Exonic
chr1	148579536	148579678	143	38.07	38.5	*PRPF3*	NM_004698.2	+	Exonic
chr1	148582358	148582614	257	9.345	41.6	*PRPF3*	NM_004698.2	+	Exonic
chr1	148583166	148583294	129	29.075	35.7	*PRPF3*	NM_004698.2	+	Exonic
chr1	148583296	148583436	141	28.84	51.1	*PRPF3*	NM_004698.2	+	Exonic
chr1	148583445	148583725	281	24.535	45.2	*PRPF3*	NM_004698.2	+	Exonic
chr1	148585030	148585311	282	26.385	46.5	*PRPF3*	NM_004698.2	+	Exonic
chr1	148585399	148585682	284	20.15	47.2	*PRPF3*	NM_004698.2	+	Exonic
chr1	148588138	148588418	281	24.355	35.2	*PRPF3*	NM_004698.2	+	Exonic
chr1	148591854	148592042	189	17.83	36.5	*PRPF3*	NM_004698.2	+	Exonic
chr1	148592096	148592386	291	16.75	43.3	*PRPF3*	NM_004698.2	+	UTR
chr1	154389931	154390247	317	9.025	60.3	*SEMA4A*	NM_022367.3	+	Exonic
chr1	154390889	154391191	303	9.785	63.4	*SEMA4A*	NM_022367.3	+	Exonic
chr1	154392751	154393051	301	11.63	55.5	*SEMA4A*	NM_022367.3	+	Exonic
chr1	154394370	154394651	282	27.995	52.1	*SEMA4A*	NM_022367.3	+	Exonic
chr1	154394704	154394988	285	37.84	53.7	*SEMA4A*	NM_022367.3	+	Exonic
chr1	154395036	154395317	282	22.69	51.1	*SEMA4A*	NM_022367.3	+	Exonic
chr1	154396765	154397045	281	26.865	55.9	*SEMA4A*	NM_022367.3	+	Exonic
chr1	154397232	154397509	278	10.715	61.9	*SEMA4A*	NM_022367.3	+	Exonic
chr1	154397685	154397997	313	5.36	70	*SEMA4A*	NM_022367.3	+	Exonic

chr1	154399283	154399565	283	15.7	53.4	*SEMA4A*	NM_022367.3	+	Exonic
chr1	154409160	154409485	326	10.225	58.3	*SEMA4A*	NM_022367.3	+	Exonic
chr1	154411150	154411428	279	14.845	59.9	*SEMA4A*	NM_022367.3	+	Exonic
chr1	154411389	154411710	322	15.35	62.4	*SEMA4A*	NM_022367.3	+	Exonic
chr1	154411905	154412154	250	13.11	55.2	*SEMA4A*	NM_022367.3	+	Exonic
chr1	154412741	154414222	1482	28.78	54.8	*SEMA4A*	NM_022367.3	+	Exonic
chr1	194887565	194887984	420	30.665	37.4	*CFH*	NM_000186.3	+	Exonic
chr1	194908650	194908978	329	18.57	32.8	*CFH*	NM_000186.3	+	Exonic
chr1	194909515	194909788	274	10.65	31.4	*CFH*	NM_000186.3	+	Exonic
chr1	194911634	194911906	273	22.9	30	*CFH*	NM_000186.3	+	Exonic
chr1	194913158	194913467	310	33.995	33.5	*CFH*	NM_000186.3	+	Exonic
chr1	194915297	194915604	308	23.53	30.8	*CFH*	NM_000186.3	+	Exonic
chr1	194920736	194921047	312	22.035	33.7	*CFH*	NM_000186.3	+	Exonic
chr1	194925849	194925928	80	26.525	40	*CFH*	NM_000186.3	+	Exonic
chr1	194925970	194926058	89	29.715	38.2	*CFH*	NM_000186.3	+	Exonic
chr1	194949502	194949709	208	23.17	33.7	*CFH*	NM_000186.3	+	Exonic
chr1	194951265	194951581	317	34.32	32.2	*CFH*	NM_000186.3	+	Exonic
chr1	194960793	194961112	320	37.55	33.4	*CFH*	NM_000186.3	+	Exonic
chr1	194962143	194962461	319	24.08	30.1	*CFH*	NM_000186.3	+	Exonic
chr1	194962407	194962750	344	25.565	32.6	*CFH*	NM_000186.3	+	Exonic
chr1	194964078	194964334	257	16.28	29.2	*CFH*	NM_000186.3	+	Exonic
chr1	194972561	194972820	260	16.145	34.6	*CFH*	NM_000186.3	+	Exonic
chr1	194973182	194973471	290	20.94	33.8	*CFH*	NM_000186.3	+	Exonic
chr1	194976291	194976359	69	22.38	21.7	*CFH*	NM_000186.3	+	Splice site
chr1	194976365	194976611	247	29.045	36.4	*CFH*	NM_000186.3	+	Exonic
chr1	194977550	194977870	321	18.65	38.6	*CFH*	NM_000186.3	+	Exonic
chr1	194979163	194979279	117	22.77	39.3	*CFH*	NM_000186.3	+	Exonic
chr1	195503952	195504286	335	21.61	46.6	*CRB1*	NM_201253.2	+	Exonic
chr1	195564124	195564815	692	46.855	43.2	*CRB1*	NM_201253.2	+	Exonic
chr1	195579953	195580292	340	22.875	43.5	*CRB1*	NM_201253.2	+	Exonic
chr1	195583016	195583287	272	43.75	37.9	*CRB1*	NM_201253.2	+	Exonic
chr1	195592514	195592834	321	24.32	45.5	*CRB1*	NM_201253.2	+	Exonic
chr1	195656672	195657772	1101	46.875	44.7	*CRB1*	NM_201253.2	+	Exonic
chr1	195663171	195663808	638	56.19	39.7	*CRB1*	NM_201253.2	+	Exonic
chr1	195665121	195665434	314	29.755	47.8	*CRB1*	NM_201253.2	+	Exonic
chr1	195670382	195671432	1051	47.34	39.8	*CRB1*	NM_201253.2	+	Exonic
chr1	195674215	195674499	285	41.135	40	*CRB1*	NM_201253.2	+	Exonic
chr1	195677833	195678117	285	26.01	44.9	*CRB1*	NM_201253.2	+	Exonic
chr1	195713336	195714263	928	36.82	40.5	*CRB1*	NM_201253.2	+	Exonic
chr1	209716431	209717151	721	31.85	44.5	*RD3*	NM_001164688.1	-	UTR
chr1	209717426	209719334	1909	22.18	53.4	*RD3*	NM_183059.2	-	Exonic
chr1	209721008	209721452	445	20.91	61.6	*RD3*	NM_183059.2	-	Exonic
chr1	209731657	209731831	175	12.57	57.7	*RD3*	NM_001164688.1	-	UTR
chr1	209731832	209732018	187	18.305	50.8	*RD3*	NM_001164688.1	-	UTR

chr1	209732047	209732925	879	26.83	53.6	*RD3*	NM_001164688.1	-	UTR
chr1	213862778	213864734	1957	34.93	35.8	*USH2A*	NM_206933.2	-	UTR
chr1	213864748	213865884	1137	34.8	34.6	*USH2A*	NM_206933.2	-	Exonic
chr1	213868703	213869065	363	26.135	48.5	*USH2A*	NM_206933.2	-	Exonic
chr1	213874348	213874710	363	33.96	44.1	*USH2A*	NM_206933.2	-	Exonic
chr1	213879014	213879301	288	26.68	37.5	*USH2A*	NM_206933.2	-	Exonic
chr1	213880447	213880754	308	23.26	51.9	*USH2A*	NM_206933.2	-	Exonic
chr1	213887416	213887757	342	23.145	50.3	*USH2A*	NM_206933.2	-	Exonic
chr1	213888417	213888792	376	19.145	51.6	*USH2A*	NM_206933.2	-	Exonic
chr1	213890488	213890825	338	9.67	50.9	*USH2A*	NM_206933.2	-	Exonic
chr1	213910869	213911320	452	24.61	41.6	*USH2A*	NM_206933.2	-	Exonic
chr1	213913987	213915648	1662	34.505	46.4	*USH2A*	NM_206933.2	-	Exonic
chr1	213920038	213920377	340	38.565	41.8	*USH2A*	NM_206933.2	-	Exonic
chr1	213967918	213968410	493	39.96	46	*USH2A*	NM_206933.2	-	Exonic
chr1	213981269	213981517	249	25.885	37.8	*USH2A*	NM_206933.2	-	Exonic
chr1	213983065	213983315	251	9.43	38.6	*USH2A*	NM_206933.2	-	Exonic
chr1	213998549	213998739	191	22.84	34	*USH2A*	NM_206933.2	-	Exonic
chr1	213999545	213999867	323	38.085	39.3	*USH2A*	NM_206933.2	-	Exonic
chr1	214006552	214006820	269	26.57	40.1	*USH2A*	NM_206933.2	-	Exonic
chr1	214019727	214020072	346	21.925	48.6	*USH2A*	NM_206933.2	-	Exonic
chr1	214021927	214022222	296	44.115	39.5	*USH2A*	NM_206933.2	-	Exonic
chr1	214022627	214022962	336	18.01	36.3	*USH2A*	NM_206933.2	-	Exonic
chr1	214026556	214026900	345	35.33	44.3	*USH2A*	NM_206933.2	-	Exonic
chr1	214029948	214030297	350	44.24	36	*USH2A*	NM_206933.2	-	Exonic
chr1	214038792	214039129	338	38.6	46.7	*USH2A*	NM_206933.2	-	Exonic
chr1	214053622	214053931	310	30.13	40.3	*USH2A*	NM_206933.2	-	Exonic
chr1	214056891	214057223	333	31.1	38.7	*USH2A*	NM_206933.2	-	Exonic
chr1	214077863	214078140	278	39.65	36	*USH2A*	NM_206933.2	-	Exonic
chr1	214084179	214084526	348	39.705	39.7	*USH2A*	NM_206933.2	-	Exonic
chr1	214085708	214086062	355	32.43	42	*USH2A*	NM_206933.2	-	Exonic
chr1	214106895	214107195	301	30.52	38.9	*USH2A*	NM_206933.2	-	Exonic
chr1	214117634	214117922	289	36.48	38.4	*USH2A*	NM_206933.2	-	Exonic
chr1	214118648	214119128	481	23.96	42.2	*USH2A*	NM_206933.2	-	Exonic
chr1	214128323	214129061	739	34.135	46.3	*USH2A*	NM_206933.2	-	Exonic
chr1	214139961	214140236	276	46.765	38.4	*USH2A*	NM_206933.2	-	Exonic
chr1	214140654	214140937	284	23.885	46.5	*USH2A*	NM_206933.2	-	Exonic
chr1	214174504	214174788	285	23.975	35.1	*USH2A*	NM_206933.2	-	Exonic
chr1	214205201	214205500	300	23.945	35.3	*USH2A*	NM_206933.2	-	Exonic
chr1	214210510	214210794	285	34.875	40.4	*USH2A*	NM_206933.2	-	Exonic
chr1	214232904	214233194	291	22.33	44.7	*USH2A*	NM_206933.2	-	Exonic
chr1	214238776	214239072	297	18.92	35.7	*USH2A*	NM_206933.2	-	Exonic
chr1	214240291	214240591	301	17.745	44.2	*USH2A*	NM_206933.2	-	Exonic
chr1	214286317	214286605	289	21.015	39.4	*USH2A*	NM_206933.2	-	Exonic
chr1	214288410	214288682	273	30.33	39.2	*USH2A*	NM_206933.2	-	Exonic
chr1	214309990	214310322	333	23.025	42.6	*USH2A*	NM_206933.2	-	Exonic

chr1	214312743	214313030	288	40.935	38.5	*USH2A*	NM_206933.2	-	Exonic
chr1	214312958	214313324	367	33.825	43.6	*USH2A*	NM_206933.2	-	Exonic
chr1	214317973	214318377	405	27.84	40.5	*USH2A*	NM_206933.2	-	Exonic
chr1	214323335	214323619	285	26.83	30.5	*USH2A*	NM_206933.2	-	Exonic
chr1	214324596	214324898	303	35.825	36.3	*USH2A*	NM_206933.2	-	Exonic
chr1	214326595	214326857	263	34.025	37.6	*USH2A*	NM_206933.2	-	Exonic
chr1	214328893	214329125	233	38.47	35.2	*USH2A*	NM_206933.2	-	Exonic
chr1	214336966	214337249	284	33.11	37.3	*USH2A*	NM_206933.2	-	Exonic
chr1	214415140	214415506	367	35.065	40.6	*USH2A*	NM_007123.5	-	Exonic
chr1	214430110	214430350	241	26.88	36.9	*USH2A*	NM_007123.5	-	Exonic
chr1	214436438	214436751	314	22.775	37.3	*USH2A*	NM_007123.5	-	Exonic
chr1	214438244	214438607	364	37.445	39	*USH2A*	NM_007123.5	-	Exonic
chr1	214439515	214440102	588	31.19	43.2	*USH2A*	NM_007123.5	-	Exonic
chr1	214447160	214447463	304	34.275	37.8	*USH2A*	NM_007123.5	-	Exonic
chr1	214457276	214457575	300	48.645	41.3	*USH2A*	NM_007123.5	-	Exonic
chr1	214471837	214472136	300	24.47	37.3	*USH2A*	NM_007123.5	-	Exonic
chr1	214486469	214487250	782	44.865	43	*USH2A*	NM_007123.5	-	Exonic
chr1	214490791	214491125	335	44.32	39.7	*USH2A*	NM_007123.5	-	Exonic
chr1	214529174	214529447	274	49.195	38.7	*USH2A*	NM_007123.5	-	Exonic
chr1	214532064	214532402	339	32.27	37.5	*USH2A*	NM_007123.5	-	Exonic
chr1	214561746	214562023	278	23.61	38.1	*USH2A*	NM_007123.5	-	Exonic
chr1	214563358	214563712	355	26.195	37.2	*USH2A*	NM_007123.5	-	Exonic
chr1	214564052	214564371	320	25.94	31.6	*USH2A*	NM_007123.5	-	Exonic
chr1	214565194	214565623	430	30.025	42.8	*USH2A*	NM_007123.5	-	Exonic
chr1	214567437	214567719	283	38.235	32.9	*USH2A*	NM_007123.5	-	Exonic
chr1	214604838	214605113	276	28.975	30.1	*USH2A*	NM_007123.5	-	Exonic
chr1	214658398	214658704	307	36.995	35.8	*USH2A*	NM_007123.5	-	Exonic
chr1	214661741	214662561	821	38.82	44	*USH2A*	NM_007123.5	-	Exonic
chr1	214663103	214663428	326	28.67	47.5	*USH2A*	NM_007123.5	-	UTR
chr1	241485862	241486220	359	11.445	62.4	*SDCCAG8*	NM_006642.3	+	Exonic
chr1	241499953	241500250	298	43.365	45.6	*SDCCAG8*	NM_006642.3	+	Exonic
chr1	241500800	241501069	270	22.54	35.9	*SDCCAG8*	NM_006642.3	+	Exonic
chr1	241504449	241504660	212	24.83	28.8	*SDCCAG8*	NM_006642.3	+	Exonic
chr1	241516110	241516394	285	26.775	35.8	*SDCCAG8*	NM_006642.3	+	Exonic
chr1	241522931	241523187	257	17.21	37	*SDCCAG8*	NM_006642.3	+	Exonic
chr1	241534544	241534800	257	13.225	25.7	*SDCCAG8*	NM_006642.3	+	Exonic
chr1	241537848	241538163	316	46.99	37.7	*SDCCAG8*	NM_006642.3	+	Exonic
chr1	241546599	241546857	259	25.815	37.8	*SDCCAG8*	NM_006642.3	+	Exonic
chr1	241560388	241560682	295	17.77	39.7	*SDCCAG8*	NM_006642.3	+	Exonic
chr1	241570880	241571156	277	35.19	35.4	*SDCCAG8*	NM_006642.3	+	Exonic
chr1	241574047	241574336	290	33.725	39.3	*SDCCAG8*	NM_006642.3	+	Exonic
chr1	241608570	241608854	285	30.595	44.2	*SDCCAG8*	NM_006642.3	+	Exonic
chr1	241645543	241645822	280	22.265	49.3	*SDCCAG8*	NM_006642.3	+	Exonic
chr1	241647796	241648066	271	36.46	36.5	*SDCCAG8*	NM_006642.3	+	Exonic
chr1	241656268	241656553	286	29.27	35	*SDCCAG8*	NM_006642.3	+	Exonic

chr1	241718856	241719138	283	22.89	58.7	*SDCCAG8*	NM_006642.3	+	Exonic
chr1	241729600	241730080	481	31.54	41.4	*AKT3*	NM_181690.2	-	Exonic
chr2	29137985	29141502	3518	32.02	53	*C2orf71*	NM_001029883.2	-	Exonic
chr2	29146888	29148440	1553	11.675	60.7	*C2orf71*	NM_001029883.2	-	Exonic
chr2	29148444	29150690	2247	25.615	54.9	*C2orf71*	NM_001029883.2	-	Exonic
chr2	96303721	96304536	816	33.84	46.3	*SNRNP200*	NM_014014.4	-	Exonic
chr2	96304585	96304664	80	52.265	52.5	*SNRNP200*	NM_014014.4	-	Exonic
chr2	96304585	96304757	173	51.08	54.3	*SNRNP200*	NM_014014.4	-	Exonic
chr2	96304782	96304901	120	40.34	42.5	*SNRNP200*	NM_014014.4	-	Exonic
chr2	96306259	96306397	139	24.315	55.4	*SNRNP200*	NM_014014.4	-	Exonic
chr2	96306418	96306539	122	27.275	63.9	*SNRNP200*	NM_014014.4	-	Exonic
chr2	96306429	96306610	182	26	56.6	*SNRNP200*	NM_014014.4	-	Exonic
chr2	96306665	96306758	94	19.52	48.9	*SNRNP200*	NM_014014.4	-	Exonic
chr2	96306924	96307042	119	18.55	59.7	*SNRNP200*	NM_014014.4	-	Exonic
chr2	96307132	96307244	113	19.05	52.2	*SNRNP200*	NM_014014.4	-	Exonic
chr2	96307159	96307305	147	17.795	52.4	*SNRNP200*	NM_014014.4	-	Exonic
chr2	96307379	96307485	107	13.575	50.5	*SNRNP200*	NM_014014.4	-	Exonic
chr2	96307617	96307891	275	18.185	53.5	*SNRNP200*	NM_014014.4	-	Exonic
chr2	96307936	96308244	309	30.905	57.3	*SNRNP200*	NM_014014.4	-	Exonic
chr2	96308156	96308524	369	29.7	50.7	*SNRNP200*	NM_014014.4	-	Exonic
chr2	96308819	96309104	286	26.27	50.7	*SNRNP200*	NM_014014.4	-	Exonic
chr2	96311183	96311470	288	36.35	54.2	*SNRNP200*	NM_014014.4	-	Exonic
chr2	96312590	96312878	289	33.89	56.7	*SNRNP200*	NM_014014.4	-	Exonic
chr2	96312919	96313245	327	24.21	56.3	*SNRNP200*	NM_014014.4	-	Exonic
chr2	96313164	96313479	316	15.485	57	*SNRNP200*	NM_014014.4	-	Exonic
chr2	96313744	96314111	368	27.75	54.6	*SNRNP200*	NM_014014.4	-	Exonic
chr2	96314566	96314865	300	24.465	52	*SNRNP200*	NM_014014.4	-	Exonic
chr2	96315699	96316014	316	16.92	53.2	*SNRNP200*	NM_014014.4	-	Exonic
chr2	96316072	96316404	333	29.565	53.2	*SNRNP200*	NM_014014.4	-	Exonic
chr2	96316357	96316681	325	29.29	52.9	*SNRNP200*	NM_014014.4	-	Exonic
chr2	96316813	96317078	266	26.95	51.5	*SNRNP200*	NM_014014.4	-	Exonic
chr2	96317232	96317516	285	46.295	53	*SNRNP200*	NM_014014.4	-	Exonic
chr2	96318019	96318300	282	36.885	51.1	*SNRNP200*	NM_014014.4	-	Exonic
chr2	96318403	96318678	276	39.33	50	*SNRNP200*	NM_014014.4	-	Exonic
chr2	96318576	96318900	325	30.115	51.4	*SNRNP200*	NM_014014.4	-	Exonic
chr2	96319184	96319530	347	24.025	50.4	*SNRNP200*	NM_014014.4	-	Exonic
chr2	96319725	96320030	306	45.395	49.7	*SNRNP200*	NM_014014.4	-	Exonic
chr2	96320065	96320347	283	40.515	49.1	*SNRNP200*	NM_014014.4	-	Exonic
chr2	96320761	96321045	285	34.325	48.8	*SNRNP200*	NM_014014.4	-	Exonic
chr2	96321135	96321435	301	26.055	57.8	*SNRNP200*	NM_014014.4	-	Exonic
chr2	96322353	96322629	277	47.745	39	*SNRNP200*	NM_014014.4	-	Exonic
chr2	96322705	96322944	240	36.485	49.6	*SNRNP200*	NM_014014.4	-	Exonic
chr2	96322955	96323034	80	20.68	42.5	*SNRNP200*	NM_014014.4	-	Exonic
chr2	96324872	96325181	310	24.95	54.2	*SNRNP200*	NM_014014.4	-	Exonic
chr2	96325921	96326216	296	25.88	52.7	*SNRNP200*	NM_014014.4	-	Exonic

chr2	96326319	96326594	276	26.41	47.1	*SNRNP200*	NM_014014.4	-	Exonic
chr2	96326752	96327054	303	40.06	49.5	*SNRNP200*	NM_014014.4	-	Exonic
chr2	96326969	96327255	287	37.245	48.8	*SNRNP200*	NM_014014.4	-	Exonic
chr2	96327667	96327940	274	51.1	43.8	*SNRNP200*	NM_014014.4	-	Exonic
chr2	96328022	96328255	234	64.6	46.6	*SNRNP200*	NM_014014.4	-	Exonic
chr2	96328161	96328487	327	53.62	47.4	*SNRNP200*	NM_014014.4	-	Exonic
chr2	96328693	96328974	282	41.51	49.3	*SNRNP200*	NM_014014.4	-	Exonic
chr2	96330344	96330623	280	32.68	41.4	*SNRNP200*	NM_014014.4	-	Exonic
chr2	96330908	96331238	331	57.035	43.8	*SNRNP200*	NM_014014.4	-	Exonic
chr2	96332547	96332859	313	38.375	45.7	*SNRNP200*	NM_014014.4	-	Exonic
chr2	96334089	96334398	310	30.315	48.7	*SNRNP200*	NM_014014.4	-	Exonic
chr2	96334774	96335048	275	27.685	61.5	*SNRNP200*	NM_014014.4	-	Exonic
chr2	98329004	98329463	460	21.81	58.9	*CNGA3*	NM_001079878.1	+	UTR
chr2	98352755	98353036	282	20.105	49.3	*CNGA3*	NM_001298.2	+	Exonic
chr2	98360489	98360773	285	17.115	55.8	*CNGA3*	NM_001298.2	+	Exonic
chr2	98362992	98363314	323	15.15	60.1	*CNGA3*	NM_001298.2	+	Exonic
chr2	98366173	98366442	270	11.605	59.3	*CNGA3*	NM_001298.2	+	Exonic
chr2	98372462	98372741	280	21.4	55	*CNGA3*	NM_001298.2	+	Exonic
chr2	98374662	98374945	284	17.26	60.6	*CNGA3*	NM_001298.2	+	Exonic
chr2	98378663	98381322	2660	33.055	48.6	*CNGA3*	NM_001298.2	+	Exonic
chr2	98381388	98381558	171	23.69	35.7	*CNGA3*	NM_001079878.1	+	UTR
chr2	110238137	110238485	349	14.265	24.1	*NPHP1*	NM_001079878.1	-	UTR
chr2	110238497	110238992	496	14.93	44	*NPHP1*	NM_000272.3	-	Exonic
chr2	110240375	110240661	287	39.38	36.2	*NPHP1*	NM_000272.3	-	Exonic
chr2	110243943	110244212	270	27.605	40	*NPHP1*	NM_000272.3	-	Exonic
chr2	110246453	110246734	282	35.965	34.4	*NPHP1*	NM_000272.3	-	Exonic
chr2	110258307	110258574	268	38.6	37.7	*NPHP1*	NM_000272.3	-	Exonic
chr2	110259249	110259533	285	36.595	34	*NPHP1*	NM_000272.3	-	Exonic
chr2	110261513	110261787	275	37.715	35.3	*NPHP1*	NM_000272.3	-	Exonic
chr2	110262688	110262934	247	37.795	30.8	*NPHP1*	NM_000272.3	-	Exonic
chr2	110264935	110265205	271	26.385	36.9	*NPHP1*	NM_000272.3	-	Exonic
chr2	110274910	110275188	279	29.51	34.4	*NPHP1*	NM_000272.3	-	Exonic
chr2	110276370	110276652	283	39.18	32.9	*NPHP1*	NM_000272.3	-	Exonic
chr2	110277809	110278091	283	36.005	41	*NPHP1*	NM_000272.3	-	Exonic
chr2	110279360	110279663	304	26.72	43.1	*NPHP1*	NM_000272.3	-	Exonic
chr2	110279864	110280102	239	20.745	37.7	*NPHP1*	NM_000272.3	-	Exonic
chr2	110283218	110283498	281	22.165	32	*NPHP1*	NM_000272.3	-	Exonic
chr2	110284595	110284917	323	32.645	40.9	*NPHP1*	NM_000272.3	-	Exonic
chr2	110293260	110293483	224	28.725	35.3	*NPHP1*	NM_000272.3	-	Exonic
chr2	110294369	110294655	287	23.515	29.3	*NPHP1*	NM_000272.3	-	Exonic
chr2	110316177	110316450	274	30.385	30.7	*NPHP1*	NM_000272.3	-	Exonic
chr2	110319685	110319986	302	24.35	68.5	*NPHP1*	NM_000272.3	-	Exonic
chr2	112372584	112372896	313	8.41	75.1	*MERTK*	NM_006343.2	+	Exonic
chr2	112403089	112403607	519	27.285	45.5	*MERTK*	NM_006343.2	+	Exonic
chr2	112418909	112419192	284	36.935	38.4	*MERTK*	NM_006343.2	+	Exonic

chr2	112421375	112421670	296	33.995	48	*MERTK*	NM_006343.2	+	Exonic
chr2	112439131	112439417	287	28.525	51.2	*MERTK*	NM_006343.2	+	Exonic
chr2	112442162	112442375	214	16.41	47.2	*MERTK*	NM_006343.2	+	Exonic
chr2	112449261	112449579	319	38.735	48.3	*MERTK*	NM_006343.2	+	Exonic
chr2	112456810	112457100	291	32.59	44	*MERTK*	NM_006343.2	+	Exonic
chr2	112468223	112468522	300	20.56	51.3	*MERTK*	NM_006343.2	+	Exonic
chr2	112471292	112471581	290	34.875	43.1	*MERTK*	NM_006343.2	+	Exonic
chr2	112475144	112475422	279	41.625	39.8	*MERTK*	NM_006343.2	+	Exonic
chr2	112477042	112477313	272	12.97	29.4	*MERTK*	NM_006343.2	+	Exonic
chr2	112477845	112478130	286	21.83	47.2	*MERTK*	NM_006343.2	+	Exonic
chr2	112482327	112482610	284	21.51	46.8	*MERTK*	NM_006343.2	+	Exonic
chr2	112483906	112484192	287	38.63	43.6	*MERTK*	NM_006343.2	+	Exonic
chr2	112493366	112493649	284	23.72	43.7	*MERTK*	NM_006343.2	+	Exonic
chr2	112495389	112495695	307	39.455	46.6	*MERTK*	NM_006343.2	+	Exonic
chr2	112496226	112496507	282	22.705	52.1	*MERTK*	NM_006343.2	+	Exonic
chr2	112502320	112503083	764	28.18	47.9	*MERTK*	NM_006343.2	+	Exonic
chr2	112503088	112503481	394	14.05	28.9	*MERTK*	NM_006343.2	+	UTR
chr2	170044162	170044439	278	24.97	65.5	*BBS5*	NM_152384.2	+	Exonic
chr2	170046900	170047186	287	31.26	30.3	*BBS5*	NM_152384.2	+	Exonic
chr2	170051711	170051992	282	27.02	33.3	*BBS5*	NM_152384.2	+	Exonic
chr2	170052461	170052718	258	16.63	27.1	*BBS5*	NM_152384.2	+	Exonic
chr2	170052621	170052931	311	17.96	28.3	*BBS5*	NM_152384.2	+	Exonic
chr2	170057557	170057831	275	25.39	31.3	*BBS5*	NM_152384.2	+	Exonic
chr2	170058396	170058645	250	28.035	27.6	*BBS5*	NM_152384.2	+	Exonic
chr2	170062265	170062544	280	20.425	26.4	*BBS5*	NM_152384.2	+	Exonic
chr2	170064158	170064437	280	23.655	30.4	*BBS5*	NM_152384.2	+	Exonic
chr2	170067747	170068024	278	31.65	34.9	*BBS5*	NM_152384.2	+	Exonic
chr2	170068925	170069178	254	22.305	28	*BBS5*	NM_152384.2	+	Exonic
chr2	170069169	170069545	377	18.165	38.7	*BBS5*	NM_152384.2	+	Exonic
chr2	170069626	170069724	99	8.835	60.6	*BBS5*	NM_152384.2	+	UTR
chr2	170071276	170071469	194	8.505	23.7	*BBS5*	NM_152384.2	+	UTR
chr2	182109572	182110880	1309	27.88	32.8	*ITGA4*	NM_000885.4	+	UTR
chr2	182110900	182110981	82	27.31	41.5	*CERKL*	NM_001030312.2	-	UTR
chr2	182111047	182111276	230	25.89	32.2	*CERKL*	NM_001030311.2	-	Exonic
chr2	182111987	182112283	297	33.73	34	*CERKL*	NM_001030311.2	-	Exonic
chr2	182117571	182117857	287	24.65	29.3	*CERKL*	NM_001030311.2	-	Exonic
chr2	182120498	182120829	332	37.03	39.8	*CERKL*	NM_001030311.2	-	Exonic
chr2	182121400	182121877	478	34.815	36.8	*CERKL*	NM_001030311.2	-	Exonic
chr2	182122492	182122781	290	34.915	33.8	*CERKL*	NM_001030311.2	-	Exonic
chr2	182131457	182131742	286	45.78	44.1	*CERKL*	NM_001030311.2	-	Exonic
chr2	182138911	182139105	195	16.045	30.3	*CERKL*	NM_001030311.2	-	Exonic
chr2	182139110	182139190	81	14.555	29.6	*CERKL*	NM_001030311.2	-	Intronic
chr2	182146640	182146914	275	15.985	31.6	*CERKL*	NM_001030311.2	-	Exonic
chr2	182176749	182177100	352	30.995	31.8	*CERKL*	NM_001030311.2	-	Exonic
chr2	182229660	182230147	488	14.885	73.2	*CERKL*	NM_001030311.2	-	Exonic

chr2	233880968	233881469	502	34.035	52.2	*SAG*	NM_000541.4	+	UTR
chr2	233882449	233882734	286	30.155	43	*SAG*	NM_000541.4	+	Exonic
chr2	233889369	233889615	247	15.12	47.8	*SAG*	NM_000541.4	+	Exonic
chr2	233892012	233892281	270	19.79	46.3	*SAG*	NM_000541.4	+	Exonic
chr2	233893968	233894268	301	25.27	60.1	*SAG*	NM_000541.4	+	Exonic
chr2	233896220	233896464	245	10.97	31.8	*SAG*	NM_000541.4	+	Exonic
chr2	233900397	233900680	284	16.065	56.7	*SAG*	NM_000541.4	+	Exonic
chr2	233901780	233902059	280	23.085	60.4	*SAG*	NM_000541.4	+	Exonic
chr2	233902774	233903055	282	33.345	44	*SAG*	NM_000541.4	+	Exonic
chr2	233904910	233905196	287	15.45	55.1	*SAG*	NM_000541.4	+	Exonic
chr2	233908265	233908542	278	33.485	52.5	*SAG*	NM_000541.4	+	Exonic
chr2	233911954	233912226	273	27.41	54.2	*SAG*	NM_000541.4	+	Exonic
chr2	233913706	233913977	272	19.275	49.6	*SAG*	NM_000541.4	+	Exonic
chr2	233915537	233915813	277	18.995	44	*SAG*	NM_000541.4	+	Exonic
chr2	233919647	233919911	265	27.045	44.5	*SAG*	NM_000541.4	+	Exonic
chr2	233920111	233920488	378	32.435	42.9	*SAG*	NM_000541.4	+	Exonic
chr3	50203970	50204310	341	11.625	61.6	*GNAT1*	NM_144499.2	+	Exonic
chr3	50205480	50205897	418	27.975	64.4	*GNAT1*	NM_144499.2	+	Exonic
chr3	50205845	50206154	310	23.955	70.6	*GNAT1*	NM_144499.2	+	Exonic
chr3	50206073	50206379	307	18.145	70.4	*GNAT1*	NM_144499.2	+	Exonic
chr3	50206443	50206727	285	14.235	68.8	*GNAT1*	NM_144499.2	+	Exonic
chr3	50206862	50207149	288	34.55	63.9	*GNAT1*	NM_144499.2	+	Exonic
chr3	50207089	50207446	358	31.635	62.8	*GNAT1*	NM_144499.2	+	Exonic
chr3	50207629	50209939	2311	19.605	53.9	*GNAT1*	NM_000172.3	+	UTR
chr3	98966204	98966569	366	26.945	58.7	*ARL6*	NM_177976.1	+	UTR
chr3	98967428	98967703	276	34.56	32.6	*ARL6*	NM_177976.1	+	UTR
chr3	98969535	98969825	291	24.975	33	*ARL6*	NM_032146.3	+	Exonic
chr3	98981578	98981851	274	31.475	32.5	*ARL6*	NM_032146.3	+	Exonic
chr3	98982032	98982311	280	21.69	28.9	*ARL6*	NM_032146.3	+	Exonic
chr3	98986385	98986672	288	14.475	32.6	*ARL6*	NM_032146.3	+	Exonic
chr3	98989442	98989721	280	34.575	32.5	*ARL6*	NM_032146.3	+	Exonic
chr3	98993183	98993421	239	23.61	32.6	*ARL6*	NM_032146.3	+	Exonic
chr3	98999477	98999910	434	18.6	28.3	*ARL6*	NM_032146.3	+	Exonic
chr3	98999932	99000128	197	8.71	28.4	*ARL6*	NM_177976.1	+	UTR
chr3	102427897	102428550	654	20.845	34.1	*IMPG2*	NM_016247.3	-	Exonic
chr3	102430221	102430490	270	30.515	40.4	*IMPG2*	NM_016247.3	-	Exonic
chr3	102430838	102431189	352	30.965	50	*IMPG2*	NM_016247.3	-	Exonic
chr3	102432413	102432738	326	50.05	45.1	*IMPG2*	NM_016247.3	-	Exonic
chr3	102434249	102434585	337	29.105	44.5	*IMPG2*	NM_016247.3	-	Exonic
chr3	102444142	102444508	367	43.79	39.8	*IMPG2*	NM_016247.3	-	Exonic
chr3	102444987	102446371	1385	55.705	42.4	*IMPG2*	NM_016247.3	-	Exonic
chr3	102447260	102447688	429	14.875	45.7	*IMPG2*	NM_016247.3	-	Exonic
chr3	102455122	102455405	284	30.275	39.8	*IMPG2*	NM_016247.3	-	Exonic
chr3	102458986	102459358	373	49.97	41.8	*IMPG2*	NM_016247.3	-	Exonic
chr3	102468905	102469187	283	25.33	34.3	*IMPG2*	NM_016247.3	-	Exonic

chr3	102470929	102471207	279	22.21	27.2	*IMPG2*	NM_016247.3	-	Exonic
chr3	102475094	102475334	241	27.045	37.3	*IMPG2*	NM_016247.3	-	Exonic
chr3	102477091	102477371	281	44.675	42	*IMPG2*	NM_016247.3	-	Exonic
chr3	102478077	102478353	277	25.53	31.8	*IMPG2*	NM_016247.3	-	Exonic
chr3	102492893	102493152	260	30.455	36.5	*IMPG2*	NM_016247.3	-	Exonic
chr3	102505602	102505903	302	40.775	40.7	*IMPG2*	NM_016247.3	-	Exonic
chr3	102521042	102521430	389	39.41	39.1	*IMPG2*	NM_016247.3	-	Exonic
chr3	102521741	102522175	435	32.15	32.9	*IMPG2*	NM_016247.3	-	Exonic
chr3	122971219	122971523	305	33.345	30.5	*IQCB1*	NM_001023571.2	-	UTR
chr3	122971559	122971643	85	37.075	27.1	*IQCB1*	NM_001023571.2	-	UTR
chr3	122971743	122971828	86	42.49	48.8	*IQCB1*	NM_001023571.2	-	UTR
chr3	122971839	122972043	205	38.55	42.4	*IQCB1*	NM_001023570.2	-	Exonic
chr3	122972074	122972177	104	20.265	47.1	*IQCB1*	NM_001023570.2	-	Exonic
chr3	122974018	122974310	293	34.635	47.4	*IQCB1*	NM_001023570.2	-	Exonic
chr3	122983210	122983474	265	26.04	38.5	*IQCB1*	NM_001023570.2	-	Exonic
chr3	122989741	122989866	126	45.06	38.9	*IQCB1*	NM_001023570.2	-	Exonic
chr3	122989925	122990025	101	33.61	39.6	*IQCB1*	NM_001023570.2	-	Exonic
chr3	122991532	122991817	286	27.345	36	*IQCB1*	NM_001023570.2	-	Exonic
chr3	123008784	123009057	274	22.705	29.2	*IQCB1*	NM_001023570.2	-	Exonic
chr3	123010350	123010595	246	33.73	32.9	*IQCB1*	NM_001023570.2	-	Exonic
chr3	123027502	123027657	156	43.44	30.8	*IQCB1*	NM_001023570.2	-	Exonic
chr3	123027681	123027786	106	35.64	43.4	*IQCB1*	NM_001023570.2	-	Exonic
chr3	123029931	123030228	298	27.02	32.9	*IQCB1*	NM_001023570.2	-	Exonic
chr3	123030304	123030442	139	20.86	30.9	*IQCB1*	NM_001023570.2	-	Exonic
chr3	123030467	123030582	116	15.985	33.6	*IQCB1*	NM_001023570.2	-	Exonic
chr3	123035821	123036103	283	28.455	44.5	*IQCB1*	NM_001023571.2	-	UTR
chr3	123036410	123036691	282	16.17	66.7	*IQCB1*	NM_001023571.2	-	UTR
chr3	130730096	130730683	588	35.72	59.5	*RHO*	NM_000539.3	+	Exonic
chr3	130732329	130732624	296	16.03	62.5	*RHO*	NM_000539.3	+	Exonic
chr3	130733718	130734002	285	36.24	59.3	*RHO*	NM_000539.3	+	Exonic
chr3	130733963	130734372	410	29.24	62.2	*RHO*	NM_000539.3	+	Exonic
chr3	130735065	130735385	321	13.48	58.6	*RHO*	NM_000539.3	+	Exonic
chr3	130735425	130736017	593	18.385	46.7	*RHO*	NM_000539.3	+	UTR
chr3	130736028	130736927	900	25.695	53	*RHO*	NM_000539.3	+	UTR
chr3	133882068	133882798	731	18.04	31.2	*NPHP3*	NM_153240.4	-	UTR
chr3	133883093	133883675	583	35.42	33.8	*NPHP3*	NM_153240.4	-	Exonic
chr3	133884149	133884422	274	30.82	32.8	*NPHP3*	NM_153240.4	-	Exonic
chr3	133884850	133885127	278	24.71	34.2	*NPHP3*	NM_153240.4	-	Exonic
chr3	133886010	133886390	381	33.66	38.6	*NPHP3*	NM_153240.4	-	Exonic
chr3	133887708	133887989	282	28.845	37.2	*NPHP3*	NM_153240.4	-	Exonic
chr3	133888577	133888853	277	19.715	29.2	*NPHP3*	NM_153240.4	-	Exonic
chr3	133890115	133890492	378	33.805	42.6	*NPHP3*	NM_153240.4	-	Exonic
chr3	133890529	133890838	310	45.675	41.3	*NPHP3*	NM_153240.4	-	Exonic
chr3	133891973	133892250	278	23.02	35.6	*NPHP3*	NM_153240.4	-	Exonic
chr3	133892627	133892903	277	25.73	36.8	*NPHP3*	NM_153240.4	-	Exonic
chr3	133894157	133894415	259	31.82	36.3	*NPHP3*	NM_153240.4	-	Exonic

chr3	133896309	133896567	259	36.43	36.7	*NPHP3*	NM_153240.4	-	Exonic
chr3	133898160	133898435	276	30.975	43.8	*NPHP3*	NM_153240.4	-	Exonic
chr3	133898698	133898974	277	23.98	30	*NPHP3*	NM_153240.4	-	Exonic
chr3	133900787	133901066	280	20.965	30	*NPHP3*	NM_153240.4	-	Exonic
chr3	133901373	133901649	277	31.455	40.1	*NPHP3*	NM_153240.4	-	Exonic
chr3	133901775	133902041	267	16.335	34.8	*NPHP3*	NM_153240.4	-	Exonic
chr3	133902868	133903151	284	33.315	40.1	*NPHP3*	NM_153240.4	-	Exonic
chr3	133905666	133905970	305	43.97	39.3	*NPHP3*	NM_153240.4	-	Exonic
chr3	133907161	133907447	287	21.985	30	*NPHP3*	NM_153240.4	-	Exonic
chr3	133909558	133909849	292	42.18	39.4	*NPHP3*	NM_153240.4	-	Exonic
chr3	133914579	133914878	300	19.385	27	*NPHP3*	NM_153240.4	-	Exonic
chr3	133916535	133916816	282	24.455	30.5	*NPHP3*	NM_153240.4	-	Exonic
chr3	133918211	133918496	286	41.48	41.3	*NPHP3*	NM_153240.4	-	Exonic
chr3	133920450	133920738	289	27.835	37	*NPHP3*	NM_153240.4	-	Exonic
chr3	133921166	133921430	93	3.78	29.8	*NPHP3*	NM_153240.4	-	Exonic
chr3	133923428	133923966	539	16.355	71.1	*NPHP3*	NM_153240.4	-	Exonic
chr3	152126963	152127759	797	23.69	39.6	*CLRN1*	NM_052995.2	-	Exonic
chr3	152127918	152128119	202	11.795	28.2	*CLRN1*	NM_052995.2	-	Intronic
chr3	152128138	152128725	588	31.67	37.1	*CLRN1*	NM_174878.2	-	Exonic
chr3	152141983	152142300	318	25.005	39	*CLRN1*	NM_174878.2	-	Exonic
chr3	152172855	152173538	684	38.48	46.6	*CLRN1*	NM_174878.2	-	Exonic
chr4	609292	609940	649	20.185	64.1	*PDE6B*	NM_000283.3	+	Exonic
chr4	618385	618683	299	18.61	59.9	*PDE6B*	NM_000283.3	+	Exonic
chr4	619573	619845	273	27.245	56	*PDE6B*	NM_000283.3	+	Exonic
chr4	637580	638030	451	10.555	64.7	*PDE6B*	NM_001145292.1	+	Exonic
chr4	638549	638769	221	26.73	61.1	*PDE6B*	NM_001145292.1	+	Exonic
chr4	639612	639900	289	26.675	65.4	*PDE6B*	NM_001145292.1	+	Exonic
chr4	639911	640191	281	25.975	40.2	*PDE6B*	NM_001145292.1	+	Exonic
chr4	640582	640874	293	15.365	63.5	*PDE6B*	NM_001145292.1	+	Exonic
chr4	641062	641333	272	9.87	63.6	*PDE6B*	NM_001145292.1	+	Exonic
chr4	642628	642907	280	19.695	60.4	*PDE6B*	NM_001145292.1	+	Exonic
chr4	644177	644458	282	18.87	59.2	*PDE6B*	NM_001145292.1	+	Exonic
chr4	645829	646114	286	19.815	62.2	*PDE6B*	NM_001145292.1	+	Exonic
chr4	646207	646472	266	18.22	59.8	*PDE6B*	NM_001145292.1	+	Exonic
chr4	646792	647068	277	10.655	62.8	*PDE6B*	NM_001145292.1	+	Exonic
chr4	647462	647734	273	13.69	70.7	*PDE6B*	NM_001145292.1	+	Exonic
chr4	647806	648088	283	11.16	64.3	*PDE6B*	NM_001145292.1	+	Exonic
chr4	648596	648833	238	18.22	58.4	*PDE6B*	NM_001145292.1	+	Exonic
chr4	648934	649213	280	20.535	63.2	*PDE6B*	NM_001145292.1	+	Exonic
chr4	650215	650495	281	11.09	65.5	*PDE6B*	NM_001145292.1	+	Exonic
chr4	651567	651861	295	10.855	55.9	*PDE6B*	NM_001145292.1	+	Exonic
chr4	653754	654034	281	20.955	49.5	*PDE6B*	NM_001145292.1	+	Exonic
chr4	654319	654743	425	34.71	43.5	*PDE6B*	NM_001145291.1	+	UTR
chr4	15080561	15080833	273	27.72	47.6	*CC2D2A*	NM_001164720.1	+	UTR
chr4	15083811	15084092	282	35.605	37.2	*CC2D2A*	NM_001164720.1	+	UTR

chr4	15086516	15086781	266	37.38	40.6	*CC2D2A*	NM_001080522.2	+	Exonic
chr4	15089337	15089614	278	29.32	41.7	*CC2D2A*	NM_001080522.2	+	Exonic
chr4	15091342	15091619	278	26.06	54.7	*CC2D2A*	NM_001080522.2	+	Exonic
chr4	15113043	15113099	57	5.3	33.3	*CC2D2A*	NM_001080522.2	+	Intronic
chr4	15113136	15113321	186	19.8	43	*CC2D2A*	NM_001080522.2	+	Exonic
chr4	15113448	15113713	266	28.65	40.2	*CC2D2A*	NM_001080522.2	+	Exonic
chr4	15120764	15120996	233	27.99	37.8	*CC2D2A*	NM_001080522.2	+	Exonic
chr4	15121887	15122206	320	23.825	45.3	*CC2D2A*	NM_001080522.2	+	Exonic
chr4	15125347	15125645	299	41.185	39.5	*CC2D2A*	NM_001080522.2	+	Exonic
chr4	15126566	15126790	225	18.575	46.2	*CC2D2A*	NM_001080522.2	+	Exonic
chr4	15127263	15127541	279	27.38	41.9	*CC2D2A*	NM_001080522.2	+	Exonic
chr4	15138087	15138437	351	47.65	41.9	*CC2D2A*	NM_001080522.2	+	Exonic
chr4	15139254	15139521	268	42.965	39.6	*CC2D2A*	NM_001080522.2	+	Exonic
chr4	15143837	15144111	275	31.205	29.8	*CC2D2A*	NM_001080522.2	+	Exonic
chr4	15147560	15147865	306	29.415	42.2	*CC2D2A*	NM_001080522.2	+	Exonic
chr4	15148550	15148926	377	17.285	53.6	*CC2D2A*	NM_001080522.2	+	Exonic
chr4	15151477	15151768	292	15.57	42.8	*CC2D2A*	NM_001080522.2	+	Exonic
chr4	15161464	15161763	300	39.05	43	*CC2D2A*	NM_001080522.2	+	Exonic
chr4	15163798	15164092	295	31.67	45.4	*CC2D2A*	NM_001080522.2	+	Exonic
chr4	15165716	15165999	284	47.33	44.7	*CC2D2A*	NM_001080522.2	+	Exonic
chr4	15167944	15168293	350	27.32	38.6	*CC2D2A*	NM_001080522.2	+	Exonic
chr4	15169786	15170059	274	31.56	36.5	*CC2D2A*	NM_001080522.2	+	Exonic
chr4	15171152	15171431	280	14.73	27.9	*CC2D2A*	NM_001080522.2	+	Exonic
chr4	15173999	15174311	313	38.87	50.2	*CC2D2A*	NM_001080522.2	+	Exonic
chr4	15178020	15178280	261	24.34	42.1	*CC2D2A*	NM_001080522.2	+	Exonic
chr4	15178307	15178588	282	33.165	35.5	*CC2D2A*	NM_001080522.2	+	Exonic
chr4	15179922	15180188	267	40.575	37.5	*CC2D2A*	NM_001080522.2	+	Exonic
chr4	15181019	15181296	278	24.695	39.6	*CC2D2A*	NM_001080522.2	+	Exonic
chr4	15184790	15185087	298	31.265	40.9	*CC2D2A*	NM_001080522.2	+	Exonic
chr4	15190611	15190957	347	44.635	36	*CC2D2A*	NM_001080522.2	+	Exonic
chr4	15196772	15197060	289	22.155	34.3	*CC2D2A*	NM_001080522.2	+	Exonic
chr4	15198443	15198725	283	25.895	40.3	*CC2D2A*	NM_001080522.2	+	Exonic
chr4	15200184	15200455	272	17.7	35.7	*CC2D2A*	NM_001080522.2	+	Exonic
chr4	15206751	15206994	244	24.59	33.2	*CC2D2A*	NM_001080522.2	+	Exonic
chr4	15208006	15208293	288	35.115	33.7	*CC2D2A*	NM_001080522.2	+	Exonic
chr4	15210173	15210491	319	29.54	38.9	*CC2D2A*	NM_001080522.2	+	Exonic
chr4	15211880	15212335	456	33.195	32.5	*CC2D2A*	NM_001080522.2	+	Exonic
chr4	15578868	15578979	112	37.815	33.9	*PROM1*	NM_001145850.1	-	UTR
chr4	15579006	15579085	80	51.735	42.5	*PROM1*	NM_001145850.1	-	UTR
chr4	15579171	15580147	977	49.665	37.5	*PROM1*	NM_001145850.1	-	UTR
chr4	15581633	15581915	283	26.585	35	*PROM1*	NM_001145848.1	-	Exonic
chr4	15589999	15590289	291	26.735	35.4	*PROM1*	NM_001145848.1	-	Exonic
chr4	15590468	15590748	281	10.435	29.9	*PROM1*	NM_001145848.1	-	Exonic
chr4	15591051	15591325	275	13.26	38.9	*PROM1*	NM_001145848.1	-	Exonic
chr4	15594883	15595158	276	22.15	40.6	*PROM1*	NM_001145848.1	-	Exonic
chr4	15596404	15596654	251	25.735	34.3	*PROM1*	NM_001145848.1	-	Exonic
chr4	15596566	15596855	290	32.15	34.8	*PROM1*	NM_001145848.1	-	Exonic

chr4	15598263	15598550	288	25.95	36.8	*PROM1*	NM_001145848.1	-	Exonic
chr4	15600351	15600634	284	30.615	43	*PROM1*	NM_001145848.1	-	Exonic
chr4	15601831	15602115	285	32.775	37.9	*PROM1*	NM_001145848.1	-	Exonic
chr4	15602890	15603171	282	38.58	37.9	*PROM1*	NM_001145848.1	-	Exonic
chr4	15604639	15604882	244	47.45	41.4	*PROM1*	NM_001145848.1	-	Exonic
chr4	15609009	15609275	267	23.025	27.7	*PROM1*	NM_001145848.1	-	Exonic
chr4	15611130	15611407	278	32.9	33.5	*PROM1*	NM_001145848.1	-	Exonic
chr4	15617179	15617480	302	28.7	56	*PROM1*	NM_001145848.1	-	Exonic
chr4	15619602	15619888	287	56.655	36.9	*PROM1*	NM_001145848.1	-	Exonic
chr4	15623878	15624160	283	38.57	37.1	*PROM1*	NM_001145848.1	-	Exonic
chr4	15626773	15627055	283	32.475	34.6	*PROM1*	NM_001145848.1	-	Exonic
chr4	15628973	15629322	350	43.995	52.6	*PROM1*	NM_001145848.1	-	Exonic
chr4	15633946	15634223	278	34.69	51.1	*PROM1*	NM_001145848.1	-	Exonic
chr4	15634902	15635181	280	33.535	36.4	*PROM1*	NM_001145848.1	-	Exonic
chr4	15635823	15636100	278	43.14	42.1	*PROM1*	NM_001145848.1	-	Exonic
chr4	15643944	15644292	349	35.095	39	*PROM1*	NM_001145848.1	-	Exonic
chr4	15649549	15649829	281	12.235	22.8	*PROM1*	NM_001145848.1	-	Exonic
chr4	15686331	15686903	573	29.975	50.8	*PROM1*	NM_001145848.1	-	Exonic
chr4	15694564	15694844	281	11.645	66.2	*PROM1*	NM_001145847.1	-	UTR
chr4	47632675	47633983	1309	48.855	38.9	*CNGA1*	NM_000087.3	-	Exonic
chr4	47633990	47634638	649	52.365	36.4	*CNGA1*	NM_000087.3	-	Exonic
chr4	47637440	47637716	277	28.77	28.2	*CNGA1*	NM_000087.3	-	Exonic
chr4	47638722	47638796	75	12.96	34.7	*CNGA1*	NM_001142564.1	-	Intronic
chr4	47638798	47638994	197	15.53	35.5	*CNGA1*	NM_000087.3	-	Exonic
chr4	47639872	47639987	116	10.475	34.5	*CNGA1*	NM_000087.3	-	Exonic
chr4	47640008	47640159	152	9.43	30.9	*CNGA1*	NM_000087.3	-	Exonic
chr4	47640168	47640294	127	7.77	32.3	*CNGA1*	NM_000087.3	-	Exonic
chr4	47646487	47646765	279	26.545	33.7	*CNGA1*	NM_000087.3	-	Exonic
chr4	47648034	47648320	287	36.355	42.9	*CNGA1*	NM_000087.3	-	Exonic
chr4	47649271	47649530	260	13.705	29.6	*CNGA1*	NM_000087.3	-	Exonic
chr4	47678071	47678352	282	29.355	31.6	*CNGA1*	NM_001142564.1	-	UTR
chr4	47709577	47709714	138	13.035	42	*CNGA1*	NM_000087.3	-	UTR
chr4	47709730	47709825	96	15.535	35.4				
chr4	122965006	122965379	374	13.585	28.1	*BBS7*	NM_176824.2	-	UTR
chr4	122965389	122966636	1248	24.17	30.4	*BBS7*	NM_176824.2	-	Exonic
chr4	122968665	122968943	279	40.3	35.1	*BBS7*	NM_018190.3	-	Exonic
chr4	122968873	122969175	303	43.61	31.7	*BBS7*	NM_018190.3	-	Exonic
chr4	122969098	122969354	257	21.62	27.6	*BBS7*	NM_018190.3	-	Exonic
chr4	122973759	122974068	310	32.345	37.1	*BBS7*	NM_018190.3	-	Exonic
chr4	122975678	122975945	268	16.765	35.1	*BBS7*	NM_018190.3	-	Exonic
chr4	122980128	122980405	278	23.83	34.2	*BBS7*	NM_018190.3	-	Exonic
chr4	122984423	122984698	276	24.41	29	*BBS7*	NM_018190.3	-	Exonic
chr4	122986032	122986352	321	26.545	31.5	*BBS7*	NM_018190.3	-	Exonic
chr4	122987909	122988196	288	28.875	33.7	*BBS7*	NM_018190.3	-	Exonic
chr4	122989341	122989625	285	19.98	33	*BBS7*	NM_018190.3	-	Exonic

chr4	122993476	122993761	286	27.18	30.8	*BBS7*	NM_018190.3	-	Exonic
chr4	122995216	122995493	278	10.185	29.5	*BBS7*	NM_018190.3	-	Exonic
chr4	122995979	122996258	280	22.545	33.9	*BBS7*	NM_018190.3	-	Exonic
chr4	122999563	122999816	254	27.82	35.4	*BBS7*	NM_018190.3	-	Exonic
chr4	123002031	123002345	315	19.035	35.9	*BBS7*	NM_018190.3	-	Exonic
chr4	123003702	123003987	286	15.58	30.1	*BBS7*	NM_018190.3	-	Exonic
chr4	123008473	123008750	278	19.12	33.5	*BBS7*	NM_018190.3	-	Exonic
chr4	123010804	123011158	355	11.51	63.7	*BBS7*	NM_018190.3	-	Exonic
chr4	123873229	123873550	322	19.14	59.9	*BBS12*	NM_152618.2	+	UTR
chr4	123882411	123882772	362	42.91	33.7	*BBS12*	NM_152618.2	+	Exonic
chr4	123882780	123885601	2822	45.06	40.4	*BBS12*	NM_152618.2	+	Exonic
chr4	155884532	155884863	332	24.665	67.8	*LRAT*	NM_004744.3	+	UTR
chr4	155884850	155885521	672	31.47	59.5	*LRAT*	NM_004744.3	+	Exonic
chr4	155889505	155891251	1747	27.82	31.8	*LRAT*	NM_004744.3	+	Exonic
chr4	155891255	155892066	812	27.29	34.2	*LRAT*	NM_004744.3	+	UTR
chr4	155892365	155893700	1336	30.99	36.3	*LRAT*	NM_004744.3	+	UTR
chr4	155893704	155893769	66	11.72	18.2	*LRAT*	NM_004744.3	+	UTR
chr4	187349587	187350204	618	5	75.2	*CYP4V2*	NM_207352.3	+	Exonic
chr4	187352553	187352836	284	28.73	40.8	*CYP4V2*	NM_207352.3	+	Exonic
chr4	187354055	187354314	260	37.755	34.6	*CYP4V2*	NM_207352.3	+	Exonic
chr4	187355041	187355345	305	33.64	33.4	*CYP4V2*	NM_207352.3	+	Exonic
chr4	187355568	187355848	281	25.515	34.5	*CYP4V2*	NM_207352.3	+	Exonic
chr4	187357021	187357287	267	40.225	35.6	*CYP4V2*	NM_207352.3	+	Exonic
chr4	187359229	187359556	328	27.625	39.3	*CYP4V2*	NM_207352.3	+	Exonic
chr4	187363253	187363530	278	53.36	41.4	*CYP4V2*	NM_207352.3	+	Exonic
chr4	187366930	187367209	280	39.76	45	*CYP4V2*	NM_207352.3	+	Exonic
chr4	187367129	187367467	339	32.32	48.4	*CYP4V2*	NM_207352.3	+	Exonic
chr4	187368538	187368974	437	41.86	36.4	*CYP4V2*	NM_207352.3	+	Exonic
chr4	187369276	187369473	198	16.825	35.9	*CYP4V2*	NM_207352.3	+	UTR
chr4	187369846	187371658	1813	31.62	34.5	*CYP4V2*	NM_207352.3	+	UTR
chr5	89890282	89890570	289	6.42	65.4	*GPR98*	NM_032119.3	+	Exonic
chr5	89946327	89946648	322	23.325	28.6	*GPR98*	NM_032119.3	+	Exonic
chr5	89949296	89949581	286	26.34	34.6	*GPR98*	NM_032119.3	+	Exonic
chr5	89950626	89950813	188	18.2	27.1	*GPR98*	NM_032119.3	+	Exonic
chr5	89954074	89954302	229	22.895	43.7	*GPR98*	NM_032119.3	+	Exonic
chr5	89956611	89956896	286	32.14	40.9	*GPR98*	NM_032119.3	+	Exonic
chr5	89958724	89959382	659	52.3	35.5	*GPR98*	NM_032119.3	+	Exonic
chr5	89960064	89960470	407	23.165	41.3	*GPR98*	NM_032119.3	+	Exonic
chr5	89960717	89961175	459	24.09	35.9	*GPR98*	NM_032119.3	+	Exonic
chr5	89966631	89966926	296	23.15	34.5	*GPR98*	NM_032119.3	+	Exonic
chr5	89969231	89969586	356	18.83	35.7	*GPR98*	NM_032119.3	+	Exonic
chr5	89974123	89974404	282	30.185	38.3	*GPR98*	NM_032119.3	+	Exonic
chr5	89974321	89974680	360	25.625	36.9	*GPR98*	NM_032119.3	+	Exonic
chr5	89975299	89975615	317	22.81	32.8	*GPR98*	NM_032119.3	+	Exonic
chr5	89976203	89976500	298	22.745	32.9	*GPR98*	NM_032119.3	+	Exonic
chr5	89977457	89977725	269	11.6	30.5	*GPR98*	NM_032119.3	+	Exonic

chr5	89978992	89979395	404	33.59	39.6	*GPR98*	NM_032119.3	+	Exonic
chr5	89983093	89983359	267	23.99	30.7	*GPR98*	NM_032119.3	+	Exonic
chr5	89983838	89984177	340	24.99	33.8	*GPR98*	NM_032119.3	+	Exonic
chr5	89984716	89985576	861	45.62	42.2	*GPR98*	NM_032119.3	+	Exonic
chr5	89989408	89989907	500	28.425	38	*GPR98*	NM_032119.3	+	Exonic
chr5	90004041	90004348	308	38.335	38.3	*GPR98*	NM_032119.3	+	Exonic
chr5	90005547	90005867	321	28.165	37.4	*GPR98*	NM_032119.3	+	Exonic
chr5	90006735	90007075	341	27.38	46.9	*GPR98*	NM_032119.3	+	Exonic
chr5	90007571	90007853	283	23.92	30.7	*GPR98*	NM_032119.3	+	Exonic
chr5	90011014	90011290	277	17.985	37.5	*GPR98*	NM_032119.3	+	Exonic
chr5	90012857	90013079	223	34.585	40.8	*GPR98*	NM_032119.3	+	Exonic
chr5	90015078	90015797	720	42.08	38.9	*GPR98*	NM_032119.3	+	Exonic
chr5	90017275	90017629	355	26.055	41.1	*GPR98*	NM_032119.3	+	Exonic
chr5	90021367	90021714	348	31.18	36.5	*GPR98*	NM_032119.3	+	Exonic
chr5	90022292	90022684	393	36.645	43.8	*GPR98*	NM_032119.3	+	Exonic
chr5	90024100	90024426	327	23.575	40.4	*GPR98*	NM_032119.3	+	Exonic
chr5	90025385	90026333	949	37.5	45	*GPR98*	NM_032119.3	+	Exonic
chr5	90028430	90028779	350	25.42	37.4	*GPR98*	NM_032119.3	+	Exonic
chr5	90035152	90035435	284	38.29	33.1	*GPR98*	NM_032119.3	+	Exonic
chr5	90035866	90036084	219	23.635	34.2	*GPR98*	NM_032119.3	+	Exonic
chr5	90036894	90037218	325	41.63	40.6	*GPR98*	NM_032119.3	+	Exonic
chr5	90037724	90037983	260	16.685	36.2	*GPR98*	NM_032119.3	+	Exonic
chr5	90040287	90040571	285	15.805	33	*GPR98*	NM_032119.3	+	Exonic
chr5	90042442	90042717	276	14.185	31.9	*GPR98*	NM_032119.3	+	Exonic
chr5	90042644	90042724	81	16	30.9	*GPR98*	NM_032119.3	+	Intronic
chr5	90042736	90042959	224	14.84	36.6	*GPR98*	NM_032119.3	+	Exonic
chr5	90043792	90044061	270	19.905	32.6	*GPR98*	NM_032119.3	+	Exonic
chr5	90047959	90048339	381	39.85	37.3	*GPR98*	NM_032119.3	+	Exonic
chr5	90051540	90051852	313	31.36	36.1	*GPR98*	NM_032119.3	+	Exonic
chr5	90052422	90052701	280	20.26	33.9	*GPR98*	NM_032119.3	+	Exonic
chr5	90056329	90056624	296	14.88	28.7	*GPR98*	NM_032119.3	+	Exonic
chr5	90056549	90056850	302	16.905	25.8	*GPR98*	NM_032119.3	+	Exonic
chr5	90057025	90057269	245	31.425	33.5	*GPR98*	NM_032119.3	+	Exonic
chr5	90060164	90060568	405	20.655	40.5	*GPR98*	NM_032119.3	+	Exonic
chr5	90061130	90061408	279	22.775	33.3	*GPR98*	NM_032119.3	+	Exonic
chr5	90076538	90076905	368	30.62	39.1	*GPR98*	NM_032119.3	+	Exonic
chr5	90077088	90077430	343	38.525	33.2	*GPR98*	NM_032119.3	+	Exonic
chr5	90082044	90082333	290	33.05	35.2	*GPR98*	NM_032119.3	+	Exonic
chr5	90085081	90085460	380	23.815	36.1	*GPR98*	NM_032119.3	+	Exonic
chr5	90086476	90086815	340	40.29	36.8	*GPR98*	NM_032119.3	+	Exonic
chr5	90087986	90088271	286	20.56	42.3	*GPR98*	NM_032119.3	+	Exonic
chr5	90088471	90088802	332	20.58	40.4	*GPR98*	NM_032119.3	+	Exonic
chr5	90090966	90091220	255	29.925	36.5	*GPR98*	NM_032119.3	+	Exonic
chr5	90094842	90095098	257	33.36	40.9	*GPR98*	NM_032119.3	+	Exonic
chr5	90105667	90105931	265	27.05	36.6	*GPR98*	NM_032119.3	+	Exonic
chr5	90107942	90108214	273	28.36	39.6	*GPR98*	NM_032119.3	+	Exonic
chr5	90109398	90109676	279	20.34	41.6	*GPR98*	NM_032119.3	+	Exonic

chr5	90109919	90110234	316	12.845	40.8	*GPR98*	NM_032119.3	+	Exonic
chr5	90110367	90110715	349	15.7	43.6	*GPR98*	NM_032119.3	+	Exonic
chr5	90112972	90113215	244	39.235	34	*GPR98*	NM_032119.3	+	Exonic
chr5	90114616	90114963	348	32.845	39.7	*GPR98*	NM_032119.3	+	Exonic
chr5	90115330	90115693	364	33.265	40.7	*GPR98*	NM_032119.3	+	Exonic
chr5	90119563	90119945	383	24.99	36.3	*GPR98*	NM_032119.3	+	Exonic
chr5	90121194	90121482	289	28.54	35.3	*GPR98*	NM_032119.3	+	Exonic
chr5	90122375	90122978	604	36.805	40.9	*GPR98*	NM_032119.3	+	Exonic
chr5	90134231	90134521	291	26.72	43	*GPR98*	NM_032119.3	+	Exonic
chr5	90136796	90137097	302	37.22	43	*GPR98*	NM_032119.3	+	Exonic
chr5	90139094	90139376	283	32.315	43.1	*GPR98*	NM_032119.3	+	Exonic
chr5	90141765	90142967	1203	45.69	39.8	*GPR98*	NM_032119.3	+	Exonic
chr5	90147100	90147329	230	19.725	40	*GPR98*	NM_032119.3	+	Exonic
chr5	90154917	90155219	303	19.175	41.6	*GPR98*	NM_032119.3	+	Exonic
chr5	90160436	90160817	382	21.975	36.6	*GPR98*	NM_032119.3	+	Exonic
chr5	90172073	90172601	529	49.78	39.9	*GPR98*	NM_032119.3	+	Exonic
chr5	90180154	90180459	306	13.76	32.7	*GPR98*	NM_032119.3	+	Exonic
chr5	90184789	90185152	364	29.835	36.3	*GPR98*	NM_032119.3	+	Exonic
chr5	90185555	90185797	243	28.925	36.2	*GPR98*	NM_032119.3	+	Exonic
chr5	90187238	90187539	302	43.305	37.1	*GPR98*	NM_032119.3	+	Exonic
chr5	90195231	90195512	282	26.48	42.2	*GPR98*	NM_032119.3	+	Exonic
chr5	90296895	90297172	278	27.38	40.6	*GPR98*	NM_032119.3	+	Exonic
chr5	90316836	90317148	313	36.115	41.9	*GPR98*	NM_032119.3	+	Exonic
chr5	90403939	90404234	296	39.65	42.2	*GPR98*	NM_032119.3	+	Exonic
chr5	90433702	90433984	283	28.655	35.3	*GPR98*	NM_032119.3	+	Exonic
chr5	90481592	90481826	235	14.55	48.5	*GPR98*	NM_032119.3	+	Exonic
chr5	90484717	90485036	320	20.225	44.1	*GPR98*	NM_032119.3	+	Exonic
chr5	90495279	90495843	565	35.25	37.5	*GPR98*	NM_032119.3	+	Exonic
chr5	149217632	149217914	283	20.27	36.7	*PDE6A*	NM_000440.2	-	UTR
chr5	149217951	149218255	305	20.235	36.4	*PDE6A*	NM_000440.2	-	UTR
chr5	149218871	149219687	817	27.165	45.2	*PDE6A*	NM_000440.2	-	UTR
chr5	149219976	149220343	368	27.62	35.3	*PDE6A*	NM_000440.2	-	UTR
chr5	149220496	149220783	288	24.445	52.4	*PDE6A*	NM_000440.2	-	Exonic
chr5	149222809	149223080	272	32.605	56.6	*PDE6A*	NM_000440.2	-	Exonic
chr5	149225829	149226093	265	37.165	44.5	*PDE6A*	NM_000440.2	-	Exonic
chr5	149227370	149227652	283	46.83	52.7	*PDE6A*	NM_000440.2	-	Exonic
chr5	149227737	149228016	280	27.92	55.7	*PDE6A*	NM_000440.2	-	Exonic
chr5	149243093	149243377	285	51.28	46	*PDE6A*	NM_000440.2	-	Exonic
chr5	149244174	149244411	238	17.445	40.8	*PDE6A*	NM_000440.2	-	Exonic
chr5	149244429	149244710	282	31.385	51.4	*PDE6A*	NM_000440.2	-	Exonic
chr5	149245926	149246207	282	18.42	54.6	*PDE6A*	NM_000440.2	-	Exonic
chr5	149254843	149255132	290	21.655	53.4	*PDE6A*	NM_000440.2	-	Exonic
chr5	149256061	149256318	258	34.78	38	*PDE6A*	NM_000440.2	-	Exonic
chr5	149256353	149256630	278	33.645	51.4	*PDE6A*	NM_000440.2	-	Exonic
chr5	149258042	149258272	231	40.125	35.5	*PDE6A*	NM_000440.2	-	Exonic
chr5	149259050	149259333	284	39.965	46.8	*PDE6A*	NM_000440.2	-	Exonic

chr5	149263277	149263552	276	16.2	41.7	*PDE6A*	NM_000440.2	-	Exonic
chr5	149266954	149267240	287	27.565	47.7	*PDE6A*	NM_000440.2	-	Exonic
chr5	149274615	149274795	181	11.755	35.4	*PDE6A*	NM_000440.2	-	Exonic
chr5	149281279	149281552	274	34.765	39.1	*PDE6A*	NM_000440.2	-	Exonic
chr5	149290704	149290982	279	40.275	43	*PDE6A*	NM_000440.2	-	Exonic
chr5	149293585	149293862	278	33.35	46.4	*PDE6A*	NM_000440.2	-	Exonic
chr5	149294243	149294539	297	37.245	46.1	*PDE6A*	NM_000440.2	-	Exonic
chr5	149303877	149304616	740	32.825	53.4	*PDE6A*	NM_000440.2	-	Exonic
chr5	178337858	178338620	763	40.365	37.4	*GRM6*	NM_000843.3	-	UTR
chr5	178338850	178340248	1399	34.175	45.2	*GRM6*	NM_000843.3	-	UTR
chr5	178340530	178341495	966	26	58.3	*GRM6*	NM_000843.3	-	Exonic
chr5	178342436	178342576	141	20.185	51.8	*GRM6*	NM_000843.3	-	Exonic
chr5	178342588	178342893	306	25.57	61.4	*GRM6*	NM_000843.3	-	Exonic
chr5	178345660	178346404	745	20.36	66.8	*GRM6*	NM_000843.3	-	Exonic
chr5	178346331	178346645	315	20.82	65.7	*GRM6*	NM_000843.3	-	Exonic
chr5	178348464	178348793	330	17.015	63.3	*GRM6*	NM_000843.3	-	Exonic
chr5	178348762	178349075	314	10.915	59.9	*GRM6*	NM_000843.3	-	Exonic
chr5	178350120	178350410	291	14.49	66.7	*GRM6*	NM_000843.3	-	Exonic
chr5	178350948	178351231	284	15.74	61.3	*GRM6*	NM_000843.3	-	Exonic
chr5	178351394	178351754	361	15.395	60.9	*GRM6*	NM_000843.3	-	Exonic
chr5	178353967	178354789	823	5.24	76.4	*GRM6*	NM_000843.3	-	Exonic
chr6	35573553	35574278	726	11.98	67.1	*TULP1*	NM_003322.3	-	Exonic
chr6	35575660	35575974	315	19.425	58.7	*TULP1*	NM_003322.3	-	Exonic
chr6	35579213	35579674	462	14.025	61.7	*TULP1*	NM_003322.3	-	Exonic
chr6	35581401	35581686	286	11.045	58	*TULP1*	NM_003322.3	-	Exonic
chr6	35581692	35582168	477	10.92	66.7	*TULP1*	NM_003322.3	-	Exonic
chr6	35584867	35585143	277	13.05	58.5	*TULP1*	NM_003322.3	-	Exonic
chr6	35585297	35585758	462	13.84	58.2	*TULP1*	NM_003322.3	-	Exonic
chr6	35586535	35586713	179	14.06	59.2	*TULP1*	NM_003322.3	-	Exonic
chr6	35586755	35586825	71	6.89	57.7	*TULP1*	NM_003322.3	-	Exonic
chr6	35587322	35587621	300	6.925	72.7	*TULP1*	NM_003322.3	-	Exonic
chr6	35587832	35588107	276	4.425	72.5	*TULP1*	NM_003322.3	-	Exonic
chr6	35588273	35588730	458	9.965	62.9	*TULP1*	NM_003322.3	-	Exonic
chr6	42231043	42231375	333	18.45	55.6	*GUCA1A*	NM_000409.3	+	UTR
chr6	42238555	42238863	309	17.025	58.6	*GUCA1A*	NM_000409.3	+	UTR
chr6	42248968	42249591	624	25.69	55.9	*GUCA1A*	NM_000409.3	+	Exonic
chr6	42253915	42254209	295	15.97	64.4	*GUCA1A*	NM_000409.3	+	Exonic
chr6	42254414	42254688	275	17.095	60	*GUCA1A*	NM_000409.3	+	Exonic
chr6	42254888	42255840	953	26.83	53.7	*GUCA1A*	NM_000409.3	+	Exonic
chr6	42258933	42259809	877	17.795	45.3	*GUCA1B*	NM_002098.5	-	UTR
chr6	42260094	42260722	629	17.275	58.3	*GUCA1B*	NM_002098.5	-	Exonic
chr6	42261304	42261587	284	41.21	57.7	*GUCA1B*	NM_002098.5	-	Exonic
chr6	42264222	42264500	279	37.48	56.3	*GUCA1B*	NM_002098.5	-	Exonic
chr6	42270249	42270731	483	25.65	58.4	*GUCA1B*	NM_002098.5	-	Exonic
chr6	42772235	42773530	1296	27.51	47.5	*PRPH2*	NM_000322.4	-	UTR
chr6	42773535	42774285	751	19.345	54.2	*PRPH2*	NM_000322.4	-	Exonic

chr6	42780000	42780391	392	16.865	59.7	*PRPH2*	NM_000322.4	-	Exonic
chr6	42797394	42798378	985	30.98	54.4	*PRPH2*	NM_000322.4	-	Exonic
chr6	64487759	64489707	1949	39.015	32.7	*EYS*	NM_001142800.1	-	Exonic
chr6	64494294	64494597	304	40.47	37.8	*EYS*	NM_001142800.1	-	Exonic
chr6	64530233	64530544	312	26.78	42.6	*EYS*	NM_001142800.1	-	Exonic
chr6	64545782	64546085	304	35.495	43.1	*EYS*	NM_001142800.1	-	Exonic
chr6	64555891	64556157	267	17.47	33.7	*EYS*	NM_001142800.1	-	Exonic
chr6	64556834	64557140	307	26.305	49.2	*EYS*	NM_001142800.1	-	Exonic
chr6	64573964	64574283	320	28.52	39.7	*EYS*	NM_001142800.1	-	Exonic
chr6	64631957	64632232	276	30.475	42.8	*EYS*	NM_001142800.1	-	Exonic
chr6	64752159	64752510	352	37.505	39.5	*EYS*	NM_001142800.1	-	Exonic
chr6	64766835	64767116	282	37.81	38.7	*EYS*	NM_001142800.1	-	Exonic
chr6	64834128	64834390	263	15.365	31.6	*EYS*	NM_001142800.1	-	Exonic
chr6	64849631	64849907	277	30.98	30.3	*EYS*	NM_001142800.1	-	Exonic
chr6	64998376	64998725	350	22.72	44	*EYS*	NM_001142800.1	-	Exonic
chr6	65074730	65074948	219	26.87	33.8	*EYS*	NM_001142800.1	-	Exonic
chr6	65155296	65155522	227	15.455	30.4	*EYS*	NM_001142800.1	-	Exonic
chr6	65202685	65202965	281	19.02	31.7	*EYS*	NM_001142800.1	-	Exonic
chr6	65205745	65206021	277	27.39	29.6	*EYS*	NM_001142800.1	-	Exonic
chr6	65356757	65358651	1895	40.035	39.2	*EYS*	NM_001142800.1	-	Exonic
chr6	65359655	65359974	320	20.92	37.2	*EYS*	NM_001142800.1	-	Exonic
chr6	65383984	65384226	243	36.135	35.8	*EYS*	NM_001142800.1	-	Exonic
chr6	65392716	65392930	215	19.255	31.2	*EYS*	NM_001142800.1	-	Exonic
chr6	65579912	65580254	343	16.005	36.7	*EYS*	NM_001142800.1	-	Exonic
chr6	65588147	65588421	275	28.835	28.7	*EYS*	NM_001142800.1	-	Exonic
chr6	65589184	65589498	315	34.16	34.9	*EYS*	NM_001142800.1	-	Exonic
chr6	65653232	65653514	283	14.63	36.4	*EYS*	NM_001142800.1	-	Exonic
chr6	65668643	65668916	274	27.065	31.4	*EYS*	NM_001142800.1	-	Exonic
chr6	65668920	65669195	276	29.465	32.2	*EYS*	NM_001142800.1	-	Exonic
chr6	65679017	65679399	383	25.105	36.3	*EYS*	NM_001142800.1	-	Exonic
chr6	65712321	65712590	270	33.47	34.1	*EYS*	NM_001142800.1	-	Exonic
chr6	65764107	65764381	275	20.795	37.8	*EYS*	NM_001142800.1	-	Exonic
chr6	65824134	65824417	284	32.93	37	*EYS*	NM_001142800.1	-	Exonic
chr6	66062397	66062799	403	25.27	34.2	*EYS*	NM_001142800.1	-	Exonic
chr6	66101523	66101819	297	33.465	32.3	*EYS*	NM_198283.1	-	Exonic
chr6	66110575	66110810	236	32.77	35.2	*EYS*	NM_198283.1	-	Exonic
chr6	66119991	66120293	303	27.14	32.7	*EYS*	NM_198283.1	-	Exonic
chr6	66150906	66151188	283	21.09	29.3	*EYS*	NM_198283.1	-	Exonic
chr6	66169008	66169248	241	17.805	27.4	*EYS*	NM_198283.1	-	Exonic
chr6	66171747	66172010	264	23.565	34.8	*EYS*	NM_198283.1	-	Exonic
chr6	66257119	66257252	134	13.82	23.1	*EYS*	NM_198283.1	-	Exonic
chr6	66257304	66257387	84	10.945	26.2	*EYS*	NM_198283.1	-	Exonic
chr6	66261246	66262273	1028	50.13	36.5	*EYS*	NM_198283.1	-	Exonic
chr6	66262390	66262663	274	26.705	32.5	*EYS*	NM_001142800.1	-	UTR
chr6	66406300	66406530	231	29.715	39	*EYS*	NM_001142800.1	-	UTR
chr6	66473643	66473927	285	25.395	36.5	*EYS*	NM_001142800.1	-	UTR
chr6	72653299	72653676	378	8.885	66.4	*RIMS1*	NM_014989.4	+	Exonic

chr6	72735296	72735579	284	38.03	42.3	*RIMS1*	NM_014989.4	+	Exonic
chr6	72863297	72863648	352	30.395	44.6	*RIMS1*	NM_014989.4	+	Exonic
chr6	72866256	72866538	283	16.82	29	*RIMS1*	NM_014989.4	+	Exonic
chr6	72945923	72946403	481	23.785	47	*RIMS1*	NM_014989.4	+	Exonic
chr6	72948642	72949638	997	9	64.8	*RIMS1*	NM_014989.4	+	Exonic
chr6	73000081	73000367	287	27.765	37.3	*RIMS1*	NM_001168410.1	+	Exonic
chr6	73001946	73002232	287	16.52	37.3	*RIMS1*	NM_001168410.1	+	Exonic
chr6	73004150	73004431	282	16.85	34	*RIMS1*	NM_001168410.1	+	Exonic
chr6	73008667	73008916	250	13.01	30	*RIMS1*	NM_001168410.1	+	Exonic
chr6	73012111	73012392	282	26.24	36.5	*RIMS1*	NM_001168410.1	+	Exonic
chr6	73014345	73014629	285	19.475	33.3	*RIMS1*	NM_001168410.1	+	Exonic
chr6	73016673	73016905	233	19.41	33.5	*RIMS1*	NM_001168410.1	+	Exonic
chr6	73017265	73017578	314	29.84	30.9	*RIMS1*	NM_001168410.1	+	Exonic
chr6	73017529	73017853	325	36.37	34.2	*RIMS1*	NM_001168410.1	+	Exonic
chr6	73019100	73019356	257	27.23	42.8	*RIMS1*	NM_001168410.1	+	Exonic
chr6	73024473	73024765	293	42.79	39.2	*RIMS1*	NM_001168410.1	+	Exonic
chr6	73025322	73025595	274	37.69	35	*RIMS1*	NM_001168410.1	+	Exonic
chr6	73027011	73027284	274	38.3	42	*RIMS1*	NM_001168410.1	+	Exonic
chr6	73031287	73031544	258	19.455	35.7	*RIMS1*	NM_014989.4	+	Exonic
chr6	73031780	73032001	222	23.975	32.9	*RIMS1*	NM_014989.4	+	Exonic
chr6	73032279	73032562	284	30.37	38.7	*RIMS1*	NM_001168410.1	+	Exonic
chr6	73040668	73040937	270	26.88	45.9	*RIMS1*	NM_014989.4	+	Exonic
chr6	73050360	73050643	284	28.98	35.6	*RIMS1*	NM_001168410.1	+	Exonic
chr6	73057023	73057340	318	34.92	50.3	*RIMS1*	NM_014989.4	+	Exonic
chr6	73058267	73058534	268	32.415	44	*RIMS1*	NM_001168410.1	+	Exonic
chr6	73073592	73073871	280	31.015	37.5	*RIMS1*	NM_001168407.1	+	Exonic
chr6	73079850	73080153	304	28.43	39.8	*RIMS1*	NM_001168407.1	+	Exonic
chr6	73099943	73100317	375	24.75	42.1	*RIMS1*	NM_001168407.1	+	Exonic
chr6	73156943	73157224	282	28.12	43.6	*RIMS1*	NM_001168411.1	+	Exonic
chr6	73159029	73159295	267	20.255	37.8	*RIMS1*	NM_001168411.1	+	Exonic
chr6	73164573	73164844	272	35.335	39	*RIMS1*	NM_001168411.1	+	Exonic
chr6	73165297	73165580	284	13.8	33.1	*RIMS1*	NM_001168411.1	+	Exonic
chr6	73166838	73167402	565	54.64	42.5	*RIMS1*	NM_001168411.1	+	Exonic
chr6	73167438	73167617	180	46.965	35	*RIMS1*	NM_014989.4	+	UTR
chr6	73167633	73168581	949	32.74	35.8	*RIMS1*	NM_014989.4	+	UTR
chr6	73168598	73168961	364	36.24	36.5	*RIMS1*	NM_014989.4	+	UTR
chr6	73168962	73169277	316	25.53	37.7	*RIMS1*	NM_014989.4	+	UTR
chr6	80251351	80253060	1710	33.12	33.1	*LCA5*	NM_181714.3	-	UTR
chr6	80253110	80254363	1254	28.345	34.4	*LCA5*	NM_001122769.2	-	Exonic
chr6	80255438	80255712	275	30.235	37.8	*LCA5*	NM_001122769.2	-	Exonic
chr6	80257948	80258188	241	24.48	35.3	*LCA5*	NM_001122769.2	-	Exonic
chr6	80258887	80259135	249	15.035	30.5	*LCA5*	NM_001122769.2	-	Exonic
chr6	80259987	80260249	263	24.785	30.4	*LCA5*	NM_001122769.2	-	Exonic
chr6	80279570	80280210	641	46.63	38.5	*LCA5*	NM_001122769.2	-	Exonic
chr6	80285062	80285572	511	36.58	38.2	*LCA5*	NM_001122769.2	-	Exonic
chr6	80291507	80291783	277	17.195	33.6	*LCA5*	NM_181714.3	-	UTR
chr6	80303481	80303936	456	13.5	64.9	*LCA5*	NM_181714.3	-	UTR

chr6	80681197	80682081	885	35.095	29.8	*ELOVL4*	NM_181714.3	-	UTR
chr6	80682087	80682294	208	29.765	27.4	*ELOVL4*	NM_022726.3	-	UTR
chr6	80682307	80682838	532	32.775	35.3	*ELOVL4*	NM_022726.3	-	UTR
chr6	80682842	80683381	540	26.195	36.7	*ELOVL4*	NM_022726.3	-	Exonic
chr6	80685774	80686054	281	29.52	33.1	*ELOVL4*	NM_022726.3	-	Exonic
chr6	80687981	80688299	319	38.365	37.6	*ELOVL4*	NM_022726.3	-	Exonic
chr6	80691296	80691559	264	21.025	28	*ELOVL4*	NM_022726.3	-	Exonic
chr6	80692552	80692879	328	31.68	34.1	*ELOVL4*	NM_022726.3	-	Exonic
chr6	80713538	80714081	544	12.34	72.4	*ELOVL4*	NM_022726.3	-	Exonic
chr6	135646728	135647724	997	20.745	30.6	*AHI1*	NM_017651.4	-	UTR
chr6	135647979	135648538	560	30.035	33.2	*AHI1*	NM_001134831.1	-	Exonic
chr6	135653154	135653433	280	31.775	42.1	*AHI1*	NM_001134831.1	-	Exonic
chr6	135663224	135663496	273	20.405	33.3	*AHI1*	NM_001134831.1	-	Exonic
chr6	135681248	135681530	283	20.52	36	*AHI1*	NM_001134831.1	-	Exonic
chr6	135685917	135686209	293	49.59	39.9	*AHI1*	NM_001134831.1	-	Exonic
chr6	135720842	135721109	268	39.83	33.6	*AHI1*	NM_001134831.1	-	Exonic
chr6	135757516	135757797	282	24.23	36.5	*AHI1*	NM_001134832.1	-	Exonic
chr6	135767644	135767930	287	38.37	32.1	*AHI1*	NM_001134832.1	-	Exonic
chr6	135774103	135774440	338	34.195	36.7	*AHI1*	NM_001134832.1	-	Exonic
chr6	135789934	135790203	270	38.635	33.7	*AHI1*	NM_001134832.1	-	Exonic
chr6	135791374	135791657	284	40.36	35.2	*AHI1*	NM_001134832.1	-	Exonic
chr6	135792625	135792892	268	17.54	29.5	*AHI1*	NM_001134832.1	-	Exonic
chr6	135793941	135794227	287	22.16	31.7	*AHI1*	NM_001134832.1	-	Exonic
chr6	135795777	135796142	366	45.735	34.2	*AHI1*	NM_001134832.1	-	Exonic
chr6	135801121	135801398	278	21.07	34.2	*AHI1*	NM_001134832.1	-	Exonic
chr6	135805332	135805602	271	33.515	36.9	*AHI1*	NM_001134832.1	-	Exonic
chr6	135809758	135810054	297	25.705	36	*AHI1*	NM_001134832.1	-	Exonic
chr6	135811045	135811361	317	24.77	34.4	*AHI1*	NM_001134832.1	-	Exonic
chr6	135816071	135816344	274	30.995	29.9	*AHI1*	NM_001134832.1	-	Exonic
chr6	135818489	135818812	324	22.35	31.5	*AHI1*	NM_001134832.1	-	Exonic
chr6	135820244	135820598	355	42.295	34.4	*AHI1*	NM_001134832.1	-	Exonic
chr6	135825875	135825972	98	24.115	28.6	*AHI1*	NM_001134832.1	-	Exonic
chr6	135825975	135826204	230	25.17	32.2	*AHI1*	NM_001134832.1	-	Exonic
chr6	135828567	135828683	117	29.88	29.9	*AHI1*	NM_001134832.1	-	Exonic
chr6	135828684	135829122	439	48.845	39.2	*AHI1*	NM_001134832.1	-	Exonic
chr6	135829133	135829268	136	23.915	33.1	*AHI1*	NM_001134832.1	-	Exonic
chr6	135830288	135830383	96	30.14	33.3	*AHI1*	NM_017651.4	-	Intronic
chr6	135830394	135830572	179	37.025	30.2	*AHI1*	NM_001134832.1	-	Exonic
chr6	135853380	135853649	270	35.98	34.4	*AHI1*	NM_001134832.1	-	Exonic
chr6	135854941	135855183	243	18.7	29.6	*AHI1*	NM_001134832.1	-	Exonic
chr6	135859910	135860179	270	32.705	32.6	*AHI1*	NM_017651.4	-	UTR
chr6	135860338	135860658	321	18.015	72.3	*AHI1*	NM_017651.4	-	UTR
chr7	23111802	23112352	551	13.615	66.1	*KLHL7*	NM_001031710.2	+	Exonic
chr7	23123897	23124177	281	38.765	38.4	*KLHL7*	NM_018846.4	+	Exonic
chr7	23129824	23130107	284	29.025	33.5	*KLHL7*	NM_001031710.2	+	Exonic

chr7	23130733	23131011	279	38.19	35.5	*KLHL7*	NM_001031710.2	+	Exonic
chr7	23131107	23131372	266	32.24	32	*KLHL7*	NM_001031710.2	+	Exonic
chr7	23146882	23147147	266	42.195	35.3	*KLHL7*	NM_001031710.2	+	Exonic
chr7	23149914	23150230	317	38.645	37.2	*KLHL7*	NM_001031710.2	+	Exonic
chr7	23158131	23158418	288	33.935	39.6	*KLHL7*	NM_001031710.2	+	Exonic
chr7	23171765	23172143	379	20.355	42.5	*KLHL7*	NM_001031710.2	+	Exonic
chr7	23173901	23174243	343	21.255	41.1	*KLHL7*	NM_001031710.2	+	Exonic
chr7	23178995	23179271	277	21.175	33.9	*KLHL7*	NM_001031710.2	+	Exonic
chr7	23180080	23181581	1502	35.93	33.4	*KLHL7*	NM_001031710.2	+	Exonic
chr7	33100854	33100941	88	9.89	15.9	*RP9*	NM_203288.1	-	UTR
chr7	33100956	33101046	91	15.275	51.6	*RP9*	NM_203288.1	-	UTR
chr7	33102510	33102785	276	28.81	35.5	*RP9*	NM_203288.1	-	Exonic
chr7	33103308	33103586	279	33.255	36.2	*RP9*	NM_203288.1	-	Exonic
chr7	33105362	33105442	81	25.665	35.8	*RP9*	NM_203288.1	-	Splice site_CANONICAL
chr7	33105527	33105639	113	24.835	47.8	*RP9*	NM_203288.1	-	Exonic
chr7	33106532	33106675	144	36.215	34	*RP9*	NM_203288.1	-	Exonic
chr7	33106706	33106813	108	31.215	29.6	*RP9*	NM_203288.1	-	Splice site
chr7	33115277	33115562	109	3.81	77.6	*RP9*	NM_203288.1	-	Exonic
chr7	33135596	33135909	314	15.68	61.5	*BBS9*	NM_198428.2	+	UTR
chr7	33135948	33136234	287	15.535	51.9	*BBS9*	NM_198428.2	+	UTR
chr7	33152289	33152575	287	25.355	35.2	*BBS9*	NM_014451.3	+	Exonic
chr7	33158757	33159051	295	20.895	36.9	*BBS9*	NM_014451.3	+	Exonic
chr7	33161661	33161944	284	21.665	31.3	*BBS9*	NM_014451.3	+	Exonic
chr7	33183526	33183803	278	31.24	32.4	*BBS9*	NM_014451.3	+	Exonic
chr7	33263292	33263608	317	12.905	36	*BBS9*	NM_014451.3	+	Exonic
chr7	33270318	33270380	63	6.195	14.3	*BBS9*	NM_014451.3	+	Intronic
chr7	33270403	33270603	201	9.11	29.4	*BBS9*	NM_014451.3	+	Exonic
chr7	33279070	33279397	328	31.78	34.5	*BBS9*	NM_014451.3	+	Exonic
chr7	33279878	33280157	280	26.325	33.9	*BBS9*	NM_014451.3	+	Exonic
chr7	33342497	33342826	330	26.885	32.7	*BBS9*	NM_014451.3	+	Exonic
chr7	33346921	33347204	284	13.675	32.4	*BBS9*	NM_014451.3	+	Exonic
chr7	33350594	33350871	278	25.085	35.6	*BBS9*	NM_014451.3	+	Exonic
chr7	33355108	33355361	254	30.72	30.3	*BBS9*	NM_014451.3	+	Exonic
chr7	33357257	33357544	288	26.795	31.9	*BBS9*	NM_001033605.1	+	Exonic
chr7	33358852	33359140	289	23.175	31.1	*BBS9*	NM_001033605.1	+	Exonic
chr7	33363926	33364201	276	33.685	35.1	*BBS9*	NM_014451.3	+	Exonic
chr7	33373803	33374036	234	35.615	35	*BBS9*	NM_014451.3	+	Exonic
chr7	33389722	33390037	316	38.03	32.6	*BBS9*	NM_014451.3	+	Exonic
chr7	33394052	33394338	287	28.065	35.9	*BBS9*	NM_014451.3	+	Exonic
chr7	33511523	33511845	323	32.72	46.1	*BBS9*	NM_014451.3	+	Exonic
chr7	33540023	33540380	358	26.725	46.4	*BBS9*	NM_014451.3	+	Exonic
chr7	33610906	33611188	283	51.535	41	*BBS9*	NM_014451.3	+	Exonic
chr7	33611301	33612255	955	22.56	35	*BBS9*	NM_014451.3	+	Exonic
chr7	127819491	127819614	124	4.91	53.2	*IMPDH1*	NM_000883.3	-	UTR

chr7	127819710	127819789	307	3.075	66.2	*IMPDH1*	NM_000883.3	-	UTR
chr7	127819896	127819973	178	3.525	66.7	*IMPDH1*	NM_000883.3	-	UTR
chr7	127820089	127820181	335	4.89	67.7	*IMPDH1*	NM_000883.3	-	UTR
chr7	127820270	127820378	87	3.465	60.6	*IMPDH1*	NM_001142576.1	-	Exonic
chr7	127821463	127821599	137	17.145	61.3	*IMPDH1*	NM_001142576.1	-	Exonic
chr7	127821619	127821741	123	18.145	56.1	*IMPDH1*	NM_001142576.1	-	Exonic
chr7	127821646	127821944	299	14.555	59.9	*IMPDH1*	NM_001142576.1	-	Exonic
chr7	127822099	127822200	102	13.865	62.7	*IMPDH1*	NM_001142576.1	-	Exonic
chr7	127822228	127822370	143	15.525	63.6	*IMPDH1*	NM_001142576.1	-	Exonic
chr7	127822305	127822467	163	13.45	64.4	*IMPDH1*	NM_001142576.1	-	Exonic
chr7	127822529	127822628	100	8.035	67	*IMPDH1*	NM_001142576.1	-	Exonic
chr7	127823788	127823926	139	8.375	62.6	*IMPDH1*	NM_001142576.1	-	Exonic
chr7	127823951	127824073	123	9.805	61.8	*IMPDH1*	NM_001142576.1	-	Exonic
chr7	127824117	127824256	140	9.495	61.4	*IMPDH1*	NM_001142576.1	-	Exonic
chr7	127824276	127824401	126	7.395	54	*IMPDH1*	NM_001142576.1	-	Exonic
chr7	127825624	127825738	115	17.475	60	*IMPDH1*	NM_001142576.1	-	Exonic
chr7	127825750	127825837	88	15.075	58	*IMPDH1*	NM_001142576.1	-	Exonic
chr7	127825867	127825969	103	10.58	50.5	*IMPDH1*	NM_001142576.1	-	Exonic
chr7	127827278	127827420	143	13.88	53.1	*IMPDH1*	NM_001142576.1	-	Exonic
chr7	127827437	127827564	128	18.08	60.2	*IMPDH1*	NM_001142576.1	-	Exonic
chr7	127827507	127827657	151	15.39	63.6	*IMPDH1*	NM_001142576.1	-	Exonic
chr7	127827795	127827897	103	6.83	58.3	*IMPDH1*	NM_001142576.1	-	Exonic
chr7	127827993	127828139	147	5.57	64.6	*IMPDH1*	NM_001142576.1	-	Exonic
chr7	127828148	127828282	135	11.36	57.8	*IMPDH1*	NM_001142576.1	-	Exonic
chr7	127828177	127828349	173	14.68	59.5	*IMPDH1*	NM_001142576.1	-	Exonic
chr7	127828372	127828492	121	15.305	57.9	*IMPDH1*	NM_001142576.1	-	Exonic
chr7	127830870	127831154	285	9.755	53.7	*IMPDH1*	NM_001142576.1	-	Exonic
chr7	127832956	127833228	273	4.77	74.7	*IMPDH1*	NM_001142576.1	-	Exonic
chr7	127836970	127837319	350	7.875	80	*IMPDH1*	NM_001142576.1	-	Exonic
chr8	38973581	38973887	213	5.36	71.3	*ADAM9*	NM_003816.2	+	Exonic
chr8	38984460	38984705	246	20.21	30.5	*ADAM9*	NM_003816.2	+	Exonic
chr8	38988224	38988495	272	26.025	30.5	*ADAM9*	NM_003816.2	+	Exonic
chr8	38990534	38990817	284	16.51	33.8	*ADAM9*	NM_003816.2	+	Exonic
chr8	38992684	38992965	282	20.775	34	*ADAM9*	NM_003816.2	+	Exonic
chr8	38993844	38994152	309	40.315	37.2	*ADAM9*	NM_003816.2	+	Exonic
chr8	38995416	38995697	282	25.135	34.8	*ADAM9*	NM_003816.2	+	Exonic
chr8	38998234	38998488	255	28.17	38	*ADAM9*	NM_003816.2	+	Exonic
chr8	38999751	39000017	267	7.955	38.2	*ADAM9*	NM_003816.2	+	Exonic
chr8	39002369	39002643	275	12.89	38.9	*ADAM9*	NM_003816.2	+	Exonic
chr8	39003274	39003544	271	44.235	39.1	*ADAM9*	NM_003816.2	+	Exonic
chr8	39018541	39018851	311	21.665	37.3	*ADAM9*	NM_003816.2	+	Exonic
chr8	39031054	39031286	233	19.96	34.8	*ADAM9*	NM_003816.2	+	Exonic
chr8	39032177	39032515	339	33.945	34.2	*ADAM9*	NM_003816.2	+	Exonic
chr8	39047876	39048146	271	11.42	35.4	*ADAM9*	NM_003816.2	+	Exonic
chr8	39053823	39054119	297	19.055	36	*ADAM9*	NM_003816.2	+	Exonic

chr8	39059226	39059300	75	16.73	33.3	*ADAM9*	NR_027638.1	+	UTR
chr8	39059306	39059486	181	25.24	37	*ADAM9*	NM_003816.2	+	Exonic
chr8	39059550	39059830	281	33.135	39.9	*ADAM9*	NM_003816.2	+	Exonic
chr8	39066645	39066884	240	23.505	35	*ADAM9*	NM_003816.2	+	Exonic
chr8	39067829	39068114	286	18.81	32.2	*ADAM9*	NM_003816.2	+	Exonic
chr8	39078427	39078700	274	15.75	30.7	*ADAM9*	NM_003816.2	+	Exonic
chr8	39080202	39080428	227	32.52	41	*ADAM9*	NM_003816.2	+	Exonic
chr8	39080432	39081624	1193	30.725	28.8	*ADAM9*	NR_027638.1	+	UTR
chr8	39081637	39081991	355	16.82	25.6	*ADAM9*	NR_027638.1	+	UTR
chr8	55691097	55691374	278	37.695	33.8	*RP1*	NM_006269.1	+	UTR
chr8	55695987	55696735	749	28.03	57.7	*RP1*	NM_006269.1	+	Exonic
chr8	55697149	55697464	316	25.695	41.8	*RP1*	NM_006269.1	+	Exonic
chr8	55699702	55705636	5935	48.91	36.8	*RP1*	NM_006269.1	+	Exonic
chr8	55705637	55705715	79	27.73	30.4	*RP1*	NM_006269.1	+	UTR
chr8	55705722	55706006	285	22.52	30.2	*RP1*	NM_006269.1	+	UTR
chr8	87655201	87657391	2191	37.555	36	*CNGB3*	NM_019098.4	-	Exonic
chr8	87657446	87657516	71	13.915	38	*CNGB3*	NM_019098.4	-	Exonic
chr8	87659956	87660273	318	20.375	45.6	*CNGB3*	NM_019098.4	-	Exonic
chr8	87660370	87660661	292	25.895	41.8	*CNGB3*	NM_019098.4	-	Exonic
chr8	87685349	87685623	275	26.96	34.5	*CNGB3*	NM_019098.4	-	Exonic
chr8	87692823	87693099	277	25.99	31.4	*CNGB3*	NM_019098.4	-	Exonic
chr8	87707230	87707503	274	26.455	35.4	*CNGB3*	NM_019098.4	-	Exonic
chr8	87710185	87710486	302	51.055	41.1	*CNGB3*	NM_019098.4	-	Exonic
chr8	87714017	87714303	287	40.45	34.5	*CNGB3*	NM_019098.4	-	Exonic
chr8	87725005	87725289	285	49.57	33.7	*CNGB3*	NM_019098.4	-	Exonic
chr8	87725851	87726081	231	8.94	32.9	*CNGB3*	NM_019098.4	-	Exonic
chr8	87729037	87729322	286	26.06	30.4	*CNGB3*	NM_019098.4	-	Exonic
chr8	87735231	87735504	274	26.195	29.9	*CNGB3*	NM_019098.4	-	Exonic
chr8	87748189	87748535	347	30.23	40.9	*CNGB3*	NM_019098.4	-	Exonic
chr8	87749286	87749531	246	27.67	39.8	*CNGB3*	NM_019098.4	-	Exonic
chr8	87752209	87752502	294	20.59	45.2	*CNGB3*	NM_019098.4	-	Exonic
chr8	87807790	87808066	277	20.41	41.5	*CNGB3*	NM_019098.4	-	Exonic
chr8	87820890	87820971	82	17.22	28	*CNGB3*	NM_019098.4	-	Intronic
chr8	87820974	87821162	189	17.86	34.4	*CNGB3*	NM_019098.4	-	Exonic
chr8	87824762	87825080	319	35.735	38.6	*CNGB3*	NM_019098.4	-	Exonic
chr8	94836182	94836596	415	24.48	60.5	*TMEM67*	NM_153704.5	+	Exonic
chr8	94837170	94837347	301	10.68	27	*TMEM67*	NM_153704.5	+	Exonic
chr8	94839783	94840039	257	15.61	30.7	*TMEM67*	NM_153704.5	+	Exonic
chr8	94845149	94845418	270	19.66	31.1	*TMEM67*	NM_153704.5	+	Exonic
chr8	94846694	94847144	451	16.885	31.7	*TMEM67*	NM_153704.5	+	Exonic
chr8	94853878	94854149	272	23.585	27.2	*TMEM67*	NM_153704.5	+	Exonic
chr8	94861921	94862213	293	35.265	31.7	*TMEM67*	NM_153704.5	+	Exonic
chr8	94862148	94862465	318	24.185	28.9	*TMEM67*	NM_153704.5	+	Exonic
chr8	94862957	94863228	272	30.01	30.1	*TMEM67*	NM_153704.5	+	Exonic
chr8	94863695	94863965	271	18.465	29.9	*TMEM67*	NM_153704.5	+	Exonic
chr8	94866605	94866838	234	32.07	30.8	*TMEM67*	NM_153704.5	+	Exonic

chr8	94867539	94867824	286	29.77	33.6	*TMEM67*	NM_153704.5	+	Exonic
chr8	94869150	94869434	285	27.305	34	*TMEM67*	NM_153704.5	+	Exonic
chr8	94872544	94872827	284	15.385	26.4	*TMEM67*	NM_153704.5	+	Exonic
chr8	94874515	94874783	269	14.83	36.8	*TMEM67*	NM_153704.5	+	Exonic
chr8	94876716	94876992	277	27.085	33.6	*TMEM67*	NM_153704.5	+	Exonic
chr8	94877252	94877472	221	22.925	32.6	*TMEM67*	NM_153704.5	+	Exonic
chr8	94878441	94878718	278	35.74	32.7	*TMEM67*	NM_153704.5	+	Exonic
chr8	94878621	94878936	316	27.545	33.2	*TMEM67*	NM_153704.5	+	Exonic
chr8	94880944	94881221	278	22.785	36.7	*TMEM67*	NM_153704.5	+	Exonic
chr8	94884897	94885174	278	32.25	32.4	*TMEM67*	NM_153704.5	+	Exonic
chr8	94886076	94886351	276	20.65	29.3	*TMEM67*	NM_153704.5	+	Exonic
chr8	94890151	94890422	272	29.87	30.5	*TMEM67*	NM_153704.5	+	Exonic
chr8	94890331	94890635	305	29.89	30.2	*TMEM67*	NM_153704.5	+	Exonic
chr8	94891094	94891365	272	20.02	29	*TMEM67*	NM_153704.5	+	Exonic
chr8	94896628	94896888	261	23.11	28.7	*TMEM67*	NM_153704.5	+	Exonic
chr8	94897695	94898673	979	20.98	27.6	*TMEM67*	NM_153704.5	+	Exonic
chr8	94898935	94899558	624	24.975	29.8	*TMEM67*	NM_001142301.1	+	UTR
chr9	2707447	2709158	1712	34.99	64.3	*KCNV2*	NM_133497.3	+	Exonic
chr9	2719380	2719823	444	25.725	46.2	*KCNV2*	NM_133497.3	+	Exonic
chr9	32530461	32531029	569	17.535	26.4	*TOPORS*	NM_005802.4	-	UTR
chr9	32531031	32534388	3358	48.475	38.9	*TOPORS*	NM_005802.4	-	Exonic
chr9	32540696	32541030	335	18.695	70.7	*TOPORS*	NM_005802.4	-	Exonic
chr9	32542355	32542667	313	20.485	59.1	*TOPORS*	NM_005802.4	-	Exonic
chr9	101901266	101901576	311	9.63	68.2	*INVS*	NM_183245.1	+	UTR
chr9	101906518	101906788	271	27.63	43.2	*INVS*	NM_014425.2	+	Exonic
chr9	101928406	101928711	306	25.14	37.6	*INVS*	NM_014425.2	+	Exonic
chr9	102028104	102028403	300	27.425	37.7	*INVS*	NM_014425.2	+	Exonic
chr9	102031682	102031997	316	23.65	40.8	*INVS*	NM_014425.2	+	Exonic
chr9	102042087	102042406	320	25.525	40.9	*INVS*	NM_014425.2	+	Exonic
chr9	102044578	102044863	286	14.09	31.1	*INVS*	NM_014425.2	+	Exonic
chr9	102048638	102048956	319	30.345	35.4	*INVS*	NM_014425.2	+	Exonic
chr9	102054309	102054599	291	29.525	38.5	*INVS*	NM_014425.2	+	Exonic
chr9	102054943	102055289	347	22.63	37.8	*INVS*	NM_014425.2	+	Exonic
chr9	102066832	102067055	224	18.475	32.6	*INVS*	NM_014425.2	+	Exonic
chr9	102074941	102075243	303	40.195	48.8	*INVS*	NM_014425.2	+	Exonic
chr9	102086346	102086766	421	17.81	53.2	*INVS*	NM_014425.2	+	Exonic
chr9	102094348	102095209	862	22.12	53.1	*INVS*	NM_014425.2	+	Exonic
chr9	102098939	102099297	359	28.825	49	*INVS*	NM_014425.2	+	Exonic
chr9	102099925	102100208	284	41.985	44	*INVS*	NM_014425.2	+	Exonic
chr9	102102591	102103250	660	37.185	40.6	*INVS*	NM_014425.2	+	Exonic
chr9	116204101	116205091	991	10.17	57.7	*DFNB31*	NM_015404.3	-	Exonic
chr9	116205228	116205509	282	11.13	62.4	*DFNB31*	NM_015404.3	-	Exonic
chr9	116205917	116206242	326	16.265	63.8	*DFNB31*	NM_015404.3	-	Exonic
chr9	116208376	116209059	684	9.675	64.2	*DFNB31*	NM_015404.3	-	Exonic
chr9	116209937	116210211	275	17.99	54.5	*DFNB31*	NM_015404.3	-	Exonic
chr9	116225339	116225673	315	8.935	61.5	*DFNB31*	NM_015404.3	-	Exonic

chr9	116226354	116226715	362	20.64	61	*DFNB31*	NM_015404.3	-	Exonic
chr9	116226967	116227242	276	21.075	52.5	*DFNB31*	NM_015404.3	-	Exonic
chr9	116228234	116228578	345	25.04	57.7	*DFNB31*	NM_015404.3	-	Exonic
chr9	116268283	116268566	284	21.84	59.5	*DFNB31*	NM_015404.3	-	Exonic
chr9	116280573	116280933	361	12.605	63.4	*DFNB31*	NM_015404.3	-	Exonic
chr9	116306217	116307605	1389	13.735	70.2	*DFNB31*	NM_015404.3	-	Exonic
chr9	118489294	118489557	264	6.47	75.4	*ASTN2*	NM_198188.2	-	UTR
chr9	118499683	118503458	3776	34.21	47.1	*TRIM32*	NM_012210.3	+	Exonic
chr9	138442813	138444135	1323	19.485	54.3	*INPP5E*	NM_019892.4	-	Exonic
chr9	138444467	138444748	282	13.32	62.1	*INPP5E*	NM_019892.4	-	Exonic
chr9	138445186	138445460	275	13.74	63.6	*INPP5E*	NM_019892.4	-	Exonic
chr9	138446063	138446315	253	11.595	67.2	*INPP5E*	NM_019892.4	-	Exonic
chr9	138446659	138446937	279	14.975	63.8	*INPP5E*	NM_019892.4	-	Exonic
chr9	138447142	138447426	285	18.34	62.5	*INPP5E*	NM_019892.4	-	Exonic
chr9	138447298	138447606	309	14.52	67.3	*INPP5E*	NM_019892.4	-	Exonic
chr9	138448209	138448495	287	16.2	66.2	*INPP5E*	NM_019892.4	-	Exonic
chr9	138448929	138449205	277	16.995	68.6	*INPP5E*	NM_019892.4	-	Exonic
chr9	138452802	138454136	1335	10.675	74.6	*INPP5E*	NM_019892.4	-	Exonic
chr10	48001417	48001709	293	13.965	29.7	*RBP3*	NM_002900.2	-	UTR
chr10	48001757	48002318	562	17.3	61.9	*RBP3*	NM_002900.2	-	Exonic
chr10	48003769	48004057	289	17.75	54.3	*RBP3*	NM_002900.2	-	Exonic
chr10	48005776	48006090	315	30.775	54	*RBP3*	NM_002900.2	-	Exonic
chr10	48007753	48011057	3305	24.545	63.6	*RBP3*	NM_002900.2	-	Exonic
chr10	55232480	55234239	1760	29.95	31.9	*PCDH15*	NM_001142771.1	-	UTR
chr10	55234240	55234693	454	25.05	36.1	*PCDH15*	NM_001142771.1	-	UTR
chr10	55234709	55235376	668	27.905	29.5	*PCDH15*	NM_001142771.1	-	UTR
chr10	55235395	55236326	932	20.3	29.2	*PCDH15*	NM_001142771.1	-	UTR
chr10	55236340	55236953	614	33.68	43.2	*PCDH15*	NM_001142772.1	-	Exonic
chr10	55240227	55240510	284	28.465	37.7	*PCDH15*	NM_001142769.1	-	Exonic
chr10	55257076	55257379	304	27.245	48.7	*PCDH15*	NM_001142767.1	-	Exonic
chr10	55258198	55258464	267	22.84	28.8	*PCDH15*	NM_001142765.1	-	Exonic
chr10	55261005	55261361	357	26.26	41.5	*PCDH15*	NM_001142767.1	-	Exonic
chr10	55270007	55270324	318	39.545	41.2	*PCDH15*	NM_001142767.1	-	Exonic
chr10	55286904	55287111	208	13.47	33.2	*PCDH15*	NM_001142767.1	-	Exonic
chr10	55296327	55296676	350	26.375	38.9	*PCDH15*	NM_001142767.1	-	Exonic
chr10	55332922	55333207	286	36.84	36.4	*PCDH15*	NM_001142767.1	-	Exonic
chr10	55368503	55368781	279	32.16	35.5	*PCDH15*	NM_001142767.1	-	Exonic
chr10	55370541	55370778	238	25.355	35.7	*PCDH15*	NM_001142767.1	-	Exonic
chr10	55389403	55389686	284	27.78	36.3	*PCDH15*	NM_001142767.1	-	Exonic
chr10	55391440	55391717	278	25.32	33.1	*PCDH15*	NM_001142767.1	-	Exonic
chr10	55425327	55425603	277	39.9	39	*PCDH15*	NM_001142767.1	-	Exonic
chr10	55449880	55450238	359	38.68	39.3	*PCDH15*	NM_001142767.1	-	Exonic
chr10	55452578	55452978	401	43.945	40.1	*PCDH15*	NM_001142767.1	-	Exonic
chr10	55496436	55496719	284	21.305	33.1	*PCDH15*	NM_001142767.1	-	Exonic
chr10	55508993	55509273	281	26.285	39.5	*PCDH15*	NM_001142767.1	-	Exonic

chr10	55519644	55519922	279	37.6	34.8	*PCDH15*	NM_001142767.1	-	Exonic
chr10	55562619	55562845	227	30.255	41	*PCDH15*	NM_001142767.1	-	Exonic
chr10	55582790	55583123	334	31.785	46.4	*PCDH15*	NM_001142767.1	-	Exonic
chr10	55613134	55613423	290	35.84	37.2	*PCDH15*	NM_001142767.1	-	Exonic
chr10	55614861	55615101	241	20.315	37.3	*PCDH15*	NM_001142767.1	-	Exonic
chr10	55625368	55625671	304	29.035	37.5	*PCDH15*	NM_001142767.1	-	Exonic
chr10	55643610	55643882	273	28.25	34.8	*PCDH15*	NM_001142767.1	-	Exonic
chr10	55666497	55666771	275	20.06	35.6	*PCDH15*	NM_001142767.1	-	Exonic
chr10	55746958	55747264	307	27.1	42.7	*PCDH15*	NM_001142767.1	-	Exonic
chr10	55759266	55759554	289	26.17	32.2	*PCDH15*	NM_001142767.1	-	Exonic
chr10	55776042	55776322	281	30.61	30.6	*PCDH15*	NM_001142767.1	-	Exonic
chr10	55798810	55799101	292	22.59	37	*PCDH15*	NM_001142767.1	-	Exonic
chr10	55808502	55808765	264	23.315	38.3	*PCDH15*	NM_001142767.1	-	Exonic
chr10	55957462	55957725	264	18.73	34.1	*PCDH15*	NM_001142767.1	-	Exonic
chr10	55958010	55958227	218	16.295	32.6	*PCDH15*	NM_001142763.1	-	Exonic
chr10	56093847	56094134	288	51.325	36.5	*PCDH15*	NM_001142767.1	-	Exonic
chr10	56230625	56231130	506	62.47	41.5	*PCDH15*	NM_001142771.1	-	UTR
chr10	72826664	72827138	475	3.86	75.4	*CDH23*	NM_052836.3	+	UTR
chr10	72869478	72869762	285	13.345	62.1	*CDH23*	NM_022124.5	+	Exonic
chr10	72875973	72876249	277	15.445	57.4	*CDH23*	NM_022124.5	+	Exonic
chr10	72939765	72940047	283	18.21	58.3	*CDH23*	NM_022124.5	+	Exonic
chr10	72940615	72941064	450	17.77	59.6	*CDH23*	NM_022124.5	+	Exonic
chr10	72996437	72996758	322	31.4	62.4	*CDH23*	NM_022124.5	+	Exonic
chr10	73000481	73000748	268	45.925	52.2	*CDH23*	NM_022124.5	+	Exonic
chr10	73007569	73007848	280	23.83	58.6	*CDH23*	NM_022124.5	+	Exonic
chr10	73045177	73045456	280	21.855	61.4	*CDH23*	NM_022124.5	+	Exonic
chr10	73046892	73047212	321	39.04	55.5	*CDH23*	NM_022124.5	+	Exonic
chr10	73075512	73075808	297	18.02	58.2	*CDH23*	NM_022124.5	+	Exonic
chr10	73076143	73076445	303	20.815	57.8	*CDH23*	NM_022124.5	+	Exonic
chr10	73104760	73105039	280	17.28	57.1	*CDH23*	NM_022124.5	+	Exonic
chr10	73107153	73107517	365	20.725	58.9	*CDH23*	NM_022124.5	+	Exonic
chr10	73109053	73109338	286	17.26	61.2	*CDH23*	NM_022124.5	+	Exonic
chr10	73112121	73112406	286	36.87	54.9	*CDH23*	NM_022124.5	+	Exonic
chr10	73117296	73117577	282	17.66	60.6	*CDH23*	NM_022124.5	+	Exonic
chr10	73120140	73120419	280	18.665	65	*CDH23*	NM_022124.5	+	Exonic
chr10	73123850	73124099	250	20.32	64	*CDH23*	NM_022124.5	+	Exonic
chr10	73125085	73125360	276	29.185	60.9	*CDH23*	NM_022124.5	+	Exonic
chr10	73131706	73132037	332	17.575	64.2	*CDH23*	NM_022124.5	+	Exonic
chr10	73132236	73132520	285	22.51	56.8	*CDH23*	NM_022124.5	+	Exonic
chr10	73134598	73134954	357	20.255	66.1	*CDH23*	NM_022124.5	+	Exonic
chr10	73136579	73136874	296	18.595	66.2	*CDH23*	NM_022124.5	+	Exonic
chr10	73138767	73139046	280	22.575	63.6	*CDH23*	NM_022124.5	+	Exonic
chr10	73142352	73142642	291	21.165	61.9	*CDH23*	NM_022124.5	+	Exonic
chr10	73153692	73153970	279	8.62	57.3	*CDH23*	NM_022124.5	+	Exonic
chr10	73155058	73155350	293	16.71	64.8	*CDH23*	NM_022124.5	+	Exonic
chr10	73160150	73160435	286	18.975	64	*CDH23*	NM_022124.5	+	Exonic

chr10	73161669	73162194	526	31.615	56.8	*CDH23*	NM_022124.5	+	Exonic
chr10	73163908	73164182	275	19.915	59.6	*CDH23*	NM_022124.5	+	Exonic
chr10	73168179	73168479	301	18.835	62.1	*CDH23*	NM_022124.5	+	Exonic
chr10	73169325	73169607	283	20.47	60.8	*CDH23*	NM_022124.5	+	Exonic
chr10	73170498	73170781	284	30.675	58.1	*CDH23*	NM_022124.5	+	Exonic
chr10	73171377	73171748	372	22.57	64	*CDH23*	NM_022124.5	+	Exonic
chr10	73207367	73207735	369	34.715	57.2	*CDH23*	NM_022124.5	+	Exonic
chr10	73207862	73208133	272	27.655	59.2	*CDH23*	NM_022124.5	+	Exonic
chr10	73208951	73209266	316	16.91	58.9	*CDH23*	NM_022124.5	+	Exonic
chr10	73213971	73214247	277	13.445	62.1	*CDH23*	NM_022124.5	+	Exonic
chr10	73214573	73214921	349	26.295	59.6	*CDH23*	NM_022124.5	+	Exonic
chr10	73215298	73215569	272	27.4	58.1	*CDH23*	NM_022124.5	+	Exonic
chr10	73218607	73218891	285	16.36	58.2	*CDH23*	NM_022124.5	+	Exonic
chr10	73219964	73220243	280	18.285	64.6	*CDH23*	NM_022124.5	+	Exonic
chr10	73220827	73221145	319	24.415	62.7	*CDH23*	NM_022124.5	+	Exonic
chr10	73222888	73223462	575	40.975	57.6	*CDH23*	NM_022124.5	+	Exonic
chr10	73226778	73227063	286	19.285	56.6	*CDH23*	NM_022124.5	+	Exonic
chr10	73228038	73228397	360	25.29	59.4	*CDH23*	NM_022124.5	+	Exonic
chr10	73228798	73229076	279	22.465	57.7	*CDH23*	NM_022124.5	+	Exonic
chr10	73229177	73229449	273	20.42	56.8	*CDH23*	NM_022124.5	+	Exonic
chr10	73230343	73230594	252	13.445	52.8	*CDH23*	NM_022124.5	+	Exonic
chr10	73232580	73232903	324	37.72	59.9	*CDH23*	NM_022124.5	+	Exonic
chr10	73232863	73233247	385	28.27	61.3	*CDH23*	NM_022124.5	+	Exonic
chr10	73235501	73235814	314	20.175	65	*CDH23*	NM_022124.5	+	Exonic
chr10	73235839	73236123	285	27.75	59.6	*CDH23*	NM_022124.5	+	Exonic
chr10	73236954	73237243	290	27.445	62.1	*CDH23*	NM_022124.5	+	Exonic
chr10	73237178	73237557	380	34.33	61.8	*CDH23*	NM_022124.5	+	Exonic
chr10	73237499	73237842	344	27.58	60.8	*CDH23*	NM_022124.5	+	Exonic
chr10	73239507	73239903	397	30.94	62.5	*CDH23*	NM_022124.5	+	Exonic
chr10	73240133	73240410	278	12.51	59.4	*CDH23*	NM_022124.5	+	Exonic
chr10	73240987	73241444	458	18.7	63.3	*CDH23*	NM_022124.5	+	Exonic
chr10	73241452	73241631	180	15.14	63.3	*CDH23*	NM_022124.5	+	Exonic
chr10	73241567	73241886	320	10.33	65.6	*CDH23*	NM_022124.5	+	Exonic
chr10	73242207	73242442	236	15.835	64.8	*CDH23*	NM_022124.5	+	Exonic
chr10	73242445	73242723	279	16.82	59.5	*CDH23*	NM_022124.5	+	Exonic
chr10	73242908	73243187	280	8.97	62.9	*CDH23*	NM_022124.5	+	Exonic
chr10	73244638	73245769	1132	27.725	58.1	*CDH23*	NM_022124.5	+	Exonic
chr10	85994697	85994983	287	10.05	62.4	*RGR*	NM_002921.3	+	Exonic
chr10	85997251	85997544	294	15.665	63.6	*RGR*	NM_002921.3	+	Exonic
chr10	85998563	85998786	224	18.355	60.7	*RGR*	NM_002921.3	+	Exonic
chr10	86002513	86002810	298	15.315	56.4	*RGR*	NM_002921.3	+	Exonic
chr10	86003970	86004255	286	34.385	55.2	*RGR*	NM_002921.3	+	Exonic
chr10	86007540	86007813	274	25.45	52.6	*RGR*	NM_002921.3	+	Exonic
chr10	86008167	86008625	459	22.86	57.1	*RGR*	NM_002921.3	+	Exonic
chr10	86008688	86008760	73	6.655	41.1	*RGR*	NM_001012720.1	+	UTR
chr10	95341507	95341915	409	31.85	41.3	*RBP4*	NM_006744.3	-	Exonic

chr10	95343489	95343854	366	24.08	58.5	*RBP4*	NM_006744.3	-	Exonic
chr10	95350042	95350296	255	34.45	56.5	*RBP4*	NM_006744.3	-	Exonic
chr10	95350332	95350611	280	25.355	66.8	*RBP4*	NM_006744.3	-	Exonic
chr10	95350551	95350845	295	11.17	72.2	*RBP4*	NM_006744.3	-	Exonic
chr10	95350772	95350858	265	4.695	72.4	*RBP4*	NM_006744.3	-	Exonic
chr10	95350868	95351080	286	3.26	79.3	*RBP4*	NM_006744.3	-	UTR
chr10	95362258	95363015	758	26.995	54.7	*PDE6C*	NM_006204.3	+	Exonic
chr10	95370299	95370579	281	63.665	43.4	*PDE6C*	NM_006204.3	+	Exonic
chr10	95370503	95370819	317	56.21	42.6	*PDE6C*	NM_006204.3	+	Exonic
chr10	95371599	95371888	290	48.535	39	*PDE6C*	NM_006204.3	+	Exonic
chr10	95375218	95375493	276	44.375	40.9	*PDE6C*	NM_006204.3	+	Exonic
chr10	95376273	95376719	447	25.575	33.6	*PDE6C*	NM_006204.3	+	Exonic
chr10	95378883	95379153	271	40.955	50.9	*PDE6C*	NM_006204.3	+	Exonic
chr10	95384428	95384720	293	36.21	36.2	*PDE6C*	NM_006204.3	+	Exonic
chr10	95385163	95385442	280	30.475	37.1	*PDE6C*	NM_006204.3	+	Exonic
chr10	95386625	95386910	286	17.55	30.4	*PDE6C*	NM_006204.3	+	Exonic
chr10	95389737	95390022	286	33.765	41.3	*PDE6C*	NM_006204.3	+	Exonic
chr10	95390101	95390377	277	23.385	43	*PDE6C*	NM_006204.3	+	Exonic
chr10	95390571	95390850	280	25.005	38.2	*PDE6C*	NM_006204.3	+	Exonic
chr10	95395605	95395881	277	35.56	41.2	*PDE6C*	NM_006204.3	+	Exonic
chr10	95405407	95405691	285	27.985	33	*PDE6C*	NM_006204.3	+	Exonic
chr10	95408556	95409011	456	23.75	36	*PDE6C*	NM_006204.3	+	Exonic
chr10	95411695	95411940	246	31.73	39	*PDE6C*	NM_006204.3	+	Exonic
chr10	95412223	95412482	260	18.525	28.1	*PDE6C*	NM_006204.3	+	Exonic
chr10	95412695	95412977	283	36.215	40.3	*PDE6C*	NM_006204.3	+	Exonic
chr10	95415026	95415480	455	24.21	29.9	*PDE6C*	NM_006204.3	+	Exonic
chr11	17471953	17472551	599	14.485	50.9	*USH1C*	NM_005709.3	-	Exonic
chr11	17474758	17475042	285	12.7	58.9	*USH1C*	NM_005709.3	-	Exonic
chr11	17476189	17476469	281	21.525	59.1	*USH1C*	NM_005709.3	-	Exonic
chr11	17479073	17479355	283	20.97	59	*USH1C*	NM_005709.3	-	Exonic
chr11	17479483	17479765	283	17.26	60.4	*USH1C*	NM_005709.3	-	Exonic
chr11	17479935	17480212	278	11.49	55.8	*USH1C*	NM_005709.3	-	Exonic
chr11	17495410	17495691	282	13.385	61.7	*USH1C*	NM_005709.3	-	Exonic
chr11	17498904	17499189	286	24.02	51.7	*USH1C*	NM_005709.3	-	Exonic
chr11	17499355	17499632	278	31.615	47.8	*USH1C*	NM_005709.3	-	Exonic
chr11	17500830	17501102	273	15.785	66.7	*USH1C*	NM_005709.3	-	Exonic
chr11	17501211	17501502	292	23.02	59.6	*USH1C*	NM_005709.3	-	Exonic
chr11	17501396	17501688	293	16.95	58	*USH1C*	NM_005709.3	-	Exonic
chr11	17502468	17502756	289	24	59.2	*USH1C*	NM_005709.3	-	Exonic
chr11	17504371	17504631	261	15.23	60.5	*USH1C*	NM_005709.3	-	Exonic
chr11	17504759	17505029	271	14.855	57.6	*USH1C*	NM_005709.3	-	Exonic
chr11	17504966	17505209	244	7.94	63.9	*USH1C*	NM_005709.3	-	Exonic
chr11	17505299	17505537	239	7.64	56.5	*USH1C*	NM_005709.3	-	Exonic
chr11	17509201	17509481	281	19.535	65.1	*USH1C*	NM_005709.3	-	Exonic
chr11	17509401	17509733	333	17.69	62.8	*USH1C*	NM_005709.3	-	Exonic
chr11	17511263	17511538	276	34.695	54.7	*USH1C*	NM_005709.3	-	Exonic
chr11	17522315	17522592	278	12.58	73	*USH1C*	NM_005709.3	-	Exonic

chr11	61473854	61474535	682	23.105	57.9	*BEST1*	NM_001139443.1	+	UTR
chr11	61475758	61476069	312	24.255	59	*BEST1*	NM_004183.3	+	Exonic
chr11	61479056	61479339	284	21.845	62.7	*BEST1*	NM_004183.3	+	Exonic
chr11	61479687	61480056	370	17.14	71.4	*BEST1*	NM_004183.3	+	Exonic
chr11	61480812	61481110	299	26.45	57.9	*BEST1*	NM_004183.3	+	Exonic
chr11	61481324	61481607	284	12.055	58.5	*BEST1*	NM_004183.3	+	Exonic
chr11	61482115	61482416	302	11.28	60.3	*BEST1*	NM_004183.3	+	Exonic
chr11	61483438	61483711	274	17.975	53.6	*BEST1*	NM_004183.3	+	Exonic
chr11	61483859	61484154	296	19.92	59.5	*BEST1*	NM_004183.3	+	Exonic
chr11	61486242	61486995	754	32.18	53.6	*BEST1*	NM_004183.3	+	Exonic
chr11	61488076	61488554	479	39.855	40.7	*BEST1*	NM_004183.3	+	Exonic
chr11	62136712	62137943	1232	7.96	68.9	*ROM1*	NM_000327.3	+	Exonic
chr11	62138225	62138610	386	15.955	59.3	*ROM1*	NM_000327.3	+	Exonic
chr11	62138556	62139216	661	10.675	55.5	*ROM1*	NM_000327.3	+	Exonic
chr11	66034578	66034855	278	13.495	65.5	*BBS1*	NM_024649.4	+	Exonic
chr11	66034947	66035404	458	12.58	50	*BBS1*	NM_024649.4	+	Exonic
chr11	66038374	66038786	413	21.095	55.4	*BBS1*	NM_024649.4	+	Exonic
chr11	66039463	66040069	607	16.365	53.9	*BBS1*	NM_024649.4	+	Exonic
chr11	66043580	66043861	282	11.045	58.9	*BBS1*	NM_024649.4	+	Exonic
chr11	66045221	66045504	284	15.655	55.3	*BBS1*	NM_024649.4	+	Exonic
chr11	66047416	66047698	283	29.425	58	*BBS1*	NM_024649.4	+	Exonic
chr11	66047657	66047996	340	24.645	61.2	*BBS1*	NM_024649.4	+	Exonic
chr11	66050059	66050339	281	19.295	54.4	*BBS1*	NM_024649.4	+	Exonic
chr11	66050617	66050912	296	16.86	55.1	*BBS1*	NM_024649.4	+	Exonic
chr11	66053783	66054063	281	16.055	67.6	*BBS1*	NM_024649.4	+	Exonic
chr11	66054862	66055146	285	20.905	58.9	*BBS1*	NM_024649.4	+	Exonic
chr11	66055600	66055873	274	18.265	55.8	*BBS1*	NM_024649.4	+	Exonic
chr11	66055917	66057696	1780	23.805	51.7	*BBS1*	NM_024649.4	+	Exonic
chr11	66979313	66979890	578	12.12	64.7	*CABP4*	NM_145200.3	+	Exonic
chr11	66980102	66980554	453	14.365	65.6	*CABP4*	NM_145200.3	+	Exonic
chr11	66981529	66981790	262	9.885	63.7	*CABP4*	NM_145200.3	+	Exonic
chr11	66982348	66982625	278	8.43	62.9	*CABP4*	NM_145200.3	+	Exonic
chr11	66982588	66983091	504	12.92	58.7	*CABP4*	NM_145200.3	+	Exonic
chr11	76516895	76517244	350	9.21	62.6	*MYO7A*	NM_001127179.2	+	UTR
chr11	76519168	76519449	282	18.08	61	*MYO7A*	NM_001127180.1	+	Exonic
chr11	76531312	76531587	276	11.445	60.9	*MYO7A*	NM_001127180.1	+	Exonic
chr11	76536412	76536707	296	19.05	69.6	*MYO7A*	NM_001127180.1	+	Exonic
chr11	76544525	76544841	317	41.895	56.8	*MYO7A*	NM_001127180.1	+	Exonic
chr11	76545264	76545547	284	18.47	62.7	*MYO7A*	NM_001127180.1	+	Exonic
chr11	76545446	76545760	315	20.87	60.6	*MYO7A*	NM_001127180.1	+	Exonic
chr11	76545879	76546154	276	13.09	57.2	*MYO7A*	NM_001127180.1	+	Exonic
chr11	76546890	76547191	302	24.655	63.9	*MYO7A*	NM_001127180.1	+	Exonic
chr11	76548030	76548316	287	19.94	59.6	*MYO7A*	NM_001127180.1	+	Exonic
chr11	76548768	76549035	268	10.255	66	*MYO7A*	NM_001127180.1	+	Exonic
chr11	76549591	76549860	270	15.28	56.7	*MYO7A*	NM_001127180.1	+	Exonic

chr11	76550736	76551079	344	31.375	58.1	*MYO7A*	NM_001127180.1	+	Exonic
chr11	76551469	76551739	271	15.15	60.5	*MYO7A*	NM_001127180.1	+	Exonic
chr11	76554652	76554936	285	20.395	57.9	*MYO7A*	NM_001127180.1	+	Exonic
chr11	76561361	76561632	272	17.67	66.2	*MYO7A*	NM_001127180.1	+	Exonic
chr11	76563370	76563656	287	14.775	63.1	*MYO7A*	NM_001127180.1	+	Exonic
chr11	76563965	76564243	279	12.505	62.7	*MYO7A*	NM_001127180.1	+	Exonic
chr11	76566139	76566417	279	26.29	59.1	*MYO7A*	NM_001127180.1	+	Exonic
chr11	76567630	76567922	293	24.21	60.1	*MYO7A*	NM_001127180.1	+	Exonic
chr11	76568350	76568711	362	16.245	66.9	*MYO7A*	NM_001127180.1	+	Exonic
chr11	76568972	76569258	287	23.16	59.6	*MYO7A*	NM_001127180.1	+	Exonic
chr11	76569998	76570330	333	12.365	64	*MYO7A*	NM_001127180.1	+	Exonic
chr11	76570614	76570903	290	11.18	61.7	*MYO7A*	NM_001127180.1	+	Exonic
chr11	76571041	76571357	317	19.99	60.9	*MYO7A*	NM_001127180.1	+	Exonic
chr11	76571655	76571935	281	13.585	61.2	*MYO7A*	NM_001127180.1	+	Exonic
chr11	76573197	76573472	276	18.46	64.1	*MYO7A*	NM_001127180.1	+	Exonic
chr11	76577949	76578236	288	12.715	63.2	*MYO7A*	NM_001127180.1	+	Exonic
chr11	76578622	76578884	263	16.205	65.8	*MYO7A*	NM_001127180.1	+	Exonic
chr11	76579309	76579627	319	26.355	61.1	*MYO7A*	NM_001127180.1	+	Exonic
chr11	76580664	76581040	377	18.905	62.9	*MYO7A*	NM_001127180.1	+	Exonic
chr11	76582978	76583280	303	13.725	61.7	*MYO7A*	NM_001127180.1	+	Exonic
chr11	76586083	76586309	227	18.94	51.5	*MYO7A*	NM_001127180.1	+	Exonic
chr11	76587100	76587385	286	11.745	61.5	*MYO7A*	NM_001127180.1	+	Exonic
chr11	76588147	76588577	431	14.725	60.3	*MYO7A*	NM_001127180.1	+	Exonic
chr11	76590060	76590396	337	17.14	59.1	*MYO7A*	NM_001127180.1	+	Exonic
chr11	76590906	76591183	278	18.4	60.4	*MYO7A*	NM_001127180.1	+	Exonic
chr11	76591677	76591973	297	18.16	67.7	*MYO7A*	NM_001127180.1	+	Exonic
chr11	76592699	76592989	291	28.17	61.2	*MYO7A*	NM_001127180.1	+	Exonic
chr11	76594078	76594367	290	19.385	68.6	*MYO7A*	NM_001127180.1	+	Exonic
chr11	76594693	76594974	282	19.55	61	*MYO7A*	NM_001127180.1	+	Exonic
chr11	76595889	76596174	286	19.59	59.8	*MYO7A*	NM_001127180.1	+	Exonic
chr11	76597020	76597291	272	29.15	55.5	*MYO7A*	NM_001127180.1	+	Exonic
chr11	76597256	76597570	315	36.67	61	*MYO7A*	NM_001127180.1	+	Exonic
chr11	76599769	76600094	326	15.475	63.8	*MYO7A*	NM_001127180.1	+	Exonic
chr11	76600426	76600710	285	14.555	59.6	*MYO7A*	NM_001127180.1	+	Exonic
chr11	76601537	76601810	274	21.68	53.6	*MYO7A*	NM_001127180.1	+	Exonic
chr11	76602467	76602747	281	28.37	61.9	*MYO7A*	NM_001127180.1	+	Exonic
chr11	76603219	76603993	775	34.21	55	*MYO7A*	NM_001127180.1	+	Exonic
chr12	1771305	1771821	517	42.435	41.8	*CACNA2D4*	NM_172364.4	-	UTR
chr12	1771828	1773253	1426	32.265	53.1	*CACNA2D4*	NM_172364.4	-	Exonic
chr12	1774625	1774901	277	16.585	57.4	*CACNA2D4*	NM_172364.4	-	Exonic
chr12	1775000	1775284	285	13.195	57.9	*CACNA2D4*	NM_172364.4	-	Exonic
chr12	1776753	1777036	284	6.505	70.8	*CACNA2D4*	NM_172364.4	-	Exonic
chr12	1778965	1779251	287	14.135	56.8	*CACNA2D4*	NM_172364.4	-	Exonic
chr12	1779305	1779581	277	24.42	60.6	*CACNA2D4*	NM_172364.4	-	Exonic
chr12	1779691	1779969	279	21.93	64.5	*CACNA2D4*	NM_172364.4	-	Exonic
chr12	1780362	1780642	281	15.91	59.8	*CACNA2D4*	NM_172364.4	-	Exonic
chr12	1780890	1781102	213	17.195	58.2	*CACNA2D4*	NM_172364.4	-	Exonic

chr12	1789590	1789871	282	20.545	61.7	*CACNA2D4*	NM_172364.4	-	Exonic
chr12	1789819	1790114	296	20.64	59.8	*CACNA2D4*	NM_172364.4	-	Exonic
chr12	1790969	1791249	281	9.755	60.9	*CACNA2D4*	NM_172364.4	-	Exonic
chr12	1820058	1820340	283	16.69	56.9	*CACNA2D4*	NM_172364.4	-	Exonic
chr12	1823745	1824029	285	20.07	58.6	*CACNA2D4*	NM_172364.4	-	Exonic
chr12	1825919	1826202	284	19.43	65.5	*CACNA2D4*	NM_172364.4	-	Exonic
chr12	1833275	1833557	283	19.91	59	*CACNA2D4*	NM_172364.4	-	Exonic
chr12	1835362	1835762	401	27.745	54.1	*CACNA2D4*	NM_172364.4	-	Exonic
chr12	1837902	1838166	265	23.03	58.9	*CACNA2D4*	NM_172364.4	-	Exonic
chr12	1839453	1839737	285	30.575	51.2	*CACNA2D4*	NM_172364.4	-	Exonic
chr12	1853916	1854194	279	33.38	47.7	*CACNA2D4*	NM_172364.4	-	Exonic
chr12	1854575	1854858	284	34.27	46.5	*CACNA2D4*	NM_172364.4	-	Exonic
chr12	1857628	1857916	289	17.905	53.6	*CACNA2D4*	NM_172364.4	-	Exonic
chr12	1858277	1858557	281	11.89	61.6	*CACNA2D4*	NM_172364.4	-	Exonic
chr12	1859120	1859397	278	24.08	59	*CACNA2D4*	NM_172364.4	-	Exonic
chr12	1862210	1862488	279	15.32	61.3	*CACNA2D4*	NM_172364.4	-	Exonic
chr12	1863559	1863845	287	32.21	53	*CACNA2D4*	NM_172364.4	-	Exonic
chr12	1864126	1864382	257	21.92	59.9	*CACNA2D4*	NM_172364.4	-	Exonic
chr12	1864301	1864583	283	14.825	56.9	*CACNA2D4*	NM_172364.4	-	Exonic
chr12	1865280	1865555	276	31.7	53.6	*CACNA2D4*	NM_172364.4	-	Exonic
chr12	1865572	1865860	289	36.06	48.1	*CACNA2D4*	NM_172364.4	-	Exonic
chr12	1866320	1866600	281	17.6	54.8	*CACNA2D4*	NM_172364.4	-	Exonic
chr12	1886787	1887050	264	19.625	45.8	*CACNA2D4*	NM_172364.4	-	Exonic
chr12	1887225	1887520	296	14.385	63.2	*CACNA2D4*	NM_172364.4	-	Exonic
chr12	1889213	1889485	273	18.09	56.8	*CACNA2D4*	NM_172364.4	-	Exonic
chr12	1892358	1892637	280	26.28	56.1	*CACNA2D4*	NM_172364.4	-	Exonic
chr12	1894171	1894459	289	20.93	54.7	*CACNA2D4*	NM_172364.4	-	Exonic
chr12	1897597	1898197	601	10.495	63.1	*CACNA2D4*	NM_172364.4	-	Exonic
chr12	15017118	15017392	275	21.705	44.4	*PDE6H*	NM_006205.2	+	UTR
chr12	15022093	15022404	312	23.595	44.9	*PDE6H*	NM_006205.2	+	Exonic
chr12	15023249	15023532	284	24.68	41.2	*PDE6H*	NM_006205.2	+	Exonic
chr12	15025520	15026128	609	34.715	40.1	*PDE6H*	NM_006205.2	+	Exonic
chr12	54400372	54400625	254	16.27	51.6	*RDH5*	NM_006205.2	+	UTR
chr12	54401124	54401604	481	22.435	60.7	*RDH5*	NM_006205.2	+	Exonic
chr12	54401674	54402056	383	15.72	59.8	*RDH5*	NM_006205.2	+	Exonic
chr12	54403856	54404167	312	16.95	59.3	*RDH5*	NM_006205.2	+	Exonic
chr12	54404294	54404842	549	21.455	53.9	*RDH5*	NM_006205.2	+	Exonic
chr12	75262371	75262846	476	18.9	25.2	*BBS10*	NM_024685.3	-	UTR
chr12	75262851	75265761	2911	42.69	35.5	*BBS10*	NM_024685.3	-	Exonic
chr12	75266004	75266399	396	27.16	62.9	*BBS10*	NM_024685.3	-	Exonic
chr12	86966854	86966950	97	13.245	27.8	*C12orf29*	NM_001009894.2	+	UTR
chr12	86966965	86967139	175	15.745	29.7	*CEP290*	NM_025114.3	-	Exonic
chr12	86967150	86967235	86	13.825	34.9	*CEP290*	NM_025114.3	-	Exonic
chr12	86968156	86968433	278	23.95	30.2	*CEP290*	NM_025114.3	-	Exonic
chr12	86971496	86971743	248	20.125	33.1	*CEP290*	NM_025114.3	-	Exonic
chr12	86972134	86972202	69	20.205	30.4	*CEP290*	NM_025114.3	-	Intronic

chr12	86972204	86972410	207	26.305	32.4	*CEP290*	NM_025114.3	-	Exonic
chr12	86973406	86973683	278	23.105	28.4	*CEP290*	NM_025114.3	-	Exonic
chr12	86976675	86976974	300	37.545	34	*CEP290*	NM_025114.3	-	Exonic
chr12	86977717	86977943	227	21.985	31.7	*CEP290*	NM_025114.3	-	Exonic
chr12	86978667	86978966	300	15.03	28	*CEP290*	NM_025114.3	-	Exonic
chr12	86980497	86980781	285	43.055	33.7	*CEP290*	NM_025114.3	-	Exonic
chr12	86981805	86982073	269	27.435	29	*CEP290*	NM_025114.3	-	Exonic
chr12	86986406	86986625	220	16.195	30.5	*CEP290*	NM_025114.3	-	Exonic
chr12	86989126	86989423	298	24.825	32.9	*CEP290*	NM_025114.3	-	Exonic
chr12	86989608	86989895	288	20.14	30.2	*CEP290*	NM_025114.3	-	Exonic
chr12	86995042	86995295	254	16.39	30.7	*CEP290*	NM_025114.3	-	Exonic
chr12	86995527	86995883	357	14.25	26.1	*CEP290*	NM_025114.3	-	Exonic
chr12	86996923	86997162	240	18.435	35.8	*CEP290*	NM_025114.3	-	Exonic
chr12	86998012	86998319	308	30.515	33.4	*CEP290*	NM_025114.3	-	Exonic
chr12	87000858	87001205	348	19.645	31.6	*CEP290*	NM_025114.3	-	Exonic
chr12	87001658	87001940	283	30.505	30.4	*CEP290*	NM_025114.3	-	Exonic
chr12	87002413	87002826	414	22.205	32.1	*CEP290*	NM_025114.3	-	Exonic
chr12	87003863	87004142	280	18.515	33.9	*CEP290*	NM_025114.3	-	Exonic
chr12	87004204	87004478	275	20.525	34.9	*CEP290*	NM_025114.3	-	Exonic
chr12	87005672	87005913	242	15.965	26.4	*CEP290*	NM_025114.3	-	Exonic
chr12	87006914	87007417	504	32.13	36.9	*CEP290*	NM_025114.3	-	Exonic
chr12	87008546	87008824	279	35	35.5	*CEP290*	NM_025114.3	-	Exonic
chr12	87010512	87010796	285	35.77	34	*CEP290*	NM_025114.3	-	Exonic
chr12	87011598	87011952	355	24.75	29.9	*CEP290*	NM_025114.3	-	Exonic
chr12	87014701	87014986	286	14.61	28.3	*CEP290*	NM_025114.3	-	Exonic
chr12	87018946	87019117	172	17.48	39	*CEP290*	NM_025114.3	-	Intronic
chr12	87020670	87020979	310	25.185	36.5	*CEP290*	NM_025114.3	-	Exonic
chr12	87024502	87024878	377	38.29	31.8	*CEP290*	NM_025114.3	-	Exonic
chr12	87024767	87025087	321	25.66	28.3	*CEP290*	NM_025114.3	-	Exonic
chr12	87026882	87027165	284	19.515	25	*CEP290*	NM_025114.3	-	Exonic
chr12	87029033	87029326	294	22.145	30.3	*CEP290*	NM_025114.3	-	Exonic
chr12	87029531	87029804	274	14.475	31	*CEP290*	NM_025114.3	-	Exonic
chr12	87032247	87032526	280	27.38	31.4	*CEP290*	NM_025114.3	-	Exonic
chr12	87032907	87033179	273	20.865	27.1	*CEP290*	NM_025114.3	-	Exonic
chr12	87034896	87035125	230	16.145	28.7	*CEP290*	NM_025114.3	-	Exonic
chr12	87036308	87036732	425	26.31	31.5	*CEP290*	NM_025114.3	-	Exonic
chr12	87037961	87038248	288	17.85	28.5	*CEP290*	NM_025114.3	-	Exonic
chr12	87038828	87039139	312	38.39	35.6	*CEP290*	NM_025114.3	-	Exonic
chr12	87043090	87043295	206	6.445	23.3	*CEP290*	NM_025114.3	-	Exonic
chr12	87044134	87044417	284	31.29	29.2	*CEP290*	NM_025114.3	-	Exonic
chr12	87046752	87047024	273	11.745	27.1	*CEP290*	NM_025114.3	-	Exonic
chr12	87047521	87047831	311	17.08	25.1	*CEP290*	NM_025114.3	-	Exonic
chr12	87048096	87048394	299	34.735	30.8	*CEP290*	NM_025114.3	-	Exonic
chr12	87048287	87048538	252	26.51	30.2	*CEP290*	NM_025114.3	-	Exonic
chr12	87048949	87049146	198	9.78	29.8	*CEP290*	NM_025114.3	-	Exonic
chr12	87054470	87054759	290	23.35	32.4	*CEP290*	NM_025114.3	-	Exonic
chr12	87056925	87057110	186	12.875	23.1	*CEP290*	NM_025114.3	-	Exonic

chr12	87057115	87057209	95	6.565	20	*CEP290*	NM_025114.3	-	Splice site
chr12	87057307	87057552	246	5.64	26.8	*CEP290*	NM_025114.3	-	Exonic
chr12	87058767	87059038	272	17.07	30.1	*CEP290*	NM_025114.3	-	Exonic
chr12	87059027	87059311	285	17.635	31.6	*CEP290*	NM_025114.3	-	Exonic
chr12	87059727	87060147	421	38.5	59.9	*CEP290*	NM_025114.3	-	UTR
chr13	113369522	113370461	940	15.425	63.5	*GRK1*	NM_002929.2	+	Exonic
chr13	113371920	113372194	275	23.445	56.4	*GRK1*	NM_002929.2	+	Exonic
chr13	113373738	113373954	217	30.65	60.4	*GRK1*	NM_002929.2	+	Exonic
chr14	20825868	20826151	284	36.46	43.7	*RPGRIP1*	NM_020366.3	+	Exonic
chr14	20832592	20832837	246	15.645	39.4	*RPGRIP1*	NM_020366.3	+	Exonic
chr14	20838904	20839295	392	15.93	63.8	*RPGRIP1*	NM_020366.3	+	Exonic
chr14	20840384	20840666	283	29.91	45.6	*RPGRIP1*	NM_020366.3	+	Exonic
chr14	20841254	20841605	352	32.025	40.3	*RPGRIP1*	NM_020366.3	+	Exonic
chr14	20845635	20845918	284	32.015	37.7	*RPGRIP1*	NM_020366.3	+	Exonic
chr14	20848445	20848731	287	23.16	27.2	*RPGRIP1*	NM_020366.3	+	Exonic
chr14	20849742	20850029	288	29.045	46.5	*RPGRIP1*	NM_020366.3	+	Exonic
chr14	20850323	20850601	279	23.68	39.8	*RPGRIP1*	NM_020366.3	+	Exonic
chr14	20855616	20855901	286	23.555	51.4	*RPGRIP1*	NM_020366.3	+	Exonic
chr14	20857939	20858237	299	41.195	42.8	*RPGRIP1*	NM_020366.3	+	Exonic
chr14	20859182	20859458	277	34.635	42.6	*RPGRIP1*	NM_020366.3	+	Exonic
chr14	20859773	20860067	295	43.585	43.4	*RPGRIP1*	NM_020366.3	+	Exonic
chr14	20862537	20863135	599	33.67	50.3	*RPGRIP1*	NM_020366.3	+	Exonic
chr14	20863153	20863445	293	22.84	51.9	*RPGRIP1*	NM_020366.3	+	Exonic
chr14	20863760	20864184	425	42.085	49.6	*RPGRIP1*	NM_020366.3	+	Exonic
chr14	20865541	20865871	331	32.13	44.4	*RPGRIP1*	NM_020366.3	+	Exonic
chr14	20866347	20866686	340	31.895	40.9	*RPGRIP1*	NM_020366.3	+	Exonic
chr14	20868176	20868427	252	14.215	37.3	*RPGRIP1*	NM_020366.3	+	Exonic
chr14	20872506	20872782	277	28.795	44.8	*RPGRIP1*	NM_020366.3	+	Exonic
chr14	20880955	20881295	341	25.345	44.9	*RPGRIP1*	NM_020366.3	+	Exonic
chr14	20883008	20883286	279	17.91	39.4	*RPGRIP1*	NM_020366.3	+	Exonic
chr14	20886110	20886365	256	27.605	32.4	*RPGRIP1*	NM_020366.3	+	Exonic
chr14	20889026	20889362	337	38.465	39.2	*RPGRIP1*	NM_020366.3	+	Exonic
chr14	23619125	23620681	1557	20.985	58.1	*NRL*	NM_006177.3	-	Exonic
chr14	23621459	23621989	531	14.335	63.5	*NRL*	NM_006177.3	-	Exonic
chr14	23623474	23623747	274	11.635	56.6	*NRL*	NM_006177.3	-	UTR
chr14	67238230	67238327	98	9.61	29.6		NM_006177.3		
chr14	67257195	67257482	288	41.51	38.9	*RDH12*	NM_152443.2	+	UTR
chr14	67258813	67259239	427	25.99	51.8	*RDH12*	NM_152443.2	+	Exonic
chr14	67260871	67261125	255	16.36	52.5	*RDH12*	NM_152443.2	+	Exonic
chr14	67261492	67261783	292	22.875	50	*RDH12*	NM_152443.2	+	Exonic
chr14	67262423	67262702	280	29.345	43.9	*RDH12*	NM_152443.2	+	Exonic
chr14	67263383	67263717	335	33.88	54.6	*RDH12*	NM_152443.2	+	Exonic
chr14	67265581	67265912	332	22.465	62.3	*RDH12*	NM_152443.2	+	Exonic
chr14	67270135	67270965	831	26.805	48.4	*RDH12*	NM_152443.2	+	Exonic
chr14	87921685	87921991	307	18.425	68.7	*SPATA7*	NM_001040428.3	+	Exonic

chr14	87927394	87927649	256	19.405	32.4	*SPATA7*	NM_001040428.3	+	Exonic
chr14	87929390	87929671	282	14.89	30.5	*SPATA7*	NM_018418.4	+	Exonic
chr14	87932126	87932340	215	24.835	33	*SPATA7*	NM_001040428.3	+	Exonic
chr14	87952726	87952958	233	22.335	28.8	*SPATA7*	NM_001040428.3	+	Exonic
chr14	87962253	87962846	594	40.615	41.8	*SPATA7*	NM_001040428.3	+	Exonic
chr14	87963614	87963897	284	18.41	29.6	*SPATA7*	NM_001040428.3	+	Exonic
chr14	87965352	87965624	273	29.905	33.7	*SPATA7*	NM_001040428.3	+	Exonic
chr14	87967150	87967424	275	33.305	34.2	*SPATA7*	NM_001040428.3	+	Exonic
chr14	87969120	87969405	286	15.245	26.2	*SPATA7*	NM_001040428.3	+	Exonic
chr14	87973521	87973799	279	20.16	25.8	*SPATA7*	NM_001040428.3	+	Exonic
chr14	87973868	87974580	713	43.49	35.8	*SPATA7*	NM_001040428.3	+	Exonic
chr14	88360655	88360975	321	20.96	66	*TTC8*	NM_144596.2	+	Exonic
chr14	88375458	88375737	280	21.21	32.9	*TTC8*	NM_144596.2	+	Exonic
chr14	88376845	88377119	275	21.11	37.5	*TTC8*	NM_144596.2	+	Exonic
chr14	88377016	88377344	329	23.6	45.6	*TTC8*	NM_144596.2	+	Exonic
chr14	88377421	88377689	269	23.895	28.3	*TTC8*	NM_144596.2	+	Exonic
chr14	88379777	88380049	273	17.93	27.5	*TTC8*	NM_144596.2	+	Exonic
chr14	88388962	88389239	278	17.665	36.3	*TTC8*	NM_144596.2	+	Exonic
chr14	88393181	88393466	286	30.465	35.3	*TTC8*	NM_144596.2	+	Exonic
chr14	88397223	88397448	226	9.73	28.3	*TTC8*	NM_144596.2	+	Exonic
chr14	88406095	88406351	257	34.06	33.9	*TTC8*	NM_144596.2	+	Exonic
chr14	88407586	88407882	297	13.65	42.1	*TTC8*	NM_144596.2	+	Exonic
chr14	88408338	88408622	285	16.71	50.9	*TTC8*	NM_144596.2	+	Exonic
chr14	88411099	88411256	158	27.21	31	*TTC8*	NM_144596.2	+	Exonic
chr14	88413325	88413927	603	20.53	32.8	*TTC8*	NM_144596.2	+	Exonic
chr14	88413963	88414141	179	5.81	24	*TTC8*	NM_198310.2	+	UTR
chr15	29080765	29080836	72	7.635	34.7				
chr15	29080952	29081482	531	31.25	35	*TRPM1*	NM_002420.5	-	Exonic
chr15	29081487	29082623	1137	34.6	42.7	*TRPM1*	NM_002420.5	-	Exonic
chr15	29105551	29105836	286	24.765	57.7	*TRPM1*	NM_002420.5	-	Exonic
chr15	29106344	29106653	310	28.15	41.6	*TRPM1*	NM_002420.5	-	Exonic
chr15	29107748	29108025	278	18.865	45	*TRPM1*	NM_002420.5	-	Exonic
chr15	29108726	29109005	280	30.085	33.2	*TRPM1*	NM_002420.5	-	Exonic
chr15	29110397	29110721	325	42.995	46.2	*TRPM1*	NM_002420.5	-	Exonic
chr15	29112106	29112491	386	41.235	46.4	*TRPM1*	NM_002420.5	-	Exonic
chr15	29114958	29115239	282	22.15	49.3	*TRPM1*	NM_002420.5	-	Exonic
chr15	29117124	29117407	284	40.645	44.7	*TRPM1*	NM_002420.5	-	Exonic
chr15	29117453	29117727	275	30.445	36.4	*TRPM1*	NM_002420.5	-	Exonic
chr15	29119535	29119898	364	24.995	56	*TRPM1*	NM_002420.5	-	Exonic
chr15	29121369	29121692	324	42.08	55.9	*TRPM1*	NM_002420.5	-	Exonic
chr15	29121705	29121801	97	22.62	41.2	*TRPM1*	NM_002420.5	-	Exonic
chr15	29125567	29125845	279	28.95	30.8	*TRPM1*	NM_002420.5	-	Exonic
chr15	29126530	29126815	286	36.455	50.7	*TRPM1*	NM_002420.5	-	Exonic
chr15	29127261	29127483	223	24.245	36.8	*TRPM1*	NM_002420.5	-	Exonic
chr15	29128788	29129074	287	37.51	50.9	*TRPM1*	NM_002420.5	-	Exonic

chr15	29129828	29130144	317	19.395	54.9	*TRPM1*	NM_002420.5	-	Exonic
chr15	29139941	29140221	281	35.28	48.4	*TRPM1*	NM_002420.5	-	Exonic
chr15	29140827	29141108	282	22.895	48.6	*TRPM1*	NM_002420.5	-	Exonic
chr15	29141989	29142267	279	35.895	35.1	*TRPM1*	NM_002420.5	-	Exonic
chr15	29142536	29142851	316	22.66	57.6	*TRPM1*	NM_002420.5	-	Exonic
chr15	29145493	29145808	316	28.765	51.9	*TRPM1*	NM_002420.5	-	Exonic
chr15	29146472	29146756	285	42.505	43.5	*TRPM1*	NM_002420.5	-	Exonic
chr15	29147293	29147650	358	21.92	51.4	*TRPM1*	NM_002420.5	-	Exonic
chr15	29149445	29149768	324	26.28	50.3	*TRPM1*	NM_002420.5	-	Exonic
chr15	29156290	29156564	275	44.435	39.6	*TRPM1*	NM_002420.5	-	Exonic
chr15	29181037	29181311	275	25.995	57.1	*TRPM1*	NM_002420.5	-	UTR
chr15	69889871	69890315	445	12.185	61.6	*NR2E3*	NM_014249.2	+	Exonic
chr15	69890792	69891071	280	10.745	69.6	*NR2E3*	NM_014249.2	+	Exonic
chr15	69891034	69891343	310	12.525	67.4	*NR2E3*	NM_014249.2	+	Exonic
chr15	69891251	69891515	265	16.05	70.9	*NR2E3*	NM_014249.2	+	Exonic
chr15	69891525	69891639	115	23.41	60.9	*NR2E3*	NM_014249.2	+	Exonic
chr15	69891664	69891966	303	24.3	61.1	*NR2E3*	NM_014249.2	+	Exonic
chr15	69892707	69893087	381	19.1	60.9	*NR2E3*	NM_014249.2	+	Exonic
chr15	69893346	69893589	244	22.705	60.2	*NR2E3*	NM_014249.2	+	Exonic
chr15	69896869	69897232	364	20.675	48.4	*NR2E3*	NM_014249.2	+	Exonic
chr15	69897234	69897631	398	19.74	36.2	*NR2E3*	NM_014249.2	+	UTR
chr15	69897635	69897715	81	9.39	44.4	*NR2E3*	NM_014249.2	+	UTR
chr15	70765466	70765735	270	18.555	61.5	*BBS4*	NM_033028.4	+	Exonic
chr15	70774449	70774682	234	16.245	32.9	*BBS4*	NM_033028.4	+	Exonic
chr15	70788987	70789264	278	33.335	37.8	*BBS4*	NM_033028.4	+	Exonic
chr15	70791549	70791800	252	34.125	40.5	*BBS4*	NM_033028.4	+	Exonic
chr15	70794595	70794874	280	37.84	44.6	*BBS4*	NM_033028.4	+	Exonic
chr15	70796062	70796329	268	31.395	32.1	*BBS4*	NM_033028.4	+	Exonic
chr15	70802069	70802348	280	34.26	38.2	*BBS4*	NM_033028.4	+	Exonic
chr15	70803835	70804114	280	29.405	38.9	*BBS4*	NM_033028.4	+	Exonic
chr15	70807215	70807490	276	30.865	40.6	*BBS4*	NM_033028.4	+	Exonic
chr15	70808928	70809176	249	20.78	46.6	*BBS4*	NM_033028.4	+	Exonic
chr15	70810619	70810910	292	40.745	46.2	*BBS4*	NM_033028.4	+	Exonic
chr15	70810838	70811188	351	35.26	44.4	*BBS4*	NM_033028.4	+	Exonic
chr15	70814391	70814671	281	20.47	48.4	*BBS4*	NM_033028.4	+	Exonic
chr15	70815138	70815421	284	41.805	44	*BBS4*	NM_033028.4	+	Exonic
chr15	70816076	70816420	345	26.445	51.6	*BBS4*	NM_033028.4	+	Exonic
chr15	70816792	70817918	1127	29.88	42	*BBS4*	NM_033028.4	+	Exonic
chr15	87554025	87554739	715	20.33	49.5	*RLBP1*	NM_033028.4	-	Exonic
chr15	87554838	87555111	274	16.645	58	*RLBP1*	NM_033028.4	-	Exonic
chr15	87555899	87556198	300	48.35	51.7	*RLBP1*	NM_033028.4	-	Exonic
chr15	87559214	87559537	324	20.715	58	*RLBP1*	NM_033028.4	-	Exonic
chr15	87561275	87561619	345	23.25	63.2	*RLBP1*	NM_033028.4	-	Exonic
chr15	87562713	87562991	279	22.35	58.8	*RLBP1*	NM_033028.4	-	Exonic
chr15	87563134	87563386	253	14.005	51	*RLBP1*	NM_033028.4	-	Exonic

chr15	87563882	87564161	280	34.07	46.1	*RLBP1*	NM_033028.4	-	UTR
chr15	87565694	87565989	296	17.795	61.1	*RLBP1*	NM_033028.4	-	UTR
chr16	4322150	4322509	360	20.335	65.8	*GLIS2*	NM_032575.2	+	Exonic
chr16	4323278	4323582	305	13.62	63.6	*GLIS2*	NM_032575.2	+	Exonic
chr16	4324733	4325028	296	16.19	66.2	*GLIS2*	NM_032575.2	+	Exonic
chr16	4324953	4325248	296	19.02	61.8	*GLIS2*	NM_032575.2	+	Exonic
chr16	4325153	4325465	313	11.23	66.5	*GLIS2*	NM_032575.2	+	Exonic
chr16	4326650	4329549	2900	14.325	65	*GLIS2*	NM_032575.2	+	Exonic
chr16	4329558	4329647	90	10.235	45.6	*GLIS2*	NM_032575.2	+	UTR
chr16	28396021	28396524	504	19.43	54.8	*CLN3*	NM_001042432.1	-	Exonic
chr16	28396470	28396749	280	11.87	62.5	*CLN3*	NM_001042432.1	-	Exonic
chr16	28400823	28401094	272	10.19	62.5	*CLN3*	NM_001042432.1	-	Exonic
chr16	28400998	28401604	607	12.845	57.5	*CLN3*	NM_001042432.1	-	Exonic
chr16	28402736	28403017	282	13.47	55.3	*CLN3*	NM_001042432.1	-	Exonic
chr16	28405103	28405573	471	9.285	62.8	*CLN3*	NM_001042432.1	-	Exonic
chr16	28406171	28406448	278	18.445	57.9	*CLN3*	NM_001042432.1	-	Exonic
chr16	28406345	28406648	304	12.88	62.2	*CLN3*	NM_001042432.1	-	Exonic
chr16	28407298	28407570	273	6.65	56.8	*CLN3*	NM_001042432.1	-	Exonic
chr16	28408014	28408298	285	14.805	55.4	*CLN3*	NM_001042432.1	-	Exonic
chr16	28410192	28410477	286	21.795	60.5	*CLN3*	NM_001042432.1	-	Exonic
chr16	28410413	28410722	310	22.33	64.2	*CLN3*	NM_001042432.1	-	Exonic
chr16	28410764	28411176	413	13.57	58.8	*CLN3*	NM_000086.2	-	UTR
chr16	52191248	52193153	1906	34.35	33.9	*RPGRIP1L*	NM_001127897.1	-	UTR
chr16	52193158	52193652	495	22.565	40.4	*RPGRIP1L*	NM_015272.2	-	Exonic
chr16	52196814	52197097	284	32.685	48.6	*RPGRIP1L*	NM_015272.2	-	Exonic
chr16	52202275	52202551	277	31.95	34.7	*RPGRIP1L*	NM_015272.2	-	Exonic
chr16	52210362	52210642	281	30.485	37.4	*RPGRIP1L*	NM_015272.2	-	Exonic
chr16	52227743	52228022	280	27.955	33.2	*RPGRIP1L*	NM_015272.2	-	Exonic
chr16	52229051	52229325	275	34.04	35.6	*RPGRIP1L*	NM_015272.2	-	Exonic
chr16	52229623	52229911	289	14.245	32.2	*RPGRIP1L*	NM_015272.2	-	Exonic
chr16	52232338	52232605	268	21.25	32.8	*RPGRIP1L*	NM_015272.2	-	Exonic
chr16	52232622	52232948	327	24.975	33.9	*RPGRIP1L*	NM_015272.2	-	Exonic
chr16	52236997	52237478	482	36.32	38	*RPGRIP1L*	NM_015272.2	-	Exonic
chr16	52240301	52240587	287	20.01	35.9	*RPGRIP1L*	NM_015272.2	-	Exonic
chr16	52243869	52244455	587	47.015	35.4	*RPGRIP1L*	NM_015272.2	-	Exonic
chr16	52247793	52248077	285	39	34	*RPGRIP1L*	NM_015272.2	-	Exonic
chr16	52248785	52249104	320	18.93	29.4	*RPGRIP1L*	NM_015272.2	-	Exonic
chr16	52249703	52249982	280	46.815	34.3	*RPGRIP1L*	NM_015272.2	-	Exonic
chr16	52250088	52250361	274	30.4	29.2	*RPGRIP1L*	NM_015272.2	-	Exonic
chr16	52256206	52256488	283	48.535	39.9	*RPGRIP1L*	NM_015272.2	-	Exonic
chr16	52262813	52263070	258	21.01	27.9	*RPGRIP1L*	NM_015272.2	-	Exonic
chr16	52264207	52264482	276	36.275	32.6	*RPGRIP1L*	NM_015272.2	-	Exonic
chr16	52266333	52266612	280	24.205	31.4	*RPGRIP1L*	NM_015272.2	-	Exonic
chr16	52277766	52278043	278	33.94	34.2	*RPGRIP1L*	NM_015272.2	-	Exonic
chr16	52279216	52279457	242	19.855	28.5	*RPGRIP1L*	NM_015272.2	-	Exonic

chr16	52283423	52283843	421	31.535	39.7	*RPGRIP1L*	NM_015272.2	-	Exonic
chr16	52287485	52287763	279	26.915	35.5	*RPGRIP1L*	NM_015272.2	-	Exonic
chr16	52291948	52292229	282	29.375	34.4	*RPGRIP1L*	NM_015272.2	-	Exonic
chr16	52295098	52295379	282	19.87	66	*RPGRIP1L*	NM_001127897.1	-	UTR
chr16	55075694	55076308	615	27.255	35.3	*BBS2*	NM_031885.3	-	Exonic
chr16	55076922	55077219	298	44.71	37.2	*BBS2*	NM_031885.3	-	Exonic
chr16	55088287	55088570	284	51.14	41.2	*BBS2*	NM_031885.3	-	Exonic
chr16	55089074	55089360	287	37.475	35.5	*BBS2*	NM_031885.3	-	Exonic
chr16	55089765	55090041	277	20.735	36.5	*BBS2*	NM_031885.3	-	Exonic
chr16	55091110	55091382	273	28.045	40.3	*BBS2*	NM_031885.3	-	Exonic
chr16	55092187	55092488	302	12.105	45.7	*BBS2*	NM_031885.3	-	Exonic
chr16	55092700	55092972	273	13.42	46.9	*BBS2*	NM_031885.3	-	Exonic
chr16	55093652	55093931	280	42.8	49.6	*BBS2*	NM_031885.3	-	Exonic
chr16	55094008	55094280	273	40.8	38.8	*BBS2*	NM_031885.3	-	Exonic
chr16	55097255	55097721	467	52.105	37	*BBS2*	NM_031885.3	-	Exonic
chr16	55101258	55101518	261	39.43	40.2	*BBS2*	NM_031885.3	-	Exonic
chr16	55102152	55102433	282	39.225	41.1	*BBS2*	NM_031885.3	-	Exonic
chr16	55102485	55102769	285	27.26	38.2	*BBS2*	NM_031885.3	-	Exonic
chr16	55105789	55106138	350	31.71	38.3	*BBS2*	NM_031885.3	-	Exonic
chr16	55111093	55111557	465	14.845	70.3	*BBS2*	NM_031885.3	-	Exonic
chr16	56473664	56473731	68	10.5	25				
chr16	56473739	56474192	454	18.61	36.8	*CNGB1*	NM_001297.4	-	UTR
chr16	56474209	56474679	471	31.81	47.3	*CNGB1*	NM_001297.4	-	UTR
chr16	56475109	56475918	810	23.42	60.5	*CNGB1*	NM_001297.4	-	Exonic
chr16	56479184	56479541	358	25.72	57	*CNGB1*	NM_001297.4	-	Exonic
chr16	56488723	56488996	274	23.855	48.5	*CNGB1*	NM_001297.4	-	Exonic
chr16	56489114	56489395	282	27.17	57.8	*CNGB1*	NM_001297.4	-	Exonic
chr16	56492649	56493109	461	32.89	57.5	*CNGB1*	NM_001297.4	-	Exonic
chr16	56495150	56495449	300	14.52	60.3	*CNGB1*	NM_001297.4	-	Exonic
chr16	56496058	56496346	289	16.405	55.7	*CNGB1*	NM_001297.4	-	Exonic
chr16	56503068	56503353	286	22.56	55.2	*CNGB1*	NM_001297.4	-	Exonic
chr16	56504301	56504502	202	11.365	61.9	*CNGB1*	NM_001297.4	-	Exonic
chr16	56506551	56506825	275	7.315	60	*CNGB1*	NM_001297.4	-	Exonic
chr16	56507423	56507691	269	21.65	52.8	*CNGB1*	NM_001297.4	-	Exonic
chr16	56508595	56508950	356	20.425	58.1	*CNGB1*	NM_001297.4	-	Exonic
chr16	56510428	56510724	297	13.285	57.6	*CNGB1*	NM_001297.4	-	Exonic
chr16	56511714	56512006	293	28.635	60.4	*CNGB1*	NM_001297.4	-	Exonic
chr16	56514586	56514863	278	9.65	56.5	*CNGB1*	NM_001297.4	-	Exonic
chr16	56523043	56523343	80	6.49	54.5	*CNGB1*	NM_001297.4	-	Exonic
chr16	56530818	56531055	238	18.925	59.2	*CNGB1*	NM_001297.4	-	Exonic
chr16	56531544	56531815	272	18.585	60.7	*CNGB1*	NM_001297.4	-	Exonic
chr16	56540650	56540775	126	12.085	57.9	*CNGB1*	NM_001297.4	-	Exonic
chr16	56540798	56540936	139	7.05	55.4	*CNGB1*	NM_001297.4	-	Exonic
chr16	56541707	56542013	307	23.81	52.4	*CNGB1*	NM_001297.4	-	Exonic
chr16	56548618	56548897	280	14.54	56.1	*CNGB1*	NM_001135639.1	-	Exonic

chr16	56549706	56549978	273	23.4	61.2	*CNGB1*	NM_001135639.1	-	Exonic
chr16	56551212	56551528	317	7.485	67.5	*CNGB1*	NM_001135639.1	-	Exonic
chr16	56551761	56552044	284	12.535	65.8	*CNGB1*	NM_001135639.1	-	Exonic
chr16	56552132	56552417	286	9.915	64.3	*CNGB1*	NM_001135639.1	-	Exonic
chr16	56553843	56554123	281	17.99	58	*CNGB1*	NM_001135639.1	-	Exonic
chr16	56554092	56554555	464	15.67	63.6	*CNGB1*	NM_001135639.1	-	Exonic
chr16	56555420	56555705	286	16.655	59.1	*CNGB1*	NM_001135639.1	-	Exonic
chr16	56555790	56556051	262	14.055	61.5	*CNGB1*	NM_001135639.1	-	Exonic
chr16	56558456	56558765	310	17.265	62.3	*CNGB1*	NM_001135639.1	-	Exonic
chr16	56562345	56562628	284	18.68	56.3	*CNGB1*	NM_001297.4	-	UTR
chr17	1500656	1500820	165	7.545	47.9	*PRPF8*	NM_006445.3	-	UTR
chr17	1500822	1501066	245	17.425	57.1	*PRPF8*	NM_006445.3	-	Exonic
chr17	1501082	1501417	336	23.34	58.6	*PRPF8*	NM_006445.3	-	Exonic
chr17	1501352	1501664	313	32.805	55.3	*PRPF8*	NM_006445.3	-	Exonic
chr17	1501579	1501886	308	30.015	54.2	*PRPF8*	NM_006445.3	-	Exonic
chr17	1503509	1503786	278	44.45	50.4	*PRPF8*	NM_006445.3	-	Exonic
chr17	1503720	1504113	394	38.35	54.3	*PRPF8*	NM_006445.3	-	Exonic
chr17	1505327	1505637	311	17.625	50.8	*PRPF8*	NM_006445.3	-	Exonic
chr17	1506370	1506673	304	30.64	48.4	*PRPF8*	NM_006445.3	-	Exonic
chr17	1506565	1506874	310	26.515	49.7	*PRPF8*	NM_006445.3	-	Exonic
chr17	1508215	1508498	284	42.54	50.7	*PRPF8*	NM_006445.3	-	Exonic
chr17	1508455	1508864	410	36.815	48.8	*PRPF8*	NM_006445.3	-	Exonic
chr17	1509360	1509655	296	23.66	53.4	*PRPF8*	NM_006445.3	-	Exonic
chr17	1509805	1510108	304	25.885	45.4	*PRPF8*	NM_006445.3	-	Exonic
chr17	1510433	1510683	251	47.705	50.6	*PRPF8*	NM_006445.3	-	Exonic
chr17	1510620	1510936	317	55.31	50.5	*PRPF8*	NM_006445.3	-	Exonic
chr17	1510960	1511259	300	41.14	52.7	*PRPF8*	NM_006445.3	-	Exonic
chr17	1511199	1511509	311	38.855	47.6	*PRPF8*	NM_006445.3	-	Exonic
chr17	1511577	1511888	312	41.375	50.6	*PRPF8*	NM_006445.3	-	Exonic
chr17	1511848	1512237	390	30.725	45.4	*PRPF8*	NM_006445.3	-	Exonic
chr17	1523034	1523316	283	30.185	55.1	*PRPF8*	NM_006445.3	-	Exonic
chr17	1523320	1523667	348	44.53	56	*PRPF8*	NM_006445.3	-	Exonic
chr17	1523724	1524004	281	26.1	52.3	*PRPF8*	NM_006445.3	-	Exonic
chr17	1524425	1524780	356	27.565	48.9	*PRPF8*	NM_006445.3	-	Exonic
chr17	1525120	1525448	329	43.49	52	*PRPF8*	NM_006445.3	-	Exonic
chr17	1525585	1525918	334	39.92	50.3	*PRPF8*	NM_006445.3	-	Exonic
chr17	1525858	1526147	290	28.935	51.7	*PRPF8*	NM_006445.3	-	Exonic
chr17	1526175	1526472	298	25.5	52.3	*PRPF8*	NM_006445.3	-	Exonic
chr17	1526468	1526815	348	29.24	56.3	*PRPF8*	NM_006445.3	-	Exonic
chr17	1526951	1527270	320	33.11	50.6	*PRPF8*	NM_006445.3	-	Exonic
chr17	1527527	1527803	277	16.8	49.8	*PRPF8*	NM_006445.3	-	Exonic
chr17	1528537	1528764	228	58.095	47.4	*PRPF8*	NM_006445.3	-	Exonic
chr17	1528715	1528998	284	54.255	50.4	*PRPF8*	NM_006445.3	-	Exonic
chr17	1528950	1529302	353	31.13	51	*PRPF8*	NM_006445.3	-	Exonic
chr17	1529230	1529519	290	24.07	52.4	*PRPF8*	NM_006445.3	-	Exonic
chr17	1529572	1529909	338	24.025	53.3	*PRPF8*	NM_006445.3	-	Exonic

chr17	1530672	1530952	281	36.995	47.3	*PRPF8*	NM_006445.3	-	Exonic
chr17	1530852	1531136	285	29.14	42.1	*PRPF8*	NM_006445.3	-	Exonic
chr17	1531451	1531793	343	37.04	44.6	*PRPF8*	NM_006445.3	-	Exonic
chr17	1531751	1532123	373	39.215	51.7	*PRPF8*	NM_006445.3	-	Exonic
chr17	1532065	1532398	334	26.655	50.9	*PRPF8*	NM_006445.3	-	Exonic
chr17	1533500	1533807	308	22.555	49	*PRPF8*	NM_006445.3	-	Exonic
chr17	1534432	1534702	271	17.95	66.8	*PRPF8*	NM_006445.3	-	Exonic
chr17	1534726	1535006	281	16.86	66.5	*PRPF8*	NM_006445.3	-	UTR
chr17	6267726	6268453	728	24.085	52.1	*AIPL1*	NM_001033054.1	-	UTR
chr17	6268892	6269358	467	15.91	45	*AIPL1*	NM_001033054.1	-	UTR
chr17	6269407	6269945	539	15.345	65.1	*AIPL1*	NM_014336.3	-	Exonic
chr17	6270578	6270864	287	28.07	63.1	*AIPL1*	NM_014336.3	-	Exonic
chr17	6270808	6271162	355	21.77	64.5	*AIPL1*	NM_014336.3	-	Exonic
chr17	6272282	6272611	330	15.445	64.5	*AIPL1*	NM_014336.3	-	Exonic
chr17	6278973	6279299	327	9.91	62.4	*AIPL1*	NM_014336.3	-	Exonic
chr17	6295238	6296251	1014	22.6	49.1	*PITPNM3*	NM_031220.3	-	UTR
chr17	6296261	6296996	736	38.165	52	*PITPNM3*	NM_031220.3	-	UTR
chr17	6297206	6299711	2506	11.88	62	*PITPNM3*	NM_001165966.1	-	Exonic
chr17	6301553	6301836	284	11.125	59.2	*PITPNM3*	NM_001165966.1	-	Exonic
chr17	6305341	6305667	327	17.435	62.4	*PITPNM3*	NM_001165966.1	-	Exonic
chr17	6307698	6307988	291	21.75	59.8	*PITPNM3*	NM_001165966.1	-	Exonic
chr17	6308138	6308429	292	19.57	59.9	*PITPNM3*	NM_001165966.1	-	Exonic
chr17	6308606	6308888	283	23.655	59.4	*PITPNM3*	NM_001165966.1	-	Exonic
chr17	6312179	6312462	284	22.3	63.7	*PITPNM3*	NM_001165966.1	-	Exonic
chr17	6314223	6314515	293	10.15	65.9	*PITPNM3*	NM_001165966.1	-	Exonic
chr17	6315145	6315467	323	11.265	66.6	*PITPNM3*	NM_001165966.1	-	Exonic
chr17	6316622	6316931	310	10.73	63.5	*PITPNM3*	NM_001165966.1	-	Exonic
chr17	6318398	6318717	320	9.955	65.6	*PITPNM3*	NM_001165966.1	-	Exonic
chr17	6320995	6321314	320	16.085	58.4	*PITPNM3*	NM_001165966.1	-	Exonic
chr17	6321932	6322162	231	9.205	66.2	*PITPNM3*	NM_001165966.1	-	Exonic
chr17	6322544	6322837	294	9.055	64.6	*PITPNM3*	NM_001165966.1	-	Exonic
chr17	6327483	6327857	375	12.085	61.1	*PITPNM3*	NM_001165966.1	-	Exonic
chr17	6328147	6328423	277	19.49	57.8	*PITPNM3*	NM_001165966.1	-	Exonic
chr17	6347449	6347726	278	12.58	56.5	*PITPNM3*	NM_001165966.1	-	Exonic
chr17	6369306	6369589	284	25.105	56.3	*PITPNM3*	NM_031220.3	-	Exonic
chr17	6381930	6382210	281	16.12	55.2	*PITPNM3*	NM_001165966.1	-	Exonic
chr17	6400348	6400662	78	0	83.8	*PITPNM3*	NM_001165966.1	-	Exonic
chr17	7846598	7846875	278	6.265	69.1	*GUCY2D*	NM_000180.3	+	UTR
chr17	7847004	7847819	816	8.535	74.9	*GUCY2D*	NM_000180.3	+	Exonic
chr17	7847813	7848258	446	18.29	67.5	*GUCY2D*	NM_000180.3	+	Exonic
chr17	7850329	7850797	469	16.025	64.2	*GUCY2D*	NM_000180.3	+	Exonic
chr17	7850999	7851283	285	14.95	57.9	*GUCY2D*	NM_000180.3	+	Exonic
chr17	7851372	7851647	276	16.52	59.1	*GUCY2D*	NM_000180.3	+	Exonic
chr17	7851878	7852157	280	28.195	56.8	*GUCY2D*	NM_000180.3	+	Exonic
chr17	7853440	7853724	285	13.595	55.1	*GUCY2D*	NM_000180.3	+	Exonic

chr17	7856106	7856455	350	18.075	59.4	*GUCY2D*	NM_000180.3	+	Exonic
chr17	7856380	7856711	332	20.19	59	*GUCY2D*	NM_000180.3	+	Exonic
chr17	7857065	7857357	293	17.72	64.2	*GUCY2D*	NM_000180.3	+	Exonic
chr17	7857843	7858130	288	17.895	62.8	*GUCY2D*	NM_000180.3	+	Exonic
chr17	7858564	7858869	306	29.395	58.2	*GUCY2D*	NM_000180.3	+	Exonic
chr17	7858786	7859146	361	26.735	57.1	*GUCY2D*	NM_000180.3	+	Exonic
chr17	7859293	7859599	307	22.74	60.6	*GUCY2D*	NM_000180.3	+	Exonic
chr17	7859737	7860133	397	17.235	70.3	*GUCY2D*	NM_000180.3	+	Exonic
chr17	7860117	7860420	304	17.135	68.8	*GUCY2D*	NM_000180.3	+	Exonic
chr17	7860355	7860667	313	11.05	70.9	*GUCY2D*	NM_000180.3	+	Exonic
chr17	7864148	7864444	297	8.59	55.9	*GUCY2D*	NM_000180.3	+	UTR
chr17	23897771	23899046	1276	19.36	56.8	*UNC119*	NM_054035.2	-	Exonic
chr17	23899021	23899329	309	13.755	63.8	*UNC119*	NM_054035.2	-	Exonic
chr17	23899643	23899918	276	18.685	57.2	*UNC119*	NM_054035.2	-	Exonic
chr17	23903417	23903821	405	7.145	77	*UNC119*	NM_054035.2	-	Exonic
chr17	24079834	24080108	275	4.885	66.5	*NEK8*	NM_178170.2	+	Exonic
chr17	24085052	24085395	344	34.435	56.1	*NEK8*	NM_178170.2	+	Exonic
chr17	24085866	24086207	342	34.01	55	*NEK8*	NM_178170.2	+	Exonic
chr17	24086314	24086583	270	29.11	59.3	*NEK8*	NM_178170.2	+	Exonic
chr17	24088383	24088712	330	25.93	63.6	*NEK8*	NM_178170.2	+	Exonic
chr17	24088663	24089160	498	17.88	64.1	*NEK8*	NM_178170.2	+	Exonic
chr17	24089130	24089446	317	11.79	65.9	*NEK8*	NM_178170.2	+	Exonic
chr17	24089707	24089985	279	22.9	57	*NEK8*	NM_178170.2	+	Exonic
chr17	24090142	24090411	270	21.605	58.5	*NEK8*	NM_178170.2	+	Exonic
chr17	24091531	24091822	292	17.295	57.9	*NEK8*	NM_178170.2	+	Exonic
chr17	24091886	24092194	309	19.58	60.5	*NEK8*	NM_178170.2	+	Exonic
chr17	24092120	24092425	306	19.47	58.5	*NEK8*	NM_178170.2	+	Exonic
chr17	24092479	24092761	283	20.77	59	*NEK8*	NM_178170.2	+	Exonic
chr17	24093028	24093467	440	26.57	56.8	*NEK8*	NM_178170.2	+	Exonic
chr17	24093708	24093810	103	5.135	53.4	*NEK8*	NM_178170.2	+	UTR
chr17	53637720	53637835	116	19.965	43.1	*MKS1*	NM_017777.3	-	UTR
chr17	53637845	53638029	185	26.5	47.6	*MKS1*	NM_017777.3	-	UTR
chr17	53638035	53638557	523	17.64	55.1	*MKS1*	NM_001165927.1	-	Exonic
chr17	53638506	53638996	491	17.805	58.9	*MKS1*	NM_001165927.1	-	Exonic
chr17	53639365	53639641	277	35.535	55.2	*MKS1*	NM_001165927.1	-	Exonic
chr17	53640158	53640630	473	18.92	54.3	*MKS1*	NM_001165927.1	-	Exonic
chr17	53640762	53641024	263	24.23	49.4	*MKS1*	NM_001165927.1	-	Exonic
chr17	53642901	53643183	283	40.42	43.8	*MKS1*	NM_001165927.1	-	Exonic
chr17	53643215	53643495	281	34.785	53.7	*MKS1*	NM_001165927.1	-	Exonic
chr17	53644616	53644898	283	41.355	47.3	*MKS1*	NM_001165927.1	-	Exonic
chr17	53645246	53645529	284	21.955	52.8	*MKS1*	NM_001165927.1	-	Exonic
chr17	53646027	53646308	282	37.535	47.9	*MKS1*	NM_001165927.1	-	Exonic
chr17	53646532	53646809	278	7.3	54.3	*MKS1*	NM_001165927.1	-	Exonic
chr17	53646999	53647284	286	10.34	50	*MKS1*	NM_001165927.1	-	Exonic
chr17	53648370	53648665	296	46.37	40.9	*MKS1*	NM_001165927.1	-	Exonic

chr17	53648914	53649190	277	35.75	44.8	*MKS1*	NM_001165927.1	-	Exonic
chr17	53650889	53651168	280	22.655	44.3	*MKS1*	NM_001165927.1	-	Exonic
chr17	53651743	53652015	273	30.36	55.3	*MKS1*	NM_001165927.1	-	Exonic
chr17	55582018	55582296	279	10.305	75.3	*CA4*	NM_000717.3	+	Exonic
chr17	55587334	55587616	283	25.89	60.1	*CA4*	NM_000717.3	+	Exonic
chr17	55588624	55588914	291	21.54	59.1	*CA4*	NM_000717.3	+	Exonic
chr17	55589497	55589777	281	18.145	60.5	*CA4*	NM_000717.3	+	Exonic
chr17	55589699	55589993	295	16.28	59.7	*CA4*	NM_000717.3	+	Exonic
chr17	55590086	55590340	255	12.23	61.2	*CA4*	NM_000717.3	+	Exonic
chr17	55590317	55590653	337	20.065	60.8	*CA4*	NM_000717.3	+	Exonic
chr17	55591293	55591738	446	15.995	59.6	*CA4*	NM_000717.3	+	Exonic
chr17	70423705	70424005	301	7.46	58.5	*USH1G*	NM_173477.2	-	UTR
chr17	70424008	70424572	565	19.38	61.8	*USH1G*	NM_173477.2	-	UTR
chr17	70424660	70425817	1158	18.275	59.3	*USH1G*	NM_173477.2	-	Exonic
chr17	70427063	70428415	1353	21.96	66.2	*USH1G*	NM_173477.2	-	Exonic
chr17	70430524	70430989	466	9.505	69.7	*USH1G*	NM_173477.2	-	Exonic
chr17	72047645	72047942	298	17.205	65.1	*PRCD*	NM_001077620.2	+	Exonic
chr17	72048067	72048336	270	11.365	63.7	*PRCD*	NM_001077620.2	+	Exonic
chr17	72050154	72050396	243	12.72	60.9	*PRCD*	NM_001077620.2	+	Exonic
chr17	72050601	72050883	283	10.43	62.5	*PRCD*	NM_001077620.2	+	UTR
chr17	72051409	72053114	1706	22.25	59.7	*PRCD*	NM_001077620.2	+	UTR
chr19	53016833	53017139	307	14.58	61.2	*CRX*	NM_000554.4	+	UTR
chr19	53029394	53029680	287	20.155	57.8	*CRX*	NM_000554.4	+	Exonic
chr19	53031231	53031523	293	16.65	64.2	*CRX*	NM_000554.4	+	Exonic
chr19	53034318	53035465	1148	19.4	59.8	*CRX*	NM_000554.4	+	Exonic
chr19	53036068	53036438	371	18.535	38.8	*CRX*	NM_000554.4	+	UTR
chr19	53036451	53036648	198	10.795	42.4	*CRX*	NM_000554.4	+	UTR
chr19	53036663	53037374	712	17.605	49.7	*CRX*	NM_000554.4	+	UTR
chr19	53037662	53038458	797	12.86	52.3	*CRX*	NM_000554.4	+	UTR
chr19	59310521	59311048	528	33.625	58.9	*TFPT*	M_013342.3	-	UTR
chr19	59313387	59313701	315	13.87	54	*PRPF31*	NM_015629.3	+	Exonic
chr19	59313627	59313932	306	12.705	51	*PRPF31*	NM_015629.3	+	Exonic
chr19	59316957	59317215	259	17.565	63.3	*PRPF31*	NM_015629.3	+	Exonic
chr19	59317589	59317868	280	25.315	55.7	*PRPF31*	NM_015629.3	+	Exonic
chr19	59318547	59318830	284	20.3	59.5	*PRPF31*	NM_015629.3	+	Exonic
chr19	59318874	59319173	300	18.64	65.7	*PRPF31*	NM_015629.3	+	Exonic
chr19	59319610	59319908	299	22.085	66.6	*PRPF31*	NM_015629.3	+	Exonic
chr19	59321669	59321894	226	12.17	64.6	*PRPF31*	NM_015629.3	+	Exonic
chr19	59323198	59323452	255	13.76	69	*PRPF31*	NM_015629.3	+	Exonic
chr19	59323345	59323634	290	12.37	70.3	*PRPF31*	NM_015629.3	+	Exonic
chr19	59324161	59324645	485	15.81	67.6	*PRPF31*	NM_015629.3	+	Exonic
chr19	59326469	59327029	561	18.68	55.1	*PRPF31*	NM_015629.3	+	Exonic
chr20	2586974	2587544	571	26.815	53.6	*IDH3B*	NM_174856.1	-	Exonic
chr20	2588085	2588524	440	27.16	55.5	*IDH3B*	NM_174856.1	-	Exonic
chr20	2588609	2588880	272	22.35	54.4	*IDH3B*	NM_174856.1	-	Exonic

chr20	2588776	2589095	320	33.47	51.6	*IDH3B*	NM_174856.1	-	Exonic
chr20	2588989	2589717	729	36.7	53.6	*IDH3B*	NM_174856.1	-	Exonic
chr20	2592001	2592290	290	43.495	49	*IDH3B*	NM_006899.2	-	Exonic
chr20	2592196	2592486	291	30.32	58.8	*IDH3B*	NM_006899.2	-	Exonic
chr20	2592435	2592707	273	31.12	63.7	*IDH3B*	NM_006899.2	-	Exonic
chr20	2592655	2592958	304	27.21	60.5	*IDH3B*	NM_006899.2	-	Exonic
chr20	10333756	10334390	635	44.445	38.9	*MKKS*	NM_170784.1	-	Exonic
chr20	10336173	10336452	280	41.83	39.3	*MKKS*	NM_170784.1	-	Exonic
chr20	10337200	10337513	314	32.25	36.6	*MKKS*	NM_170784.1	-	Exonic
chr20	10341097	10342592	1496	46.66	38.2	*MKKS*	NM_170784.1	-	Exonic
chr20	10349100	10349465	366	40.14	40.7	*MKKS*	NM_170784.1	-	UTR
chr20	10362665	10362936	272	22.695	70.2	*MKKS*	NM_170784.1	-	UTR
chrX	38013290	38013564	275	9.65	24.4	*RPGR*	NM_000328.2	-	UTR
chrX	38013571	38013811	241	11.415	27	*RPGR*	NM_000328.2	-	UTR
chrX	38013816	38014040	225	11.94	33.8	*RPGR*	NM_000328.2	-	Exonic
chrX	38017479	38017764	286	24.59	31.8	*RPGR*	NM_000328.2	-	Exonic
chrX	38019254	38019446	193	20.045	33.2	*RPGR*	NM_000328.2	-	Exonic
chrX	38020708	38021027	320	36.1	36.9	*RPGR*	NM_000328.2	-	Exonic
chrX	38031213	38031510	298	47.41	44.3	*RPGR*	NM_001034853.1	-	Exonic
chrX	38031980	38032271	292	34.675	39.4	*RPGR*	NM_001034853.1	-	Exonic
chrX	38035038	38035234	197	6.245	27.9	*RPGR*	NM_001034853.1	-	Exonic
chrX	38035496	38035764	269	14.755	30.5	*RPGR*	NM_001034853.1	-	Exonic
chrX	38041403	38041712	310	19.535	37.7	*RPGR*	NM_001034853.1	-	Exonic
chrX	38043077	38043399	323	31.455	39.3	*RPGR*	NM_001034853.1	-	Exonic
chrX	38045358	38045642	285	17.2	30.2	*RPGR*	NM_001034853.1	-	Exonic
chrX	38048756	38049052	297	16.77	34	*RPGR*	NM_001034853.1	-	Exonic
chrX	38054735	38055032	298	20.475	43.3	*RPGR*	NM_001034853.1	-	Exonic
chrX	38061447	38061722	276	20.785	36.6	*RPGR*	NM_001034853.1	-	Exonic
chrX	38062956	38063241	286	31.565	40.6	*RPGR*	NM_001034853.1	-	Exonic
chrX	38065108	38065377	270	20.25	37	*RPGR*	NM_001034853.1	-	Exonic
chrX	38066946	38067212	267	16.645	32.2	*RPGR*	NM_001034853.1	-	Exonic
chrX	38067509	38067785	277	19.47	31	*RPGR*	NM_001034853.1	-	Exonic
chrX	38071457	38071794	338	12.72	70.4	*RPGR*	NM_001034853.1	-	Exonic
chrX	41191591	41192161	571	15.11	54.5	*NYX*	NM_022567.2	+	Exonic
chrX	41217630	41219386	1757	15.61	69.2	*NYX*	NM_022567.2	+	Exonic
chrX	41219392	41219898	507	12.82	47.9	*NYX*	NM_022567.2	+	UTR
chrX	46581214	46581639	426	12.555	63.8	*RP2*	NM_006915.2	+	Exonic
chrX	46597776	46598566	791	39.9	41	*RP2*	NM_006915.2	+	Exonic
chrX	46604273	46604557	285	16.84	34.4	*RP2*	NM_006915.2	+	Exonic
chrX	46621776	46622055	280	16.525	31.8	*RP2*	NM_006915.2	+	Exonic
chrX	46624039	46625851	1813	17.36	30.3	*RP2*	NM_006915.2	+	Exonic
chrX	46626138	46626771	634	8.62	25.2	*RP2*	NM_006915.2	+	UTR
chrX	48948391	48948829	439	14.94	60.4	*CACNA1F*	NM_005183.2	-	Exonic
chrX	48948943	48949278	336	16.485	62.2	*CACNA1F*	NM_005183.2	-	Exonic
chrX	48949832	48950109	278	9.635	60.4	*CACNA1F*	NM_005183.2	-	Exonic

chrX	48950035	48950314	280	14.93	63.9	*CACNA1F*	NM_005183.2	-	Exonic
chrX	48950280	48950590	311	12.535	58.2	*CACNA1F*	NM_005183.2	-	Exonic
chrX	48951839	48952155	317	11.27	54.9	*CACNA1F*	NM_005183.2	-	Exonic
chrX	48952585	48952858	274	13.595	52.6	*CACNA1F*	NM_005183.2	-	Exonic
chrX	48952947	48953219	273	16.09	58.6	*CACNA1F*	NM_005183.2	-	Exonic
chrX	48953245	48953519	275	15.425	60	*CACNA1F*	NM_005183.2	-	Exonic
chrX	48953615	48953889	275	16.875	53.8	*CACNA1F*	NM_005183.2	-	Exonic
chrX	48953901	48954169	269	15.69	60.6	*CACNA1F*	NM_005183.2	-	Exonic
chrX	48954285	48954570	286	16.58	52.1	*CACNA1F*	NM_005183.2	-	Exonic
chrX	48954649	48954951	303	17.73	55.4	*CACNA1F*	NM_005183.2	-	Exonic
chrX	48955202	48955478	277	18.9	53.1	*CACNA1F*	NM_005183.2	-	Exonic
chrX	48955544	48955814	271	15.21	54.6	*CACNA1F*	NM_005183.2	-	Exonic
chrX	48955985	48956269	285	18.62	52.3	*CACNA1F*	NM_005183.2	-	Exonic
chrX	48956199	48956525	327	15.72	52	*CACNA1F*	NM_005183.2	-	Exonic
chrX	48957118	48957394	277	16.97	55.6	*CACNA1F*	NM_005183.2	-	Exonic
chrX	48957472	48957755	284	17.81	55.3	*CACNA1F*	NM_005183.2	-	Exonic
chrX	48958409	48958709	301	16.04	56.1	*CACNA1F*	NM_005183.2	-	Exonic
chrX	48958631	48959011	381	13.81	54.6	*CACNA1F*	NM_005183.2	-	Exonic
chrX	48959705	48959994	290	17.12	57.9	*CACNA1F*	NM_005183.2	-	Exonic
chrX	48961038	48961492	455	20.64	54.5	*CACNA1F*	NM_005183.2	-	Exonic
chrX	48961762	48962219	458	8.04	60.9	*CACNA1F*	NM_005183.2	-	Exonic
chrX	48962145	48962446	302	7.885	55	*CACNA1F*	NM_005183.2	-	Exonic
chrX	48962650	48962915	266	20.96	58.3	*CACNA1F*	NM_005183.2	-	Exonic
chrX	48962961	48963155	195	17.375	62.1	*CACNA1F*	NM_005183.2	-	Exonic
chrX	48963752	48964038	287	6.85	54.7	*CACNA1F*	NM_005183.2	-	Exonic
chrX	48964363	48964590	228	12.39	50.9	*CACNA1F*	NM_005183.2	-	Exonic
chrX	48965820	48966097	278	19.485	57.2	*CACNA1F*	NM_005183.2	-	Exonic
chrX	48965997	48966299	303	19.985	54.1	*CACNA1F*	NM_005183.2	-	Exonic
chrX	48966223	48966609	387	10.095	56.3	*CACNA1F*	NM_005183.2	-	Exonic
chrX	48968087	48968456	370	16.07	54.9	*CACNA1F*	NM_005183.2	-	Exonic
chrX	48969237	48969526	290	8.64	62.4	*CACNA1F*	NM_005183.2	-	Exonic
chrX	48969454	48969764	311	9.805	56.3	*CACNA1F*	NM_005183.2	-	Exonic
chrX	48969680	48970188	509	11.755	60.5	*CACNA1F*	NM_005183.2	-	Exonic
chrX	48970273	48970590	318	16.815	59.4	*CACNA1F*	NM_005183.2	-	Exonic
chrX	48971343	48971623	281	24.81	54.8	*CACNA1F*	NM_005183.2	-	Exonic
chrX	48971537	48971909	373	22.79	59.8	*CACNA1F*	NM_005183.2	-	Exonic
chrX	48973550	48973841	292	17.38	58.2	*CACNA1F*	NM_005183.2	-	Exonic
chrX	48973755	48974062	308	14.995	67.9	*CACNA1F*	NM_005183.2	-	Exonic
chrX	48974175	48974452	278	10.645	62.2	*CACNA1F*	NM_005183.2	-	Exonic
chrX	48974512	48974789	278	11.175	61.9	*CACNA1F*	NM_005183.2	-	Exonic
chrX	48975018	48975391	374	14.5	61.5	*CACNA1F*	NM_005183.2	-	Exonic
chrX	48976583	48976870	288	7.43	59	*CACNA1F*	NM_005183.2	-	Exonic
chrX	85002770	85004766	1997	19.13	33.6	*CHM*	NM_000390.2	-	UTR
chrX	85005025	85006536	1512	26.69	34.2	*CHM*	NM_000390.2	-	Exonic
chrX	85014633	85014901	269	26.86	36.1	*CHM*	NM_000390.2	-	Exonic

chrX	85020524	85020797	274	24.355	30.7	*CHM*	NM_000390.2	-	Exonic
chrX	85035751	85036025	275	23.275	36.7	*CHM*	NM_000390.2	-	Exonic
chrX	85042203	85042472	270	19.49	33.3	*CHM*	NM_000390.2	-	Exonic
chrX	85042648	85042930	283	22.015	36	*CHM*	NM_000390.2	-	Exonic
chrX	85052890	85053081	192	3.765	31.8	*CHM*	NM_000390.2	-	Exonic
chrX	85097733	85098102	370	11.955	36.5	*CHM*	NM_000390.2	-	Exonic
chrX	85099426	85099704	279	19.915	29	*CHM*	NM_000390.2	-	Exonic
chrX	85100431	85100704	274	23.885	31.4	*CHM*	NM_000390.2	-	Exonic
chrX	85105250	85105767	518	30.48	38.6	*CHM*	NM_000390.2	-	Exonic
chrX	85120339	85120619	281	32.99	35.2	*CHM*	NM_001145414.1	-	Exonic
chrX	85123283	85123488	206	12.85	35	*CHM*	NM_001145414.1	-	Exonic
chrX	85123492	85123559	68	10.905	26.5	*CHM*	NM_000390.2	-	Intronic
chrX	85169038	85169264	227	13.825	32.6	*CHM*	NM_001145414.1	-	Exonic
chrX	85189047	85189312	266	37.87	46.6	*CHM*	NM_001145414.1	-	Exonic

Chromosome: reference chromosome of the target. **Start position**: start position of the target on the chromosome. **End position**: end position of the target on the chromosome. **Size**: size of the target. **Avg coverage**: the amount of sequence mapped to the target divided by the target length**. % GC**: the percentage of G and C nucleotides of the target. **Gene name:** the gene that overlaps with the target. **RefSeq Id:** The RefSeq gene id of the gene that overlaps with the target. **Strand**: The strand on which the gene is located. **Location**: The location of the target within the gene: Exonic, Intronic or UTR.

Supp. Table S3A. Statistics of the 12 individual RD samples

**Sample**	**10064**	**10559**	**10719**	**13079**	**18459**	**18872**	**21067**	**21933**	**21974**	**29964**	**36392**	**44010**
**Sequencing statistics**												
Total mapped bases	39,446,322	40814589	32556436	23904345	31215044	20103801	20843364	34098716	28438916	27633825	30410359	25063233
Total bases mapped within target regions	24,545,061	25788618	21163111	14909845	19308649	12557905	12950224	21267750	17664790	17635347	19036137	15377105
% Total bases mapped within target regions	62.22%	63.18%	65.00%	62.37%	61.86%	62.47%	62.13%	62.37%	62.11%	63.82%	62.60%	61.35%
Total bases mapped near target regions	11,580,754	12088657	9611377	7516538	10100280	6654117	6800581	11361839	9506469	8647722	9809169	8251345
% Total bases mapped near target regions	29.36%	29.62%	29.52%	31.44%	32.36%	33.10%	32.63%	33.32%	33.43%	31.29%	32.26%	32.92%
Median coverage across target regions	29.99	32.36	27.03	18.15	23.86	14.76	15.79	22.66	21.77	21.97	23.36	19.09
Average coverage across target regions	32.77	34.84	28.85	19.80	25.97	16.73	17.57	24.21	23.81	23.90	25.68	20.64
% target regions covered >=10x	96.82%	97.22%	93.98%	85.58%	92.99%	76.63%	78.57%	93.83%	90.55%	90.35%	92.14%	87.52%
Evenness score	97.35%	96.49%	97.04%	96.22%	97.09%	96.70%	96.47%	96.85%	97.15%	97.12%	96.74%	96.94%
**Variant statistics**												
Total number of detected variants	1288	1361	1236	1059	1275	966	986	1305	1206	1059	1230	1213
Excluding intergenic variants	1142	1215	1159	979	1174	932	929	1224	1149	999	1156	1148
Excluding variants in UTRs	944	1017	981	813	977	776	762	1059	965	827	960	960
Exclude variants in Introns	216	243	255	209	232	197	190	250	230	191	223	252
Exclude synonymous variants	129	138	144	126	135	114	103	149	139	117	132	142
Exclude known SNPs	21	17	18	18	13	14	12	21	20	11	18	19
Exclude variants with >5% frequency in 100 exomes	21	17	16	18	13	14	11	19	20	11	17	19
Exclude variants with >15% frequency in 100 blindness samples	18	15	11	13	9	9	9	15	16	10	11	14
Excluding low quality variants (<10 reads or < 20% variant reads)	8	9	10	9	6	4	5	8	11	8	9	10

Total mapped bases: the total amount of sequence that was mapped to the NCBI 36.1 reference genome. **Total mapped bases within target regions**: total amount of sequence that was mapped to target regions. **% Total mapped bases within target regions**: the percentage of the mapped sequence that was mapped to the target regions. **Total bases mapped near target regions**: The amount of sequence mapped within a 500bp distance of any target. **% Total bases mapped near target regions**: the percentage of the mapped sequence that was mapped within a 500bp distance of the target regions. **Median coverage across target regions**: The median over all targets of the amount of sequence mapped to a target divided by the target length. **Average coverage across target regions**: The average over all targets of the amount of sequenced mapped to a target divided by the target length. **% target regions covered >= 10x**: The percentage of targets of which the average sequence coverage is 10 or more. **Evenness score**: score describing the uniformity of base coverage over targeted regions. **Variant statistics**: the number of detected variants after the application of different exclusion filters.

Supp. Table S3B. Statistics of the 100 individual RP samples

**Sample**	**7554**	**8322**	**8625**	**9398**	**9402**	**9437**	**9444**	**9470**	**9472**	**9493**	**9506**	**9509**	**9511**	**9518**	**9520**	**9527**	**9528**	**9537**	**9539**	**9543**
**Sequencing statistics**																				
Total mapped bases	35,318,903	23,100,765	25,451,848	28,699,692	26,956,395	30,241,905	32,204,733	24,028,168	18,732,046	31,113,077	26,791,315	32,079,797	27,908,181	29,407,208	34,820,857	25,540,550	28,444,001	32,679,048	20,257,429	29,159,175
Total bases mapped within target regions	22,941,616	13,868,266	16,751,287	16,991,540	16,766,596	19,317,221	20,519,202	15,158,049	10,903,378	18,620,924	17,186,613	20,234,367	17,869,716	17,093,406	21,493,991	15,815,845	18,040,248	21,220,827	12,774,883	18,453,094
% Total bases mapped within target regions	64.96	60.03	65.82	59.20	62.20	63.88	63.71	63.08	58.21	59.85	64.15	63.08	64.03	58.13	61.73	61.92	63.42	64.94	63.06	63.28
Total bases mapped near target regions	10,621,937	7,195,677	7,414,017	10,490,118	7,941,773	9,400,834	10,290,127	7,582,586	5,750,873	9,556,145	8,460,247	10,249,677	8,790,264	11,092,091	9,617,329	8,292,343	9,154,474	9,666,267	6,261,306	9,369,457
% Total bases mapped near target regions	30.07	31.15	29.13	36.55	29.46	31.09	31.95	31.56	30.70	30.71	31.58	31.95	31.50	37.72	27.62	32.47	32.18	29.58	30.91	32.13
Median coverage across target regions	28.81	17.03	21.56	20.72	21.17	24.07	26.22	18.83	13.76	23.61	21.55	25.38	22.69	21.12	28	19.75	22.8	26.95	16.18	23.43
Average coverage across target regions	30.68	18.77	22.50	22.35	22.31	25.54	27.50	20.65	14.43	24.97	22.87	27.40	24.38	22.49	28.85	21.53	24.74	28.53	17.53	25.12
% target regions covered >=10x	95.87	81.45	88.86	82.30	89.61	92.69	95.52	85.58	70.06	92.69	89.86	94.33	90.60	80.61	95.62	84.73	90.65	95.38	78.92	90.65
Evenness score	97.53%	96.95%	97.29%	97.31%	97.28%	97.30%	97.28%	97.26%	96.34%	97.15%	97.42%	97.43%	97.21%	97.38%	97.61%	97.15%	97.26%	97.45%	96.62%	97.31%
**Variant statistics**																				
Total number of detected variants	1329	1167	1148	1276	1398	1402	1330	1060	1292	1222	1261	1226	1182	1328	1474	1097	1138	1201	953	1178
Excluding intergenic variants	1193	1049	1069	1174	1164	1207	1254	997	988	1128	1192	1138	1107	1216	1192	984	1084	1102	884	1099
Excluding variants in UTRs	1006	874	888	961	976	1008	1024	846	833	945	1016	930	939	1034	1008	824	903	937	734	917
Exclude variants in Introns	222	205	226	186	212	233	238	211	229	200	244	223	211	210	233	197	243	196	201	220
Exclude synonymous variants	131	116	137	111	120	147	133	122	124	113	143	130	126	119	142	110	143	118	113	129
Exclude known SNPs	17	15	25	9	15	27	12	12	15	14	12	14	14	11	21	8	16	11	11	13
Exclude variants with >5% frequency in 100 exomes	17	15	25	8	15	27	12	12	15	14	11	14	14	10	21	7	15	11	11	12
Exclude variants with >15% frequency in 100 blindness samples	15	11	23	4	11	24	10	10	10	8	9	12	7	6	18	6	12	8	8	7
Excluding low quality variants (<10 reads or < 20% variant reads)	6	6	13	3	9	12	7	6	8	6	5	10	6	1	7	4	4	6	6	4

Sample	9552	11005	11319	11893	11919	13023	13480	14181	14182	14192	14267	14428	14705	14812	15569	16389	16430	17593	17597	17792
**Sequencing statistics**																				
Total mapped bases	26,640,697	23,159,231	24,486,791	31,482,579	35,012,219	31,803,061	36,283,211	30,097,915	33,384,582	29,255,610	29,040,386	29,841,777	24,736,433	33,763,979	30,855,605	26,925,121	29,466,254	41,210,335	23,395,249	25,874,338
Total bases mapped within target regions	16,641,700	14,473,538	15,221,646	20,477,646	22,612,278	20,770,916	23,053,594	19,084,185	21,053,662	18,899,026	18,127,269	18,458,727	16,287,802	20,686,594	19,340,741	16,691,382	17,823,018	24,253,837	14,235,137	15,177,457
% Total bases mapped within target regions	62.47	62.50	62.16	65.04	64.58	65.31	63.54	63.41	63.06	64.60	62.42	61.86	65.85	61.27	62.68	61.99	60.49	58.85	60.85	58.66
Total bases mapped near target regions	8,748,817	7,744,818	8,098,815	9,472,177	10,505,044	9,286,977	11,374,406	9,461,708	10,563,914	8,875,914	9,284,429	9,467,182	7,313,335	10,150,389	9,583,412	8,383,247	9,870,159	15,029,617	8,338,706	8,739,852
% Total bases mapped near target regions	32.84	33.44	33.07	30.09	30.00	29.20	31.35	31.44	31.64	30.34	31.97	31.72	29.57	30.06	31.06	31.14	33.50	36.47	35.64	33.78
Median coverage across target regions	20.45	18.2	19.2	26	28.18	26.2	29.08	24.19	27.18	23.6	23.37	23.09	20.84	25.88	24.43	21.54	21.38	30.23	17.05	17.86
Average coverage across target regions	22.39	19.64	20.19	27.34	30.17	27.91	30.70	25.39	28.17	25.18	24.19	24.66	21.78	28.21	25.83	22.44	23.31	31.97	19.15	20.65
% target regions covered >=10x	87.37	84.73	88.17	92.44	95.52	92.49	97.02	94.43	96.62	91.30	93.04	94.33	88.41	94.13	94.63	91.65	89.41	92.74	72.65	78.67
Evenness score	97.24%	96.99%	96.95%	97.36%	97.49%	97.61%	97.30%	97.30%	97.35%	97.28%	97.07%	97.27%	97.23%	97.29%	97.25%	97.06%	97.14%	97.73%	97.14%	97.06%
**Variant statistics**																				
Total number of detected variants	1156	1112	1197	1212	1375	1310	1428	1242	1360	1313	1201	1163	1173	1417	1357	1166	1331	1612	1172	978
Excluding intergenic variants	1107	1059	1136	1093	1212	1172	1301	1180	1275	1196	1135	1080	1050	1160	1235	1061	1207	1456	1067	915
Excluding variants in UTRs	934	883	945	900	1022	1004	1071	957	1065	1006	936	910	859	956	1024	896	987	1218	916	782
Exclude variants in Introns	208	208	216	195	242	243	245	230	252	221	208	217	217	255	247	216	215	220	218	203
Exclude synonymous variants	118	123	125	128	142	141	145	130	137	130	111	125	127	158	153	137	135	129	123	129
Exclude known SNPs	12	9	14	15	14	14	14	13	15	11	12	14	23	31	18	15	14	7	14	11
Exclude variants with >5% frequency in 100 exomes	11	9	14	13	13	14	13	12	15	11	11	14	22	31	18	15	14	7	14	11
Exclude variants with >15% frequency in 100 blindness samples	9	8	12	11	9	10	9	11	12	8	11	12	19	26	14	11	10	6	11	9
Excluding low quality variants (<10 reads or < 20% variant reads)	4	5	6	6	6	4	7	8	6	5	5	6	13	16	10	8	6	2	8	5

Sample	18060	18130	18336	18351	18390	18777	19051	19531	19689	19693	19733	19735	20703	20984	21010	21017	21141	21213	21334
**Sequencing statistics**																			
Total mapped bases	27,184,322	29,763,106	33,142,180	26,711,812	29,802,750	28,984,902	25,597,796	30,519,987	37,467,417	28,934,057	35,994,518	23,800,056	29,268,489	40,303,705	40,190,371	29,829,617	35,001,321	39,762,652	29,908,123
Total bases mapped within target regions	15,978,987	17,697,313	19,754,436	15,884,868	18,434,048	17,240,427	15,006,370	18,462,984	22,575,858	17,561,176	21,323,503	14,174,986	17,823,131	23,915,710	25,022,724	18,564,096	22,100,093	24,144,863	18,190,362
% Total bases mapped within target regions	58.78	59.46	59.61	59.47	61.85	59.48	58.62	60.49	60.25	60.69	59.24	59.56	60.90	59.34	62.26	62.23	63.14	60.72	60.82
Total bases mapped near target regions	8,779,877	9,825,806	10,570,117	8,692,068	9,152,079	9,205,795	8,516,123	9,934,754	13,521,977	9,410,057	12,413,426	8,406,937	10,070,431	14,418,818	12,945,815	9,878,295	11,434,530	12,630,044	10,422,358
% Total bases mapped near target regions	32.30	33.01	31.89	32.54	30.71	31.76	33.27	32.55	36.09	32.52	34.49	35.32	34.41	35.78	32.21	33.12	32.67	31.76	34.85
Median coverage across target regions	19.09	20.8	23.62	18.38	23.48	20.92	18.15	22.63	28.1	21.05	26.08	16.91	21.53	29.62	30.66	22.94	27.25	30.3	22.26
Average coverage across target regions	21.79	24.27	26.95	21.44	24.85	23.48	20.46	25.21	29.83	23.66	27.85	18.42	23.31	31.48	32.52	24.40	28.97	32.51	23.75
% target regions covered >=10x	83.69	83.19	88.36	80.56	92.29	86.33	79.31	87.92	89.96	84.93	93.09	80.56	89.46	92.74	94.53	88.86	92.29	96.22	89.61
Evenness score	97.20%	97.28%	97.28%	96.96%	97.25%	97.03%	96.95%	97.02%	97.73%	97.09%	97.52%	96.79%	97.22%	97.72%	97.47%	97.30%	97.45%	97.51%	97.19%
**Variant statistics**																			
Total number of detected variants	931	1008	1116	888	1325	1023	926	1030	1366	1050	1371	1130	1388	1609	1299	1253	1449	1461	1280
Excluding intergenic variants	845	944	1029	841	1161	966	861	972	1235	981	1227	1079	1314	1400	1195	1167	1359	1227	1194
Excluding variants in UTRs	696	801	868	685	975	808	724	824	1039	816	1025	904	1115	1188	991	991	1135	1027	1000
Exclude variants in Introns	185	205	216	194	256	192	184	202	209	230	233	194	235	233	204	210	249	232	221
Exclude synonymous variants	107	123	132	114	146	114	110	121	127	135	135	109	145	140	119	128	141	144	129
Exclude known SNPs	9	9	16	9	19	9	12	7	11	14	20	13	23	14	13	14	26	27	10
Exclude variants with >5% frequency in 100 exomes	9	8	15	9	18	9	12	7	11	14	18	13	23	13	13	14	26	27	10
Exclude variants with >15% frequency in 100 blindness samples	9	7	14	7	13	9	9	5	8	12	13	11	17	11	10	9	23	24	7
Excluding low quality variants (<10 reads or < 20% variant reads)	7	6	6	3	7	5	8	1	7	7	9	5	11	6	6	3	14	14	3

**Sample**	**21642**	**21933**	**22312**	**22315**	**22383**	**22393**	**22731**	**22777**	**23244**	**23422**	**27528**	**27585**	**27790**	**28064**	**28557**	**28692**	**29883**	**30364**	**31035**
**Sequencing statistics**																			
Total mapped bases	24,936,171	30,276,433	23,106,246	26,136,484	35,525,630	35,492,177	29,872,378	28,638,782	33,742,298	37,891,157	36,115,029	38,726,208	27,811,032	37,333,312	35,108,007	24,272,098	30,969,828	46,174,629	40,293,579
Total bases mapped within target regions	15,741,710	18,606,760	13,996,051	15,866,524	21,288,895	21,200,950	17,741,919	17,278,256	19,993,076	22,724,238	21,549,777	25,038,584	14,388,872	22,250,136	20,972,177	14,486,823	20,407,124	28,556,719	24,157,348
% Total bases mapped within target regions	63.13	61.46	60.57	60.71	59.93	59.73	59.39	60.33	59.25	59.97	59.67	64.66	51.74	59.60	59.74	59.69	65.89	61.85	59.95
Total bases mapped near target regions	7,973,782	10,300,282	8,027,073	9,086,111	10,999,144	11,357,174	9,724,137	8,961,549	10,921,216	11,691,918	11,435,221	11,513,070	6,284,744	13,616,728	10,521,919	7,465,756	9,168,368	13,618,796	12,337,410
% Total bases mapped near target regions	31.98	34.02	34.74	34.76	30.96	32.00	32.55	31.29	32.37	30.86	31.66	29.73	22.60	36.47	29.97	30.76	29.60	29.49	30.62
Median coverage across target regions	19.87	22.66	16.94	19.17	26.72	26.29	22.03	21.06	24.84	28.03	26.66	31.67	18.31	27.72	25.58	17.34	26.22	36.08	30.14
Average coverage across target regions	20.80	24.21	18.33	20.55	28.34	28.15	23.56	22.99	26.43	29.88	28.61	33.61	19.16	29.35	27.95	19.29	27.63	38.04	32.02
% target regions covered >=10x	88.12	90.05	79.51	84.88	92.69	93.19	88.96	88.17	90.95	93.59	94.13	94.78	84.98	90.95	91.05	79.86	89.86	95.28	93.83
Evenness score	97.16%	97.17%	96.85%	97.06%	97.26%	97.39%	97.32%	97.24%	97.28%	97.50%	97.52%	97.60%	96.99%	97.63%	97.45%	96.92%	97.50%	97.49%	97.47%
**Variant statistics**																			
Total number of detected variants	1336	1265	1245	1118	1540	1306	1271	1248	1474	1422	1410	1400	1376	1457	1322	1306	1287	1467	1435
Excluding intergenic variants	1200	1208	1170	1053	1243	1142	1130	1104	1284	1220	1237	1249	940	1291	1104	1126	1156	1241	1179
Excluding variants in UTRs	999	1042	978	869	1045	969	967	944	1089	1037	1013	1033	781	1096	919	958	966	1028	974
Exclude variants in Introns	235	258	227	167	242	214	226	221	248	240	234	230	192	223	217	225	233	211	236
Exclude synonymous variants	136	157	133	99	147	128	140	134	138	140	142	137	115	134	133	149	133	121	139
Exclude known SNPs	11	22	16	11	23	24	16	16	21	15	20	19	16	18	17	31	14	18	20
Exclude variants with >5% frequency in 100 exomes	11	20	16	11	23	23	16	16	20	15	20	19	16	18	16	31	14	18	19
Exclude variants with >15% frequency in 100 blindness samples	9	14	15	8	19	19	12	11	16	11	17	16	12	13	15	29	8	14	15
Excluding low quality variants (<10 reads or < 20% variant reads)	6	8	10	6	11	9	6	8	9	2	11	9	11	9	8	17	3	3	8

Sample	31343	31494	31723	31910	31933	31994	32111	32594	32655	32825	33626	33672	33884	34624	34886	36832	37360	37370	38290	41611
**Sequencing statistics**																				
Total mapped bases	27,079,474	47,135,901	15,122,598	16,770,111	30,907,472	31,244,140	21,619,374	40,754,589	31,744,517	39,667,401	28,265,016	27,369,987	41,452,915	29,194,731	31,495,920	43,736,891	39,399,792	36,576,468	35,977,731	40,470,464
Total bases mapped within target regions	17,501,769	29,647,700	7,983,610	10,345,426	19,658,071	20,285,116	13,298,723	26,688,186	20,777,993	23,588,785	18,154,824	17,196,929	24,656,503	19,007,330	21,021,822	26,235,512	23,932,654	22,077,079	23,759,393	22,211,896
% Total bases mapped within target regions	64.63	62.90	52.79	61.69	63.60	64.92	61.51	65.49	65.45	59.47	64.23	62.83	59.48	65.11	66.74	59.98	60.74	60.36	66.04	54.88
Total bases mapped near target regions	8,322,303	12,518,626	4,114,904	5,356,595	9,025,986	8,581,691	6,970,345	12,083,131	9,491,801	14,512,619	8,615,999	8,284,734	14,747,784	8,270,603	8,838,550	15,521,787	13,975,102	13,032,965	10,488,099	9,451,185
% Total bases mapped near target regions	30.73	26.56	27.21	31.94	29.20	27.47	32.24	29.65	29.90	36.59	30.48	30.27	35.58	28.33	28.06	35.49	35.47	35.63	29.15	23.35
Median coverage across target regions	21.79	38.09	9.67	12.9	25	25.78	16.68	33.91	26.41	29.1	22.9	21.94	30.52	24.21	27.03	32.36	27.07	27.07	30.18	28.07
Average coverage across target regions	23.46	39.26	10.45	13.63	26.11	27.18	17.43	35.75	27.76	30.83	24.06	22.93	32.18	25.33	28.14	34.15	28.86	28.86	31.98	29.56
% target regions covered >=10x	85.98	98.16	47.54	69.47	93.49	90.85	81.15	96.57	93.78	91.75	91.25	88.66	92.94	89.56	94.68	96.17	92.39	92.69	95.72	96.17
Evenness score	97.35%	97.64%	95.18%	96.18%	97.30%	97.53%	96.58%	97.49%	97.46%	97.72%	97.41%	97.27%	97.68%	97.41%	97.33%	97.71%	97.68%	97.57%	97.53%	97.48%
**Variant statistics**																				
Total number of detected variants	1178	1717	969	1034	1355	1190	1179	1447	1266	1380	1330	1392	1448	1306	1371	1612	1603	1466	1421	1621
Excluding intergenic variants	1091	1378	764	914	1175	1043	1025	1269	1109	1263	1186	1156	1270	1133	1222	1429	1444	1276	1222	1123
Excluding variants in UTRs	932	1152	601	760	991	844	839	1036	924	1048	999	993	1035	950	1023	1216	1231	1059	999	921
Exclude variants in Introns	209	264	170	201	219	205	208	245	231	193	244	220	195	221	243	226	251	211	231	216
Exclude synonymous variants	131	149	98	112	134	125	121	151	144	111	147	132	110	131	137	136	153	115	123	130
Exclude known SNPs	13	23	15	7	20	23	15	26	21	9	14	8	9	16	11	10	16	16	10	23
Exclude variants with >5% frequency in 100 exomes	12	23	14	7	20	23	15	26	20	8	14	8	9	16	11	10	16	16	10	23
Exclude variants with >15% frequency in 100 blindness samples	9	19	10	7	19	20	11	26	15	5	12	7	8	15	10	6	12	15	10	21
Excluding low quality variants (<10 reads or < 20% variant reads)	6	13	7	7	11	12	6	15	8	4	9	4	6	10	9	4	8	9	4	14

Total mapped bases: the total amount of sequence that was mapped to the NCBI 36.1 reference genome. **Total mapped bases within target regions**: total amount of sequence that was mapped to target regions. **% Total mapped bases within target regions**: the percentage of the mapped sequence that was mapped to the target regions. **Total bases mapped near target regions**: The amount of sequence mapped within a 500bp distance

of any target. **% Total bases mapped near target regions**: the percentage of the mapped sequence that was mapped within a 500bp distance of the target regions. **Median coverage across target regions**: The median over all targets of the amount of sequenced mapped to a target divided by the target length. **Average coverage across target regions**: The average over all targets of the amount of sequenced mapped to a target divided by the target length. **% target regions covered >= 10x**: The percentage of targets of which the average sequence coverage is 10 or more. **Evenness score**: score describing the uniformity of base coverage over targeted regions. **Variant statistics**: the number of detected variants after the application of different exclusion filters.

Supp. Table S4A. Proof of principle for the determination of pathogenicity

Gene	cDNA	Protein	SIFT	Polyphen	Mut pred	PhyloP	Frequency in exome	Reference	Classification Frequency	Classification predicitons	PhyloP Classification	Majority vote
ABCA4	c.2552G>A	p.G851D	Tolerated	Probably damaging	0.869	2.88	0.00	1	Pathogenic	Pathogenic	Pathogenic	Pathogenic
ABCA4	c.5657G>A	p.G1886E	Damaging	Probably damaging	0.905	5.28	0.00	1	Pathogenic	Pathogenic	Pathogenic	Pathogenic
ABCA4	c.6112C>T	p.R2038W	Damaging	Probably damaging	0.815	5.23	0.00	1	Pathogenic	Pathogenic	Pathogenic	Pathogenic
CNGA1	c.959C>T	p.S320F	Tolerated	Possibly damaging	0.836	3.72	0.00	2	Pathogenic	Unknown	Pathogenic	Pathogenic
CRX	c.122G>A	p.R41Q	Damaging	Probably damaging	0.934	5.41	0.00	3	Pathogenic	Pathogenic	Pathogenic	Pathogenic
CRX	c.239A>C	p.E80A	Damaging	Possibly damaging	0.902	5.16	0.00	3	Pathogenic	Pathogenic	Pathogenic	Pathogenic
CRX	c.268C>T	p.R90W	Damaging	Probably damaging	0.915	1.75	0.00	3	Pathogenic	Pathogenic	Unknown	Pathogenic
GNAT1	c.113G>A	p.G38D	Damaging	Probably damaging	0.949	4.44	0.00	4	Pathogenic	Pathogenic	Pathogenic	Pathogenic
GUCY2D	c.2512C>T	p.R838C	Damaging	Probably damaging	0.666	1.86	0.00	5	Pathogenic	Pathogenic	Unknown	Pathogenic
MERTK	c.2530C>T	p.R844C	Damaging	Probably damaging	0.964	3.56	0.00	6	Pathogenic	Pathogenic	Pathogenic	Pathogenic
NPHP4	c.2260G>A	p.G754R	Damaging	Probably damaging	0.832	1.43	0.00	7	Pathogenic	Pathogenic	Unknown	Pathogenic
NRL	c.479T>C	p.L160P	Damaging	Probably damaging	0.872	5.09	0.00	8	Pathogenic	Pathogenic	Pathogenic	Pathogenic
RDH12	c.139G>A	p.A47T	Damaging	Probably damaging	0.95	4.98	0.00	9	Pathogenic	Pathogenic	Pathogenic	Pathogenic
RDH12	c.451C>A	p.H151D	Damaging	Probably damaging	0.937	5.46	0.00	9	Pathogenic	Pathogenic	Pathogenic	Pathogenic
RDH12	c.854G>A	p.C285Y	Damaging	Probably damaging	0.859	6.23	0.00	9	Pathogenic	Pathogenic	Pathogenic	Pathogenic
RHO	c.68C>A	p.P23H	Damaging	Probably damaging	0.832	5.54	0.00	10	Pathogenic	Pathogenic	Pathogenic	Pathogenic
RLBP1	c.700C>T	p.R234W	Damaging	Probably damaging	0.971	1.82	0.00	11	Pathogenic	Pathogenic	Unknown	Pathogenic
RPE65	c.65T>C	p.L22P	Damaging	Benign	0.78	4.82	0.00	12	Pathogenic	Pathogenic	Pathogenic	Pathogenic
RPE65	c.74C>T	p.P25L	Damaging	Probably damaging	0.834	6.05	0.00	12	Pathogenic	Pathogenic	Pathogenic	Pathogenic
RPGRIP1	c.2627A>G	p.D876G	Damaging	Probably damaging	0.6	4.40	0.00	7	Pathogenic	Pathogenic	Pathogenic	Pathogenic

SIFT (13), **Polyphen** (14), **Mut pred** (15): prediction programs used for determination of pathogenicity. **PhyloP (16)**: PhyloP score for evolutionary conservation, **Frequency in exome**: the frequency at which the variant was found within the in-house database, **References**: Reference number for this mutation, **Classification Frequency**: final classification based on frequency, **Classification predicitons**: final classification based on prediction programs, **PhyloP Classification**: final classification based on PhyloP score, **Majority vote:** combined prediction based on a majority vote

Reference List

1 Sun H, Smallwood PM, Nathans J. Biochemical defects in ABCR protein variants associated with human retinopathies. Nat Genet 2000; 26(2):242-246.

2 Dryja TP, Finn JT, Peng YW, McGee TL, Berson EL, Yau KW. Mutations in the gene encoding the alpha subunit of the rod cGMP-gated channel in autosomal recessive retinitis pigmentosa. Proc Natl Acad Sci U S A 1995; 92(22):10177-10181.

3 Chen S, Wang QL, Xu S et al. Functional analysis of cone-rod homeobox (CRX) mutations associated with retinal dystrophy. Hum Mol Genet 2002; 11(8):873-884.

4 Muradov KG, Artemyev NO. Loss of the effector function in a transducin-alpha mutant associated with Nougaret night blindness. J Biol Chem 2000; 275(10):6969-6974.

5 Wilkie SE, Newbold RJ, Deery E et al. Functional characterization of missense mutations at codon 838 in retinal guanylate cyclase correlates with disease severity in patients with autosomal dominant cone-rod dystrophy. Hum Mol Genet 2000; 9(20):3065-3073.

6 McHenry CL, Liu Y, Feng W et al. MERTK arginine-844-cysteine in a patient with severe rod-cone dystrophy: loss of mutant protein function in transfected cells. Invest Ophthalmol Vis Sci 2004; 45(5):1456-1463.

7 Roepman R, Letteboer SJ, Arts HH et al. Interaction of nephrocystin-4 and RPGRIP1 is disrupted by nephronophthisis or Leber congenital amaurosis-associated mutations. Proc Natl Acad Sci U S A 2005; 102(51):18520-18525.

8 Nishiguchi KM, Friedman JS, Sandberg MA, Swaroop A, Berson EL, Dryja TP. Recessive NRL mutations in patients with clumped pigmentary retinal degeneration and relative preservation of blue cone function. Proc Natl Acad Sci U S A 2004; 101(51):17819-17824.

9 Thompson DA, Janecke AR, Lange J et al. Retinal degeneration associated with RDH12 mutations results from decreased 11-cis retinal synthesis due to disruption of the visual cycle. Hum Mol Genet 2005; 14(24):3865-3875.

10 Illing ME, Rajan RS, Bence NF, Kopito RR. A rhodopsin mutant linked to autosomal dominant retinitis pigmentosa is prone to aggregate and interacts with the ubiquitin proteasome system. J Biol Chem 2002; 277(37):34150-34160.

11 He X, Lobsiger J, Stocker A. Bothnia dystrophy is caused by domino-like rearrangements in cellular retinaldehyde-binding protein mutant R234W. Proc Natl Acad Sci U S A 2009; 106(44):18545-18550.

12 Lorenz B, Poliakov E, Schambeck M, Friedburg C, Preising MN, Redmond TM. A comprehensive clinical and biochemical functional study of a novel RPE65 hypomorphic mutation. Invest Ophthalmol Vis Sci 2008; 49(12):5235-5242.

13 Ng PC, Henikoff S. SIFT: Predicting amino acid changes that affect protein function. Nucleic Acids Res 2003; 31(13):3812-3814.

14 Adzhubei IA, Schmidt S, Peshkin L et al. A method and server for predicting damaging missense mutations. Nat Methods 2010; 7(4):248-249.

15 Li B, Krishnan VG, Mort ME et al. Automated inference of molecular mechanisms of disease from amino acid substitutions. Bioinformatics 2009; 25(21):2744-2750.

16 Pollard KS, Hubisz MJ, Rosenbloom KR, Siepel A. Detection of nonneutral substitution rates on mammalian phylogenies. Genome Res 2010; 20(1):110-121.

Supp. Table S4B. Proof of principle for the determination of non-pathogenic variants

Gene	cDNA	Protein	SNP_ID	SIFT	Polyphen	Mutpred	PhyloP	Classification predicitons	PhyloP Classification	Final vote
ABCA4	c.2828C>T	p.R943Q	rs1801581	Tolerated	Probably damaging	0.816	0.52	Pathogenic	Benign	Unknown
CACNA2D4	c.979T>C	p.I327V	rs10735005	Tolerated	Benign	0.546	3.93	Benign	Pathogenic	Unknown
CFH	c.184G>A	p.V62I	rs800292	Tolerated	Benign	0.58	1.46	Benign	Unknown	Benign
CFH	c.2808G>T	p.E936D	rs1065489	Tolerated	Possibly damaging	0.51	-0.57	Benign	Benign	Benign
CNGB3	c.702A>C	p.C234W	rs6471482	Tolerated	Benign	0.628	4.10	Benign	Pathogenic	Unknown
CNGB3	c.892T>G	p.T298P	rs4961206	Tolerated	Benign	0.653	0.92	Benign	Benign	Benign
CNGB3	c.919T>C	p.I307V	rs13265557	Tolerated	Benign	0.415	-1.05	Benign	Benign	Benign
CNGB3	c.2264T>C	p.E755G	rs3735972	Tolerated	Benign	0.122	1.06	Benign	Unknown	Benign
CYP4V2	c.775C>A	p.Q259K	rs13146272	Tolerated	Benign	0.511	1.33	Benign	Unknown	Benign
ELOVL4	c.895T>C	p.M299V	rs3812153	Tolerated	Benign	0.156	3.67	Benign	Pathogenic	Unknown
EYS	c.359G>A	p.T120M	rs12193967	Damaging	Unknown	0.723	-0.58	Pathogenic	Benign	Unknown
EYS	c.1712T>C	p.Q571R	rs61753610	Tolerated	Unknown	0.392	2.08	Benign	Unknown	Benign
EYS	c.1891C>T	p.G631S	rs9342464	Damaging	Unknown	0.877	0.84	Pathogenic	Benign	Unknown
EYS	c.1922T>A	p.E641V	rs17411795	Damaging	Unknown	0.623	0.44	Pathogenic	Benign	Unknown
EYS	c.2555A>G	p.L852P	rs9294631	Tolerated	Probably damaging	0.653	2.54	Pathogenic	Pathogenic	Pathogenic
EYS	c.3787T>C	p.I1263V	rs17404123	Tolerated	Benign	0.422	-0.59	Benign	Benign	Benign
EYS	c.6977C>T	p.R2326Q	rs4710457	Tolerated	Possibly damaging	0.54	0.77	Benign	Benign	Benign
EYS	c.7666T>A	p.S2556C	rs66462731	Tolerated	Probably damaging	0.828	1.23	Pathogenic	Unknown	Pathogenic
KCNV2	c.1597C>G	p.L533V	rs12352254	Tolerated	Benign	0.202	-0.52	Benign	Benign	Benign
LCA5	c.71A>G	p.L24S	rs2655655	Tolerated	Benign	0.101	1.80	Benign	Unknown	Benign
LCA5	c.77T>G	p.D26A	rs34068461	Damaging	Probably damaging	0.153	1.68	Unknown	Unknown	Unknown
LCA5	c.1967C>T	p.G656D	rs1875845	Tolerated	Benign	0.067	0.90	Benign	Benign	Benign
MERTK	c.353G>A	p.S118N	rs13027171	Tolerated	Benign	0.326	-0.90	Benign	Benign	Benign
MERTK	c.1397G>A	p.R466K	rs7604639	Tolerated	Benign	0.138	-1.06	Benign	Benign	Benign
MERTK	c.1552A>G	p.I518V	rs2230515	Tolerated	Benign	0.306	-0.83	Benign	Benign	Benign

PDE6B	c.121G>A	p.V320I	rs10902758	Tolerated	Benign	0.527	1.73	Benign	Unknown	Benign
PDE6C	c.808T>A	p.S270T	rs701865	Tolerated	Benign	0.42	2.13	Benign	Unknown	Benign
RP1	c.2953A>T	p.N985Y	rs2293869	Damaging	Possibly damaging	0.152	0.65	Unknown	Benign	Benign
RP1	c.5008G>A	p.A1670T	rs446227	Tolerated	Benign	0.073	-0.40	Benign	Benign	Benign
RP1	c.5071T>C	p.S1691P	rs414352	Tolerated	Benign	0.072	0.61	Benign	Benign	Benign
RP1	c.6098G>A	p.C2033Y	rs61739567	Tolerated	Benign	0.235	0.28	Benign	Benign	Benign
SPATA7	c.220G>A	p.V42M	rs3179969	Tolerated	Benign	0.091	0.63	Benign	Benign	Benign
TRPM1	c.95C>T	p.S32N	rs2241493	Tolerated	Benign	0.08	-0.62	Benign	Benign	Benign
TRPM1	c.3686T>G	p.N1229T	rs17227996	Tolerated	Benign	0.136	0.81	Benign	Benign	Benign
TRPM1	c.4183C>T	p.V1395I	rs3784588	Tolerated	Benign	0.076	0.55	Benign	Benign	Benign
TULP1	c.783C>G	p.K261N	rs2064318	Tolerated	Benign	0.071	2.28	Benign	Unknown	Benign

SIFT (1), **Polyphen** (2), **Mut pred** (3): prediction programs used for determination of pathogenicity. **PhyloP** (4): PhyloP score for evolutionary conservation, **Classification predicitons**: final classification based on prediction programs, **PhyloP Classification**: final classification based on PhyloP score, **Final vote**: Two classifications of the same category, or a combination of a category combined with an "unknown" classification result in a final vote of the respective category. The combination of a "pathogenic" and a "benign" classification results in a final "unknown" classification.

Reference List

1 Ng PC, Henikoff S. SIFT: Predicting amino acid changes that affect protein function. Nucleic Acids Res 2003; 31(13):3812-3814.

2 Adzhubei IA, Schmidt S, Peshkin L et al. A method and server for predicting damaging missense mutations. Nat Methods 2010; 7(4):248-249.

3 Li B, Krishnan VG, Mort ME et al. Automated inference of molecular mechanisms of disease from amino acid substitutions. Bioinformatics 2009; 25(21):2744-2750.

4 Pollard KS, Hubisz MJ, Rosenbloom KR, Siepel A. Detection of nonneutral substitution rates on mammalian phylogenies. Genome Res 2010; 20(1):110-121.

Supp. Table S5. Diagnostic results of 92 RP patients with validated variants

Patient	Gene	Mode of inheritance	Gender	Validated mutations	Segregation		Bioinformatic prediction	Conclusion
					in family	not in pedigree…		
7554	*ABCA4*	ar	F	M1 = c.1554G>A (p.(?))	Yes		Pathogenic	Pathogenic
	ABCA4	ar		M2 = c.4254-2A>G (p.(?))	Yes		Pathogenic	
	CA4	ad		M = c.869C>T (p.(P290L))	No		Benign	Benign
	RP2	xl		M = c.844C>T (p.(R282W))	No	9402	Benign	Benign
8322	*TOPORS*	ad	M	M = c.1730C>A (p.(S577Y))	Unknown		Pathogenic	Benign[5]
8625	*ELOVL4*	ad	F	M = c.800T>C (p.(I267T))	No	14705	Unknown	Benign
	IMPG2	ar		M1 = c.379G>A (p.(R127*))	Yes		Pathogenic	Pathogenic
	IMPG2	ar		M2 = c.3423-8_c.3423-5del (p.(?))	Yes		Pathogenic	
9398	*CA4*	ad	M	M = c.700G>A (p.(V234I))	Unknown		Benign	Benign
9402	*CC2D2A*	ar	M	M1 = c.685_687del (p.(E229del))[1]	Yes		Unknown	Unknown
	CC2D2A	ar		M2 = c.1810G>A (p.(G604S))	Yes		Pathogenic	
	CRB1	ar, ad		M = c.614T>C (p.(I205T))	No		Pathogenic	Benign[3]
	RP2	xl		M = c.844C>T (p.(R282W))	No	7554	Benign	Benign
	SEMA4A	ad		M = c.2138G>A (p.(R713Q))	Yes	11893, 21213, 28064, 31124, 33626	Benign	Benign
	TOPORS	ad		M = c.58C>T (p.(P20S))	No	9437, 9444	Benign	Benign
	USH2A	ar		M1 = c.688G>A (p.(V230M))	No		Unknown	Benign
	USH2A	ar		M2 = c.9959G>A (p.(G3320D))	No		Pathogenic	Benign
9437	*CRB1*	ar, ad	M	M = c.14A>G (p.(N5S))	No		Benign	Benign
	TOPORS	ad		M = c.58C>T (p.(P20S))	No	9402, 9444	Benign	Benign
	USH2A	ar		M1 = c.486-14G>A (p.(?))	Yes		Pathogenic	Pathogenic
	USH2A	ar		M2 = c.12729G>A (p.(W4243*))	Yes		Pathogenic	
9444	*ABCA4*	ar	M	M1 = c.5843C>T (p.(P1948L))	No		Unknown	Benign
	ABCA4	ar		M2 = c.6529G>A (p.(D2177N))	No		Pathogenic	Benign
	CRB1	ar, ad		M = c.484G>A (p.V162M))	No		Benign	Benign
	SEMA4A	ad		M = c.2044C>T (p.(P682S))	No	23224	Unknown	Benign
	TOPORS	ad		M = c.58C>T (p.(P20S))	No	9402, 9437	Benign	Benign
9470	*GUCA1B*	ad	M	M1 = c.465G>T (p.(E155D))	Unknown	31343	Benign	Benign
	GUCA1B	ad		M2 = c.475+8G>A (p.(?))	Unknown	13480, 14181	Benign	Benign
	RP2	xl		M = c.323G>A (p.(C108Y))	Unknown		Pathogenic	Pred Pathogenic
9472			M					

				(p.(?))				
	PRPH2	ad		M = c.424C>T (p.(R142W))	No		Pathogenic	Contributing[2]
	RGR	ar, ad		M = c.756+5A>G (p.(?))	No		Benign	Benign
	SNRNP200	ad		M = c.6340A>G (p.(M2114V))	No		Pathogenic	Benign
9493	*AHI1*	ar	M	M/M = c.2961+7_21delins20 (p.(?))	Unknown		Benign	Benign
	EYS	ar		M1 = c.4891C>T (p.(P1631S))	Unknown		Benign	Benign
	EYS	ar		M2 = c.7796A>G (p.(H2599R))	Unknown		Benign	
	PDE6B	ar, ad		M1 = c.1043_1044insCG (p.(A349fs))	Unknown		Pathogenic	Pathogenic
	PDE6B	ar, ad		M2 = c.1927_1969delinsGG (p.(N643fs))	Unknown		Pathogenic	
9506	*USH2A*	ar	M	c.[4976G>A;10561T>C] (p.[(R1659K);(W3521R)])	No		Pathogenic	Benign
	USH2A	ar			No		Unknown	
9509	*SEMA4A*	ad	F	M = c.2138G>A (p.(R713Q))	Unknown	11893, 21213, 28064, 31124, 33626	Benign	Benign
9511	*GUCY2D*	ar,ad	M	M = c.3225-7C>T (p.(?))	Unknown	21010	Benign	Benign
	RP1	ar, ad		M = c.4735T>G (p.(L1579V))	Unknown		Unknown	Unknown
9518	*RP1*	ar, ad	M	M/M = c.515T>G (p.(L172R))	Yes		Pathogenic	Pred Pathogenic
9520	*GUCA1A*	ad	F	M = c.149C>T (p.(P50L))	No		Unknown	Benign
	PCDH15	ar		M1 = c.4552insTTG (p.(1518insL))[1]	No		Unknown	Benign
	PCDH15	ar		M2 = c.3389-4C>T (p.(?))	No		Unknown	
9527	*MYO7A*	ar	M	M = c.[5560G>A;5156A>G] (p.[(Y1719C);(V1854M)])	No		Pathogenic	Benign
	MYO7A	ar			No		Unknown	
9537	*RP2*	xl		M = c.844C>T (p.(R282W))	Unknown	7554, 9402	Benign	Benign
9539	*GPR98*	ar	F	M = c.[8407G>A;12269C>A] (p.[(A2803T);(T4090N)])	Unknown	18336	Pathogenic	Benign
	GPR98	ar			Unknown	18336	Pathogenic	
9543	*RP1*	ad, ar	M	M = c.616-6T>C (p.(?))	No		Unknown	Benign
9552	*RP1*	ad, ar	F	M = c.616-6T>C (p.(?))	Unknown	9543	Unknown	Benign
11005	*GUCY2D*	ar, ad	M	M/M = c.154G>T (p.(A52S))	Unknown		Unknown	Benign[5]
	RPGR	xl		M = c.[223A>G;1579_1581del] (p.[(I75V);(Q526del)])[1]	Unknown		Benign	Benign
	RPGR	xl			Unknown	16430	Unknown	Benign
11319			M					

				(p.(R519Q))	n	16430		
	RP2	xl		M = c.844C>T (p.(R282W))	Unknown	7554, 9402	Benign	Benign
	RPGRIP1L	ar		M1 = c.972C>G (p.(C324W))	Unknown		Unknown	Unknown
	RPGRIP1L	ar		M2 = c.2240G>A (p.(R747Q))	Unknown		Unknown	
	PROM1	ar,ad		M = c.1034T>C (p.(V345A))	Unknown	22383	Pathogenic	Benign
	SNRNP200	ad		M = IVS31-9G>A (p.(?))	Unknown		Unknown	Unknown
11893	*CRB1*	ar, ad	M	M = c.2842+5G>A (p.(?))	No		Pathogenic	Benign[3]
	PDE6B	ar, ad		M = c.496G>A (p.(E166K))	No		Benign	Benign
	SEMA4A	ad		M = c.2138G>A (p.(R713Q))	No	21213, 28064, 31124, 33626	Benign	Benign
11919	*CACNA1F*	xl	F	M/M = c.1556G>A (p.(R519Q))	Unknown	14181, 16430	Benign	Benign
	TOPORS	ad		M = c.58C>T (p.(P20S))	Unknown	9402, 9437, 9444	Benign	Benign
13480	*GUCA1B*	ad	M	M = c.475+8G>A (p.(?))	No	14181	Benign	Benign
	RPE65	ar		M1 = c.208T>G (p.(F70V))	Yes		Pathogenic	Pred Pathogenic
	RPE65	ar		M2 = c.1102T>C (p.(Y368H))	Yes		Pathogenic	
14181	*CACNA1F*	xl	F	M = c.1556G>A (p.(R519Q))	No	16430	Benign	Benign
	GUCA1B	ad		M = c.475+8G>A (p.(?))	No	13480	Benign	Benign
	PDE6B	ar, ad		M = c.1107+3A>G (p.(?))	No		Pathogenic	Benign[3]
	EYS	ar		M/M = c.6045-4_6045-3del (p.(?))	Yes		Benign	Benign
14182	*SEMA4A*	ad	F	M = c.2138G>A (p.(R713Q))	Unknown	11893, 21213, 28064, 31124, 33626	Benign	Benign
14192	*TOPORS*	ad	M	M = c.c.58C>T (p.(P20S))	Unknown	9402, 9437, 9444	Benign	Benign
14267	*GUCA1B*	ad	M	M = c.475+8G>A (p.(?))	Unknown	13480, 14181	Benign	Benign
	PDE6B	ar, ad		M = c.1060-13G>A (p.(?))	Unknown		Unknown	Benign[3]
14428	*CACNA1F*	xl	F	M = c.811G>A (p.(G271R))	No		Pathogenic	Benign
	GPR98	ar		M1 = c.7582C>T (p.(P2528S))	No		Pathogenic	Benign
	GPR98	ar		M2 = c.[9607T>A;15440A>G] (p.[(S3203T);(D5147G)])	No		Pathogenic	
	GPR98	ar			No		Unknown	Benign
14705	*ELOVL4*	ad	F	M = c.800T>C (p.(I267T))	No	8625	Unknown	Benign
	PCDH15	ar		M/M = c.3733-19C>A (p.(?))	No		Benign	Benign
	RIMS1	ad		M = c.2699-8T>C (p.(?))	No		Unknown	Benign
	USH2A	ar		M1 = c.6257C>A (p.(T2086N))	No		Unknown	Benign
	USH2A	ar		M2 = c.11815G>A (p.(E3939K))	No		Pathogenic	
14812	*INVS*	ar	M	M =	No		Benign	Benign

	INVS	ar		c.[1948G>C;3138insTTC] (p.[(A650P);(S1046dup)])[1]	No		Unknown	
	PDE6B	ar, ad		M = c.3G>T (p.(M1?))	No		Pathogenic	Benign[3]
15569	*CACNA1F*	xl	F	M = c.1903G>A (p.(V635I))	Yes		Pathogenic	Benign[4]
	GPR98	ar		M = c.[3151G>T;3191A>C] (p.[(E1064A);(D1051Y)])	No		Pathogenic	Benign
	GPR98	ar			No		Unknown	
	PRPH2	ad		M = c.441del (p.(P147fs))	Yes		Pathogenic	Pathogenic
	SNRNP200	ad		M = c.1159A>G (p.(M387V))	Yes		Unknown	Benign[5]
16389	*SEMA4A*	ad	M	M = c.2044C>T (p.(P682S))	Unknown	9444	unknown	Benign
16430	*CACNA1F*	xl	F	M = c.1556G>A (p.(R519Q))	No	14181	Benign	Benign
	RPGR	xl		M = c.1579_1581del (p.(Q527del))[1]	No		Unknown	Benign
	SNRNP200	ad		M = c.5134-20T>C (p.(?))	No		Benign	Benign
17597	*CC2D2A*	ar	M	M/M = c.685_687del (p.(E229del))[1]	Yes		Unknown	Unknown
	CHM	xl		M = c.265A>T (p.(S89C))	No	28064, 18351	Benign	Benign
	SNRNP200	ad		M = c.5134-20T>C (p.(?))	No	16430	Benign	Benign
	USH2A	ar		M1 = c.10525A>T (p.(K3509*))	Yes		Pathogenic	Pred Pathogenic
	USH2A	ar		M2 = c.[12343C>T;13274C>T] (p.[(R4115C);(T4425M)])	Yes		Pathogenic	
	USH2A	ar			Yes		Pathogenic	
17792	*RHO*	ar, ad	F	M = c.403C>T (p.(R135W))	Unknown		Pathogenic	Pred Pathogenic
	RPGR	xl		M = c.1579_1581del (p.(Q526del))[1]	Unknown	16430	Unknown	Benign
18060	*SPATA7*	ar	F	M1 = c.3G>A (p.(M1?))	Unknown		Pathogenic	Pathogenic
	SPATA7	ar		M2 = c.322C>T (p.(R108*))	Unknown		Pathogenic	
18336	*GPR98*	ar	M	M = c.[8407G>A;12269C>A] (p.[(A2803T);(T4090N)])	No		Pathogenic	Pred Pathogenic
	GPR98	ar			No		Pathogenic	
	PDE6B	ar, ad		M1 = c.[1401+4_1401+16delins14; 2326G>A] (p.[(?);(D776N)])	Yes		Unknown	Pred Pathogenic
	PDE6B	ar, ad			Yes		Pathogenic	
	PDE6B	ar, ad		M2 = c.1927_1969delinsGG (p.(N643fs))	Yes		Pathogenic	
18351	*CHM*	xl	F	M = c.265A>T (p.(S89C))	No	17597, 28064	Benign	Benign
	GUCY2D	ar, ad		M/M = c.154G>T (p.(A52S))	Yes		Unknown	Benign[5]
	SNRNP200	ad		M = c.3005A>G (p.(N1002S))	No		Pathogenic	Benign

18390	*ABCA4*	ar	F	M/M = c.5843C>T (p.(P1948L))	Unknown		Unknown	Benign[5]
	GUCA1B	ad		M = c.465G>T (p.(E155D))	Unknown	31343	Benign	Benign
	RP2	xl		M = c.844C>T (p.(R282W))	Unknown	7554, 9402	Benign	Benign
18777	*C2ORF71*	ar	F	M = c.2600C>T (p.(P867L))	Unknown		Benign	Benign
	SEMA4A	ad		M = c.2138G>A (p.(R713Q))	Unknown	11893, 21213, 28064, 31124, 33626	Benign	Benign
	SNRNP200	ad		M/M = c.4165G>A (p.(V1389I))	Unknown		Unknown	Unknown
19081	*CA4*	ad	F	M = c.700G>A (p.(V234I))	No		Benign	Benign
	PDE6B	ar, ad		M = c.1060-13G>A (p.(?))	No		Unknown	Benign
	TOPORS	ad		M = c.1730C>A (p.(S577Y))	Yes		Pathogenic	Benign[5]
19531	*CHM*	xl	F	M = c.265A>T (p.(S89C))	Unknown	17597, 18351, 28064	Benign	Benign
19689	*CA4*	ad	F	M = c.700G>A (p.(V234I))	Unknown		Benign	Benign
	GUCA1B	ad		M = c.475+8G>A (p.(?))	Unknown	13480, 14181	Benign	Benign
	RGR	ar, ad		M = c.756+5A>G (p.(?))	Unknown	9472	Benign	Benign
19693	*PDE6B*	ar, ad	M	M1 = c.299G>A (p.(R100H))	Unknown		Pathogenic	Pathogenic
	PDE6B	ar, ad		M2 = c.1927_1969delinsGG (p.(N643fs))	Unknown		Pathogenic	
19733	*USH2A*	ar	F	M1 = c.[4957C>T;7379G>A] (p.[(R1653*);(R2460H)])	Yes		Pathogenic	Pred Pathogenic
	USH2A	ar			Yes		Pathogenic	
	USH2A	ar		M2 = c.10073G>A (p.(C3358Y))	Yes		Pathogenic	
19735	*RPE65*	ar	M	M1 = c.271C>T (p.(R91W))	Yes		Pathogenic	Pred Pathogenic
	RPE65	ar		M2 = c.715T>G (p.(Y239D))	Yes		Pathogenic	
	TOPORS	ad		M = c.58C>T (p.(P20S))	No	9402, 9437, 9444	Benign	Benign
20703	*ABCA4*	ar	M	M = c.[2828G>A;6764G>T] (p.[(R943Q);(S2255I)])	Yes		Benign	Benign
	ABCA4	ar			Yes		Benign	
	GUCY2D	ar, ad		M = c.3225-7C>T (p.(?))	No	21010	Benign	Benign
	NPHP3	ar		M1 = c.154G>A (p.(A52T))	No		Benign	Benign
	NPHP3	ar		M2 = c.944A>T (p.(D315V))	No		Pathogenic	
	PDE6B	ar, ad		M = c.801C>A (p.(Y267*))	No		Pathogenic	Benign
	RP9	ad		M = c.629A>G (p.(K210R))	No		Unknown	Benign
20984	*PDE6B*	ar, ad		M/M = c.1107+3A>G (p.(?))	Yes		Pathogenic	Pathogenic

21010	*ABCA4*	ar	M	M = c.[1522C>T;5381C>A] (p.[(R508C);(A1794D)])	No		Pathogenic	Benign
	ABCA4	ar			No		Pathogenic	
	GUCY2D	ar, ad		M = c.3225-7C>T (p.(?))	No		Benign	Benign
	EYS	ar		M = c.3444-5C>T (p.(?))	No		Benign	Benign
21017	*GUCA1A*	ad	M	M = c.598G>A (p.(A200T))	No		Unknown	Benign
21141	*CDH23*	ar	M	M = c.[3074G>A;4846-19G>C] (p.[(G1025D);(p.(?)])	No		Probably Pathogenic	Benign
	CDH23	ar			No		Benign	Benign
	CDH23	ar		M = c.5419G>A (p.(V1807M))	No		Unknown	Benign
	CEP290	ar		M1 = c.3559del (p.(L1187fs))	Yes		Pathogenic	Pathogenic
	CEP290	ar		M2 = c.4705-1G>T (p.(?))	Yes		Pathogenic	
	CRX	ar, ad		M = c.472G>A (p.(A158T))	No		Unknown	Benign
	RIMS1	ad		M = c.5053A>G (p.(T1685A))	No		Unknown	Benign
21213	*CACNA1F*	xl	F	M = c.1546G>C (p.(R516G))	No		Pathogenic	Benign
	SEMA4A	ad		M = c.2138G>A (p.(R713Q))	No	11893, 28064, 31124, 33626	Benign	Benign
	USH2A	ar		M1 = c.10448G>A (p.(G3483E))	No		Pathogenic	Benign
	USH2A	ar		M2 = c.10561T>C (p.(W3521R))	No		Pathogenic	Benign
21334	*CACNA1F*	xl	M	M = c.220T>C (p.(C74R))	Yes		Pathogenic	Pred Pathogenic
	PDE6B	ar, ad		M = c.655T>C (p.(Y219H))	No	27585	Benign	Benign
21384	*ABCA4*	ar	F	M1 = c.3191-2_3191del (p.(?))	Yes		Pathogenic	Benign
	ABCA4	ar		M2 = c.5603A>T (p.(N1868I))	Yes		Benign	
	GPR98	ar		M1 = c.[3151G>T;3191A>C] (p.[(D1051Y);(E1064A)])	No		Pathogenic	Benign
	GPR98	ar			No		Unknown	
	GPR98	ar		M2 = c.9650C>T (p.(A3217V))	No		Unknown	
21933	*GUCY2D*	ar, ad	M	M = c.154G>T (p.(A52S))	Unknown	31343	Unknown	Benign
	PDE6B	ar, ad		M1 = c.655T>C (p.(Y219H))	Unknown		Benign	Benign
	PDE6B	ar, ad		M2 = c.2503+5G>C (p.(?))	Unknown		Pathogenic	
	RP1	ar, ad		M1 = c.368_369dup (p.(P214fs))	Unknown		Pathogenic	Pathogenic
	RP1	ar, ad		M2 = c.4241_4242del (p.(H1414fs))	Unknown		Pathogenic	
	SEMA4A	ad		M1 = c.463-17C>A (p.(?))	Unknown		Unknown	Unknown
	SEMA4A	ad		M2 = c.2138G>A (p.(R713Q))	Unknown	11893, 21213, 28064, 31124, 33626	Benign	Benign

22312	*GUCY2D*	ar, ad	M	M = c.1724C>T (p.(P575L))	Yes		Pathogenic	Benign[5]
	MYO7A	ar		M1 = c.4589C>T (p.(S1530L))	No		Benign	Benign
	MYO7A	ar		M2 = c.6640G>A (p.(G2214S))	No		Benign	
22315	*AIPL1*	ar, ad	M	M = c.937C>A (p.(A313S))	No		Unknown	Benign
	RHO	ar, ad		M = c.538C>T (p.(P180S))	Yes		Pathogenic	Pred Pathogenic
22383	*GUCY2D*	ar, ad	M	M = c.3225-7C>T (p.(?))	No	21010	Benign	Benign
	PROM1	ar, ad		M = c.1034T>C (p.(V345A))	No		Pathogenic	Benign
	RPGR	xl		M = c.1367A>G (p.(Q456R))	Yes		Benign	Benign
	TOPORS	ad		M = c.58C>T (p.(P20S))	No	9402, 9437, 9444	Benign	Benign
	USH2A	ar		M/M = c.1434G>C (p.(E478D))	Yes		Unknown	Unknown
22393	*CACNA1F*	xl	M	M = c.1546C>G (p.(R516G))	Unknown	21213 (NA father is carrier)	Pathogenic	Benign
	PDE6B	ar, ad		M/M = c.892C>T (p.(Q298*))	Unknown		Pathogenic	Pathogenic
22731	*CEP290*	ar	F	M1 = c.1825-14_1825-13del (p.(?))	Unknown		Benign	Benign
	CEP290	ar		M2 = c.1991A>G (p.(D664G))	Unknown		Pathogenic	
	SEMA4A	ad		M = c.2138G>A (p.(R713Q))	Unknown	11893, 21213, 28064, 31124, 33626	Benign	Benign
22777	*USH2A*	ar	F	M1 = c.1256G>T (p.(C419F))	Unknown		Pathogenic	Pred Pathogenic
	USH2A	ar		M2 = c.[12343C>T;13274C>T] (p.[(R4115C);(T4425M)])	Unknown		Pathogenic	
	USH2A	ar			Unknown		Pathogenic	
23224	*CRB1*	ar, ad	M	M = c.135C>G (p.(C45W))	No	27585	Pathogenic	Benign[3]
	PDE6B	ar, ad		M1 = c.655T>C (p.(Y219H))	Yes		Benign	Benign
	PDE6B	ar, ad		M2 = c.[1401+4C>T;2326G>A] (p.[(?);(D776N)])	Yes		Unknown	
	PDE6B	ar, ad			Yes		Pathogenic	
	SEMA4A	ad		M = c.2044C>T (p.(P682S))	No	9444	Unknown	Benign
27528	*CRB1*	ar, ad	M	M = c.71-12A>T (p.(?))	Unknown		Benign	Benign
	GUCA1B	ad		M = c.476-9C>G (p.(?))	Unknown		Benign	Benign
	SNRNP200	ad		M = c.575-7G>T (p.(?))	Unknown		Benign	Benign
27585	*C2ORF71*	ar	M	M = c.3671_3673dup (p.(S1225dup))[1]	No		Unknown	Benign
	CACNA2D4	ar		M1 = c.782-19C>T (p.(?))	No		Benign	Benign
	CACNA2D4	ar		M2 = c.2793-15G>A (p.(?))	No		Benign	Benign
	CRB1	ar, ad		M = c.135C>G (p.(C45W))	No		Pathogenic	Benign
	PDE6B	ar, ad		M = c.655T>C (p.(Y219H))	No	21334	Benign	Benign

27790	*PROM1*	xl	M	M = c.524A>C (p.(Y175S))	No		Pathogenic	Benign
	PRPF31	ad		M = c.553G>T (p.(E185*))	Yes		Pathogenic	Pathogenic
28064	*CHM*	xl	M	M = c.265A>T (p.(S89C))	No	17597, 18351, 28064	Benign	Benign
	RPGR	xl		M = c.1793_1794insT (p.(N599fs))	No		Pathogenic	Benign
	SEMA4A	ad		M = c.2138G>A (p.(R713Q))	No	11893, 21213, 31124, 33626	Benign	Benign
28557	*SEMA4A*	ad	F	M = c.2138G>A (p.(R713Q))	Unknown	11893, 21213, 28064, 31124, 33626	Benign	Benign
	USH2A	ar		M1 = c.2276G>T (p.(C759F))	Unknown		Pathogenic	Pred Pathogenic
	USH2A	ar		M2 = c.5576T>G (p.(F1859C))	Unknown		Pathogenic	
28692	*GUCY2D*	ar, ad	F	M = c.3225-7C>T (p.(?))	Unknown	21010	Benign	Benign
	GPR98	ar		M1 = c.4864T>C (p.(Y1622H))	Unknown		Unknown	Unknown
	GPR98	ar		M2 = c.6229G>A (p.(E2077K))	Unknown		Unknown	
	USH2A	ar		M1 = c.1434G>C (p.(E478D))	Unknown		Unknown	Benign
	USH2A	ar		M2 = c.14543G>A (p.(R4848Q))	Unknown		Benign	
29883	*GUCY2D*	ar, ad	M	M = c.2165G>A (p.(R722Q))	No		Unknown	Benign
31035	*ABCA4*	ar	M	M = c.[2828G>A;6764G>T] (p.[(R943Q);(S2255I)])	No		Benign	Benign
	ABCA4	ar			No		Benign	Benign
	AIPL1	ar, ad		M = c.932G>C (p.(R311L))	No	32594	Pathogenic	Benign
	GUCY2D	ar, ad		M = c.61T>C (p.(W21R))	No		Benign	Benign
	NR2E3	ar, ad		M = c.95G>A (p.(W32*))	Yes		Pathogenic	Pathogenic
31124	*ABCA4*	ar	M	M1 = c.635G>A (p.(R212H))	No		Benign	Benign
	ABCA4	ar		M2 = c.2701A>G (p.(T901A))	No		Unknown	
	SEMA4A	ad		M = c.2138G>A (p.(R713Q))	No	11893, 21213, 28064, 33626	Benign	Benign
	USH2A	ar		M1 = c.486-14G>A (p.(?))	Yes		Pathogenic	Pred Pathogenic
	USH2A	ar		M2 = c.2276G>T (p.(C759F))	Yes		Pathogenic	
31343	*GUCA1B*	ad	F	M = c.465G>T (p.(E155D))	No		Benign	Benign
	GUCY2D	ar, ad		M = c.154G>T (p.(A52S))	No		Unknown	Benign
	NYX	xl		M = c.1394G>T (p.(C465F))	No		Benign	Benign
	USH2A	ar		M1 = c.917_918insGCTG (p.(S307fs))	Yes		Pathogenic	Pred Pathogenic
	USH2A	ar		M2 = c.11007C>A (p.(S3669R))	Yes		Pathogenic	

31494	*CHM*	xl	F	M = c.265A>T (p.(S89C))	Unknown	17597, 18351, 20864	Benign	Benign
	CRX	ar, ad		M = c.196G>A (p.(V66I))	Unknown		Unknown	Benign[5]
	RPGR	xl		M = c.[1367A>G;1579_1581del] (p.[(Q456R);(Q527del)])[1]	Unknown		Benign	Benign
	RPGR	xl			Unknown	16430	Unknown	
31723	*ARL6*	ar	F	M/M = c.185+1G>A (p.(?))	Yes		Pathogenic	Pathogenic
	IMPDH1	ad		M = c.1030C>T (p.(R344C))	No		Pathogenic	Benign
	RPGRIP1	ar		M = c.574A>G (p.(K192E))	No		Benign	Benign
31910	*EYS*	ar	F	M/M = c.2309A>C (p.(Q770P))	Yes		Unknown	Unknown
31933	*CRB1*	ar, ad	M	M1 = c.1602G>T (p.(K534N))	Unknown		Pathogenic	Pred Pathogenic
	CRB1	ar, ad		M2 = c.2234C>T (p.(T745M))	Unknown		Pathogenic	
31994	*AIPL1*	ar, ad	M	M = c.286G>A (p.(V96I))	Unknown		Benign	Benign
	RHO	ar, ad		M = c.641T>A (p.(I214N))	Unknown		Pathogenic	Pred Pathogenic
32111	*GUCY2D*	ar, ad	F	M = c.154G>T (p.(A52S))	Unknown	31343	Unknown	Benign
	USH2A	ar		M = c.486-13G>A (p.(?))	No		Pathogenic	Benign
	USH2A	ar		M = c.6236A>C (p.(K2079T))	No		Unknown	Benign
	USH2A	ar		M = c.6240G>T (p.(K2080N))	No		Unknown	Benign
	USH2A	ar		M = c.12445T>C (p.(W4149R))	No		Pathogenic	Benign
32594	*AIPL1*	ar, ad	M	M = c.932G>T (p.(R311L))	No		Pathogenic	Benign
	C2ORF71	ar		M1 = c.[755C>A;3265_3267del] (p.[(A252D);(P1089del)])[1]	No		Unknown	Benign
	C2ORF71	ar		M2 = c.3265_3267del (p.(P1089del))[1]	No		Unknown	
	CFH	ar		M1 = c.2669G>T (p.(S890I))	No		Benign	Benign
	CFH	ar		M2 = c.3019G>T (p.(V1007L))	No		Benign	
	GPR98	ar		M = c.[9607T>A;15440A>G] (p.[(S3203T);(D5147G)])	No		Pathogenic	Benign
	GPR98	ar			No		Unknown	
	NRL	ar		M1 = c.508C>A (p.(R170S))	Yes		Pathogenic	Pred Pathogenic
	NRL	ar		M2 = c.654del (p.(C219fs))	Yes		Pathogenic	
32655	*BEST1*	ar		M = c.535-7_535-6insCTC (p.(?))	No		Unknown	Benign
	RP2	xl		M = c.318_319delAG (p.(D107fs))	Yes		Pathogenic	Pathogenic
32825	*GUCY2D*	ar, ad		M = c.2101C>T (p.(P701S))	Unknown		Benign	Benign

33626	*SEMA4A*	ad	F	M = c.2138G>A (p.(R713Q))	No	11893, 21213, 28064, 31124	Benign	Benign
	USH2A	ar		M1 = c.1227G>A (p.(W409*))	Yes		Pathogenic	Pred Pathogenic
	USH2A	ar		M2 = c.12575G>A (p.(R4192H))	Yes		Unknown	
33672	*RDH12*	ar		M/M = c.658+591_*603delinsCT (p.(?))	Yes		Pathogenic	Pathogenic
33884	*CA4*	ad	F	M = c.700G>A (p.(V234I))	Unknown		Benign	Benign
	ELOVL4	ad		M = c.800T>C (p.(I267T))	Unknown	8625, 14705	Unknown	Benign
34624	*CHM*	xl	M	M = c.265A>T (p.(S89C))	Unknown	28064	Benign	Benign
34886	*GUCY2D*	ar, ad	F	M = c.154G>T (p.(A52S))	Unknown	31343	Unknown	Benign
	RP1	ar, ad		M = c.1118C>T (p.(T373I))	Unknown		Benign	Benign
36832	*PRPF31*	ad	F	M = c.910C>T (p.(R304C))	No		Pathogenic	Benign
37360	*GUCY2D*	ar, ad	F	M = c.1571C>T (p.(A524V))	Unknown		Benign	Benign
	RP1	ar, ad		M = c.4250T>C (p.(L1417P))	Unknown		Unknown	Benign[5]
37370	*BEST1*	ad, ar	F	M = c.1699C>T (p.(L567F))	No		Benign	Benign
	CRB1	ar, ad		M = c.614T>C (p.(I205T))	No	9402	Pathogenic	Benign[3]
	RLBP1	ar		M/M = c.525_945del (p.(?))	Yes		Pathogenic	Pathogenic
	NPHP4	ar		M1 = c.2902G>A (p.(A968T))	Yes		Benign	Benign
	NPHP4	ar		M2 = c.3385G>A (p.(V1129M))	Yes		Unknown	
41611	*TRPM1*	ar	F	M1 = c.2647G>A (p.(E883K))	Yes		Pathogenic	Unknown
	TRPM1	ar		M2 = c.3841A>G (p.(E1281K))	Yes		Unknown	

Patient: patient identifier. **Gene**: RefSeq gene name of the gene in which mutations were identified. **Inheritance**: known inheritance of phenotype for known mutations. **Gender**: patient gender. **Validated Mutations**: cDNA and protein notation of the identified mutations. **Segregation in Family**: whether the variant segregates with the disease within the family. **Segregation in other pedigrees**: patient identifier of other patients in which the mutation was identified. **Bioinformatic prediction**: *in silico* prediction whether the mutation is likely benign, pathogenic or unknown (see Material and Methods). **Conclusion:** final assessment whether the mutation is pathogenic based on the overall mutational load. **Pred Pathogenic**: Conclusion for pathogenicity of variants that are based on Prediction for at least one variant. [1]: due to specific features of the bioinformatic prediction tools that we have used, it was not possible to systematically assess the pathogenicity of single amino acid insertions, -deletions or –duplications. Hence, these variants are predicted to have an unknown effect. [2]: known mutation, not present in siblings but potentially contributing to phenotype, [3]: single heterozygous mutations in *PDE6B* and *CRB1* are, although predicted to be potentially pathogenic, considered benign since for each gene, only 1 case of dominant inheritance has been reported. [4]: Considered benign in this female patient as the variant is X-linked. [5]: Although these variants are predicted to be pathogenic or unknown by our pipeline, we realized their presence in the Exome Variant Server (http://evs.gs.washington.edu/EVS/, Release Version: v.0.0.10) at frequencies to high for variants in genes with the respective inhertitance, leading to the final conclusion that these variants are probably benign. Nucleotide numbering reflects cDNA numbering with +1 corresponding to the A of the ATG translation initiation codon in the reference sequence, according to journal guidelines (www.hgvs.org/mutnomen). The initiation codon is codon 1.

Supp. Table S6. Clinical characteristics of all RP patients

Proband	Gender	Age of onset RD (yrs)	Retinal phenotype	Extra-ocular abnormalities	(Probable) causative gene	Unknown
7554*	F	7	CRD	-	*ABCA4*	
8322	M	38	RP	-		
8625	F	16	RP	-	*IMPG2*	
9398	M	20	RP	Hearing loss		
9402	M	1st decade	RP	-		*CC2D2A*
9437**	M	20	RP	Hearing loss	*USH2A*	
9444	M	20	RP	-		
9470	M	22	RP	-	*RP2*	
9472	M	6	RP	-	*PDE6B*	
9493	M	unknown	RP	-	*PDE6B*	
9506	M	15	RP	Congenital hearing loss		
9509	F	unknown	RP	Unilateral hearing loss		
9511	M	35	RP	-		*RP1*
9518	M	unknown	RP	-	*RP1*	
9520	F	25	RP	-		
9527	M	unknown	RP	-		
9528	M	26	RP	-		
9537	F	31	RP	-		
9539	M	unknown	RP	-		
9543	M	unknown	RP	-		
9552	F	36	RP	-		
11005	M	37	RP	-		
11319	M	unknown	RP	-		*RPGRIP1L, SNRNP200*
11893	M	68	RP	-		
11919	F	25	RP	-		
13023	F	49	RP	Neurosensory hearing loss		
13480	M	unknown	RP	-	*RPE65*	
14181	F	unknown	RP	-		
14182	F	25	RP	-		
14192	M	29	RP	-		
14267	M	unknown	RP	-		
14428	F	unknown	RP	-		
14705	F	35	RP	-		
14812	M	unknown	RP	-		
15569	F	50	RP	-	*PRPH2*	
16389	M	unknown	Secundary photoreceptor damage due to uveïtis ODS	-		

16430	F	15	RP	-		
17593	M	unknown	RP			
17597	F	44	RP	-	*USH2A*	
17792	F	unknown	RP		*RHO (potentially de novo)*	
18060	F	9	RP	-	*SPATA7*	
18130	F	unknown	RP	-		
18336	M	27	RP	-	*PDE6B*	
18351	F	16	RP	-		
18390	F	10	Juvenile MD			
18777	F	5	Early-onset RP	Mild intellectual disability, obesitas		*SNRNP200*
19081	F	unknown	RP	-		
19531	F	8	RP			
19689	F	unknown	RP	-		
19693	M	12	RP	-	*PDE6B*	
19733	F	61	RP	-	*USH2A*	
19735	F	Childhood	LCA / EOSRD	Tinnitus: BAE normal	*RPE65*	
20703	M	4	RP	Wilm's tumor		*ABCA4*
20984	F	10	RP	-	*PDE6B*	
21010	M	6	EOSRD	-		
21017	M	6	EOSRD	-		
21141**	M	Congenital	LCA / EOSRD	(Mild) intellectual disability	*CEP290*	
21213	F	50	RP	-		
21334*	M	Congenital	CSNB	-	*CACNA1F*	
21384	F	9	RP	-		
21642	F	20	RP	-		
21933	M	unknown	RP	Hypertension	*RP1*	*SEMA4A*
22312	M	15	RP	-		
22315	M	10	RP	-	*RHO (de novo)*	
22383	M	16	RP	-		*USH2A*
22393	M	10	RP	Age-related mild hearing loss, psoriasis	*PDE6B*	
22731	F	43	RP	Diabetes mellitus, hypertension, hearing loss		
22777	F	39	RP	-	*USH2A*	
23224	M	7	RP	-		
23422	F	28	RP	-		
27528	M	11	RP	-		
27585	M	unknown	RP	-		
27790	M	18	RP	-	*PRPF31 (de novo)*	
28064	M	45	RP	Mantle cell lymphoma		
28557	F	35	RP	-	*USH2A(one allele de novo)*	

28692	F	58	RP	Malignant hypertension with renal failure, followed by transplant		
29883	M	18	RP	-		
30364	M	26	RP	-		
31035	M	45	RP	-	*NR2E3 (potentially de novo)*	
31124	M	29	RP	-	*USH2A*	
31343	F	34	RP	Epilepsy	*USH2A*	
31494	F	34	RP	Subtle hearing loss, fibromyalgia		
31723**	F	9	RP	Obesity, familial hypercholesterolemia, recurrent cystitis (5 yrs), heart murmur	*ARL6*	
31910	F	58	RP	Cardiomyopathy (hypertrophic)		*EYS*
31933	M	19	RP	-	*CRB1*	
31994	M	40	RP	Secundary diabetes mellitus type 1 (due to pancreatitis)	*RHO*	
32111	F	58	RP	Diabetes mellitus type 2		
32594	M	1	Early-onset CPRD	Ferriprive anemia	*NRL*	
32655	M	4	EOSRD	-	*RP2*	
32825	F	28	RP	-		
33626	F	29	RP	-	*USH2A*	
33672	M	26	RP	-	*RDH12*	
33884	F	33	RP	-		
34624	M	Childhood	RP	-		
34886	F	unknown	RP	Hyperornithemia		
36832	F	65	RP	-		
37360	M	18	RP	-		
37370	F	4	EOSRD	-	*RLBP1*	
38290	F	8	RP	-		
41611	F	unknown	RP	-		*TRPM1*

*****Although these patients were initially diagnosed with RP, clinical reassessment upon identification of the genetic causes led to a re-appreasal of the retinal phenotype as indicated in column D. ****** Upon identification of the causative gene, patients were diagnosed with extra-ocular features previously reported to be associated with the corresponding genes. **Abbreviations**: **CPRD**: clumped pigmentary retinal degeneration; **CSNB**: congenital stationary night blindness; **EOSRD**:early-onset severe retinal degeneration; **LCA**: Leber congenital amaurosis; **MD**; macular dystrophy; **RP**; retinitis pigmentosa.
